# Lead-Structure-Based Rigidization Approach to Optimize SirReal-Type Sirt2 Inhibitors

**DOI:** 10.3390/molecules30081728

**Published:** 2025-04-11

**Authors:** Matthias Frei, Thomas Wein, Franz Bracher

**Affiliations:** Department of Pharmacy—Center for Drug Research, Ludwig-Maximilians University, Butenandtstr. 5–13, 81377 Munich, Germany; matthias.frei@cup.uni-muenchen.de (M.F.); thomas.wein@cup.uni-muenchen.de (T.W.)

**Keywords:** sirtuin 2 inhibitor, Sirt2 inhibitor, SirReal2, structure–activity relationship, rigidization

## Abstract

Sirtuins are involved in cellular processes in multiple ways. Therefore, the development of potent and selective Sirt2 inhibitors provides an important contribution to understanding physiological and pathophysiological mechanisms, particularly for the research and treatment of cancer and neurodegenerative diseases. Based on established SirReal-type lead inhibitors, further selective Sirt2 inhibitors were synthesized in a docking-guided rigidization approach, and the knowledge regarding requirements and properties of the Sirt2-binding pocket was expanded by means of a comprehensive SAR study. Naphthalene derivative **FM69** emerged from the screening as the most potent rigidized inhibitor, which, with an IC_50_ value of 0.15 µM against Sirt2, represents a promising foundation for the further development of novel potent and selective Sirt2 inhibitors based on the presented rigidization strategy.

## 1. Introduction

Histone deacetylases (HDACs) are enzymes that are involved in the post-translational epigenetic regulation of histone modification and thus play a fundamental role in physiological and pathophysiological processes [1,2]. Besides the general classification into four main classes (I–IV), different catalysis mechanisms allow a further characterization into zinc-dependent (Class I, II, and IV) and NAD^+^-dependent histone deacetylases (Class III, sirtuins) [3]. Vorinostat, a zinc-chelating hydroxamic acid derivative, is the first FDA-approved histone deacetylase inhibitor to be successfully used in the treatment of cancer [4].

**Figure 1:**
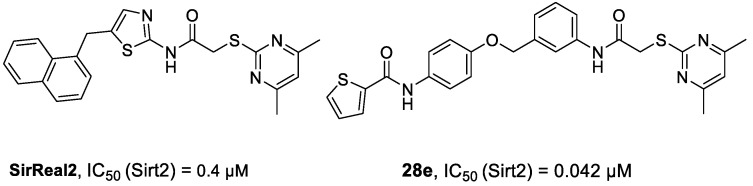
Highly potent and selective SirReal-type Sirt2 inhibitors: chemical structures and inhibitory potency of first-generation inhibitor **SirReal2** and its corresponding structural optimization **28e**.

Further drug approvals targeting zinc-dependent HDACs followed, while the evaluation of drugs targeting sirtuin remains a focus of ongoing research and development [5,6]. Furthermore, Sirt2 is not limited to histones but also targets a range of non-histone substrates, such as α-tubulin, p53, α-synuclein, FOXO3a, CDK9, and NF-_Κ_B as well, showing the comprehensive involvement of Sirt2 in various cellular processes [7]. The inhibition of Sirt2 is associated with promising neuroprotective, anticarcinogenic, and anti-inflammatory effects; therefore, the development of highly potent and selective inhibitors of this subtype provides an important contribution to the understanding and treatment of diseases such as cancer, Alzheimer’s disease, Huntington’s disease, and Parkinson’s disease [8,9,10,11,12]. Throughout time, several potent and selective Sirt2 inhibitors based on different chemical scaffolds were reported, providing foundation for sound structure–activity relationship analysis and further improvements [13]. The discovery of the SirReal-type inhibitors, in particular, **SirReal2** (IC_50_ = 0.4 µM, Sirt2), by Rumpf et al. in 2015 [14] and corresponding further developments by Schiedel et al. [15], represents a major impact on insights into structure–activity relationships of potent and selective Sirt2 inhibitors. Based on **SirReal2**, Yang et al. presented a highly potent and selective Sirt2 inhibitor **28e** (IC_50_ = 0.042 µM) with more extensive structural changes replacing the characteristic 5-(arylmethyl)thiazole structure [16] (Figure 1).

During the binding process, the SirReal (sirtuin-rearranging ligand)-type inhibitors induce the formation of a so termed selectivity pocket, which is accommodated by the respective selectivity pocket binder structure motif, a 2-((4,6-dimethylpyrimidin-2-yl)thio)acetamide moiety [17]. Thereby, the benzyl-ether-mediated spatial orientation of the thiophenecarboxamide of **28e** enables advantageous interactions in the Sirt2 substrate channel pocket and contributes significantly to an increased inhibitory activity [16].

Consequently, we concluded that the geometric angulation of the molecule caused by the ether group plays an essential role, the specific orientation of which could be fixed by means of docking-guided rigidization to improve the inhibitory activity of lead structure **28e**.

Rigidization is an established medicinal chemistry approach that restricts the spatial degrees of the freedom of a ligand to reduce the entropic disadvantage of a flexible molecule by pre-organizing it for advantageous binding processes [18]. Successful examples have been reviewed by Fang et al. in 2014 [19]. More recent examples deal with small molecules targeting survinin [20], kinases [21], 5-HT receptors [22,23], and tryptophan 2,3-dioxygenase [24].

In this study, we aimed to enhance the inhibitory activity of **28e** against Sirt2 through a docking-guided structural rigidization approach, where various conformationally restricted analogues will be evaluated for their selectivity and potency towards Sirt2. The envisaged target molecules contain biaryl motifs, which should allow convenient synthetic access via palladium-catalyzed cross-coupling reactions, and were further expected to show enhanced metabolic stability.

## 2. Results

### 2.1. Initial Rigidization Strategy and Underlying Docking Simulations

**Figure 2:**
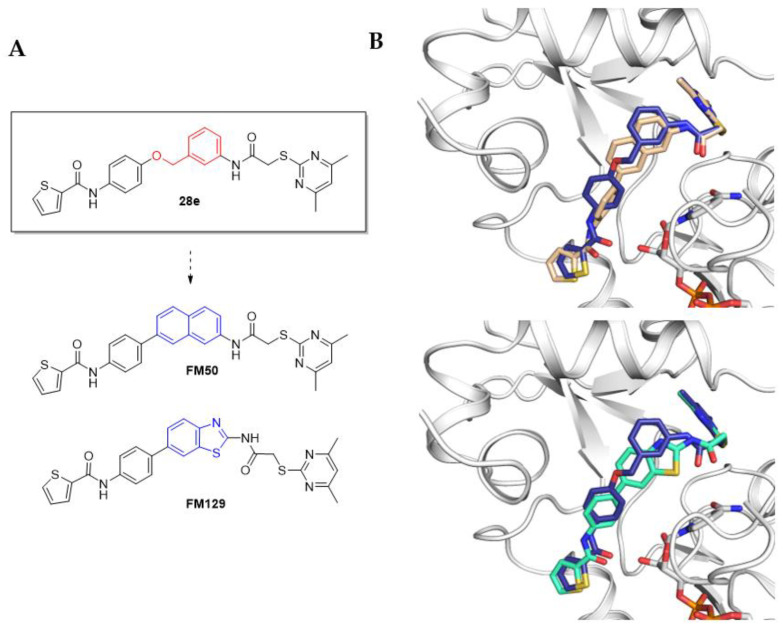
(**A**) General rigidization approach of lead compound **28e**. The benzyl ether structure (in red) of **28e** is replaced by rigid elements such as naphthalene (**FM50**) and benzothiazole (**FM129**) (both in blue), providing the first two envisaged rigidized target compounds. (**B**) Docking pose of lead compound **28e** (blue, based on PDB ID: 5YQO prediction), superimposed with naphthalene-based rigid candidate **FM50** (creme) and benzothiazole-based rigid candidate **FM129** (green). The position of cofactor NAD^+^ is visualized by overlay with PDB ID: 4RMG.

At first glance, the rigidization of lead structure **28e** can be achieved while maintaining the desired spatial orientation by replacing the benzyl ether structure with a naphthalene ring. By using a benzothiazole instead, a further rigid element was investigated, which, with a slightly different spatial orientation and with reference to the aminothiazole-containing **SirReal2**, should serve for further structural diversity regarding SAR analyses (Figure 2).

**Figure 3:**
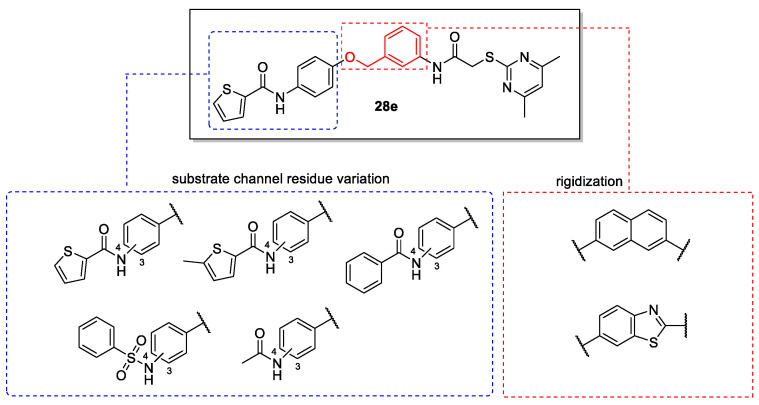
Comprehensive overview of the envisaged compound library, developed within the framework of the rigidization approach of lead compound 28e. Thereof, 10 naphthalene-based and 10 benzothiazole-based rigid target candidates are defined.

Based on these encouraging docking results, further structural modifications were included in the investigation. These modifications were inspired by previous evidence on SAR from the synthesis efforts of Yang et al. [16]. Therefore, different amide variants of the thiophene-2-carboxamide residue were attached in *meta*- and *para*-position to the phenyl ring connected with the rigid element (naphthalene or benzothiazole), and all hybrids (Figure 3) were evaluated for their sirtuin-inhibitory activity and toxicity.

### 2.2. Chemistry

**Scheme 1:**
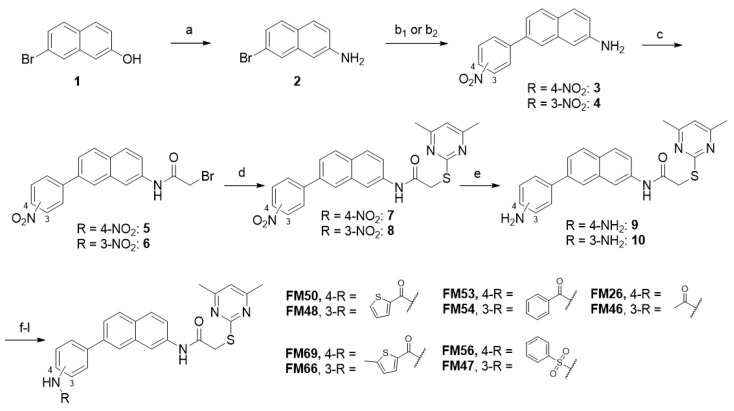
Conditions: (a) NaHSO_3_, NH_4_OH, 180 °C, 72 h, 88%; (b_1_) 4-nitrophenylboronic acid, K_2_CO_3_, Pd(PPh_3_)_4_, DMF, 105 °C, 23 h, 42% for **3**; (b_2_) 3-nitrophenylboronic acid, K_2_CO_3_, Pd(PPh_3_)_4_, DMF, 105 °C, 23 h, 38% for **4**; (c) bromoacetic acid, DCM, 0 °C, 2 h, 89% for **5** and 90% for **6**; (d) 4,6-dimethylpyrimidine-2-thiol, DMF, *t*-BuOK, rt, 4 h, 83% for **7** and 87% for **8**; (e) Fe, AcOH, 50 °C, 3 h, 80% for **9** and 75% for **10**; (f) 2-thiophenecarbonyl chloride, NEt_3_, DCM, rt, 2 h, 53% for **FM50** and 31% for **FM48**; (g) 5-methylthiophene-2-carbonyl chloride, NEt_3_, DCM, 40 °C, 3 h, 74% for **FM69**; (h) 5-methylthiophene-2-carbonyl chloride, NEt_3_, DCM, rt, 5 h, 83% for **FM66**; (i) benzoyl chloride, NEt_3_, DCM, rt, 2 h, 78% for **FM53** and 87% for **FM54**; (j) benzenesulfonyl chloride, NEt_3_, DCM, 40 °C, 7 h, 40% for **FM56**; (k) benzenesulfonyl chloride, NEt_3_, DCM, rt, 4 h, 47% for **FM47**; (l) acetic anhydride, NEt_3_, DCM, rt, 2 h, 72% for **FM26** and 91% for **FM46**.

**Scheme 2:**
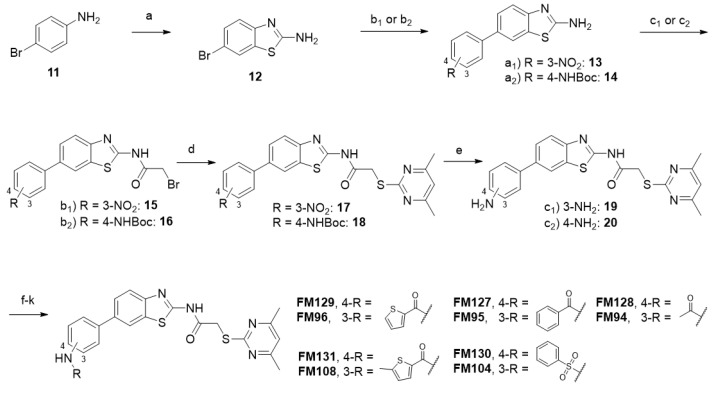
Conditions: (a) KSCN, Br_2_, AcOH, 0 °C, 23 h, 35%; (b_1_) (3-nitrophenyl)boronic acid, Cs_2_CO_3_, Pd(PPh_3_)_4_, dioxane/water, 80 °C, 21 h, 61% for **13**; (b_2_) 4-(*N*-Boc-amino)phenylboronic acid, Cs_2_CO_3_, Pd(PPh_3_)_4_, dioxane/water, 80 °C, 21 h, 28% for **14**; (c_1_) bromoacetic acid, DCM, NEt_3_, DCM/DMF (6:1), 40 °C, 3 h, 26% for **15**; (c_2_) bromoacetic acid NEt_3_, EtOAc, 40 °C, 3 h, 45% for **16**; (d) 4,6-dimethylpyrimidine-2-thiol, DMF, *t*-BuOK, rt, 3 h, 67% for **17** and 78% for **18**; (e) Fe, MeOH/water, NH_4_Cl, 65 °C, 3 h, 74% for **19**; TFA, chloroform, rt, 24 h, 83% for **20**. (f) 2-thiophenecarbonyl chloride, NEt_3_, DCM, rt, 1 h, 77% for **FM129** and 81% for **FM96**; (g) 5-methylthiophene-2-carbonyl chloride, NEt_3_, DCM, rt, 2 h, 45% for **FM131** and 46% for **FM108**; (h) benzoyl chloride, NEt_3_, DCM, rt, 3 h, 46% for **FM127** and 66% for **FM95**; (i) benzenesulfonyl chloride, NEt_3_, DCM, 40 °C, 8 h, 59% for **FM130**, (j) benzenesulfonyl chloride, NEt_3_, DCM, rt, 8 h, 15% for **FM104**; (k) acetic anhydride, NEt_3_, DCM, rt, 3 h, 60% for **FM128** and 96% for **FM94**.

The general synthesis approach is based on previous work on lead compound **28e** by Yang et al. [16]; however, the rigidization efforts required adapted synthesis strategies with special regard to the linking of the selected rigid elements. Due to individual solubility requirements, recurring reaction types were adapted to the respective properties by adjusting solvent, reaction time, and temperature. The starting point for the naphthalene-based rigid candidates was literature-known 7-bromo-naphtalene-2-amine (**2**), prepared by means of a Bucherer amination protocol [25] of 7-bromo-2-naphthol (**1**) (Scheme 1). The benzothiazole-based rigid candidates were built up from 2-amino-6-bromobenzothiazole, which was prepared following a literature-known protocol [26] using 4-bromoaniline, potassium thiocyanate, and bromine (Scheme 2). By using appropriate *meta*- and *para*- substituted nitrophenyl boronic acids, intermediates **3**, **4**, and **13** were prepared via Suzuki cross-coupling; in the case of biaryl **14**, Boc-protected 4-aminophenylboronic acid was chosen instead of the nitro compound due to the poor solubility properties of the latter. The corresponding 2-bromoacetamides **5** and **6** as well as **15** and **16** were prepared by *N*-acylation, followed by the nucleophilic displacement of the bromo substituents by 4,6-dimethylpyrimidine-2-thiol to provide the structural part known as the “selectivity pocket binder” (compounds **7** and **8** as well as **17** and **18**). Iron-mediated reduction in the nitro group of compounds **7**, **8**, and **17** and the TFA-based cleavage of the Boc-protecting group of **18** yielded amines **9** and **10** as well as **19** and **20**. The *N*-acylation of the respective primary amines using appropriate acid chlorides or acetic anhydride as well as sulfonylation with benzenesulfonyl chloride yielded the envisaged 10 naphthalene-based and 10 benzothiazole-based rigidized target compounds.

### 2.3. Biological Evaluation of Inhibitory Activity and Subtype Selectivity

All 20 rigid target candidates, including the lead compounds **28e** (synthesized according to the literature [16] and **SirReal2** (commercially available from Sigma Aldrich), were evaluated for inhibitory potency (IC_50_) on Sirt2, as well as subtype selectivity on closely related isoforms Sirt1, Sirt3, and Sirt5, using a fluorescence-based assay performed by Reaction Biology Corporation (Malvern, PA, USA) (Table 1). **SirReal2** (published IC_50_ = 0.44 µM) was determined in this test system with an IC_50_ value of 0.24 µM towards Sirt2, lead compound **28e** (published IC_50_ = 0.041 µM) with 0.087 µM against Sirt2. The subtype selectivity of the synthesized target substances with IC_50_ values below 100 µM at Sirt2 was evaluated by determining the inhibitory activity in percent at subtypes Sirt1, Sirt3, and Sirt5 at a fixed inhibitor concentration of 50 µM. None of the rigid test substances examined showed significant inhibitory activity on the corresponding subtypes as all individual inhibition data on respective subtypes are suggesting IC_50_ values greater than 50 µM. Therefore, considering the measured inhibitory activity against Sirt2, the rigid target substances investigated are highly selective towards Sirt2. Consequently, our results are in a comparable range as published data if deviating values due to different test conditions are taken into account and reflect the measurable advantage of **28e** as a further development of **SirReal2**. Of the 20 rigidized test compounds examined, five substances (**FM50**, **FM69**, **FM131**, **FM53**, and **FM127**) showed interesting submicromolar IC_50_ values, the remaining 15 compounds gave IC_50_ values above 1 µM, whereby three of them (**FM96**, **FM66**, and **FM95**) with IC_50_ values above 100 µM showed neglectable inhibitory activity. The rigidization strategy revealed interesting conclusions about underlying structure–activity relationships. In principle, the attachment of the (sulfon)amide residues at the *meta* position of the phenyl ring (addressing the substrate-binding pocket) is to be regarded as disadvantageous in the majority of cases, which becomes particularly clear when comparing the *para*-substituted **FM69** (IC_50_ = 0.15 µM, Sirt2) and the *meta*-substituted **FM66** (IC_50_ > 100 µM). Except for the *para*-substituted benzenesulfonamide **FM130** (IC_50_ = 12 µM, Sirt2) and the *meta*-substituted analogue **FM47** (IC_50_ = 1.3 µM, Sirt2), this trend is maintained throughout the other rigid test compounds. A comparison of the selected rigid elements naphthalene and benzothiazole shows a consistent superiority of naphthalene-based rigidization over benzothiazole-based rigidization. Although benzothiazole-based test compounds such as **FM131** (IC_50_ = 0.61 µM) can achieve significant inhibitory potency, they are still consistently behind their corresponding naphthalene-based analogues, e.g., for **FM131** analogue **FM69** (IC_50_ = 0.15 µM).

**Figure 4:**
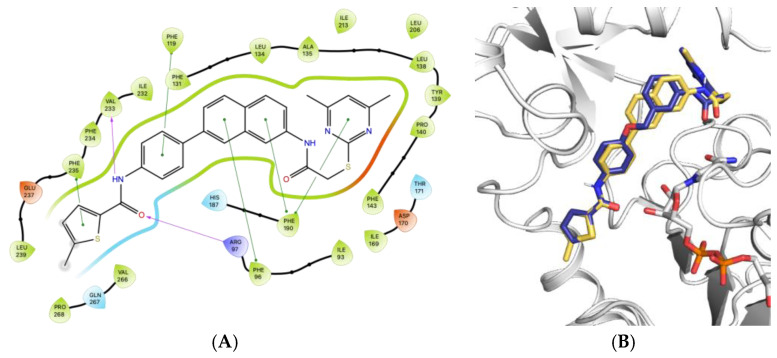
(**A**) Illustration of the docking pose of **FM69** and corresponding protein–ligand interactions as a 2D detailed view of the Sirt2-binding pocket. (**B**) Docking pose of lead compound **28e** (blue, based on PDB ID: 5YQO prediction), superimposed with naphthalene-based rigid inhibitor **FM69** (yellow). The position of cofactor NAD^+^ is visualized by overlay with PDB ID: 4RMG.

Not only the respective rigidizing element (naphthalene or benzothiazole) and the corresponding substitution pattern (*meta*/*para*) of the amide-based ligand for the substrate-binding pocket has a significant influence on the inhibitory potency of the rigidized target substances but also the underlying specific amide residue of this moiety: the thiophene-2-carboxamide (**FM50**: IC_50_ = 0.37 µM), 5-methylthiophene-2-carboxamides (**FM69**: IC_50_ = 0.15 µM; **FM131**: IC_50_ = 0.61 µM), and benzamides (**FM53**: IC_50_ = 0.22 µM; **FM127**: IC_50_ = 0.42 µM) proved to be particularly favorable and represent the most potent rigid test candidates in this screening. Therefore, **FM50**, the analogue of lead compound **28e**, which was directly rigidized with a naphthalene element in the initial docking experiments (Figure 2), is among the most potent test candidates. **FM129**, the variation of lead compound **28e** rigidized with a benzothiazole element, was determined with an IC_50_ value of 1.2 µM on Sirt2 and thus cannot quite meet the expectations from the docking study, which is further transferable to the other benzothiazole-based rigidization approaches. The most potent rigid compound is **FM69** with an IC_50_ value of 0.15 µM at Sirt2 and is characterized by a 5-methylthiophene-2-carboxeamide-based, *para*-located ligand for the substrate-binding pocket with a naphthalene rigidification element. Although rigidization did not result in compounds with superior inhibitory activity compared to lead compound **28e** (IC_50_ = 0.087 µM), our comprehensive SAR analysis provided valuable insights and correlations that can serve as a foundation for the design of future Sirt2 inhibitors. **FM69**, with an IC_50_ of 0.15 µM, emerged as the best rigidized analogue of **28e** and was further investigated in a docking simulation regarding its spatial orientation (see Figure 4).

According to docking simulations, **FM69** exhibits a very similar spatial binding mode to the lead compound **28e** and is consistent with the initial calculations made for **FM50**. The protein–ligand interactions of **FM69** are characterized by various π–π interactions, including those with Phe96, Phe119, Phe190, and Phe235. Additionally, the amide structure within the substrate-binding pocket mediates polar hydrogen bonding with Arg97 and Val233. As expected, the 2-((4,6-dimethylpyrimidin-2-yl)thio)acetamide moiety occupies the induced hydrophobic binding pocket (referred to as the selectivity pocket) through interactions with neighboring hydrophobic residues, including Ile213, Leu206, Leu136, Tyr139, Pro140, and Phe143. Furthermore, the docking pose reveals the characteristic and essential intramolecular hydrogen bond between the amide-NH and the pyrimidine nitrogen (selectivity pocket binder structure motif), as described by Schiedel et al. [15]. The methyl group attached to the thiophene ring in **FM69** increases the lipophilic surface area, facilitating hydrophobic interactions with the Leu239 and Pro268 sidechains, which improves its inhibitory potency compared to the initial rigid concept compound **FM50**, as reflected by a better IC_50_ value. Structural variations in the amide substitutions of residues binding to the substrate-binding pocket have shown a notable impact on inhibitory activity against Sirt2. These findings suggest that this approach offers a promising strategy for the further optimization of naphthalene-based rigid analogues of **28e**.

## 3. Conclusions

The rigidization approach of lead structures **SirReal2** (IC_50_ = 0.24 µM) and **28e** (IC_50_ = 0.087 µM) as outstanding published Sirt2 inhibitors yielded several new highly selective Sirt2 inhibitors with submicromolar IC_50_ values, which, although not exceeding the inhibitory activity of lead structure **28e**, displays a valuable contribution to the understanding of the structure–activity relationships of SirReal-type inhibitors. Of the 20 rigid target compounds synthesized, five substances showed sub-micromolar IC_50_ values, of which **FM69** (IC_50_ = 0.15 µM) exhibited the strongest inhibitory effect, representing a respectable inhibitory potency and consequently ranking in the potency range of the strongest known selective Sirt2 inhibitors, surpassing **SirReal2** (IC_50_ = 0.24 µM).

The rigidization strategy is a well-established medicinal chemistry approach for structure optimization, presenting both opportunities and challenges. The entropic binding advantage is contrasted by potential steric hindrances and divergent interactions, possibly explaining the partly very different inhibitory activities of the rigidized target substances. The binding pocket of Sirt2 possesses high structural requirements, which is made clear by the distinct *para*-substitution pattern preference for the *N*-acyl residue and the superiority of the naphthalene-based rigid element in comparison to the benzothiazole-based analogues. By applying the rigidization principle to lead compounds **SirReal2** and **28e**, an ample compound library was built up, which, through corresponding SAR analysis, provides a platform for the further development and future design of SirReal-type Sirt2 inhibitors, especially through the further variation of the amide-based ligands for the substrate-binding pocket.

## 4. Experimental

### 4.1. Chemical Synthesis

Solvents and reagents were purchased commercially and used without further purification. Reaction control was performed via thin-layer chromatography (TLC) using 0.2 mm silica-gel-coated POLYGRAM^®^ SIL G/UV254 polyester sheets (Macherey-Nagel, Düren, Germany) by UV-light visualization (254 nm). Flash column chromatography was performed using SiO_2_ 60 (0.040–0.063 mm, 230–400 mesh ASTM) by Merk (Darmstadt, Germany). Melting points were determined using a Büchi B-540 melting point meter (Fawil, Switzerland). The Perkin Elmer FT-IR BXII/1000 spectrometer (Waltham, MA, USA) in combination with DuraSamp IR II Diamond ATR sensor (Smiths Detection, London, UK) was used to perform infrared spectroscopy (IR). The IR spectra was recorded from wavenumbers 4000 to 650 cm^−1^, and crucial absorption bands are given as wavenumber ($\overset{˷}{\nu }$) in cm^−1^. High-resolution mass spectroscopy (HR-MS) was performed using a Jeol Mstation 700 (Akishima, Japan) or JMS GCmate II Jeol (Akishima, Japan) instrument for electron impact ionization (EI). Electrospray ionization (ESI) HR-MS was performed using a Thermo Finnigan LTQ (Thermo Fisher Scientific, Waltham, MA, USA) instrument. NMR spectra were recorded with Avance III HD 400 MHz Bruker BioSpin (Bruker Corporation, Billerica, MA, USA) for ^1^H NMR: 400 MHz and for ^13^C NMR: 101 MHz or Avance III HD 500 MHz Bruker BioSpin for ^1^H NMR: 500 MHz and for ^13^C NMR: 126 MHz. MestreNova 14.3.0 (Mestrelab Research S.L., Santiago de Compostela, Spain) was used as analysis program, and the obtained chemical shifts δ (ppm, parts per million) were referenced to the deuterated solvent peak (DMSO-*d*_6_: δH = 2.50 ppm, δC = 39.52 ppm; CD_2_Cl_2_: δH = 5.32 ppm, δC = 53.84 ppm; CDCl_3_: δH = 7.26 ppm, δC = 77.16 ppm). Coupling constants J are assigned in Hertz (Hz) and multiplicities are reported as follows: s (singlett), d (doublet), t (triplet), and q (quartet); derivatives of that or m (multiplet). The compound numbering follows IUPAC conventions, but, for clarity purposes, the assignment of the hierarchy levels of NMR signals (indicated by apostrophes) was carried out based on the order of synthesis. HPLC purity was determined at 210 nm and 254 nm with 1100/1200 diode array detector using HP Agilent 1100 HPLC device by Agilent (Santa Clara, CA, USA) with Zorbax Eclipse Plus^®^ C18 5 µm (4.6 × 150 mm) column by Agilent and acetonitrile/water as eluent and Chromeleon 7.2.9 Software by Thermo Fisher Scientific (Waltham, MA, USA). ^1^H and ^13^C NMR spectra of synthesized compounds and HPLC chromatograms of final test substances are provided in Supplementary Materials.

7-(4-Nitrophenyl)naphthalen-2-amine (**3**). At room temperature, 7-bromonaphthalene-2-amine (**2**, 3.40 g, 15.3 mmol, 1.00 eq), 4-nitrophenyl boronic acid (3.06 g, 18.4 mmol, 1.20 eq), K_2_CO_3_ (6.32 g, 38.3 mmol, 2.50 eq), and Pd(PPh_3_)_4_ (0.884 g, 0.765 mmol, 0.0500 eq) were weighed out into a flask, which was put under nitrogen afterwards. Degassed anhydrous DMF (15 mL) was added via syringe, and the reaction mixture was stirred for 23 h at 105 °C and then for another 2 h at room temperature under nitrogen atmosphere. Subsequently, the mixture was diluted with water (100 mL) and brine (200 mL) and extracted with EtOAc (4 × 100 mL). The combined organic layers were dried over sodium sulfate. After evaporating the solvent in vacuo, the crude product was purified via flash column chromatography (hexanes/EtOAc 7:3 → 3:7) to yield the title compound as a red-orange solid (1.70 g, 6.44 mmol, 42%). m.p.: 233 °C, R*f*: 0.16 (hexanes/EtOAc 7:3). ^1^H NMR (400 MHz, DMSO-*d*_6_) δ [ppm] = 8.34–8.29 (m, 2H, 3′-H, 5′-H), 8.08–8.02 (m, 2H, 2′-H, 6′-H), 7.95 (d, *J* = 1.9 Hz, 1H, 8-H), 7.77 (d, *J* = 8.5 Hz, 1H, 5-H), 7.65 (d, *J* = 8.7 Hz, 1H, 4-H), 7.48 (dd, *J* = 8.4, 1.9 Hz, 1H, 6-H), 7.00 (dd, *J* = 8.7, 2.2 Hz, 1H, 3-H), 6.93 (d, *J* = 2.2 Hz, 1H, 1-H), 5.52 (s, 2H, NH_2_). ^13^C NMR (101 MHz, DMSO-*d_6_*) δ [ppm] = 147.4 (C-2), 147.2 (C-1′), 146.4 (C-4′), 135.2 (C-7), 135.0 (C-8a), 128.6 (C-5), 128.3 (C-4), 127.9 (C-2′, C-6′), 126.2 (C-4a), 124.04 (C-3′, C-5′), 123.97 (C-8), 119.6 (C-6), 119.5 (C-3), 106.3 (C-1). IR (ATR) ν῀ [cm^−1^] = 3442, 3355, 1630, 1588, 1501, 1462, 1392, 1340, 1279, 1248, 1108, 895, 855, 835, 750, 696. HRMS (EI): *m/z* = [M]^•^^+^ calculated for C_16_H_12_N_2_O_2_^•^^+^: 264.0893; found: 264.0899.

7-(3-Nitrophenyl)naphthalen-2-amine (**4**). At room temperature, 7-bromonaphthalene-2-amine (**2**, 3.40 g, 15.8 mmol, 1.00 eq), 3-nitrophenyl boronic acid (3.16 g, 18.9 mmol, 1.20 eq), K_2_CO_3_ (6.32 g, 38.3 mmol, 2.50 eq), and Pd(PPh_3_)_4_ (0.991 g, 0.788 mmol, 0.0500 eq) were weighed out into a flask, which was put under nitrogen afterwards. Degassed anhydrous DMF (15 mL) was added via syringe, and the reaction mixture was stirred for 23 h at 105 °C and then for another 2 h at room temperature under nitrogen atmosphere. Subsequently, the mixture was diluted with water (100 mL) and brine (200 mL) and extracted with EtOAc (4 × 100 mL). The combined organic layers were dried over sodium sulfate. After evaporating the solvent in vacuo, the crude product was purified via flash column chromatography (hexanes/EtOAc 7:3 → 3:7) to yield the title compound as a pink-red solid (1.61 g, 6.07 mmol, 38%). m.p.: 160 °C, *Rf*_:_ 0.23 (hexanes/EtOAc 7:3). ^1^H NMR (400 MHz, DMSO-*d_6_*) δ [ppm] = 8.52 (t, *J* = 2.1 Hz, 1H, 2′-H), 8.26–8.18 (m, 2H, 4′-H, 6′-H), 7.94 (d, *J* = 1.9 Hz, 1H, 8-H), 7.82–7.73 (m, 2H, 5-H, 5′-H), 7.65 (d, *J* = 8.7 Hz, 1H, 4-H), 7.48 (dd, *J* = 8.4, 1.9 Hz, 1H, 6-H), 6.98 (dd, *J* = 8.7, 2.2 Hz, 1H, 3-H), 6.94 (d, *J* = 2.2 Hz, 1H, 1-H), 5.50 (s, 2H, NH_2_). ^1^^3^C NMR (101 MHz, DMSO-*d_6_*) δ [ppm] = 148.5 (C-3′), 147.3 (C-2), 142.3 (C-1′), 135.3 (C-7), 135.1 (C-8a), 133.4 (C-6′), 130.4 (C-5′), 128.6 (C-5), 128.3 (C-4), 126.0 (C-4a), 123.4 (C-8), 121.9 (C-4′), 121.1 (C-2′), 119.6 (C-6), 119.2 (C-3), 106.2 (C-1). IR (ATR) ν῀ [cm^−1^] = 3450, 3372, 1631, 1526, 1513, 1485, 1460, 1388, 1340, 1308, 1269, 1224, 1190, 1138, 1101, 1082, 890, 870, 838, 814, 776, 738, 716, 682, 659. HRMS (EI): *m/z* = [M]^•^^+^ calculated for C_16_H_12_N_2_O_2_^•^^+^: 264.0893; found: 264.0897.

2-Bromo-*N*-(7-(4-nitrophenyl)naphthalen-2-yl)acetamide (**5**). At 0 °C, bromoacetyl bromide (1.38 mL, 15.9 mmol, 8.00 eq) was added dropwise to a stirred suspension of amine **3** (525 mg, 1.99 mmol, 1.00 eq) in anhydrous DCM (100 mL). After 2 h, water (50 mL) and brine (150 mL) were added, and the reaction mixture was extracted with DCM (4 × 100 mL). The combined organic layers were dried over sodium sulfate. After evaporating the solvent in vacuo, the crude product was purified via flash column chromatography (hexanes/EtOAc 7:3 → 1:9) to yield the title compound as a yellow-orange solid (684 mg, 1.78 mmol, 89%). m.p.: 189 °C, R*f*: 0.20 (hexanes/EtOAc 7:3). ^1^H NMR (400 MHz, CD_2_Cl_2_) δ [ppm] = 8.35 (d, *J* = 1.9 Hz, 1H, 1-H), 8.35–8.31 (m, 2H, 3′-H, 5′-H), 8.26 (bs, 1H, NHCO), 8.12 (d, *J* = 2.3 Hz, 1H, 8-H), 7.95 (d, *J* = 8.5 Hz, 1H, 4-H), 7.93–7.87 (m, 3H, 5-H, 2′-H, 6′-H), 7.74 (dd, *J* = 8.5, 1.9 Hz, 1H, 6-H), 7.55 (dd, *J* = 8.8, 2.2 Hz, 1H, 3-H), 4.09 (s, 2H, CH_2_). ^1^^3^C NMR (101 MHz, CD_2_Cl_2_) δ [ppm] = 164.3 (NHCO), 147.83 (C-1′), 147.80 (C-4′), 137.4 (C-2), 136.1 (C-7), 134.4 (C-8a), 131.3 (C-4a), 129.3 (C-4), 129.2 (C-5), 128.6 (C-2′, C-6′), 127.2 (C-8), 125.0 (C-6), 124.7 (C-3′, C-5′), 121.2 (C-3), 117.7 (C-1), 30.2 (CH_2_). IR (ATR) ν῀ [cm^−1^] = 3302, 2926, 1670, 1590, 1561, 1548, 1506, 142, 1397, 1334, 1287, 1214, 1108, 903, 850, 833, 750, 716, 690, 657. HRMS (ESI): *m/z* = [M-H]^−^ calculated for C_18_H_12_^79^BrN_2_O_3_^−^: 383.0037; found: 383.0041.

2-Bromo-*N*-(7-(3-nitrophenyl)naphthalen-2-yl)acetamide (**6**). At 0 °C, bromoacetyl bromide (736 µL, 8.45 mmol, 3.00 eq) was added dropwise to a stirred suspension of amine **4** (745 mg, 2.82 mmol, 1.00 eq) in anhydrous DCM (100 mL). After 2 h, water (50 mL) and brine (150 mL) were added, and the reaction mixture was extracted with DCM (4 × 100 mL). The combined organic layers were dried over sodium sulfate. After evaporating the solvent in vacuo, the crude product was purified via flash column chromatography (hexanes/EtOAc 7:3 → 1:9) to yield the title compound as a dark-yellow solid (981 mg, 2.55 mmol, 90%). m.p.: 200 °C, R*f*: 0.20 (hexanes/EtOAc 7:3). ^1^H NMR (500 MHz, CD_2_Cl_2_) δ [ppm] = 8.58 (t, *J* = 2.0 Hz, 1H, 2′-H), 8.34 (d, *J* = 2.2 Hz, 1H, 1-H), 8.27 (s, 1H, NHCO), 8.23 (ddd, *J* = 8.2, 2.3, 1.0 Hz, 1H, 4′-H), 8.11 (dd, *J* = 1.8, 0.8 Hz, 1H, 8-H), 8.08 (ddd, *J* = 7.7, 1.9, 1.0 Hz, 1H, 6′-H), 7.96 (d, *J* = 8.5 Hz, 1H, 5-H), 7.90 (d, *J* = 8.7 Hz, 1H, 4-H), 7.74 (dd, *J* = 8.5, 1.9 Hz, 1H, 6-H), 7.68 (t, *J* = 8.0 Hz, 1H, 5′-H), 7.55 (dd, *J* = 8.8, 2.2 Hz, 1H, 3-H), 4.09 (s, 2H, CH_2_). ^1^^3^C NMR (126 MHz, CD_2_Cl_2_) δ [ppm] = 164.3 (NHCO), 149.4 (C-3′), 143.1 (C-1′), 137.4 (C-7), 136.1 (C-8a), 134.4 (C-2), 133.9 (C-6′), 131.1 (C-4a), 130.5 (C-5′), 129.3 (C-4), 129.2 (C-5), 126.7 (C-8), 125.0 (C-6), 122.7 (C-4′), 122.6 (C-2′), 121.0 (C-3), 117.6 (C-1), 30.2 (CH_2_). IR (ATR) ν῀ [cm^−1^] = 3266, 3078, 1687, 1662, 1632, 1611, 1528, 1516, 1463, 1398, 1334, 1271, 1243, 1227, 1187, 1172, 1098, 984, 953, 885, 866, 842, 804, 741, 830, 680. HRMS (ESI): *m/z* = [M-H]^−^ calculated for C_18_H_12_^7^^9^BrN_2_O_3_^−^: 383.0037; found: 383.0035.

2-((4,6-Dimethylpyrimidin-2-yl)thio)-*N*-(7-(4-nitrophenyl)naphthalen-2-yl)acetamide (**7**). At room temperature, potassium *tert*-butoxide (346 mg, 3.08 mmol, 2.00 eq) was added to a stirred solution of 2-bromoacetamide **5** (594 mg, 1.54 mmol, 1.00 eq) and 4,6-dimethylpyrimidine-2-thiol (260 mg, 1.85 mmol, 1.20 eq) in anhydrous DMF (10 mL). After 3 h, water (150 mL) and brine (100 mL) were added, and the reaction mixture was extracted with EtOAc (4 × 100 mL). The combined organic layers were dried over sodium sulfate. After evaporating the solvent in vacuo, the crude product was purified via flash column chromatography (hexanes/EtOAc 6:4) to yield the title compound as a yellow-orange solid (684 mg, 1.78 mmol, 89%). m.p.: 185 °C, R*f*: 0.18 (hexanes/EtOAc 6:4). ^1^H NMR (500 MHz, DMSO-*d_6_*) δ [ppm] = 10.53 (s, 1H, NHCO), 8.42 (d, *J* = 2.2 Hz, 1H, 1-H), 8.37–8.30 (m, 2H, 3′-H, 5′-H), 8.29 (d, *J* = 1.9 Hz, 1H, 8-H), 8.15–8.10 (m, 2H, 2′-H, 6′-H), 7.99 (d, *J* = 8.5 Hz, 1H, 5-H), 7.94 (d, *J* = 8.8 Hz, 1H, 4-H), 7.82 (dd, *J* = 8.5, 1.9 Hz, 1H, 6-H), 7.67 (dd, *J* = 8.8, 2.1 Hz, 1H, 3-H), 6.96 (s, 1H, 5″-H), 4.12 (s, 2H, CH_2_), 2.33 (s, 6H, 4″-CH_3_, 6″-CH_3_). ^1^^3^C NMR (126 MHz, DMSO-*d_6_*) δ [ppm] = 169.3 (C-2″), 166.98 (C-4″, C-6″), 166.94 (NHCO), 146.7 (C-4′), 146.5 (C-1′), 137.3 (C-2), 135.6 (C-7), 133.6 (C-8a), 129.6 (C-4a), 128.6 (C-5), 128.3 (C-4), 128.1 (C-2′, C-6′), 126.2 (C-8), 124.1 (C-3′, C-5′), 123.5 (C-6), 120.9 (C-3), 116.1 (C-5″), 115.8 (C-1), 35.6 (CH_2_), 23.3 (4″-CH_3_, 6″-CH_3_). IR (ATR) ν῀ [cm^−1^] = 3240, 3078, 1667, 1584, 1560, 1512, 1428, 1393, 1344, 1315, 1268, 1221, 1175, 1152, 1107, 904, 884, 847, 752, 696. HRMS (ESI): *m/z* = [M+H]^+^ calculated for C_24_H_21_N_4_O_3_S^+^: 445.1329; found: 445.1329.

2-((4,6-Dimethylpyrimidin-2-yl)thio)-*N*-(7-(3-nitrophenyl)naphthalen-2-yl)acetamide (**8**). At room temperature, potassium *tert*-butoxide (522 mg, 4.65 mmol, 2.00 eq) was added to a stirred solution of 2-bromoacetamide **6** (896 mg, 2.32 mmol, 1.00 eq) and 4,6-dimethylpyrimidine-2-thiol (391 mg, 2.79 mmol, 1.20 eq) in anhydrous DMF (10 mL). After 3 h, water (150 mL) and brine (100 mL) were added, and the reaction mixture was extracted with EtOAc (4 × 100 mL). The combined organic layers were dried over sodium sulfate. After evaporating the solvent in vacuo, the crude product was purified via flash column chromatography (hexanes/EtOAc 6:4) to yield the title compound as a white-pink solid (895 mg, 2.01 mmol, 87%). m.p.: 94–98 °C, R*f*: 0.17 (hexanes/EtOAc 6:4). ^1^H NMR (500 MHz, DMSO-*d_6_*) δ [ppm] = 10.51 (s, 1H, NHCO), 8.60 (t, *J* = 2.1 Hz, 1H, 2′-H), 8.42 (d, *J* = 2.1 Hz, 1H, 1-H), 8.34–8.28 (m, 2H, 8-H, 6′-H), 8.25 (ddd, *J* = 8.0, 2.3, 0.9 Hz, 1H, 4′-H), 7.99 (d, *J* = 8.5 Hz, 1H, 5-H), 7.93 (d, *J* = 8.8 Hz, 1H, 4-H), 7.86–7.77 (m, 2H, 6-H, 5′-H), 7.65 (dd, *J* = 8.8, 2.1 Hz, 1H, 3-H), 6.97 (s, 1H, 5″-H), 4.12 (s, 2H, CH_2_), 2.33 (s, 6H, 4″-CH_3_, 6″-CH_3_). ^1^^3^C NMR (126 MHz, DMSO-*d_6_*) δ [ppm] = 169.3 (C-2″), 167.0 (C-4″, C-6″), 166.9 (NHCO), 148.5 (C-3′), 141.7 (C-1′), 137.3 (C-2), 135.6 (C-7), 133.7 (C-8a), 133.6 (C-6′), 130.5 (C-5′), 129.4 (C-4a), 128.5 (C-5), 128.3 (C-4), 125.7 (C-8), 123.5 (C-6), 122.2 (C-4′), 121.4 (C-2′), 120.6 (C-3), 116.1 (C-5″), 115.7 (C-1), 35.6 (CH_2_), 23.3 (4″-CH_3_, 6″-CH_3_). IR (ATR) ν῀ [cm^−1^] = 3289, 3066, 1688, 1582, 1527, 1512, 1433, 1341, 1266, 1240, 893, 841, 806, 737, 685. HRMS (ESI): *m/z* = [M+H]^+^ calculated for C_24_H_21_N_4_O_3_S^+^: 445.1329; found: 445.1324.

*N*-(7-(4-Aminophenyl)naphthalen-2-yl)-2-((4,6-dimethylpyrimidin-2-yl)thio)acetamide (**9**). Under nitrogen atmosphere, nitro compound **7** (0.533 g, 1.20 mmol, 1.00 eq) was suspended in acetic acid (5 mL), and iron powder (2.34 g, 42.0 mmol, 35.0 eq) was added. After the reaction mixture was stirred at 50 °C for 3 h, it was filtered and then diluted with water (50 mL) and brine (100 mL). The mixture was extracted with EtOAc (4 × 50 mL), and the combined organic layers were dried over sodium sulfate. After evaporating the solvent in vacuo, the crude product was purified via flash column chromatography (hexanes/EtOAc 1:1) to yield the title compound as a yellow solid (397 mg, 0.957 mmol, 80%). m.p.: 202 °C, R*f*: 0.10 (hexanes/EtOAc 1:1). ^1^H NMR (500 MHz, DMSO-*d_6_*) δ [ppm] = 10.42 (s, 1H, NHCO), 8.25 (d, *J* = 2.0 Hz, 1H, 1-H), 7.91 (d, *J* = 1.9 Hz, 1H, 8-H), 7.84–7.79 (m, 2H, 4-H, 5-H), 7.64 (dd, *J* = 8.6, 1.8 Hz, 1H, 6-H), 7.53 (dd, *J* = 8.7, 2.1 Hz, 1H, 3-H), 7.52–7.49 (m, 2H, 2′-H, 6′-H), 6.97 (s, 1H, 5″-H), 6.70–6.64 (m, 2H, 3′-H, 5′-H), 5.27 (s, 2H, NH_2_), 4.10 (s, 2H, CH_2_), 2.34 (s, 6H, 4″-CH_3_, 6″-CH_3_). ^1^^3^C NMR (126 MHz, DMSO-*d_6_*) δ [ppm] = 169.4 (C-2″), 167.0 (C-4″, C-6″), 166.8 (NHCO), 148.6 (C-4′), 138.5 (C-7), 136.8 (C-2), 134.0 (C-8a), 128.2 (C-4a), 128.0 (C-4), 127.8 (C-5), 127.5 (C-2′, C-6′), 127.0 (C-1′), 123.3 (C-6), 122.4 (C-8), 119.1 (C-3), 116.1 (C-5″), 115.3 (C-1), 114.3 (C-3′, C-5′), 35.6 (CH_2_), 23.3 (4″-CH_3_, 6″-CH_3_). IR (ATR) ν῀ [cm^−1^] = 3439, 3355, 3248, 2968, 2911, 1660, 1618, 1606, 1577, 1532, 1506, 1338, 1264, 1248, 1228, 1190, 1168, 984, 952, 896, 851, 828, 802, 728, 706. HRMS (ESI): *m/z* = [M-H]^−^ calculated for C_24_H_21_N_4_OS^−^: 413.1442; found: 413.1442.

*N*-(7-(3-Aminophenyl)naphthalen-2-yl)-2-((4,6-dimethylpyrimidin-2-yl)thio)acetamide (**10**). Under nitrogen atmosphere, nitro compound **8** (0.684 g, 1.54 mmol, 1.00 eq) was suspended in acetic acid (5 mL), and iron powder (3.01 g, 53.9 mmol, 35.0 eq) was added. After the reaction mixture was stirred at 50 °C for 3 h, it was filtered and then diluted with water (50 mL) and brine (100 mL). The mixture was extracted with EtOAc (4 × 50 mL), and the combined organic layers were dried over sodium sulfate. After evaporating the solvent in vacuo, the crude product was purified via flash column chromatography (hexanes/EtOAc 1:1) to yield the title compound as a yellow solid (480 mg, 1.16 mmol, 75%). m.p.: 167 °C, R*f*: 0.11 (hexanes/EtOAc 1:1). ^1^H NMR (500 MHz, DMSO-*d_6_*) δ [ppm] = 10.46 (s, 1H, NHCO), 8.30 (d, *J* = 2.1 Hz, 1H, 1-H), 7.95 (d, *J* = 1.8 Hz, 1H, 8-H), 7.92–7.85 (m, 2H, 4-H, 5-H), 7.66–7.58 (m, 2H, 3-H, 6-H), 7.14 (t, *J* = 7.8 Hz, 1H, 5′-H), 6.99 (t, *J* = 2.0 Hz, 1H, 2′-H), 6.98 (s, 1H, 5″-H), 6.92 (ddd, *J* = 7.6, 1.8, 1.0 Hz, 1H, 6′-H), 6.60 (ddd, *J* = 7.9, 2.2, 0.9 Hz, 1H, 4′-H), 5.17 (s, 2H, NH_2_), 4.11 (s, 2H, CH_2_), 2.34 (s, 6H, 4″-CH_3_, 6″-CH_3_). ^1^^3^C NMR (126 MHz, DMSO-*d_6_*) δ [ppm] = 169.3 (C-2″), 170.0 (C-4″, C-6″), 166.8 (NHCO), 149.2 (C-3′), 140.7 (C-1′), 138.9 (C-7), 137.0 (C-2), 133.7 (C-8a), 129.5 (C-5′), 128.9 (C-4a), 128.2 (C-5), 128.0 (C-4), 124.2 (C-8), 123.9 (C-6), 119.8 (C-3), 116.1 (C-5″), 115.4 (C-1), 114.6 (C-6′), 113.3 (C-4′), 112.4 (C-2′), 35.6 (CH_2_), 23.3 (4″-CH_3_, 6″-CH_3_). IR (ATR) ν῀ [cm^−1^] = 3430, 3224, 3097, 1656, 1631, 1611, 1584, 1546, 1516, 1493, 1404, 1341, 1322, 1272, 1236, 922, 940, 904, 886, 858, 840, 774, 749, 691. HRMS (ESI): *m/z* = [M-H]^−^ calculated for C_24_H_21_N_4_OS^−^: 413.1442; found: 413.1440.

*N*-(7-(4-Acetamidophenyl)naphthalen-2-yl)-2-((4,6-dimethylpyrimidin-2-yl)thio)acetamide (**FM26**). At room temperature, acetic anhydride (9.03 µL, 0.0955 mmol, 2.00 eq) was added dropwise to a stirred solution of amine **9** (19.8 mg, 0.0478 mmol, 1.00 eq) and NEt_3_ (13.3 µL, 0.0955 mmol, 2.00 eq) in anhydrous DCM (5 mL). After 2 h, water (25 mL) and brine (25 mL) were added, and the reaction mixture was extracted with EtOAc (3 × 25 mL). The combined organic layers were dried over sodium sulfate. After evaporating the solvent in vacuo, the crude product was purified via flash column chromatography (hexanes/EtOAc 2:8) to yield the title compound as a white solid (15.6 mg, 0.0342 mmol, 72%). m.p.: 194 °C, R*f*: 0.23 (hexanes/EtOAc 2:8). ^1^H NMR (500 MHz, DMSO-*d_6_*) δ [ppm] = 10.46 (s, 1H, 2′-NHCO), 10.05 (s, 1H, 4′-NHCO), 8.32 (d, *J* = 2.0 Hz, 1H, 8-H), 8.06 (d, *J* = 1.8 Hz, 1H, 1-H), 7.89 (d, *J* = 8.6 Hz, 1H, 5-H), 7.87 (d, *J* = 8.8 Hz, 1H, 4-H), 7.80–7.73 (m, 2H, 2′-H, 6′-H), 7.73–7.68 (m, 3H, 3-H, 3′-H, 5′-H), 7.59 (dd, *J* = 8.8, 2.1 Hz, 1H, 6-H), 6.97 (s, 1H, 5″-H), 4.11 (s, 2H, CH_2_), 2.33 (s, 6H, 4″-CH_3_, 6″-CH_3_), 2.08 (s, 3H, CH_3_). ^1^^3^C NMR (126 MHz, DMSO-*d_6_*) δ [ppm] = 169.4 (C-2″), 168.4 (4′-NHCO), 167.0 (C-4″, C-6″), 166.8 (2″-NHCO), 138.9 (C-4′), 137.5 (C-7), 137.0 (C-2), 134.4 (C-1′), 133.8 (C-8a), 128.8 (C-4a), 128.2 (C-4), 128.1 (C-5), 127.2 (C-2′, C-6′), 124.0 (C-8), 123.5 (C-6), 119.8 (C-3), 119.3 (C-3′, C-5′), 116.1 (C-5″), 115.5 (C-1), 35.6 (CH_2_), 24.1 (CH_3_), 23.3 (4″-CH_3_, 6″-CH_3_). IR (ATR) ν῀ [cm^−1^] = 3250, 3050, 2926, 1658, 1578, 1521, 1504, 1424, 1400, 1369, 1337, 1316, 1291, 1266, 1239, 1224, 1180, 1164, 1015, 984, 956, 892, 836, 826, 804, 713. HRMS (ESI): *m/z* = [M-H]^−^ calculated for C_26_H_23_N_4_O_2_S^−^: 445.1547; found: 445.1546. Purity (HPLC): ≥98%.

*N*-(7-(3-Acetamidophenyl)naphthalen-2-yl)-2-((4,6-dimethylpyrimidin-2-yl)thio)acetamide (**FM46**). At room temperature, acetic anhydride (36.5 µL, 0.386 mmol, 2.00 eq) was added dropwise to a stirred solution of amine **10** (80.1 mg, 0.193 mmol, 1.00 eq) and NEt_3_ (53.9 µL, 0.386 mmol, 2.00 eq) in anhydrous DCM (5 mL). After 2 h, water (25 mL) and brine (25 mL) were added, and the reaction mixture was extracted with EtOAc (3 × 25 mL). The combined organic layers were dried over sodium sulfate. After evaporating the solvent in vacuo, the crude product was purified via flash column chromatography (hexanes/EtOAc 3:7) to yield the title compound as a white solid (80.2 mg, 0.176 mmol, 91%). m.p.: 213 °C, R*f*: 0.12 (hexanes/EtOAc 3:7). ^1^H NMR (500 MHz, DMSO-*d_6_*) δ [ppm] = 10.48 (s, 1H, 2-NHCO), 10.05 (s, 1H, 3′-NHCO), 8.33 (d, *J* = 2.1 Hz, 1H, 1-H), 8.01 (d, *J* = 1.8 Hz, 1H, 8-H), 8.00 (t, *J* = 2.0 Hz, 1H, 2′-H), 7.93 (d, *J* = 8.6 Hz, 1H, 5-H), 7.90 (d, *J* = 8.8 Hz, 1H, 4-H), 7.66 (dd, *J* = 8.5, 1.8 Hz, 1H, 6-H), 7.64–7.59 (m, 2H, 3-H, 4′-H), 7.47 (dt, *J* = 7.7, 1.5 Hz, 1H, 6′-H), 7.41 (t, *J* = 7.8 Hz, 1H, 5′-H), 6.97 (s, 1H, 5″-H), 4.12 (s, 2H, CH_2_), 2.34 (s, 6H, 4″-CH_3_, 6″-CH_3_), 2.08 (s, 3H, CH_3_). ^1^^3^C NMR (126 MHz, DMSO-*d_6_*) δ [ppm] = 169.3 (C-2″), 168.4 (3′-NHCO), 167.0 (C-4″, C-6″), 166.9 (2-NHCO), 140.5 (C-1′), 139.9 (C-3′), 138.1 (C-7), 137.1 (C-2), 133.7 (C-8a), 129.4 (C-5′), 129.0 (C-4a), 128.3 (C-4), 128.2 (C-5), 124.7 (C-8), 123.7 (C-6), 121.7 (C-6′), 120.1 (C-3), 118.2 (C-4′), 117.6 (C-2′), 116.1 (C-5″), 115.4 (C-1), 35.6 (CH_2_), 24.1 (4″-CH_3_, 6″-CH_3_), 23.3 (CH_3_). IR (ATR) ν῀ [cm^−1^] = 3290, 2923, 2854, 1665, 1609, 1584, 1559, 1542, 1489, 1433, 1394, 1372, 1320, 1268, 1218, 1194, 1172, 1150, 1022, 970, 901, 885, 838, 679, 778, 720, 692. HRMS (ESI): *m/z* = [M-H]^−^ calculated for C_26_H_23_N_4_O_2_S^−^: 445.1547; found: 445.1542. Purity (HPLC): >99%.

2-((4,6-Dimethylpyrimidin-2-yl)thio)-*N*-(7-(3-(phenylsulfonamido)phenyl)naphthalen-2-yl)acetamide (**FM47**). At room temperature, benzenesulfonyl chloride (49.3 µL, 0.386 mmol, 2.00 eq) was added dropwise to a stirred solution of amine **10** (80.1 mg, 0.193 mmol, 1.00 eq) and NEt_3_ (53.9 µL, 0.386 mmol, 2.00 eq) in anhydrous DCM (5 mL). After 4 h, water (25 mL) and brine (25 mL) were added, and the reaction mixture was extracted with EtOAc (4 × 25 mL). The combined organic layers were dried over sodium sulfate. After evaporating the solvent in vacuo, the crude product was purified via flash column chromatography (hexanes/EtOAc 1:1) to yield the title compound as a white solid (119 mg, 0.0899 mmol, 47%). m.p.: 176 °C (decomposition), R*f*: 0.19 (hexanes/EtOAc 1:1). ^1^H NMR (500 MHz, DMSO-*d_6_*) δ [ppm] = 10.49 (s, 1H, NHCO), 10.42 (s, 1H, NHSO_2_), 8.32 (d, *J* = 2.1 Hz, 1H, 1-H), 7.93–7.87 (m, 3H, 4-H, 5-H, 8-H), 7.85–7.79 (m, 2H, 2‴-H, 6‴-H), 7.64–7.55 (m, 4H, 3-H, 3‴-H, 4‴-H, 5‴-H), 7.54 (dd, *J* = 8.4, 1.8 Hz, 1H, 6-H), 7.46 (dt, *J* = 8.0, 1.1 Hz, 1H, 6′-H), 7.44 (t, *J* = 2.0 Hz, 1H, 2′-H), 7.35 (t, *J* = 7.9 Hz, 1H, 5′-H), 7.12 (ddd, *J* = 8.1, 2.2, 1.0 Hz, 1H, 4′-H), 6.97 (s, 1H, 5″-H), 4.11 (s, 2H, CH_2_), 2.34 (s, 6H, 4″-CH_3_, 6″-CH_3_). ^1^^3^C NMR (126 MHz, DMSO-*d_6_*) δ [ppm] = 169.3 (C-2″), 167.0 (C-4″, C-6″), 166.9 (NHCO), 141.0 (C-1′), 139.5 (C-1‴), 138.3 (C-3′), 137.4 (C-7), 137.2 (C-2), 133.6 (C-8a), 133.0 (C-4‴), 129.8 (C-5′), 129.3 (C-3‴, C-5‴), 129.1 (C-4a), 128.3 (C-5), 128.2 (C-4), 126.8 (C-2‴, C-6‴), 124.7 (C-8), 123.5 (C-6), 122.9 (C-6′), 120.2 (C-3), 119.2 (C-4′), 118.6 (C-2′), 116.1 (C-5″), 115.4 (C-1), 35.6 (CH_2_), 23.3 (4″-CH_3_, 6″-CH_3_). IR (ATR) ν῀ [cm^−1^] = 3294, 3211, 2912, 1670, 1581, 1530, 1511, 1489, 1446, 1385, 1330, 1309, 1265, 1226, 1165, 1094, 944, 906, 893, 846, 839, 786, 758, 737, 685. HRMS (ESI): *m/z* = [M-H]^−^ calculated for C_3__0_H_25_N_4_O_3_S_2_^−^: 553.1374; found: 553.1370. Purity (HPLC): >99%.

*N*-(3-(7-(2-((4,6-Dimethylpyrimidin-2-yl)thio)acetamido)naphthalen-2-yl)phenyl)thiophene-2-carboxamide (**FM48***).* At room temperature, 2-thiophenecarbonyl chloride (21.9 µL, 0.205 mmol, 2.00 eq) was added dropwise to a stirred solution of amine **10** (42.4 mg, 0.102 mmol, 1.00 eq) and NEt_3_ (28.5 µL, 0.205 mmol, 2.00 eq) in anhydrous DCM (5 mL). After 2 h, water (25 mL) and brine (25 mL) were added, and the reaction mixture was extracted with DCM (3 × 25 mL). The combined organic layers were dried over sodium sulfate. After evaporating the solvent in vacuo, the crude product was purified via flash column chromatography (hexanes/EtOAc 1:1) to yield the title compound as a white solid (16.3 mg, 0.0313 mmol, 31%). m.p.: 180 °C, R*f*: 0.21 (hexanes/EtOAc 1:1). ^1^H NMR (500 MHz, DMSO-*d_6_*) δ [ppm] = 10.49 (s, 1H, 2-NHCO), 10.33 (s, 1H, 3′-NHCO), 8.36 (d, *J* = 2.1 Hz, 1H, 1-H), 8.15 (t, *J* = 1.9 Hz, 1H, 2′-H), 8.09–8.05 (m, 2H, 8-H, 3‴-H), 7.96 (d, *J* = 8.5 Hz, 1H, 5-H), 7.91 (d, *J* = 8.9 Hz, 1H, 4-H), 7.88 (dd, *J* = 5.0, 1.2 Hz, 1H, 5‴-H), 7.81 (ddd, *J* = 8.0, 2.2, 1.1 Hz, 1H, 4-H), 7.72 (dd, *J* = 8.5, 1.8 Hz, 1H, 6-H), 7.63 (dd, *J* = 8.8, 2.1 Hz, 1H, 3-H), 7.56 (dt, *J* = 7.8, 1.4 Hz, 1H, 6′-H), 7.49 (t, *J* = 7.9 Hz, 1H, 5′-H), 7.25 (dd, *J* = 5.0, 3.7 Hz, 1H, 4‴-H), 6.97 (s, 1H, 5″-H), 4.12 (s, 2H, CH_2_), 2.34 (s, 6H, 4″-CH_3_, 6″-CH_3_). ^1^^3^C NMR (126 MHz, DMSO-*d_6_*) δ [ppm] = 169.3 (C-2″), 167.0 (C-4″, C-6″), 166.9 (2-NHCO), 160.0 (3′-NHCO), 140.5 (C-1′), 140.0 (C-2‴), 139.4 (C-3′), 137.9 (C-7), 137.2 (C-2), 133.8 (C-8a), 132.0 (C-5‴), 129.4 (C-5′), 129.2 (C-3‴), 129.1 (C-4a), 128.3 (C-5), 128.2 (C-4), 128.1 (C-4‴), 124.7 (C-8), 123.7 (C-6), 122.4 (C-6′), 120.1 (C-4), 119.4 (C-4′), 118.9 (C-2′), 116.1 (C-5″), 115.5 (C-1), 35.6 (CH_2_), 23.3 (4″-CH_3_, 6″-CH_3_). IR (ATR) ν῀ [cm^−1^] = 3299, 3078, 2957, 1660, 1634, 1608, 1583, 1538, 1487, 1428, 1335, 1306, 1265, 1227, 891, 841, 786, 718, 698. HRMS (ESI): *m/z* = [M-H]^−^ calculated for C_29_H_23_N_4_O_2_S_2_^−^: 525.1268; found: 523.1263. Purity (HPLC): >99%.

*N*-(4-(7-(2-((4,6-Dimethylpyrimidin-2-yl)thio)acetamido)naphthalen-2-yl)phenyl)thiophene-2-carboxamide (**FM50***).* At room temperature, 2-thiophenecarbonyl chloride (17.6 µL, 0.165 mmol, 2.00 eq) was added dropwise to a stirred solution of amine **9** (34.1 mg, 0.0823 mmol, 1.00 eq) and NEt_3_ (22.9 µL, 0.165 mmol, 2.00 eq) in anhydrous DCM (5 mL). After 2 h, water (25 mL) and brine (25 mL) were added, and the reaction mixture was extracted with EtOAc (3 × 25 mL). The combined organic layers were dried over sodium sulfate. After evaporating the solvent in vacuo, the crude product was purified via flash column chromatography (hexanes/EtOAc 1:1) to yield the title compound as a white solid (53.3 mg, 0.0438 mmol, 53%). m.p.: 195–198 °C, R*_f_*: 0.25 (hexanes/EtOAc 1:1). ^1^H NMR (500 MHz, DMSO-d_6_) δ [ppm] = 10.47 (s, 1H, 2-NHCO), 10.34 (s, 1H, 4′-NHCO), 8.33 (d, *J* = 2.0 Hz, 1H, 1-H), 8.11 (d, *J* = 1.9 Hz, 1H, 8-H), 8.06 (dd, *J* = 3.8, 1.2 Hz, 1H, 3‴-H), 7.91 (d, *J* = 8.6 Hz, 1H, 5-H), 7.90–7.85 (m, 4H, 4-H, 3′-H, 5′-H, 5‴-H), 7.84 (d, *J* = 8.9 Hz, 2H, 2′-H, 6′-H), 7.76 (dd, *J* = 8.5, 1.8 Hz, 1H, 6-H), 7.61 (dd, *J* = 8.8, 2.1 Hz, 1H, 3-H), 7.25 (dd, *J* = 5.0, 3.7 Hz, 1H, 4‴-H), 6.97 (s, 1H, 5″-H), 4.12 (s, 2H, CH_2_), 2.34 (s, 6H, 4″-CH_3_, 6″-CH_3_). ^1^^3^C NMR (126 MHz, DMSO-d_6_) δ [ppm] = 169.4 (C-2″), 167.0 (C-4″, C-6″), 166.8 (2-NHCO), 159.9 (4′-NHCO), 140.0 (C-2‴), 138.4 (C-4′), 137.4 (C-7), 137.0 (C-2), 135.1 (C-1′), 133.9 (C-8a), 132.0 (C-5‴), 129.2 (C-3‴), 128.9 (C-4a), 128.2 (C-5), 128.14 (C-4), 128.11 (C-4‴), 127.2 (C-2′, C-6′), 124.1 (C-8), 123.5 (C-6), 120.6 (C-3′, C-5′), 119.9 (C-3), 116.1 (C-5″), 115.5 (C-1), 35.6 (CH_2_), 23.3 (4″-CH_3_, 6″-CH_3_). IR (ATR) ν῀ [cm^−1^] = 3509, 3242, 1665, 1632, 1584, 1529, 1507, 1424, 1355, 1339, 1264, 1250, 1229, 1191, 984, 952, 901, 865, 834, 801, 732, 718. HRMS (ESI): *m*/*z* = [M+H]^+^ calculated for C_29_H_25_N_4_O_2_S_2_^+^: 525.1413; found: 525.1408. Purity (HPLC): ≥98%.

*N*-(4-(7-(2-((4,6-Dimethylpyrimidin-2-yl)thio)acetamido)naphthalen-2-yl)phenyl)benzamide (**FM53***).* At room temperature, benzoyl chloride (36.7 µL, 0.316 mmol, 2.00 eq) was added dropwise to a stirred solution of amine **9** (65.5 mg, 0.158 mmol, 1.00 eq) and NEt_3_ (44.0 µL, 0.316 mmol, 2.00 eq) in anhydrous DCM (5 mL). After 2 h, water (25 mL) and brine (25 mL) were added, and the reaction mixture was extracted with EtOAc (3 × 25 mL). The combined organic layers were dried over sodium sulfate. After evaporating the solvent in vacuo, the crude product was purified via flash column chromatography (DCM/MeOH 100:2) to yield the title compound as a white solid (64.1 mg, 0.124 mmol, 78%). m.p.: 207 °C, R*_f_*: 0.24 (DCM/MeOH 100:2). ^1^H NMR (500 MHz, DMSO-d_6_) δ [ppm] = 10.47 (s, 1H, 2-NHCO), 10.37 (s, 1H, 4′-NHCO), 8.34 (d, *J* = 2.0 Hz, 1H, 1-H), 8.11 (d, *J* = 1.9 Hz, 1H, 8-H), 8.01–7.97 (m, 2H, 2‴-H, 6‴-H), 7.96–7.90 (m, 3H, 5-H, 3′-H, 5′-H), 7.88 (d, *J* = 8.9 Hz, 1H, 4-H), 7.85–7.82 (m, 2H, 2′-H, 6′-H), 7.76 (dd, *J* = 8.5, 1.9 Hz, 1H, 6-H), 7.63–7.59 (m, 2H, 3-H, 4‴-H), 7.57–7.53 (m, 2H, 3‴-H, 5‴-H), 6.97 (s, 1H, 5″-H), 4.12 (s, 2H, CH_2_), 2.34 (s, 6H, 4″-CH_3_, 6″-CH_3_). ^1^^3^C NMR (126 MHz, DMSO-d_6_) δ [ppm] = 169.4 (C-2″), 167.0 (C-4″, C-6″), 166.8 (2-NHCO), 165.6 (4′-NHCO), 138.8 (C-4′), 137.5 (C-7), 137.0 (C-2), 135.0 (C-1′), 134.9 (C-1‴), 133.9 (C-8a), 131.6 (C-4‴), 128.8 (C-4a), 128.4 (C-3‴, C-5‴), 128.2 (C-4), 128.1 (C-5), 127.7 (C-2‴, C-6‴), 127.1 (C-2′, C-6′), 124.1 (C-8), 123.6 (C-6), 120.6 (C-3′, C-5′), 119.8 (C-3), 116.1 (C-5″), 115.5 (C-1), 35.6 (CH_2_), 23.3 (4″-CH_3_, 6″-CH_3_). IR (ATR) ν῀ [cm^−1^] = 3283, 3038, 1680, 1645, 1581, 1521, 1504, 1426, 1323, 1263, 892, 823, 795, 688. HRMS (ESI): *m*/*z* = [M-H]^−^ calculated for C_31_H_25_N_4_O_2_S^−^: 517.1704; found: 517.1701. Purity (HPLC): >99%.

*N*-(3-(7-(2-((4,6-Dimethylpyrimidin-2-yl)thio)acetamido)naphthalen-2-yl)phenyl)benzamide (**FM54***).* At room temperature, benzoyl chloride (34.7 µL, 0.299 mmol, 2.00 eq) was added dropwise to a stirred solution of amine **10** (62.0 mg, 0.150 mmol, 1.00 eq) and NEt_3_ (41.7 µL, 0.299 mmol, 2.00 eq) in anhydrous DCM (5 mL). After 2 h, water (25 mL) and brine (25 mL) were added, and the reaction mixture was extracted with DCM (3 × 25 mL). The combined organic layers were dried over sodium sulfate. After evaporating the solvent in vacuo, the crude product was purified via flash column chromatography (hexanes/EtOAc 1:1) to yield the title compound as a white solid (68.0 mg, 0.131 mmol, 87%). m.p.: 192 °C, R*_f_*: 0.20 (hexanes/EtOAc 1:1). ^1^H NMR (500 MHz, DMSO-d_6_) δ [ppm] = 10.49 (s, 1H, 2-NHCO), 10.35 (s, 1H, 3′-NHCO), 8.36 (d, *J* = 2.1 Hz, 1H, 1-H), 8.23 (t, *J* = 1.9 Hz, 1H, 2′-H), 8.07 (d, *J* = 1.8 Hz, 1H, 8-H), 8.02–7.99 (m, 2H, 2‴-H, 6‴-H), 7.96 (d, *J* = 8.6 Hz, 1H, 5-H), 7.91 (d, *J* = 8.8 Hz, 1H, 4-H), 7.87 (ddd, *J* = 8.1, 2.2, 1.1 Hz, 1H, 4′-H), 7.72 (dd, *J* = 8.5, 1.8 Hz, 1H, 6-H), 7.65–7.59 (m, 2H, 3-H, 4‴-H), 7.58–7.54 (m, 3H, 6′-H, 3‴-H, 5‴-H), 7.49 (t, *J* = 7.9 Hz, 1H, 5′-H), 6.97 (s, 1H, 5″-H), 4.12 (s, 2H, CH_2_), 2.34 (s, 6H, 4″-CH_3_, 6″-CH_3_). ^1^^3^C NMR (126 MHz, DMSO-d_6_) δ [ppm] = 169.3 (C-2″), 167.0 (C-4″, C-6″), 166.9 (2-NHCO), 165.6 (3′-NHCO), 140.4 (C-1′), 139.7 (C-3′), 138.0 (C-7), 137.2 (C-2), 134.8 (C-1‴), 133.8 (C-8a), 131.7 (C-4‴), 129.3 (C-5′), 129.1 (C-4a), 128.4 (C-3‴, C-5‴), 128.3 (C-4), 128.2 (C-5), 127.7 (C-2‴, C-6‴), 124.7 (C-8), 123.7 (C-6), 122.3 (C-6′), 120.1 (C-3), 119.4 (C-4′), 118.9 (C-2′), 116.1 (C-5″), 115.5 (C-1), 35.6 (CH_2_), 23.3 (4″-CH_3_, 6″-CH_3_). IR (ATR) ν῀ [cm^−1^] = 3282, 3059, 1667, 1649, 1608, 1581, 1537, 1487, 1438, 1396, 1370, 1333, 1299, 1265, 1222, 1027, 889, 838, 787, 697. HRMS (ESI): *m*/*z* = [M-H]^−^ calculated for C_31_H_25_N_4_O_2_S^−^: 517.1704; found: 517.1701. Purity (HPLC): >99%.

2-((4,6-Dimethylpyrimidin-2-yl)thio)-*N*-(7-(4-(phenylsulfonamido)phenyl)naphthalen-2-yl)acetamide (**FM56**). At room temperature, benzenesulfonyl chloride (19.9 µL, 0.156 mmol, 0.95 eq) was added dropwise to a stirred solution of amine **9** (67.9 mg, 0.164 mmol, 1.00 eq) and NEt_3_ (21.7 µL, 0.156 mmol, 0.95 eq) in anhydrous DCM (5 mL). After heating to reflux for 7h, water (25 mL) and brine (25 mL) were added, and the reaction mixture was extracted with DCM (3 × 25 mL). The combined organic layers were dried over sodium sulfate. After evaporating the solvent in vacuo, the crude product was purified via flash column chromatography (DCM/MeOH 120:1) to yield the title compound as a white solid (39.5 mg, 0.0617 mmol, 40%). m.p.: 135 °C (decomposition), R*_f_*: 0.04 (DCM/MeOH 120:1). ^1^H NMR (500 MHz, DMSO-*d*_6_) δ [ppm] = 10.45 (s, 2H, NHCO, NHSO_2_), 8.29 (d, *J* = 2.0 Hz, 1H, 1-H), 8.00 (d, *J* = 1.8 Hz, 1H, 8-H), 7.87 (d, *J* = 6.0 Hz, 1H, 5-H), 7.85 (d, *J* = 6.2 Hz, 1H, 4-H), 7.83–7.80 (m, 2H, 2‴-H, 6‴-H), 7.71–7.68 (m, 2H, 2′-H, 6′-H), 7.65 (dd, *J* = 8.6, 1.9 Hz, 1H, 6-H), 7.63–7.55 (m, 4H, 3-H, 3‴-H, 4‴-H, 5‴-H), 7.24–7.19 (m, 2H, 3′-H, 5′-H), 6.97 (s, 1H, 5″-H), 4.10 (s, 2H, CH_2_), 2.33 (s, 6H, 4″-CH_3_, 6″-CH_3_). ^13^C NMR (126 MHz, DMSO-*d*_6_) δ [ppm] = 169.3 (C-2″), 167.0 (C-4″, C-6″), 166.8 (NHCO), 139.6 (C-1‴), 137.2 (C-4′), 137.1 (C-7), 137.0 (C-2), 135.5 (C-1′), 133.7 (C-8a), 133.0 (C-4‴), 129.4 (C-3‴, C-5‴), 128.8 (C-4a), 128.14 (C-5), 128.11 (C-4), 127.7 (C-2′, C-6′), 126.6 (C-2‴, C-6‴), 124.2 (C-8), 123.5 (C-6), 120.2 (C-3′, C-5′), 119.9 (C-3), 116.1 (C-5″), 115.4 (C-1), 35.6 (CH_2_), 23.3 (4″-CH_3_, 6″-CH_3_). IR (ATR) *ν῀*[cm^−1^] = 3056, 2923, 2854, 1668, 1583, 1532, 1502, 1329, 1265, 1158, 1091, 902, 830, 720, 687. HRMS (ESI): *m*/*z* = [M-H]^-^ calculated for C_30_H_25_N_4_O_3_S_2_^-^: 553.1374; found: 553.1373. Purity (HPLC): >99%.

*N*-(3-(7-(2-((4,6-Dimethylpyrimidin-2-yl)thio)acetamido)naphthalen-2-yl)phenyl)-5-methylthiophene-2-carboxamide (**FM66**). At room temperature, previously prepared 5-methylthiophene-2-carbonyl chloride (27.7 µL, 0.228 mmol, 2.00 eq) was added dropwise to a stirred solution of amine **10** (47.2 mg, 0.114 mmol, 1.00 eq) and NEt_3_ (31.7 µL, 0.228 mmol, 2.00 eq) in anhydrous DCM (5 mL). After 5 h, water (25 mL) and brine (25 mL) were added, and the reaction mixture was extracted with DCM (3 × 25 mL). The combined organic layers were dried over sodium sulfate. After evaporating the solvent in vacuo, the crude product was purified via flash column chromatography (hexanes/EtOAc 1:1) to yield the title compound as a white solid (51.0 mg, 0.0947 mmol, 83%). m.p.: 122 °C (decomposition), R*_f_*: 0.28 (hexanes/EtOAc 1:1). ^1^H NMR (500 MHz, DMSO-d_6_) δ [ppm] = 10.49 (s, 1H, 2-NHCO), 10.20 (s, 1H, 3′-NHCO), 8.35 (d, *J* = 2.0 Hz, 1H, 1-H), 8.14 (t, *J* = 2.0 Hz, 1H, 2′-H), 8.07 (d, *J* = 1.8 Hz, 1H, 8-H), 7.95 (d, *J* = 8.6 Hz, 1H, 5-H), 7.91 (d, *J* = 8.9 Hz, 1H, 4-H), 7.87 (d, *J* = 3.7 Hz, 1H, 3‴-H), 7.79 (ddd, *J* = 8.0, 2.1, 1.0 Hz, 1H, 4′-H), 7.71 (dd, *J* = 8.5, 1.8 Hz, 1H, 6-H), 7.63 (dd, *J* = 8.8, 2.0 Hz, 1H, 3-H), 7.54 (dt, *J* = 7.9, 1.4 Hz, 1H, 6′-H), 7.47 (t, *J* = 7.9 Hz, 1H, 5′-H), 6.97 (s, 1H, 5″-H), 6.95 (dd, *J* = 3.8, 1.2 Hz, 1H, 4‴-H), 4.12 (s, 2H, CH_2_), 2.51 (s, 3H, CH_3_), 2.34 (s, 6H, 4″-CH_3_, 6″-CH_3_). ^1^^3^C NMR (126 MHz, DMSO-d_6_) δ [ppm] = 169.3 (2″-H), 167.0 (C-4″, C-6″), 166.9 (2-NHCO), 159.9 (3′-NHCO), 146.0 (C-5‴), 140.5 (C-1′), 139.5 (C-3′), 137.9 (C-7), 137.4 (C-2‴), 137.1 (C-2), 133.8 (C-8a), 129.4 (C-3‴), 129.3 (C-5′), 129.1 (C-4a), 128.3 (C-5), 128.2 (C-4), 126.7 (C-4‴), 124.7 (C-8), 123.7 (C-6), 122.2 (C-6′), 120.1 (C-3), 119.3 (C-4′), 118.8 (C-2′), 116.1 (C-5″), 115.5 (C-1), 35.6 (CH_2_), 23.3 (4″-CH_3_, 6″-CH_3_), 15.3 (CH_3_). IR (ATR) ν῀ [cm^−1^] = 3287, 3059, 2920, 1633, 1607, 1582, 1531, 1510, 1486, 1459, 1428, 1398, 1336, 1303, 1263, 1171, 1085, 1032, 891, 839, 801, 785, 730, 697. HRMS (EI): *m*/*z* = [M]^•^^+^ calculated for C_3__0_H_26_N_4_O_2_S_2_^•^^+^: 538.1492; found: 538.1500. Purity (HPLC): >96%.

*N*-(4-(7-(2-((4,6-Dimethylpyrimidin-2-yl)thio)acetamido)naphthalen-2-yl)phenyl)-5-methylthiophene-2-carboxamide (**FM69**). At room temperature, previously prepared 5-methylthiophene-2-carbonyl chloride (24.5 µL, 0.201 mmol, 2.00 eq) was added dropwise to a stirred solution of amine **9** (41.7 mg, 0.101 mmol, 1.00 eq) and NEt_3_ (28.0 µL, 0.201 mmol, 2.00 eq) in anhydrous DCM (5 mL). After heating to reflux for 3 h, water (25 mL) and brine (25 mL) were added, and the reaction mixture was extracted with DCM (3 × 25 mL). The combined organic layers were dried over sodium sulfate. After evaporating the solvent in vacuo, the crude product was purified via flash column chromatography (DCM/MeOH 120:1) to yield the title compound as a white solid (40.2 mg, 0.0746 mmol, 74%). m.p.: 191–193 °C, R*_f_*: 0.07 (DCM/MeOH 120:1). ^1^H NMR (500 MHz, DMSO-d_6_) δ [ppm] = 10.47 (s, 1H, 2-NHCO), 10.22 (s, 1H, 4′-NHCO), 8.33 (d, *J* = 2.0 Hz, 1H, 1-H), 8.10 (d, *J* = 1.8 Hz, 1H, 8-H), 7.91 (d, *J* = 8.6 Hz, 1H, 5-H), 7.89–7.79 (m, 6H, 4-H, 2′-H, 3′-H, 5′-H, 6′-H, 3‴-H), 7.75 (dd, *J* = 8.5, 1.8 Hz, 1H, 6-H), 7.60 (dd, *J* = 8.8, 2.1 Hz, 1H, 3-H), 6.97 (s, 1H, 5″-H), 6.95–6.93 (m, 1H, 4‴-H), 4.11 (s, 2H, CH_2_), 2.51 (s, 3H, CH_3_), 2.34 (s, 6H, 4″-CH_3_, 6″-CH_3_). ^1^^3^C NMR (126 MHz, DMSO-d_6_) δ [ppm] = 169.4 (C-2″), 167.0 (C-4″, C-6″), 166.8 (2-NHCO), 159.8 (4′-NHCO), 146.0 (C-5‴), 138.5 (C-4′), 137.43 (C-7, C-2‴), 137.0 (C-2), 134.9 (C-1′), 133.9 (C-8a), 129.5 (C-3‴), 128.8 (C-4a), 128.2 (C-5), 128.1 (C-4), 127.1 (C-2′, C-6′), 126.7 (C-4‴), 124.1 (C-8), 123.5 (C-6), 120.5 (C-3′, C-5′), 119.8 (C-3), 116.1 (C-5″), 115.5 (C-1), 35.6 (CH_2_), 23.3 (4″-CH_3_, 6″-CH_3_), 15.3 (CH_3_). IR (ATR) ν῀ [cm^−1^] = 3446, 3241, 2914, 1663, 1613, 1583, 1526, 1505, 1458, 1398, 1338, 1320, 1264, 1249, 1228, 1190, 1170, 1093, 984, 952, 901, 873, 832, 802, 741. HRMS (EI): *m*/*z* = [M]^•^^+^ calculated for C_3__0_H_26_N_4_O_2_S_2_^•^^+^: 538.1492; found: 538.1494. Purity (HPLC): >98%.

6-(3-Nitrophenyl)benzo[*d*]thiazol-2-amine (**13***).* 2-Amino-6-bromobenzothiazole (**12**, 1.86 g, 8.14 mmol, 1.00 eq) and Pd(PPh_3_)_4_ (0.941 g, 0.814 mmol, 0.100 eq) were weighed out into a flask, which was put under nitrogen afterwards. Degassed 1,4-dioxane (40 mL) was added via syringe, and the mixture was stirred for 10 min at room temperature before 3-nitrophenyl boronic acid (1.63 g, 9.77 mmol, 1.20 eq), Cs_2_CO_3_ (13.3 g, 40.7 mmol, 5.00 eq), and degassed water (17.5 mL) were added under nitrogen atmosphere. The reaction mixture was stirred for 21 h at 80 °C, cooled to room temperature, diluted with water (200 mL), and extracted with EtOAc (4 × 100 mL). The combined organic layers were dried over sodium sulfate. After evaporating the solvent in vacuo, the crude product was purified via flash column chromatography (hexanes/EtOAc 7:3) to yield the title compound as an orange solid (1.35 g, 4.97 mmol, 61%). m.p.: 218–219 °C (decomposition), R*_f_*: 0.05 (hexanes/EtOAc 7:3). ^1^H NMR (500 MHz, CDCl_3_) δ [ppm] = 8.46 (t, *J* = 2.1 Hz, 1H, 2′-H), 8.18 (ddd, *J* = 8.2, 2.3, 1.0 Hz, 1H, 4′-H), 7.92 (ddd, *J* = 7.7, 1.9, 1.0 Hz, 1H, 6′-H), 7.87 (d, *J* = 2.1 Hz, 1H, 7-H), 7.67–7.56 (m, 3H, 4-H, 5-H, 5′-H), 5.30 (s, 2H, NH_2_). ^1^^3^C NMR (126 MHz, CDCl_3_) δ [ppm] = 166.5 (C-2), 152.6 (C-3a), 148.9 (C-3′), 142.8 (C-1′), 133.3 (C-6), 133.1 (C-7a), 133.0 (C-6′), 129.9 (C-5′), 125.5 (C-5), 121.9 (C-2′), 121.9 (C-4′), 119.9 (C-4), 119.7 (C-7). IR (ATR) ν῀ [cm^−1^] = 3422, 3290, 3066, 1638, 1599, 1530, 1512, 1486, 1456, 1345, 1304, 1275, 1094, 1064, 906, 876, 862, 801, 742, 728, 714, 685, 674. HRMS (EI): *m*/*z* = [M]^•^^+^ calculated for C_13_H_9_N_3_O_2_S^•^^+^: 271.0410; found: 271.0411.

*tert*-Butyl (4-(2-aminobenzo[*d*]thiazol-6-yl)phenyl)carbamate (**14***).* 2-Amino-6-bromobenzothiazole (**12**, 1.21 g, 5.28 mmol, 1.00 eq) and Pd(PPh_3_)_4_ (610 mg, 0.528 mmol, 0.100 eq) were weighed out into a flask, which was put under nitrogen afterwards. Degassed 1,4-dioxane (30 mL) was added via syringe, and the mixture was stirred for 10 min at room temperature before 4-(*N*-boc-amino)phenylboronic acid pinacol ester (2.02 g, 6.34 mmol, 1.20 eq), Cs_2_CO_3_ (8.60 g, 26.4 mmol, 5.00 eq), and degassed water (10 mL) were added under nitrogen atmosphere. The reaction mixture was stirred for 20 h at 80 °C, cooled to room temperature, diluted with water (250 mL), and extracted with EtOAc (4 × 100 mL). The combined organic layers were dried over sodium sulfate. After evaporating the solvent in vacuo, the crude product was purified via flash column chromatography (hexanes/EtOAc 1:1) to yield the title compound as a light-yellow solid (498 mg, 1.46 mmol, 28%). m.p.: 320 °C (decomposition), R*_f_*: 0.16 (hexanes/EtOAc 1:1). ^1^H NMR (500 MHz, DMSO-d_6_) δ [ppm] = 9.40 (s, 1H, NHCO), 7.92 (d, *J* = 1.9 Hz, 1H, 7-H), 7.57–7.54 (m, 2H, 2′-H, 6′-H), 7.53–7.48 (m, 4H, 3′-H, 5′-H, NH_2_), 7.47 (dd, *J* = 8.4, 1.9 Hz, 1H, 5-H), 7.36 (d, *J* = 8.3 Hz, 1H, 4-H), 1.49 (s, 9H, C(CH_3_)_3_). ^1^^3^C NMR (126 MHz, DMSO-d_6_) δ [ppm] = 166.6 (NHCO), 152.8 (C-2), 151.8 (C-3a), 138.4 (C-4′), 133.9 (C-1′), 132.9 (C-6), 131.8 (C-7a), 126.5 (C-2′, C-6′), 123.8 (C-5), 118.43 (C-7), 118.38 (C-3′, C-5′), 117.8 (C-4), 79.1 (C(CH_3_)_3_), 28.1 (C(CH_3_)_3_). IR (ATR) ν῀ [cm^−1^] = 3351, 2981, 2932, 1699, 1635, 1588, 1522, 1504, 1459, 1417, 1389, 1367, 1314, 1301, 1233, 1160, 1110, 1053, 1023, 835, 812, 772, 762. HRMS (EI): *m*/*z* = [M]^•^^+^ calculated for C_18_H_19_N_3_O_2_S^•^^+^: 341.1192; found: 341.1193.

2-Bromo-*N*-(6-(3-nitrophenyl)benzo[*d*]thiazol-2-yl)acetamide (**15***).* At 0 °C, bromoacetyl bromide (851 µL, 9.77 mmol, 1.20 eq) was added dropwise to a stirred suspension of amine **13** (2.21 g, 8.14 mmol, 1.00 eq) and NEt_3_ (1.36 mL, 9.77 mmol, 1.20 eq) in anhydrous DCM/DMF (6:1, 35 mL). After heating to reflux for 3 h, water (150 mL) and brine (50 mL) were added, and the reaction mixture was extracted with EtOAc (4 × 100 mL). The combined organic layers were dried over sodium sulfate. After evaporating the solvent in vacuo, the crude product was purified via flash column chromatography (hexanes/EtOAc 8:2) to yield the title compound as a light-yellow solid (827 mg, 2.11 mmol, 26%). m.p.: 186 °C, R*_f_*: 0.07 (hexanes/EtOAc 8:2). ^1^H NMR (500 MHz, CDCl_3_) δ [ppm] = 9.74 (s, 1H, NHCO), 8.51 (t, *J* = 2.0 Hz, 1H, 2′-H), 8.23 (ddd, *J* = 8.2, 2.2, 1.0 Hz, 1H, 4′-H), 8.09 (dd, *J* = 1.9, 0.6 Hz, 1H, 7-H), 7.97 (ddd, *J* = 7.7, 1.8, 1.0 Hz, 1H, 6′-H), 7.92 (dd, *J* = 8.4, 0.6 Hz, 1H, 4-H), 7.73 (dd, *J* = 8.5, 1.9 Hz, 1H, 5-H), 7.65 (t, *J* = 8.0 Hz, 1H, 5′-H), 4.15 (s, 2H, CH_2_). ^1^^3^C NMR (126 MHz, CDCl_3_ δ [ppm] = 164.2 (NHCO), 157.9 (C-2), 149.0 (C-3′), 148.7 (C-3a), 142.5 (C-1′), 135.4 (C-6), 133.6 (C-7a), 133.3 (C-6′), 130.1 (C-5′), 126.1 (C-5), 122.3 (C-4′), 122.3 (C-2′), 122.1 (C-4), 120.3 (C-7), 27.9 (CH_2_). IR (ATR) ν῀ [cm^−1^] = 2968, 1653, 1610, 1574, 1548, 1530, 1452, 1342, 1320, 1281, 1111, 1000, 874, 863, 825, 799, 770, 746, 730, 718, 679. HRMS (EI): *m/z* = [M]^•^^+^ calculated for C_15_H_1__0_^7^^9^BrN_3_O_3_S^•^^+^: 390.9621; found: 390.9615.

*tert*-Butyl (4-(2-(2-bromoacetamido)benzo[*d*]thiazol-6-yl)phenyl)carbamate (**16***).* At 0 °C, bromoacetyl bromide (206 µL, 2.37 mmol, 1.00 eq) was added dropwise to a stirred suspension of amine **14** (809 mg, 2.37 mmol, 1.00 eq) and NEt_3_ (661 µL, 4.74 mmol, 2.00 eq) in anhydrous EtOAc (20 mL). After heating to reflux for 3 h, water (100 mL) and brine (50 mL) were added, and the reaction mixture was extracted with EtOAc (4 × 100 mL). The combined organic layers were dried over sodium sulfate. After evaporating the solvent in vacuo, the crude product was purified via flash column chromatography (hexanes/EtOAc 7:3) to yield the title compound as a light-yellow solid (495 mg, 1.07 mmol, 45%). m.p.: 300 °C (decomposition), R*_f_*: 0.28 (hexanes/EtOAc 7:3). ^1^H NMR (500 MHz, CDCl_3_) δ [ppm] = 8.00 (d, *J* = 1.8 Hz, 1H, 7-H), 7.85 (d, *J* = 8.4 Hz, 1H, 4-H), 7.68 (dd, *J* = 8.5, 1.8 Hz, 1H, 5-H), 7.60–7.55 (m, 2H, 2′-H, 6′-H), 7.50–7.43 (m, 2H, 3′-H, 5′-H), 7.26 (s, 1H, 4-NHCO), 6.59 (bs, 1H, 4′-NHCO), 4.14 (s, 2H, CH_2_), 1.55 (s, 9H, C(CH_3_)_3_). ^1^^3^C NMR (126 MHz, CDCl_3_) δ [ppm] = 164.2 (2-NHCO), 157.5 (4′-NHCO), 152.9 (C-2), 146.7 (C-3a), 138.1 (C-4′), 137.8 (C-6), 135.3 (C-1′), 132.9 (C-7a), 128.0 (C-2′, C-6′), 126.1 (C-5), 121.3 (C-4), 119.5 (C-7), 119.1 (C-3′, C-5′), 80.9 (C(CH_3_)_3_), 28.5 (C(CH_3_)_3_), 27.9 (CH_2_). IR (ATR) ν῀ [cm^−1^] = 3370, 3215, 1687, 1602, 1543, 1524, 1502, 1454, 1393, 1368, 1329, 1299, 1272, 1234, 1158, 1058, 982, 834, 812, 754, 720. HRMS (EI): *m*/*z* = [M-H]^−^ calculated for C_2__0_H_19_^7^^9^BrN_3_O_3_S^−^: 460.0338; found: 460.0336.

2-((4,6-Dimethylpyrimidin-2-yl)thio)-*N*-(6-(3-nitrophenyl)benzo[*d*]thiazol-2-yl)acetamide (**17***).* At room temperature, potassium *tert*-butoxide (236 mg, 2.10 mmol, 2.00 eq) was added to a stirred solution of 2-bromoacetamide **15** (413 mg, 1.05 mmol, 1.00 eq) and 4,6-dimethylpyrimidine-2-thiol (177 mg, 1.26 mmol, 1.20 eq) in anhydrous DMF (5 mL). After 3 h, water (50 mL) and brine (100 mL) were added, and the reaction mixture was extracted with EtOAc (3 × 100 mL). The combined organic layers were dried over sodium sulfate. After evaporating the solvent in vacuo, the crude product was purified via flash column chromatography (hexanes/EtOAc 7:3) to yield the title compound as a yellow solid (319 mg, 0.707 mmol, 67%). m.p.: 205 °C, R*_f_*: 0.09 (hexanes/EtOAc 7:3). ^1^H NMR (400 MHz, DMSO-d_6_) δ [ppm] = 12.75 (s, 1H, NHCO), 8.51 (t, *J* = 2.1 Hz, 1H, 2′-H), 8.49–8.44 (m, 1H, 7-H), 8.24–8.19 (m, 2H, 4′-H, 6′-H), 7.90–7.85 (m, 2H, 4-H, 5-H), 7.78 (t, *J* = 8.0 Hz, 1H, 5′-H), 6.97 (s, 1H, 5″-H), 4.20 (s, 2H, CH_2_), 2.29 (s, 6H, 4″-CH_3_, 6″-CH_3_). ^1^^3^C NMR (101 MHz, DMSO-d_6_) δ [ppm] = 168.8 (NHCO), 168.3 (C-2″), 167.1 (C-4″, C-6″), 159.1 (C-2), 148.9 (C-3a), 148.5 (C-3′), 141.6 (C-1′), 133.3 (C-6′), 133.2 (C-6), 132.6 (C-7a), 130.5 (C-5′), 125.4 (C-5), 121.9 (C-4′), 121.1 (C-2′), 121.0 (C-4), 120.5 (C-7), 116.2 (C-5″), 34.5 (CH_2_), 23.2 (4″-CH_3_, 6″-CH_3_). IR (ATR) ν῀ [cm^−1^] = 2985, 1694, 1666, 1608, 1564, 1526, 1452, 1342, 1304, 1262, 1156, 893, 864, 800, 745, 730, 716, 676. HRMS (EI): *m*/*z* = [M]^•^^+^ calculated for C_21_H_17_N_5_O_3_S_2_•^+^: 451.0767; found: 451.0762.

*tert*-Butyl (4-(2-(2-((4,6-dimethylpyrimidin-2-yl)thio)acetamido)benzo[*d*]thiazol-6 yl)phenyl)carbamate (**18***).* At room temperature, potassium *tert*-butoxide (274 mg, 2.44 mmol, 2.00 eq) was added to a stirred solution of 2-bromoacetamide **16** (564 mg, 1.22 mmol, 1.00 eq) and 4,6-dimethylpyrimidine-2-thiol (205 mg, 1.46 mmol, 1.20 eq) in anhydrous DMF (5 mL). After 3 h, water (50 mL) and brine (100 mL) were added, and the reaction mixture was extracted with EtOAc (4 × 100 mL). The combined organic layers were dried over sodium sulfate. After evaporating the solvent in vacuo, the title compound was yielded as a yellow solid (499 mg, 0.956 mmol, 78%). m.p.: 220 °C, R*_f_*: 0.10 (hexanes/EtOAc 7:3). ^1^H NMR (400 MHz, DMSO-d_6_) δ [ppm] = 12.64 (s, 1H, 2-NHCO), 9.44 (s, 1H, 4′-NHCO), 8.23 (d, *J* = 1.8 Hz, 1H, 7-H), 7.78 (d, *J* = 8.5 Hz, 1H, 4-H), 7.70 (dd, *J* = 8.5, 1.9 Hz, 1H, 5-H), 7.65–7.60 (m, 2H, 2′-H, 6′-H), 7.59–7.51 (m, 2H, 3′-H, 5′-H), 6.96 (s, 1H, 5″-H), 4.19 (s, 2H, CH_2_), 2.29 (s, 6H, 4″-CH_3_, 6″-CH_3_), 1.49 (s, 9H, C(CH_3_)_3_). ^1^^3^C NMR (101 MHz, DMSO-d_6_) δ [ppm] = 168.8 (C-2″), 168.1 (2-NHCO), 167.0 (C-4″, C-6″), 158.0 (C-2), 152.7 (4′-NHCO), 147.7 (C-3a), 138.9 (C-4′), 135.5 (C-6), 133.5 (C-1′), 132.4 (C-7a), 126.9 (C-2′, C-6′), 124.7 (C-5), 120.7 (C-4), 119.0 (C-7), 118.4 (C-3′, C-5′), 116.2 (C-5″), 79.1 (C(CH_3_)_3_), 34.4 (CH_2_), 28.13 (C(CH_3_)_3_), 23.2 (4″-CH_3_, 6″-CH_3_). IR (ATR) ν῀ [cm^−1^] = 2976, 1720, 1700, 1583, 1536, 1458, 1367, 1326, 1264, 1233, 1154, 1119, 1053, 1024, 846, 819, 779, 478. HRMS (EI): *m/z* = [M]^•^^+^ calculated for C_26_H_27_N_5_O_3_S_2_^•^^+^: 521.1550; found: 521.1550.

*N*-(6-(3-Aminophenyl)benzo[*d*]thiazol-2-yl)-2-((4,6-dimethylpyrimidin-2-yl)thio)acetamide (**19**). Under nitrogen atmosphere, nitro compound **17** (317 mg, 0.808 mmol, 1.00 eq) was suspended in MeOH/water (10:1, 2.2 mL), and iron powder (266 mg, 4.04 mmol, 5.00 eq) and ammonium chloride (216 mg, 4.04 mmol, 5.00 eq) were added. The reaction mixture was heated to reflux for 3 h, and, after cooling, it was diluted with water (100 mL). Subsequently, the mixture was extracted with EtOAc (4 × 50 mL), and the combined organic layers were dried over sodium sulfate. After evaporating the solvent in vacuo, the crude product was purified via flash column chromatography (hexanes/EtOAc 1:1) to yield the title compound as a light-yellow solid (251 mg, 0.596 mmol, 74%). m.p.: 118–122 °C, R*_f_*: 0.12 (hexanes/EtOAc 1:1). ^1^H NMR (400 MHz, DMSO-d_6_) δ [ppm] = 12.65 (s, 1H, NHCO), 8.14 (d, *J* = 1.8 Hz, 1H, 7-H), 7.79 (d, *J* = 8.4 Hz, 1H, 4-H), 7.62 (dd, *J* = 8.5, 1.9 Hz, 1H, 5-H), 7.10 (t, *J* = 7.8 Hz, 1H, 5′-H), 6.96 (s, 1H, 5″-H), 6.88 (t, *J* = 2.0 Hz, 1H, 2′-H), 6.82 (ddd, *J* = 7.6, 1.8, 1.0 Hz, 1H, 6′-H), 6.56 (ddd, *J* = 7.9, 2.2, 1.0 Hz, 1H, 4′-H), 5.15 (s, 2H, NH_2_), 4.19 (s, 2H, CH_2_), 2.29 (s, 6H, 4″-CH_3_, 6″-CH_3_). ^1^^3^C NMR (101 MHz, DMSO-d_6_) δ [ppm] = 168.8 (NHCO), 168.1 (C-2″), 167.0 (C-4″, C-6″), 158.1 (C-2), 149.1 (C-3′), 147.9 (C-3a), 140.7 (C-1′), 136.8 (C-6), 132.3 (C-7a), 129.4 (C-5′), 125.0 (C-5), 120.6 (C-4), 119.4 (C-7), 116.1 (C-5″), 114.5 (C-6′), 113.0 (C-4′), 112.3 (C-2′), 34.5 (CH_2_), 23.3 (4″-CH_3_, 6″-CH_3_). IR (ATR) ν῀ [cm^−1^] = 2921, 1693, 1602, 1583, 1560, 1532, 1453, 1265, 1135, 864, 824, 782, 733, 694. HRMS (EI): *m*/*z* = [M]^•^^+^ calculated for C_21_H_19_N_5_OS_2_^•^^+^: 421.1026; found: 421.1011.

*N*-(6-(4-Aminophenyl)benzo[*d*]thiazol-2-yl)-2-((4,6-dimethylpyrimidin-2-yl)thio)acetamide (**20**). At room temperature, Boc-protected amine **18** (1.12 g, 2.15 mmol, 1.00 eq) was suspended in chloroform (10 mL), and TFA (3.22 mL, 42.9 mmol, 25.0 eq) was added. After the reaction mixture was stirred for 24 h, it was alkalized with saturated NaHCO_3_ solution and extracted with DCM (4 × 50 mL). The combined organic layers were dried over sodium sulfate. After evaporating the solvent in vacuo, the crude product was purified via flash column chromatography (hexanes/EtOAc 1:1) to yield the title compound as a beige solid (755 mg, 1.79 mmol, 83%). m.p.: 146 °C (decomposition), R*_f_*: 0.12 (hexanes/EtOAc 1:1). ^1^H NMR (500 MHz, DMSO-d_6_) δ [ppm] = 12.58 (s, 1H, NHCO), 8.11 (d, *J* = 1.8 Hz, 1H, 7-H), 7.72 (d, *J* = 8.4 Hz, 1H, 4-H), 7.61 (dd, *J* = 8.5, 1.9 Hz, 1H, 5-H), 7.44–7.37 (m, 2H, 2′-H, 6′-H), 6.97 (s, 1H, 5″-H), 6.68–6.61 (m, 2H, 3′-H, 5′-H), 5.22 (s, 2H, NH_2_), 4.18 (s, 2H, CH_2_), 2.30 (s, 6H, 4″-CH_3_, 6″-CH_3_). ^1^^3^C NMR (101 MHz, DMSO-d_6_) δ [ppm] = 168.9 (C-2″), 168.0 (NHCO), 167.1 (C-4″, C-6″), 157.4 (C-2), 148.3 (C-4′), 146.8 (C-3a), 136.6 (C-6), 132.4 (C-7a), 127.7 (C-2′, C-6′), 127.3 (C-1′), 124.9 (C-5), 124.1 (C-4), 120.6 (C-7), 117.9 (C-5″), 116.2 (C-3′, C-5′), 34.4 (CH_2_), 23.3 (4″-CH_3_, 6″-CH_3_). IR (ATR) ν῀ [cm^−1^] = 2922, 1692, 1603, 1583, 1536, 1455, 1265, 1184, 1136, 818. HRMS (EI): *m/z* = [M]^•^^+^ calculated for C_21_H_19_N_5_OS_2_^•^^+^: 421.1026; found: 421.1024.

*N*-(6-(3-Acetamidophenyl)benzo[*d*]thiazol-2-yl)-2-((4,6-dimethylpyrimidin-2-yl)thio)acetamide (**FM94***).* At room temperature, acetic anhydride (22.3 µL, 0.237 mmol, 2.00 eq) was added dropwise to a stirred solution of amine **19** (50.0 mg, 0.119 mmol, 1.00 eq) and NEt_3_ (33.1 µL, 0.237 mmol, 2.00 eq) in anhydrous DCM (2 mL). After 3 h, water (50 mL) was added, and the reaction mixture was extracted with DCM (4 × 50 mL). The combined organic layers were dried over sodium sulfate. After evaporating the solvent in vacuo, the crude product was purified via flash column chromatography (DCM/MeOH 100:1) to yield the title compound as a white solid (53.0 mg, 0.114 mmol, 96%). m.p.: 162 °C (decomposition), R*_f_*: 0.03 (DCM/MeOH 100:1). ^1^H NMR (500 MHz, DMSO-d_6_) δ [ppm] = 12.68 (s, 1H, 2-NHCO), 10.03 (s, 1H, 3′-NHCO), 8.21 (d, *J* = 1.9 Hz, 1H, 7-H), 7.93 (t, *J* = 1.9 Hz, 1H, 2′-H), 7.83 (d, *J* = 8.4 Hz, 1H, 4-H), 7.66 (dd, *J* = 8.5, 1.9 Hz, 1H, 5-H), 7.56 (dt, *J* = 7.2, 2.1 Hz, 1H, 4′-H), 7.44–7.32 (m, 2H, 5′-H, 6′-H), 6.96 (s, 1H, 5″-H), 4.20 (s, 2H, CH_2_), 2.29 (s, 6H, 4″-CH_3_, 6″-CH_3_), 2.07 (s, 3H, CH_3_). ^1^^3^C NMR (126 MHz, DMSO-d_6_) δ [ppm] = 168.8 (C-2″), 168.4 (3′-NHCO), 168.2 (2-NHCO), 167.1 (C-4″, C-6″), 162.3 (C-2′), 158.4 (C-2), 148.2 (C-3a), 140.5 (C-1′), 139.9 (C-3′), 135.9 (C-6), 132.4 (C-7a), 129.3 (C-5′), 125.1 (C-5), 121.6 (C-6′), 120.8 (C-4), 119.7 (C-7), 117.9 (C-4′), 117.4 (C-2′), 116.2 (C-5″), 34.5 (CH_2_), 24.1 (CH_3_), 23.3 (4″-CH_3_, 6″-CH_3_). IR (ATR) ν῀ [cm^−1^] = 2922, 1694, 1605, 1583, 1537, 1456, 1428, 1314, 1263, 1146, 1033, 874, 824, 790, 696. HRMS (EI): *m/z* = [M]^•^^+^ calculated for C_23_H_21_N_5_O_2_S_2_^•^^+^: 463.1131; found: 463.1128. Purity (HPLC): >99%.

*N*-(3-(2-(2-((4,6-Dimethylpyrimidin-2-yl)thio)acetamido)benzo[*d*]thiazol-6-yl)phenyl)benzamide (**FM95***).* At room temperature, benzoyl chloride (26.6 µL, 0.229 mmol, 2.00 eq) was added dropwise to a stirred solution of amine **19** (48.3 mg, 0.115 mmol, 1.00 eq) and NEt_3_ (31.9 µL, 0.229 mmol, 2.00 eq) in anhydrous DCM (2 mL). After 3 h, water (50 mL) was added, and the reaction mixture was extracted with DCM (4 × 50 mL). The combined organic layers were dried over sodium sulfate. After evaporating the solvent in vacuo, the crude product was purified via flash column chromatography (DCM/MeOH 120:1) to yield the title compound as a white solid (39.7 mg, 0.0755 mmol, 66%). m.p.: 262 °C, R*_f_*: 0.17 (DCM/MeOH 120:1). ^1^H NMR (500 MHz, DMSO-d_6_) δ [ppm] = 12.70 (s, 1H, 2-NHCO), 10.34 (s, 1H, 3′-NHCO), 8.27 (d, *J* = 1.8 Hz, 1H, 7-H), 8.17 (t, *J* = 1.5 Hz, 1H, 2′-H), 8.02–7.97 (m, 2H, 2‴-H, 6‴-H), 7.86 (d, *J* = 8.4 Hz, 1H, 4-H), 7.85–7.79 (m, 1H, 4′-H), 7.73 (dd, *J* = 8.5, 1.9 Hz, 1H, 5-H), 7.63–7.59 (m, 1H, 4‴-H), 7.58–7.53 (m, 2H, 3‴-H, 5‴-H), 7.49–7.44 (m, 2H, 5′-H, 6′-H), 6.96 (s, 1H, 5″-H), 4.20 (s, 2H, CH_2_), 2.30 (s, 6H, 4″-CH_3_, 6″-CH_3_). ^1^^3^C NMR (126 MHz, DMSO-d_6_) δ [ppm] = 168.8 (C-2″), 168.2 (2-NHCO), 167.1 (C-4″, C-6″), 165.6 (3′-NHCO), 158.4 (C-2), 148.2 (C-3a), 140.4 (C-1′), 139.8 (C-3′), 135.8 (C-6), 134.9 (C-1‴), 132.5 (C-7a), 131.6 (C-4‴), 129.3 (C-5′), 128.4 (C-3‴, C-5‴), 127.7 (C-2‴, C-6‴), 125.1 (C-5), 122.1 (C-6′), 120.9 (C-4), 119.7 (C-7), 119.1 (C-4′), 118.7 (C-2′), 116.2 (C-5′), 34.5 (CH_2_), 23.3 (4″-CH_3_, 6″-CH_3_). IR (ATR) ν῀ [cm^−1^] = 3295, 2956, 1718, 1647, 1582, 1526, 1464, 1432, 1389, 1368, 1307, 1264, 1152, 884, 876, 884, 892, 762, 706, 694. HRMS (EI): *m*/*z* = [M]^•^^+^ calculated for C_28_H_23_N_5_O_2_S_2_^•^^+^: 525.1288; found: 525.1294. Purity (HPLC): >99%.

*N*-(3-(2-(2-((4,6-Dimethylpyrimidin-2-yl)thio)acetamido)benzo[*d*]thiazol-6-yl)phenyl)thiophene-2-carboxamide (**FM96***).* At room temperature, 2-thiophenecarbonyl chloride (15.6 µL, 0.145 mmol, 1.10 eq) was added dropwise to a stirred solution of amine **19** (55.7 mg, 0.132 mmol, 1.00 eq) and NEt_3_ (20.3 µL, 0.145 mmol, 1.10 eq) in anhydrous DCM (2 mL). After 1 h, water (50 mL) was added, and the reaction mixture was extracted with DCM (4 × 50 mL). The combined organic layers were dried over sodium sulfate. After evaporating the solvent in vacuo, the crude product was purified via flash column chromatography (hexanes/EtOAc 6:4) to yield the title compound as a white solid (57.0 mg, 0.107 mmol, 81%). m.p.: 226 °C, R*_f_*: 0.25 (hexanes/EtOAc 6:4). ^1^H NMR (400 MHz, DMSO-d_6_) δ [ppm] = 12.70 (s, 1H, 2-NHCO), 10.32 (s, 1H, 3′-NHCO), 8.27 (d, *J* = 1.9 Hz, 1H, 7-H), 8.10 (s, 1H, 2′-H), 7.93–7.81 (m, 2H, 4-H, 5‴-H), 7.81–7.68 (m, 2H, 5-H, 6′-H), 7.51–7.42 (m, 2H, 4′-H, 5′-H), 7.25 (dd, *J* = 5.0, 3.7 Hz, 1H, 4‴-H), 6.97 (s, 1H, 5″-H), 4.20 (s, 2H, CH_2_), 2.30 (s, 6H, 4″-CH_3_, 6″-CH_3_). ^1^^3^C NMR (126 MHz, DMSO-d_6_) δ [ppm] = 168.8 (2-NHCO), 168.2 (C-2‴), 167.1 (C-4″, C-6″), 156.0 (3′-NHCO), 158.5 (C-2), 148.3 (C-3a), 140.4 (C-1′), 140.0 (C-2‴), 139.3 (C-3′), 135.7 (C-6), 132.5 (C-7a), 132.0 (C-5‴), 129.4 (C-5′), 129.2 (C-3‴), 128.1 (C-4‴), 125.1 (C-5), 122.2 (C-4′), 120.9 (C-4), 119.7 (C-7), 119.1 (C-6′), 118.8 (C-2′), 116.2 (C-5″), 34.5 (CH_2_), 23.3 (4″-CH_3_, 6″-CH_3_). IR (ATR) ν῀ [cm^−1^] = 2964, 1704, 1657, 1605, 1583, 1544, 1491, 1454, 1313, 1266, 1212, 1152, 875, 837, 792, 732, 696. HRMS (EI): *m/z* = [M]^•^^+^ calculated for C_26_H_21_N_5_O_2_S_3_^•^^+^: 531.0852; found: 531.0843. Purity (HPLC): >99%.

2-((4,6-Dimethylpyrimidin-2-yl)thio)-*N*-(6-(3-(phenylsulfonamido)phenyl)benzo[*d*]thiazol-2-yl)acetamide (**FM104***).* At room temperature, benzenesulfonyl chloride (23.4 µL, 0.184 mmol, 0.95 eq) was added dropwise to a stirred solution of amine **19** (81.5 mg, 0.193 mmol, 1.00 eq) and NEt_3_ (25.6 µL, 0.184 mmol, 0.95 eq) in anhydrous DCM (5 mL). After 1 h, water (25 mL) and brine (25 mL) were added, and the reaction mixture was extracted with DCM (3 × 50 mL). The combined organic layers were dried over sodium sulfate. After evaporating the solvent in vacuo, the crude product was purified via flash column chromatography (DCM/MeOH 100:1) to yield the title compound as a white solid (16.3 mg, 0.0290 mmol, 15%). m.p.: 204–209 °C, R*_f_*: 0.06 (DCM/MeOH 100:1). ^1^H NMR (500 MHz, DMSO-d_6_) δ [ppm] = 12.69 (s, 1H, NHCO), 10.40 (s, 1H, NHSO_2_), 8.13 (d, *J* = 1.9 Hz, 1H, 7-H), 7.85–7.78 (m, 3H, 4-H, 2‴-H, 6‴-H), 7.63–7.59 (m, 1H, 4‴-H), 7.58–7.53 (m, 3H, 5-H, 3‴-H, 5‴-H), 7.41–7.35 (m, 2H, 2′-H, 6′-H), 7.32 (t, *J* = 7.8 Hz, 1H, 5′-H), 7.07 (ddd, *J* = 7.8, 2.2, 1.1 Hz, 1H, 4′-H), 6.96 (s, 1H, 5″-H), 4.19 (s, 2H, CH_2_), 2.29 (s, 6H, 4″-CH_3_, 6″-CH_3_). ^1^^3^C NMR (126 MHz, DMSO-d_6_) δ [ppm] = 168.8 (C-2″), 168.2 (NHCO), 167.1 (C-4″, C-6″), 158.6 (C-2), 148.3 (C-3a), 141.0 (C-1′), 139.4 (C-1‴), 138.3 (C-3′), 135.2 (C-6), 133.0 (C-4‴), 132.5 (C-7a), 129.8 (C-5′), 129.3 (C-3‴, C-5‴), 126.7 (C-2‴, C-6‴), 125.0 (C-5), 122.7 (C-6′), 120.9 (C-4), 119.7 (C-7), 118.8 (C-4′), 118.4 (C-2′), 116.2 (C-5″), 34.5 (CH_2_), 23.3 (4″-CH_3_, 6″-CH_3_). IR (ATR) ν῀ [cm^−1^] = 3186, 2965, 1720, 1602, 1580, 1454, 1331, 1309, 1265, 1154, 1088, 955, 882, 836, 794, 751, 714, 685. HRMS (EI): *m/z* = [M]^•^^+^ calculated for C_27_H_23_N_5_O_3_S_3_^•^^+^: 561.0958; found: 561.0956. Purity (HPLC): >99%.

*N*-(3-(2-(2-((4,6-Dimethylpyrimidin-2-yl)thio)acetamido)benzo[*d*]thiazol-6-yl)phenyl)-5-methylthiophene-2-carboxamide (**FM108***).* At room temperature, previously prepared 5-methylthiophene-2-carbonyl chloride (26.8 µL, 0.220 mmol, 2.00 eq) was added dropwise to a stirred solution of amine **19** (46.4 mg, 0.110 mmol, 1.00 eq) and NEt_3_ (30.7 µL, 0.220 mmol, 2.00 eq) in anhydrous DCM (5 mL). After 2 h, water (50 mL) was added, and the reaction mixture was extracted with DCM (3 × 50 mL). The combined organic layers were dried over sodium sulfate. After evaporating the solvent in vacuo, the crude product was purified via flash column chromatography (DCM/MeOH 100:2) to yield the title compound as a white solid (27.6 mg, 0.0506 mmol, 46%). m.p.: 122 °C (decomposition), R*_f_*: 0.04 (DCM/MeOH 100:2). ^1^H NMR (500 MHz, DMSO-d_6_) δ [ppm] = 12.69 (s, 1H, 2-NHCO), 10.19 (s, 1H, 3′-NHCO), 8.26 (d, *J* = 1.9 Hz, 1H, 7-H), 8.09 (t, *J* = 1.3 Hz, 1H, 2′-H), 7.89–7.83 (m, 2H, 4-H, 3‴-H), 7.75–7.70 (m, 2H, 5-H, 4′-H), 7.48–7.40 (m, 2H, 5′-H, 6′-H), 6.97 (s, 1H, 5″-H), 6.94 (dd, *J* = 3.7, 1.1 Hz, 1H, 4‴-H), 4.20 (s, 2H, CH_2_), 2.51 (s, 3H, CH_3_), 2.30 (s, 6H, 4″-CH_3_, 6″-CH_3_). ^1^^3^C NMR (126 MHz, DMSO-d_6_) δ [ppm] = 168.8 (C-2″), 168.2 (2-NHCO), 167.1 (C-4″, C-6″), 159.9 (3′-NHCO), 158.4 (C-2), 148.2 (C-3a), 146.0 (C-5‴), 140.4 (C-1′), 139.5 (C-3′), 137.4 (C-2‴), 135.7 (C-6), 132.5 (C-7a), 129.4 (C-3‴), 129.3 (C-5′), 126.7 (C-4‴), 125.1 (C-5), 122.1 (C-6′), 120.9 (C-4), 119.7 (C-7), 119.0 (C-4′), 118.6 (C-2′), 116.2 (C-5″), 34.5 (CH_2_), 23.3 (4″-CH_3_, 6″-CH_3_), 15.3 (CH_3_). IR (ATR) ν῀ [cm^−1^] = 3268, 2956, 1720, 1626, 1581, 1544, 1525, 1486, 1455, 1422, 1307, 1264, 1212, 1152, 1095, 978, 881, 852, 834, 816, 790, 756, 742, 698, 664. HRMS (EI): *m/z* = [M]^•^^+^ calculated for C_3__0_H_26_N_4_O_2_S_2_^•^^+^: 545.1008; found: 545.1005. Purity (HPLC): >99%.

*N*-(4-(2-(2-((4,6-Dimethylpyrimidin-2-yl)thio)acetamido)benzo[*d*]thiazol-6-yl)phenyl)benzamide (**FM127***).* At room temperature, benzoyl chloride (43.0 µL, 0.371 mmol, 2.00 eq) was added dropwise to a stirred solution of amine **20** (78.1 mg, 0.185 mmol, 1.00 eq) and NEt_3_ (51.6 µL, 0.371 mmol, 2.00 eq) in anhydrous DCM (5 mL). After 3 h, water (50 mL) was added, and the reaction mixture was extracted with DCM (4 × 50 mL). The combined organic layers were dried over sodium sulfate. After evaporating the solvent in vacuo, the crude product was purified via flash column chromatography (hexanes/EtOAc 1:1) to yield the title compound as a white solid (44.3 mg, 0.0843 mmol, 46%). m.p.: 262 °C, R*_f_*: 0.14 (hexanes/EtOAc 1:1). ^1^H NMR (400 MHz, DMSO-d_6_) δ [ppm] = 12.67 (s, 1H, 2-NHCO), 10.35 (s, 1H, 4′-NHCO), 8.30 (d, *J* = 1.8 Hz, 1H, 7-H), 8.02–7.94 (m, 2H, 2‴-H, 6‴-H), 7.94–7.88 (m, 2H, 3′-H, 5′-H), 7.82 (d, *J* = 8.5 Hz, 1H, 4-H), 7.78–7.71 (m, 3H, 5-H, 2′-H, 6′-H), 7.64–7.52 (m, 3H, 3‴-H, 4‴-H, 5‴-H), 6.97 (s, 1H, 5″-H), 4.20 (s, 2H, CH_2_), 2.30 (s, 6H, 4″-CH_3_, 6″-CH_3_). ^1^^3^C NMR (101 MHz, DMSO-d_6_) δ [ppm] = 168.9 (C-2″), 168.2 (2-NHCO), 167.1 (C-4″, C-6″), 165.6 (4′-NHCO), 158.2 (C-2), 147.9 (C-3a), 138.6 (C-4′), 135.4 (C-6), 135.0 (C-1‴), 134.9 (C-1′), 132.5 (C-7a), 131.6 (C-4‴), 128.4 (C-3‴, C-5‴), 127.7 (C-2‴, C-6‴), 126.9 (C-5), 124.8 (C-4), 120.7 (C-3′, C-5′), 119.2 (C-7), 116.2 (C-5″), 34.5 (CH_2_), 23.3 (4″-CH_3_, 6″-CH_3_). IR (ATR) ν῀ [cm^−1^] = 3367, 1684, 1673, 1604, 1566, 1550, 1527, 1493, 1455, 1394, 1342, 1324, 1298, 1267, 1245, 1227, 1189, 1129, 1031, 897, 870, 834, 808, 750, 702, 690, 676. HRMS (EI): *m/z* = [M]^•^^+^ calculated for C_28_H_23_N_5_O_2_S_2_^•^^+^: 525.1288; found: 525.1281. Purity (HPLC): >99%.

*N*-(6-(4-Acetamidophenyl)benzo[*d*]thiazol-2-yl)-2-((4,6-dimethylpyrimidin-2-yl)thio)acetamide (**FM128***).* At room temperature, acetic anhydride (23.7 µL, 0.253 mmol, 2.00 eq) was added dropwise to a stirred solution of amine **20** (53.3 mg, 0.126 mmol, 1.00 eq) and NEt_3_ (35.2 µL, 0.253 mmol, 2.00 eq) in anhydrous DCM (2 mL). After 3 h, water (50 mL) was added, and the reaction mixture was extracted with DCM (4 × 50 mL). The combined organic layers were dried over sodium sulfate. After evaporating the solvent in vacuo, the crude product was purified via flash column chromatography (hexanes/EtOAc 4:6) to yield the title compound as a white solid (35.1 mg, 0.0757 mmol, 60%). m.p.: 255 °C, R*_f_*: 0.06 (hexanes/EtOAc 4:6). ^1^H NMR (500 MHz, DMSO-d_6_) δ [ppm] = 12.65 (s, 1H, 2-NHCO), 10.02 (s, 1H, 4′-NHCO), 8.24 (d, *J* = 1.8 Hz, 1H, 7-H), 7.79 (d, *J* = 8.4 Hz, 1H, 4-H), 7.71 (dd, *J* = 8.5, 1.9 Hz, 1H, 5-H), 7.69–7.64 (m, 4H, 2′-H, 3′-H, 5′-H, 6′-H), 6.96 (s, 1H, 5″-H), 4.19 (s, 2H, CH_2_), 2.29 (s, 6H, 3″-CH_3_, 4″-CH_3_), 2.07 (s, 3H, CH_3_). ^1^^3^C NMR (126 MHz, DMSO-d_6_) δ [ppm] = 168.8 (C-2″), 168.3 (4′-NHCO), 168.1 (2-NHCO), 167.1 (C-4″, C-6″), 158.1 (C-2), 147.8 (C-3a), 138.7 (C-4′), 135.5 (C-6), 134.4 (C-1′), 132.4 (C-7a), 127.0 (C-2′, C-6′), 124.8 (C-5), 120.8 (C-4), 119.3 (C-3′, C-5′), 119.1 (C-7), 116.2 (C-5″), 34.4 (CH_2_), 24.1 (CH_3_), 23.3 (3″-CH_3_, 4″-CH_3_). IR (ATR) ν῀ [cm^−1^] = 3361, 1679, 1658, 1600, 1551, 1531, 1460, 1394, 1340, 1330, 1292, 1275, 1264, 1225, 1192, 1128, 1092, 830, 807, 799, 748, 734, 692. HRMS (EI): *m/z* = [M]^•^^+^ calculated for C_23_H_21_N_5_O_2_S_2_^•^^+^: 463.1131; found: 463.1132. Purity (HPLC): >99%.

*N*-(4-(2-(2-((4,6-Dimethylpyrimidin-2-yl)thio)acetamido)benzo[*d*]thiazol-6-yl)phenyl)thiophene-2-carboxamide (**FM129***).* At room temperature, 2-thiophenecarbonyl chloride (23.8 µL, 0.222 mmol, 1.20 eq) was added dropwise to a stirred solution of amine **20** (78.1 mg, 0.185 mmol, 1.00 eq) and NEt_3_ (23.8 µL, 0.222 mmol, 1.20 eq) in anhydrous DCM (5 mL). After 1 h, water (50 mL) was added, and the reaction mixture was extracted with DCM (4 × 50 mL). The combined organic layers were dried over sodium sulfate. After evaporating the solvent in vacuo, the crude product was purified via flash column chromatography (DCM/MeOH 100:1) to yield the title compound as a white solid (75.7 mg, 0.142 mmol, 77%). m.p.: 280–284 °C, R*_f_*: 0.29 (DCM/MeOH 100:1). ^1^H NMR (400 MHz, DMSO-d_6_) δ [ppm] = 12.68 (s, 1H, 2-NHCO), 10.33 (s, 1H, 4′-NHCO), 8.30 (d, *J* = 1.8 Hz, 1H, 7-H), 8.05 (dd, *J* = 3.8, 1.2 Hz, 1H, 3‴-H), 7.88 (dd, *J* = 5.0, 1.1 Hz, 1H, 5‴-H), 7.86–7.79 (m, 3H, 4-H, 3′-H, 5′-H), 7.79–7.72 (m, 3H, 5-H, 2′-H, 6′-H), 7.24 (dd, *J* = 5.0, 3.8 Hz, 1H, 4‴-H), 6.97 (s, 1H, 5″-H), 4.20 (s, 2H, CH_2_), 2.29 (s, 6H, 4″-CH_3_, 6″-CH_3_). ^1^^3^C NMR (101 MHz, DMSO-d_6_) δ [ppm] = 168.8 (C-2″), 168.2 (2-NHCO), 167.0 (C-4″, C-6″), 159.9 (4′-NHCO), 158.2 (C-2), 147.9 (C-3a), 140.0 (C-2‴), 138.1 (C-4′), 135.3 (C-6), 135.1 (C-1′), 132.5 (C-7a), 132.0 (C-5‴), 129.2 (C-3‴), 128.1 (C-4‴), 127.0 (C-2′, C-6′), 124.8 (C-5), 120.8 (C-4), 120.7 (C-3′, C-5′), 119.2 (C-7), 116.2 (C-5″), 34.5 (CH_2_), 23.3 (4″-CH_3_, 6″-CH_3_). IR (ATR) ν῀ [cm^−1^] = 3371, 1682, 1659, 1600, 1531, 1457, 1421, 1326, 1263, 1228, 1188, 1091, 864, 832, 805, 750, 722. HRMS (EI): *m*/*z* = [M]^•^^+^ calculated for C_26_H_21_N_5_O_2_S_3_^•^^+^: 531.0852; found: 531.0860. Purity (HPLC): >99%.

2-((4,6-Dimethylpyrimidin-2-yl)thio)-*N*-(6-(3-(phenylsulfonamido)phenyl)benzo[*d*]thiazol-2-yl)acetamide (**FM130***).* At room temperature, benzenesulfonyl chloride (26.1 µL, 0.204 mmol, 0.95 eq) was added dropwise to a stirred solution of amine **20** (90.6 mg, 0.215 mmol, 1.00 eq) and NEt_3_ (28.5 µL, 0.204 mmol, 0.95 eq) in anhydrous DCM (5 mL). After heating to reflux for 8 h, water (25 mL) and brine (25 mL) were added, and the reaction mixture was extracted with DCM (3 × 50 mL). The combined organic layers were dried over sodium sulfate. After evaporating the solvent in vacuo, the crude product was purified via flash column chromatography (DCM/MeOH/AcOH 100:0.5:0.25) to yield the title compound as a white solid (68.0 mg, 0.121 mmol, 59%). m.p.: 233 °C, R*_f_*: 0.07 (DCM/MeOH/AcOH 100:0.5:0.25). ^1^H NMR (500 MHz, CDCl_3_) δ [ppm] = 11.86 (s, 1H, NHCO), 7.91 (d, *J* = 1.8 Hz, 1H, 7-H), 7.84–7.78 (m, 2H, 2‴-H, 6‴-H), 7.74 (d, *J* = 8.4 Hz, 1H, 4-H), 7.57–7.52 (m, 2H, 5-H, 4‴-H), 7.52–7.48 (m, 2H, 2′-H, 6′-H), 7.48–7.43 (m, 2H, 3‴-H, 5‴-H), 7.18–7.12 (m, 2H, 3′-H, 5′-H), 6.88 (s, 1H, 5″-H), 6.78 (s, 1H, NHSO_2_), 3.98 (s, 2H, CH_2_), 2.57 (s, 6H, 4″-CH_3_, 6″-CH_3_). ^1^^3^C NMR (126 MHz, CDCl_3_) δ [ppm] = 169.9 (C-2″), 168.4 (NHCO), 168.3 (C-4″, C-6″), 158.0 (C-2), 148.4 (C-3a), 139.2 (C-1‴), 138.2 (C-1′), 136.2 (C-6), 135.6 (C-4′), 133.4 (C-7a), 133.2 (C-4‴), 129.2 (C-3‴, C-5‴), 128.2 (C-2′, C-6′), 127.4 (C-2‴, C-6‴), 125.5 (C-5), 122.4 (C-3′, C-5′), 121.3 (C-4), 119.6 (C-7), 117.3 (C-5″), 34.8 (CH_2_), 24.0 (4″-CH_3_, 6″-CH_3_). IR (ATR) ν῀ [cm^−1^] = 3260, 2918, 1695, 1604, 1583, 1560, 1540, 1517, 1464, 1407, 1332, 1312, 1295, 1274, 1227, 1151, 1090, 1031, 929, 847, 824, 754, 718, 687. HRMS (EI): *m/z* = [M]^•^^+^ calculated for C_27_H_23_N_5_O_3_S_3_^•^^+^: 561.0958; found: 561.0954. Purity (HPLC): >99%.

*N*-(4-(2-(2-((4,6-Dimethylpyrimidin-2-yl)thio)acetamido)benzo[*d*]thiazol-6-yl)phenyl)-5-methylthiophene-2-carboxamide (**FM131**). At room temperature, previously prepared 5-methylthiophene-2-carbonyl chloride (25.2 µL, 0.202 mmol, 1.10 eq) was added dropwise to a stirred solution of amine **20** (77.4 mg, 0.184 mmol, 1.10 eq) and NEt_3_ (28.2 µL, 0.202 mmol, 1.10 eq) in anhydrous DCM (5 mL). After 2 h, water (50 mL) was added, and the reaction mixture was extracted with DCM (3 × 50 mL). The combined organic layers were dried over sodium sulfate. After evaporating the solvent in vacuo, the crude product was purified via flash column chromatography (hexanes/EtOAc 6:4) to yield the title compound as a white solid (45.3 mg, 0.083 mmol, 45%). m.p.: 294 °C, R*_f_*: 0.10 (hexanes/EtOAc 6:4). ^1^H NMR (400 MHz, DMSO-d_6_) δ [ppm] = 12.66 (s, 1H, 2-NHCO), 10.19 (s, 1H, 4′-NHCO), 8.29 (d, *J* = 1.8 Hz, 1H, 7-H), 7.87–7.78 (m, 4H, 4-H, 3′-H, 5′-H, 3‴-H), 7.78–7.70 (m, 3H, 2′-H, 6′-H, 5-H), 6.97 (s, 1H, 5″-H), 6.94 (dd, *J* = 3.7, 1.2 Hz, 1H, 4‴-H), 4.20 (s, 2H, CH_2_), 2.30 (s, 6H, 4″-CH_3_, 6″-CH_3_). ^1^^3^C NMR (126 MHz, DMSO-d_6_) δ [ppm] = 168.8 (C-2″), 168.1 (2-NHCO), 167.1 (C-4″, C-6″), 159.8 (4′-NHCO), 158.1 (C-2), 147.9 (C-3a), 145.9 (C-5‴), 138.2 (C-4′), 137.4 (C-2‴), 135.4 (C-6), 134.9 (C-1′), 132.5 (C-7a), 129.4 (C-3‴), 126.9 (C-2′, C-6′), 126.7 (C-4‴), 124.8 (C-5), 120.8 (C-4), 120.5 (C-3′, C-5′), 119.2 (C-7), 116.2 (C-5″), 34.4 (CH_2_), 23.3 (4″-CH_3_, 6″-CH_3_), 15.3 (CH_3_). IR (ATR) ν῀ [cm^−1^] = 3361, 1679, 1658, 1600, 1551, 1531, 1460, 1394, 1340, 1330, 1292, 1275, 1264, 1255, 1192, 1128, 1092, 899, 863, 830, 807, 799, 748, 734, 692. HRMS (EI): *m*/*z* = [M]^•^^+^ calculated for C_3__0_H_26_N_4_O_2_S_2_^•^^+^: 545.1008; found: 545.1008. Purity (HPLC): >99%.

### 4.2. Computational Methods

Docking simulations were conducted using the Schrödinger software suite (version 2020-3, Schrödinger Inc., New York City, NY, USA)[27]. Crystal structures of Sirt2 and lead compounds were obtained from the Protein Data Bank (PDB) [28] (**SirReal2**: PDB ID: 4RMG [14]; **24a**: PDB ID: 5YQO [29]) and prepared using the Protein Preparation Wizard (Schrödinger Inc., New York City, NY, USA). Ligand preparation was performed through Ligprep (Schrödinger Inc.), and Epik served for respective charge calculations and protonation states [30]. Docking simulations were executed using Glide in standard precision (SP) mode. The docking grid center was x = −13.495516, y = −10.113182, z = −18.406236, the INNERBOX size 10 and OUTERBOX 30.6493 for x, y, and z dimensions. Five poses per ligand were stored, and ligands were treated as flexible with 10 post-docking minimizations. All other docking parameters were left at their default settings. The ligand L5C of the X-ray structure 5YQO was redocked into 5YQO using the described parameters. A very low rmsd value of 0.89 was obtained, indicating the excellent applicability for the calculation of Sirt2 ligands. Pymol 2.5.8 (Schrödinger Inc.) was used for visual depiction. The poses with the best docking scores were examined, taking additionally into account the position of the pyrimidine ring similar to the crystal structures of the corresponding lead compounds.

### 4.3. Biological Investigations

The inhibitory activity of the synthesized target compounds was determined by Reaction Biology (Malvern, PA, USA) using an internal fluorescence-based assay protocol. All respective Sirt enzymes used in the assays originated from in-house sources at Reaction Biology Corporation (Malvern, USA). Sirt1: accession number: NM_012238; includes amino acids: 1–747 (C-term.); tag: N-terminal His-tag; expression system: *E. coli*; purity: >85% by SDS-PAGE; supplied as solution of purified recombinant protein in 50 mM Tris/HCl pH 7.5, 100 mM NaCl, 10% glycerol (*v*/*v*). Sirt2: accession number: NM_012237; includes amino acids: 50–389 (C-term.); tag: N-terminal His-tag; expression system: E. coli; purity: >90% by SDS-PAGE; supplied as solution of purified recombinant protein in 50 mM Tris/HCl pH 7.5, 500 mM NaCl, 1 mM TCEP, 10% glycerol (*v*/*v*). Sirt3: accession number: NM_012239.3; includes amino acids: 101–399; tag: N-terminal GST-tag; expression system: *E. coli*; purity: >85% by SDS-PAGE; supplied as solution of purified recombinant protein in 50 mM Tris/HCl pH 7.5, 500 mM NaCl, 10% glycerol (*v*/*v*). Sirt5: accession number: NM_012241; includes amino acids: 37–310 (C-term.); tag: N-terminal His-tag; expression system: *E. coli*; purity: >95% by SDS-PAGE; supplied as solution of purified recombinant protein in 50 mM Tris/HCl pH 7.5, 500 mM NaCl, 1 mM TCEP, 10% glycerol (*v*/*v*). Briefly, the test compounds (prepared in DMSO) were incubated with the respective sirtuin enzyme in reaction buffer (Tris-HCl, pH = 8) for 10 min at 30 °C. The deacetylation reaction was then initiated by adding the substrate mixture (cofactor NAD^+^ and 7-amino-4-methyl coumarin-based fluorogenic p53 residues 379–382 peptide substrate). The deacetylation reaction was stopped after 2 h of incubation at 30 °C by the addition of the universal inhibitor nicotinamide (2 mM) as well as the protease-based developer, cleaving of 7-amino-4-methyl coumarin to generate the characteristic fluorescence, which was measured 1 h later at 30 °C (extinction/emission = 360 nm/460 nm). For standardization purposes, a no-inhibitor control is included (corresponding to 100% enzyme activity). The IC_50_ values with Sirt2 were determined in triplicates, using a 10-dose 3-fold serial dilution starting at 50 µM (final reaction concentration), and 100 µM (final reaction concentration) was used if necessary. From the respective replicates of a dilution series of the triplicates, 3 individual IC_50_ values were determined based on sigmoidal curve fitting using Prism 8.0.2 software (GraphPad Software, Boston, MA, USA), which were then averaged to provide a mean IC_50_ value with the corresponding standard deviation. For the determination of subtype selectivity against Sirt1, Sirt3, and Sirt5, a single-dose duplicate mode at 50 µM final test compound concentration was used, and the inhibitory activity in percentage was presented as the mean of the duplicate values, including standard deviation, relative to a no-inhibitor control (corresponding to 100% activity).

## Figures and Tables

**Table 1:** Sirtuin-inhibitory activities of the rigidized analogues of **SirReal2** and compound **28e**: IC_50_ values shown represent the mean of 3 individual IC_50_ values obtained from serially diluted replicates of triplicates. The corresponding standard deviation (SD) is derived from the 3 determined IC_50_ values. In terms of subtype selectivity, the residual enzyme activity of Sirt1, Sirt3, and Sirt5 was determined at 50 µM inhibitor concentration in duplicate, and the inhibition (%) was calculated and presented as mean value including standard deviation thereof. Abbreviation: n.d. (not determined).

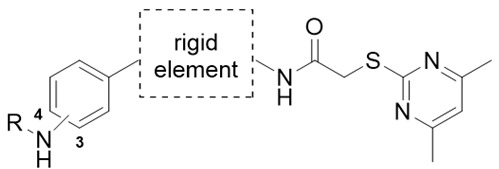							
ID	Position	R	Rigid Element	IC_50_ [µM] ± SD Sirt2	Subtype Selectivity Enzyme Inhibition [%] at 50 µM ± SD		
					Sirt1	Sirt3	Sirt5
**FM48**	3	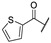	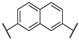	7.4 ± 2.4	6.1 ± 0.4	2.9 ± 1.2	15 ± 1
**FM50**	4			0.37 ± 0.04	7.4 ± 0.9	1.1 ± 0.1	3.3 ± 1.2
**FM96**	3	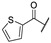	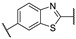	>100	n.d.	n.d.	n.d.
**FM129**	4			1.2 ± 0.4	1.4 ± 4.1	15 ± 1	16 ± 1
**FM66**	3	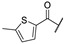	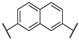	>100	n.d.	n.d.	n.d.
**FM69**	4			0.15 ± 0.01	−6.4 ± 1.9	−26 ± 4	−2.6 ± 0.3
**FM108**	3	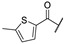	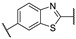	79 ± 23	6.2 ± 0.8	8.4 ± 0.2	14 ± 1
**FM131**	4			0.61 ± 0.03	6.8 ± 0.1	−8.6 ± 0.8	7.8 ± 0.7
**FM54**	3	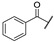	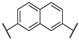	5.9 ± 1.6	0.60 ± 0.12	2.9 ± 1.3	11 ± 5
**FM53**	4			0.22 ± 0.03	−2.0 ± 2.5	−1.9 ± 1.5	−1.0 ± 1.4
**FM95**	3	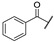	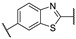	>100	n.d.	n.d.	n.d.
**FM127**	4			0.42 ± 0.03	4.8 ± 1.0	3.0 ± 0.1	13 ± 2
**FM47**	3	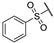	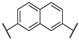	1.3 ± 0.2	0.0 ± 0.9	0.40 ± 0.38	16 ± 0
**FM56**	4			10 ± 1	3.5 ± 0.4	3.8 ± 1.3	12 ± 1
**FM104**	3	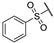	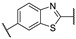	14 ± 4	14 ± 1	18 ± 1	22 ± 1
**FM130**	4			12 ± 2	16 ± 2	20 ± 1	22 ± 1
**FM46**	3	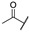	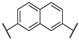	2.0 ± 0.3	0.40 ± 1.54	12 ± 1	1.0 ± 0.3
**FM26**	4			1.2 ± 0.2	20 ± 1	6.4 ± 2.1	11 ± 3
**FM94**	3	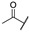	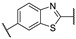	4.6 ± 1.2	16 ± 1	18 ± 2	15 ± 1
**FM128**	4			6.2 ± 1.3	14 ± 1	32 ± 1	14 ± 1
**28e**				0.087 ± 0.009	n.d.	n.d.	n.d.
**SirReal2**				0.24 ± 0.01	n.d.	n.d.	n.d.

## Data Availability

The entirety of the experimental data and protocols are stored in the university’s electronic lab journal at LMU Munich.

## Supplementary Materials

The following supporting information can be downloaded at https://www.mdpi.com/article/10.3390/molecules30081728/s1: ^1^H and ^13^C NMR spectra of synthesized compounds and HPLC chromatograms of final test substances.

## Abbreviations

The following abbreviations are used in this manuscript:

AcOH	Acetic acid
DCM	Dichloromethane
DMSO	Dimethylsulfoxide
EtOAc	Ethyl acetate
MeOH	Methanol
SAR	Structure–activity relationship
Sirt	Sirtuin
*t*-BuOK	Potassium tert-butoxide
