# Supplementary material for: Lead-Structure-Based Rigidization Approach to Optimize SirReal-Type Sirt2 Inhibitors
Source: Molecules. 2025 Apr 11;30(8):1728. doi: 10.3390/molecules30081728 (PMC12029821; doi:10.3390/molecules30081728)
Supplement: Supplementary file 1 [file molecules-30-01728-s001.zip › molecules-3547002-supplementary.pdf]

SUPPORTING INFORMATION

Lead structure-based rigidization approach to optimize SirReal-type Sirt2 inhibitors

Matthias Frei, Thomas Wein and Franz Bracher*

Department of Pharmacy – Center for Drug Research, Ludwig-Maximilians University, Butenandtstr. 5-13, 81377 Munich, Germany

Content:

NMR data of synthesized compounds

HPLC purity data of final test compounds

^{1}H and ^{13}C NMR spectra of 7-(4-Nitrophenyl)naphthalen-2-amine (**3**)

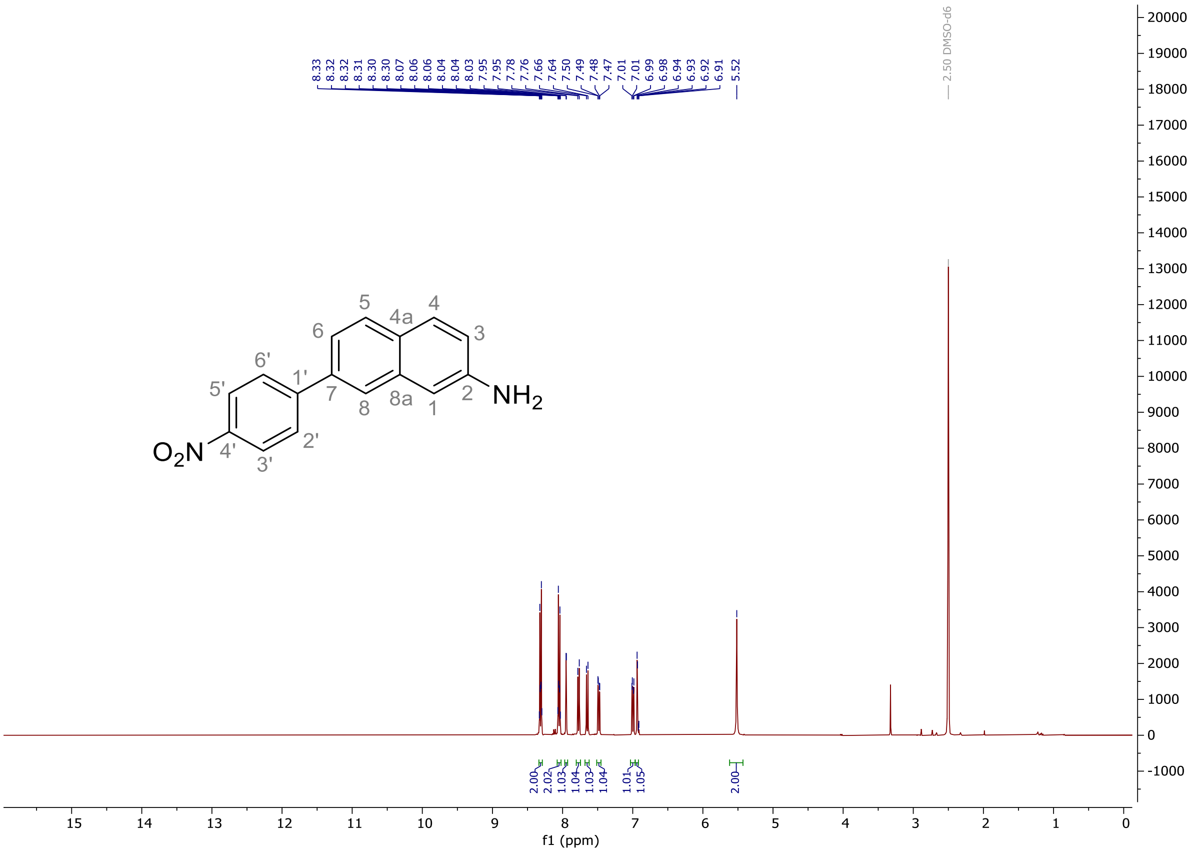

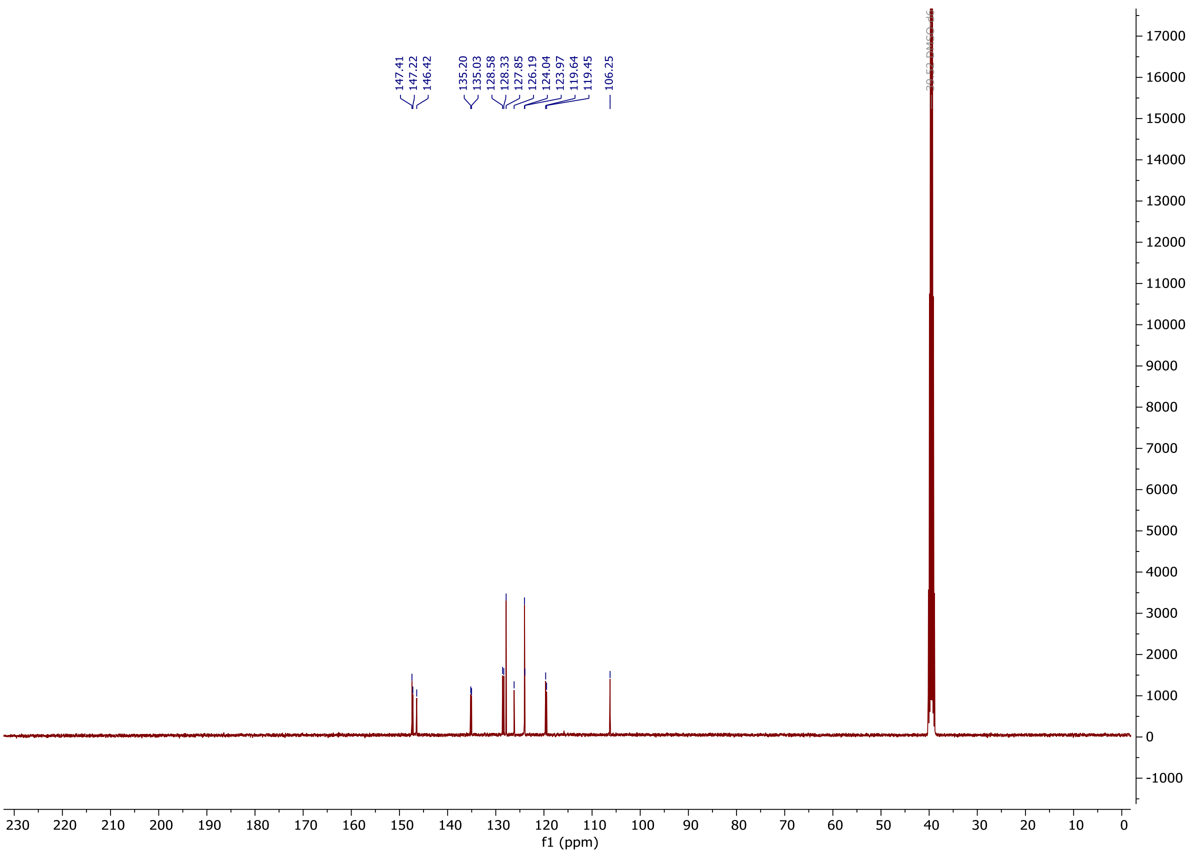

^{1}H and ^{13}C NMR spectra of 7-(3-Nitrophenyl)naphthalen-2-amine (**4**)

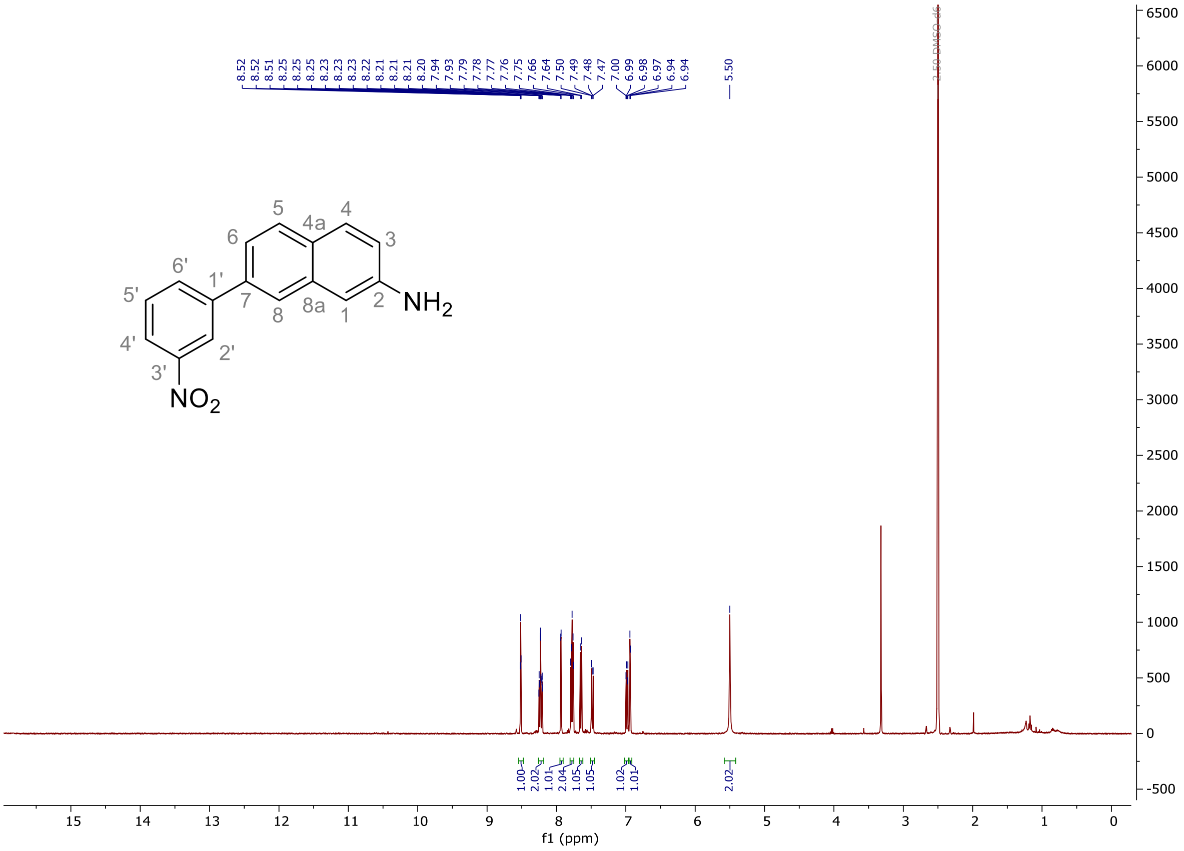

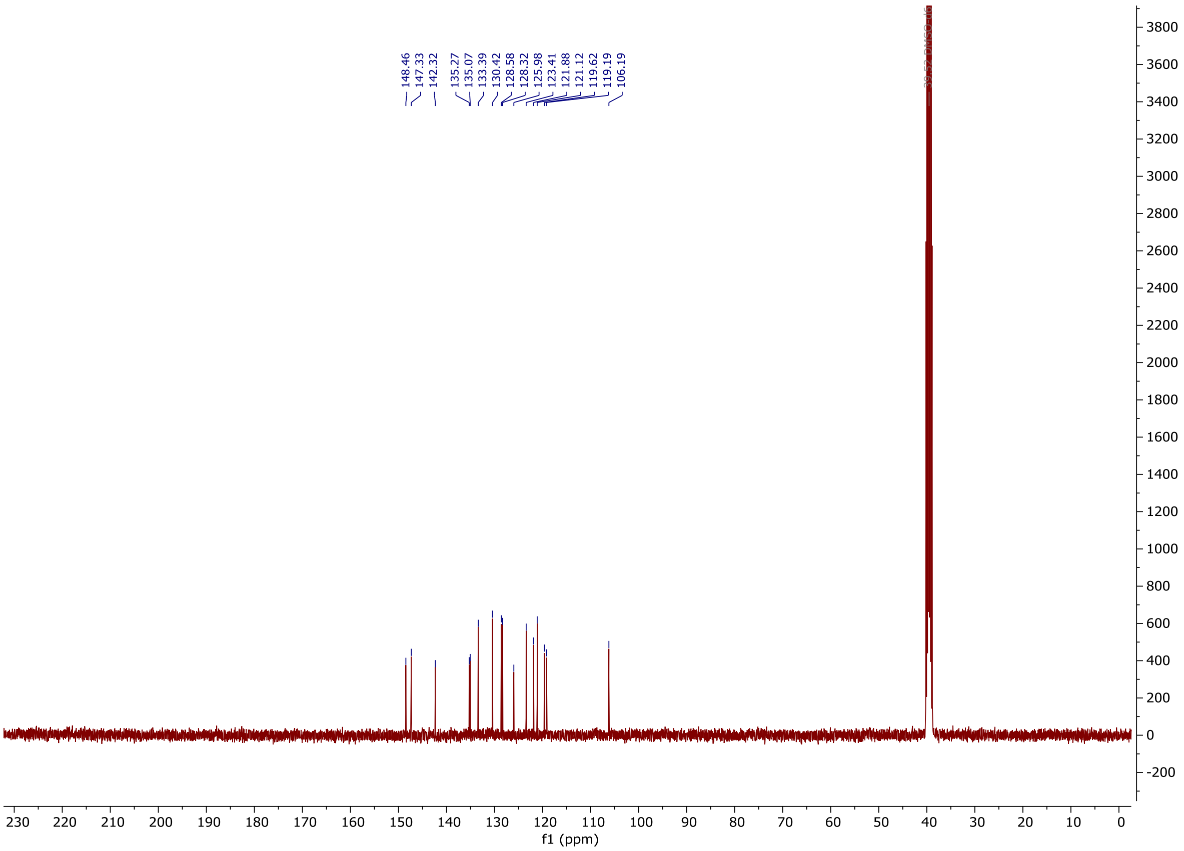

^{1}H and ^{13}C NMR spectra of 2-Bromo-*N*-(7-(4-nitrophenyl)naphthalen-2-yl)acetamide (**5**).

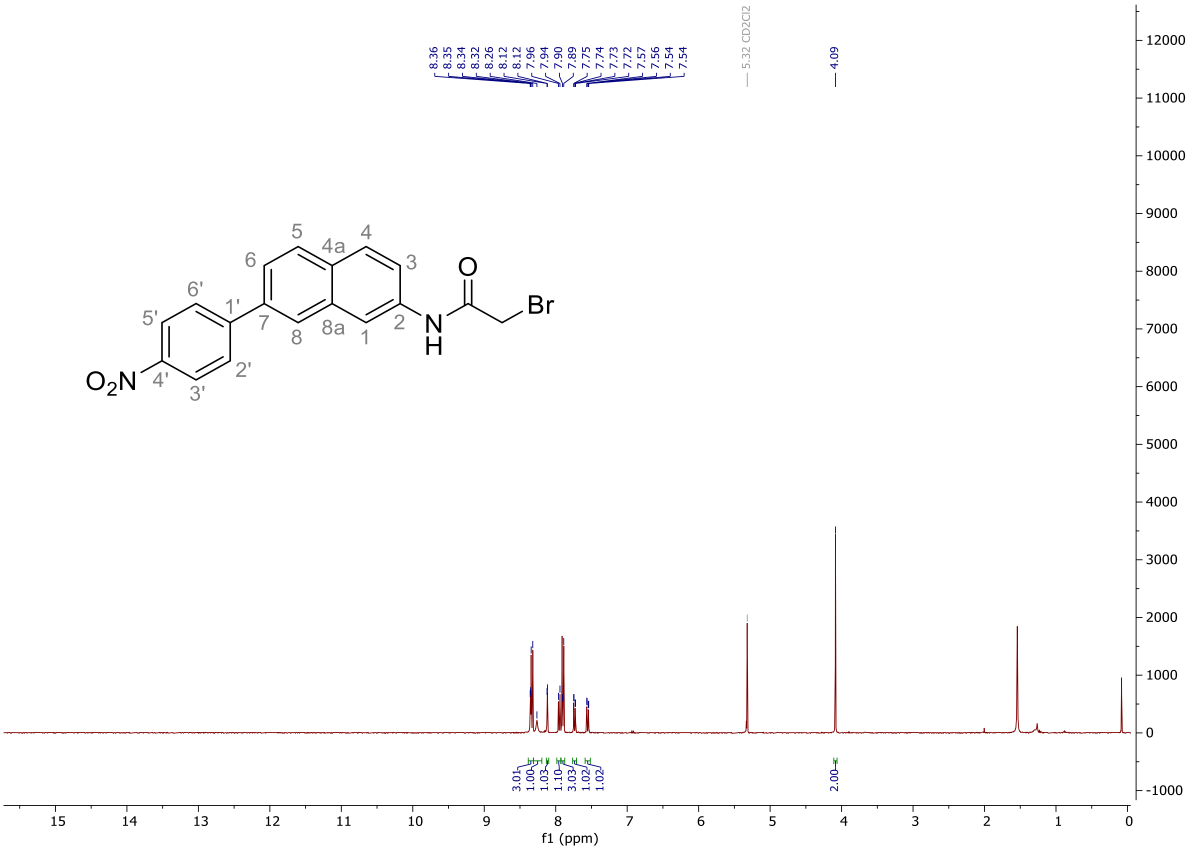

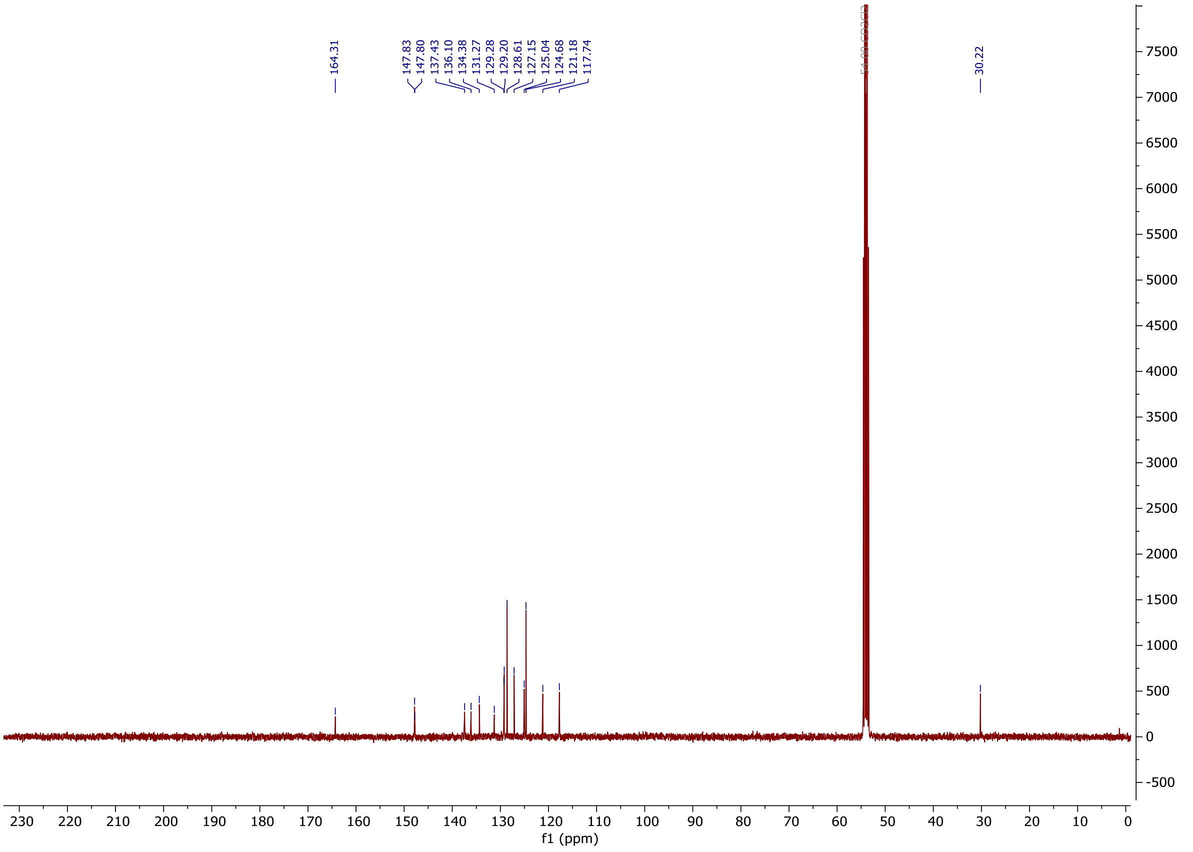

^{1}H and ^{13}C NMR spectra of 2-Bromo-*N*-(7-(3-nitrophenyl)naphthalen-2-yl)acetamide (**6**)

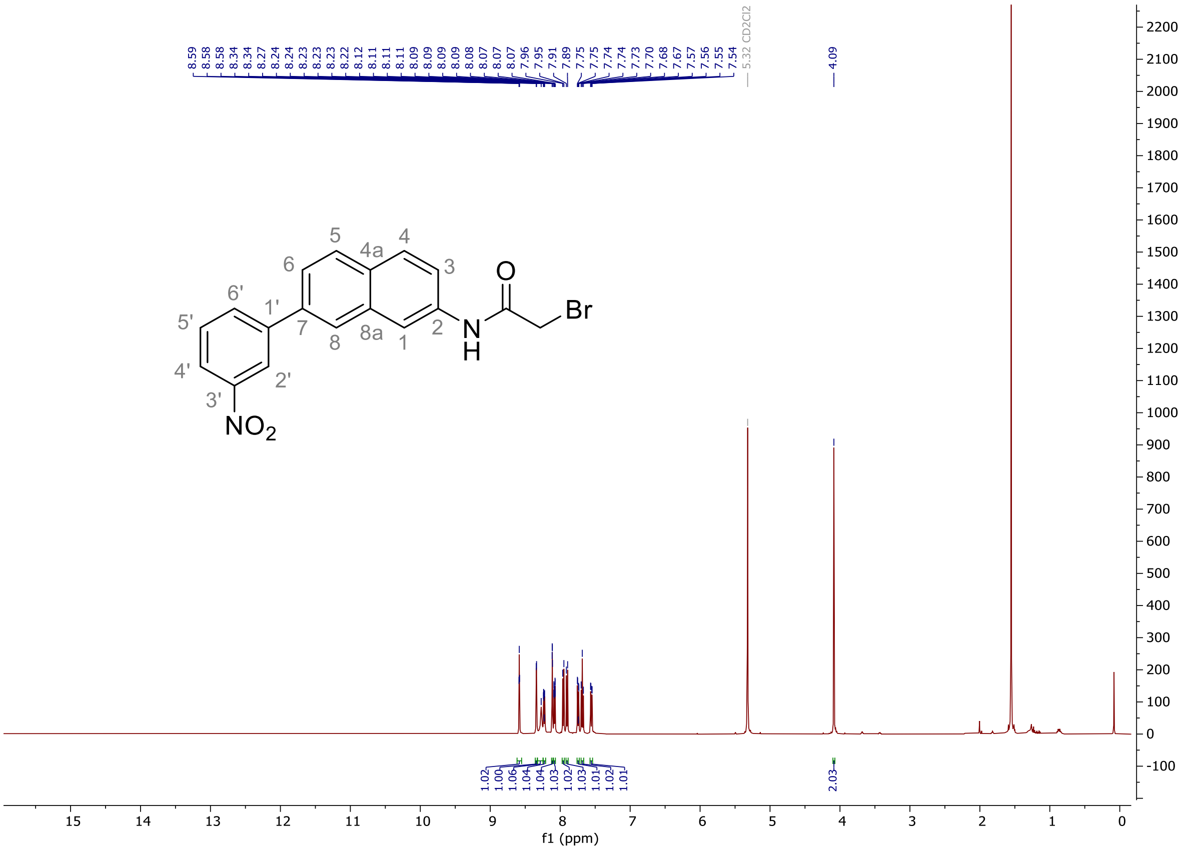

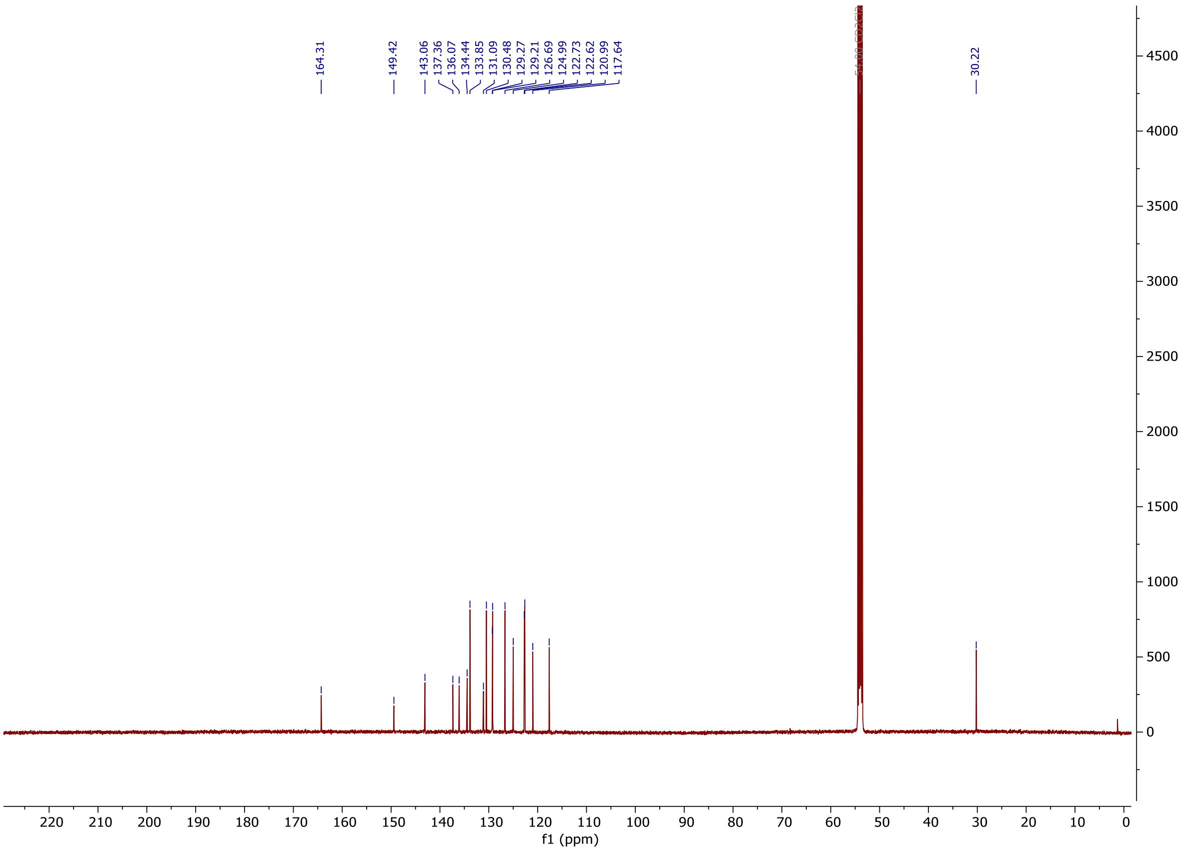

^{1}H and ^{13}C NMR spectra of

2-((4,6-Dimethylpyrimidin-2-yl)thio)-*N*-(7-(4 nitrophenyl)naphthalen-2-yl)acetamide (**7**)

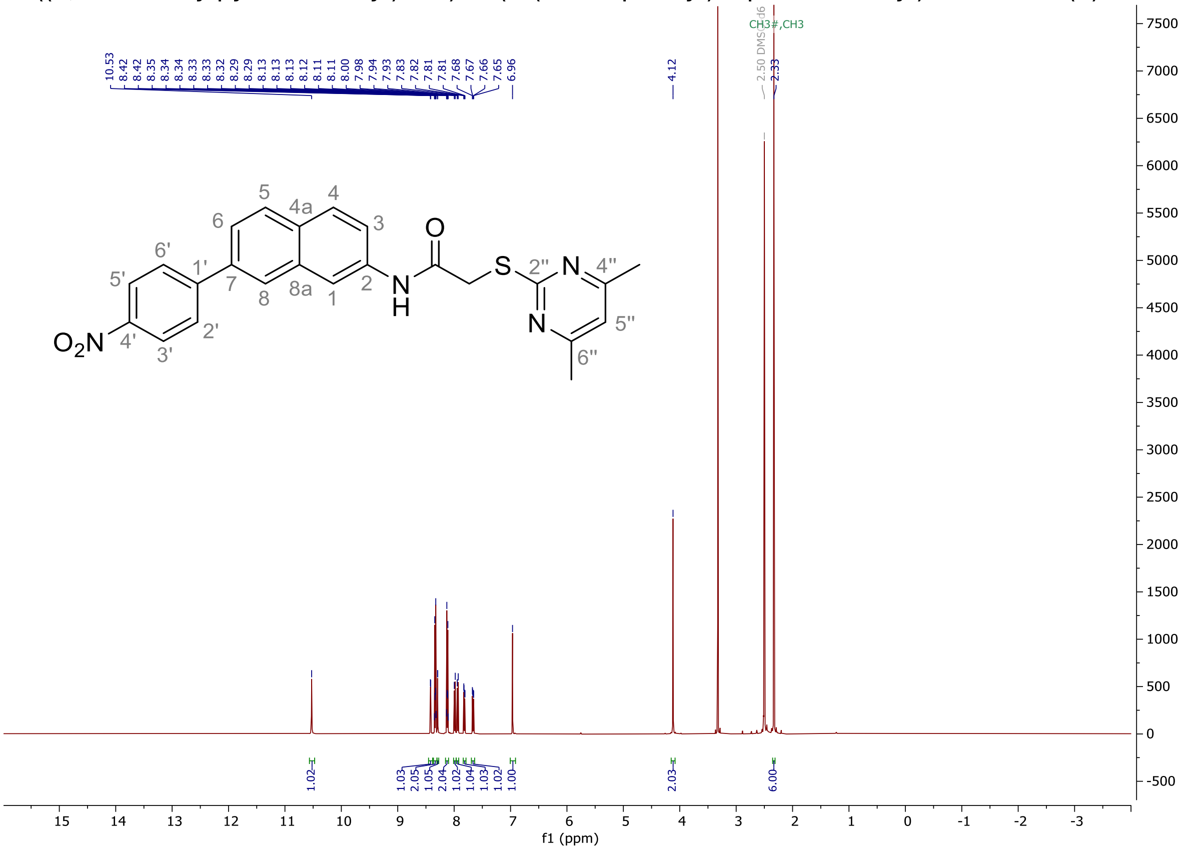

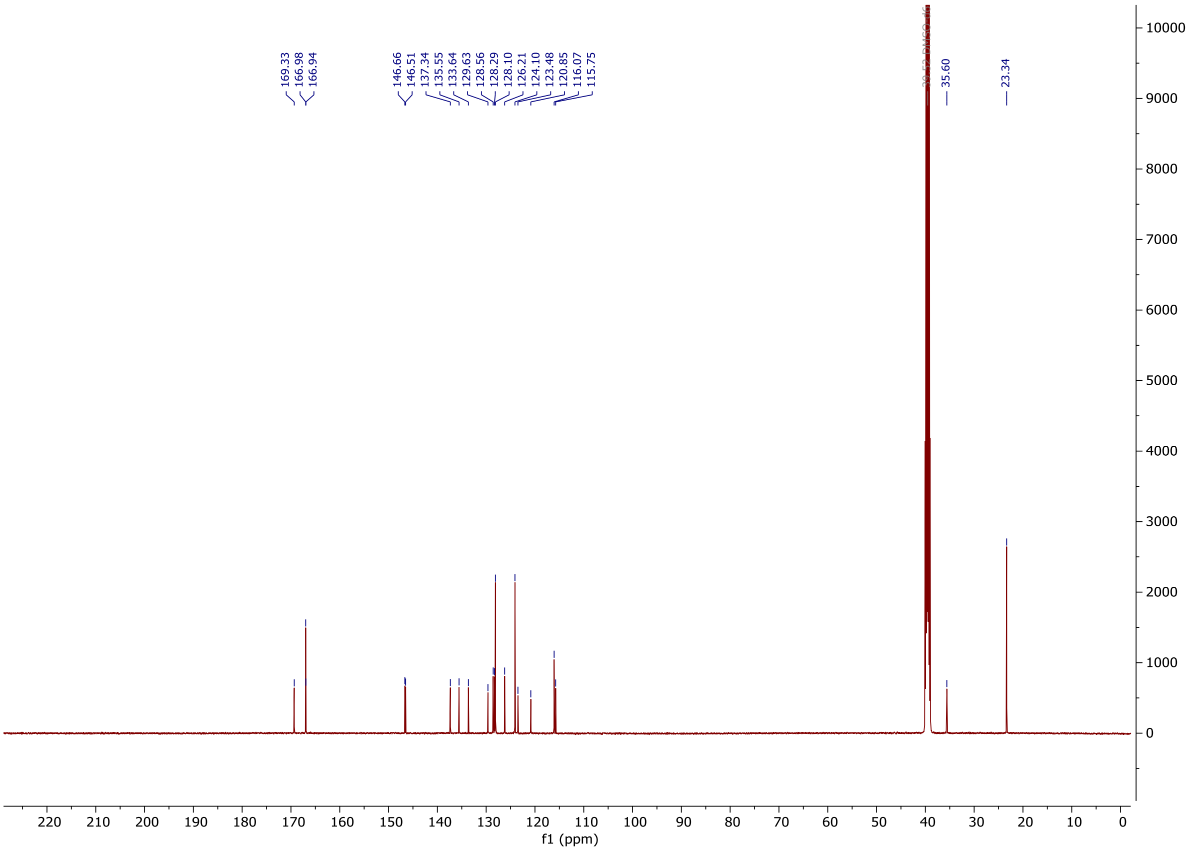

^1H and ^{13}C NMR spectra of

2-((4,6-Dimethylpyrimidin-2-yl)thio)-*N*-(7-(3-nitrophenyl)naphthalen-2-yl)acetamide (**8**)

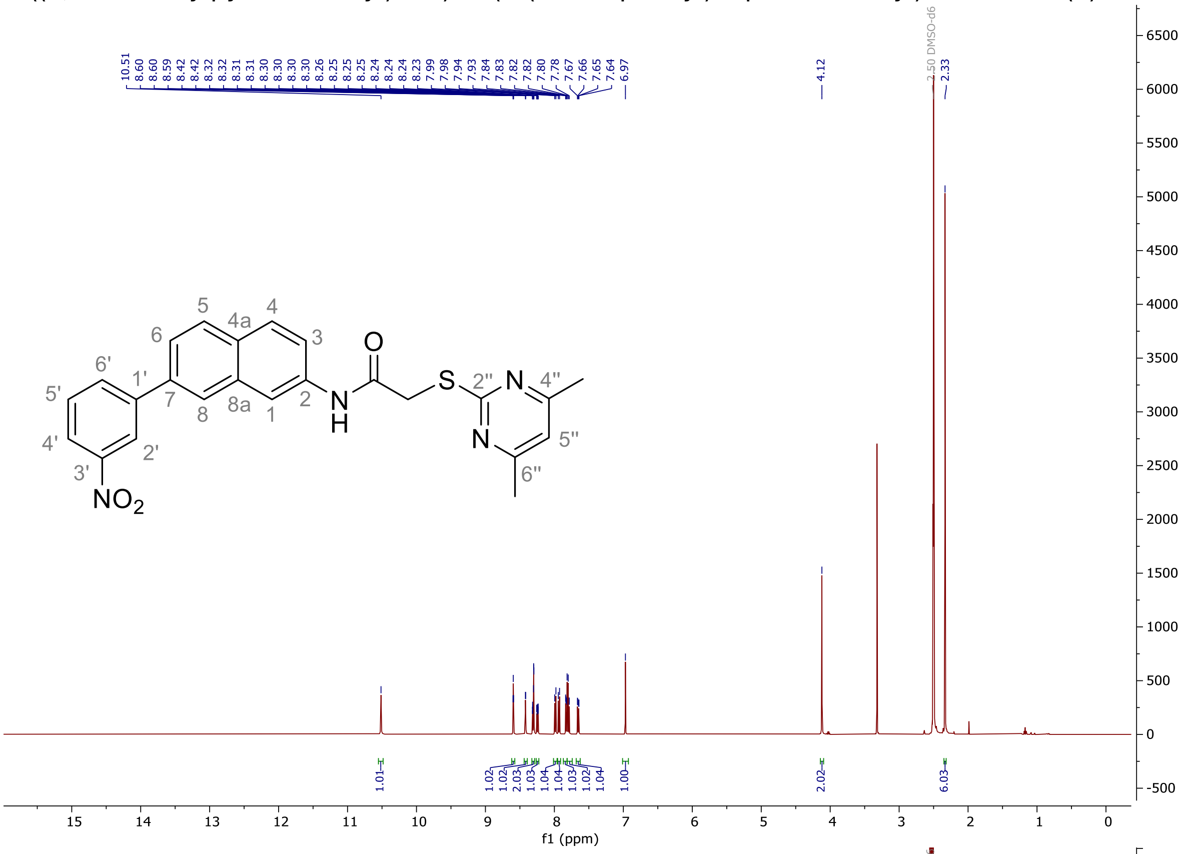

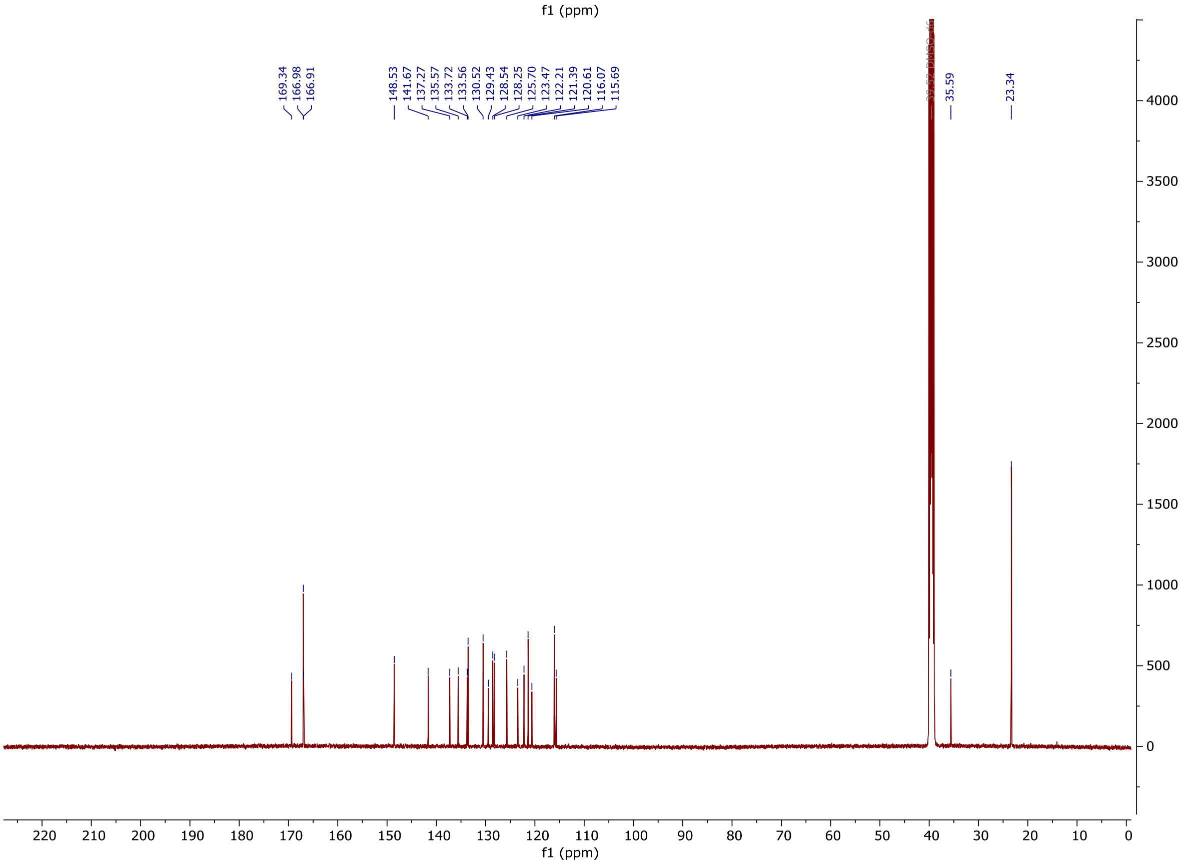

^{1}H and ^{13}C NMR spectra of
N-(7-(4-Aminophenyl)naphthalen-2-yl)-2-((4,6-dimethylpyrimidin-2-yl)thio)acetamide (**9**).

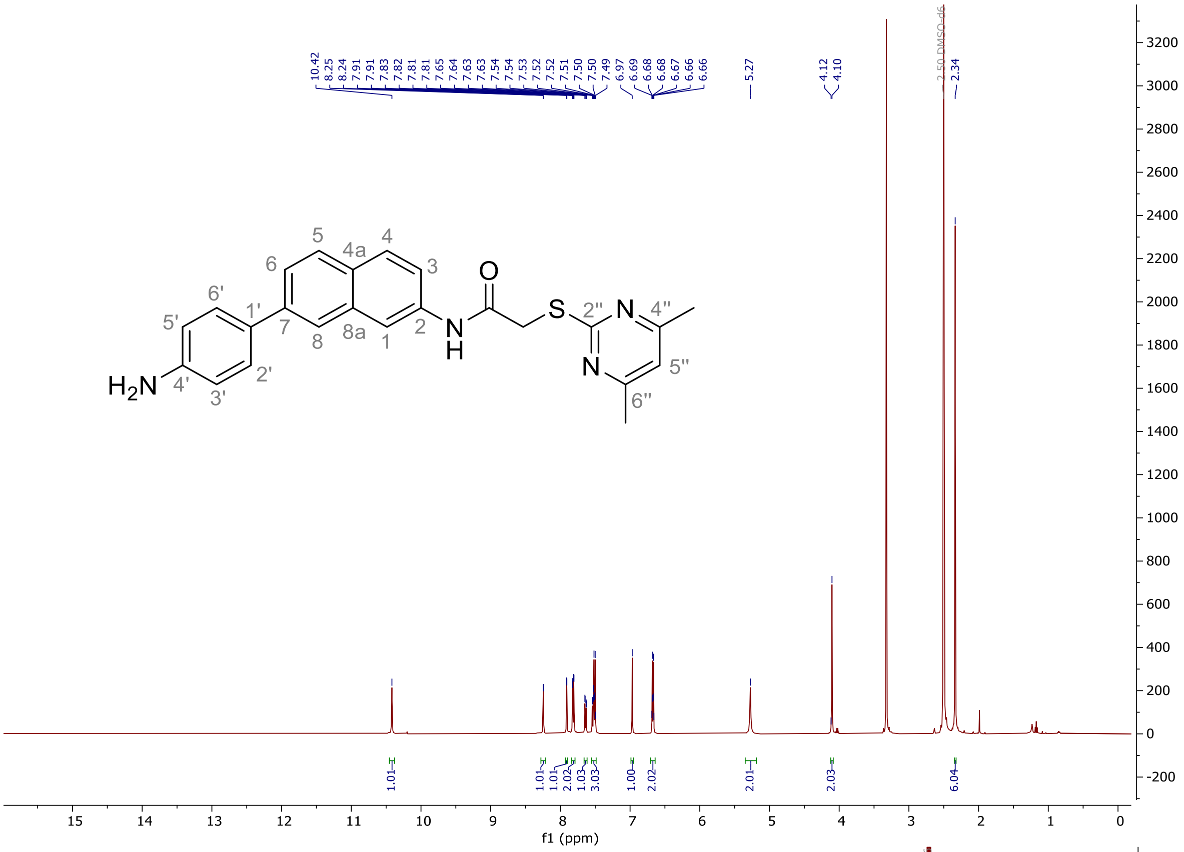

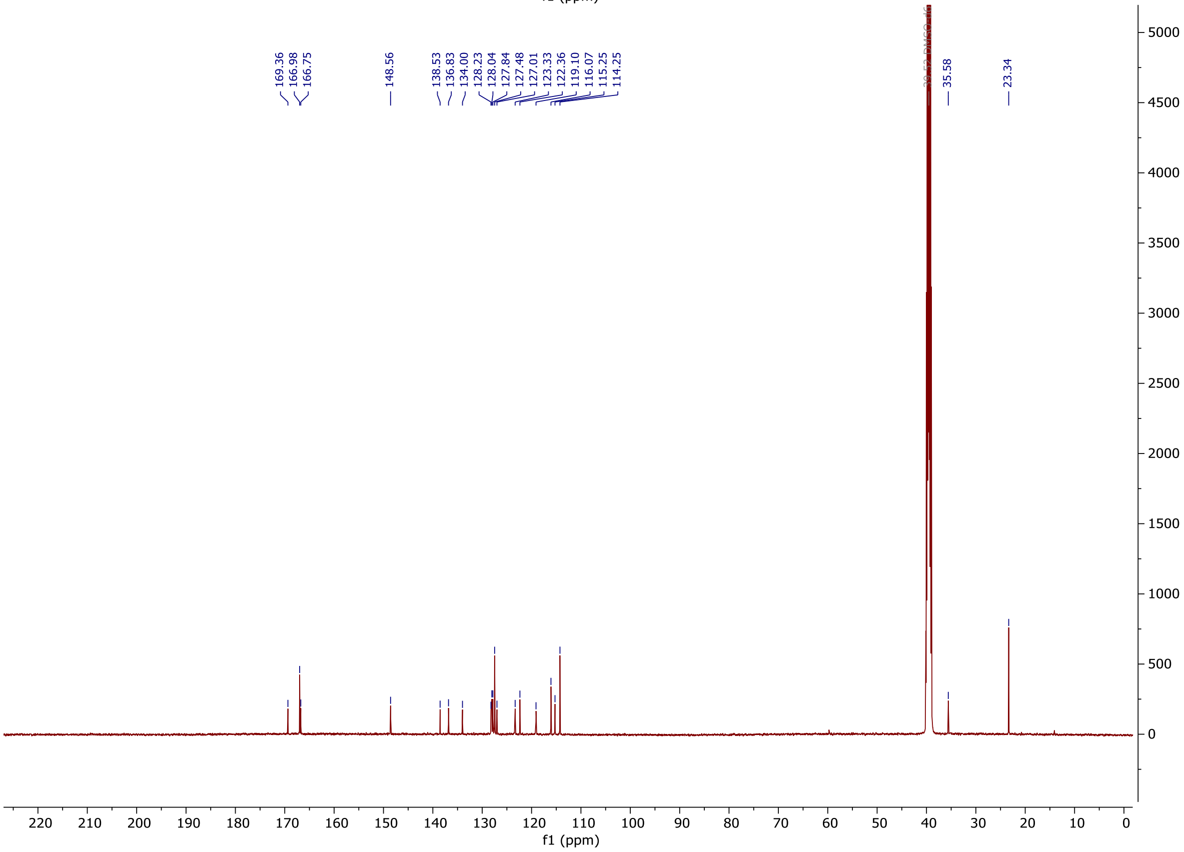

^{1}H and ^{13}C NMR spectra of

N-(7-(3-Aminophenyl)naphthalen-2-yl)-2-((4,6-dimethylpyrimidin-2-yl)thio)acetamide (**10**)

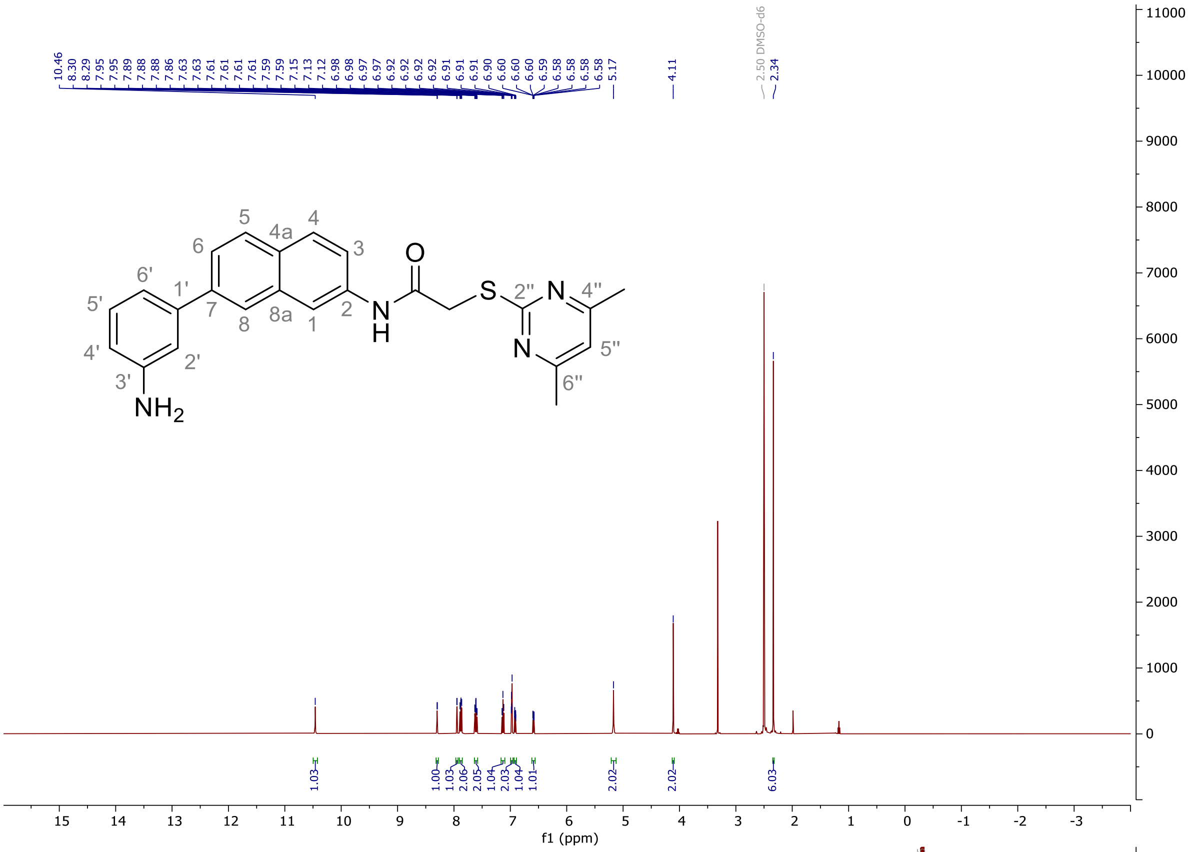

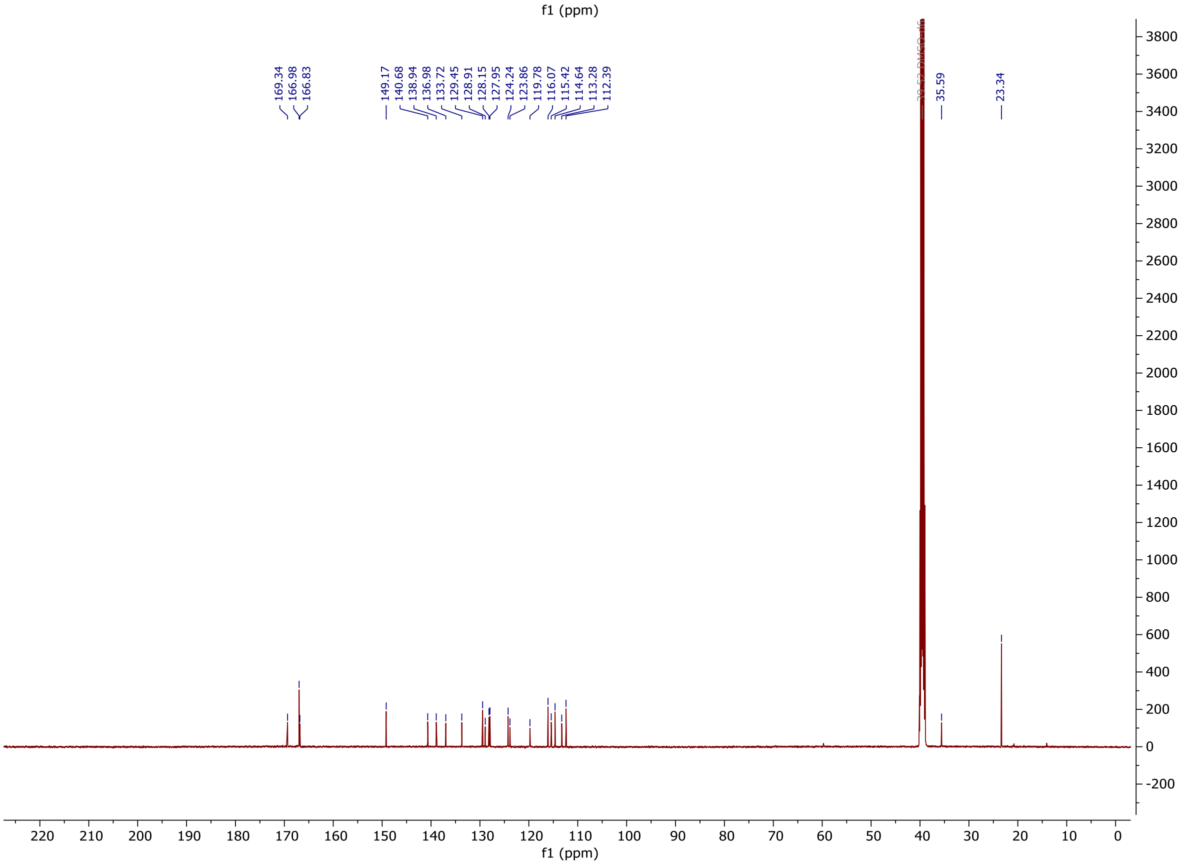

^{1}H and ^{13}C NMR spectra of
N-(7-(4-Acetamidophenyl)naphthalen-2-yl)-2-((4,6-dimethylpyrimidin-2-yl)thio)acetamide (**FM26**)

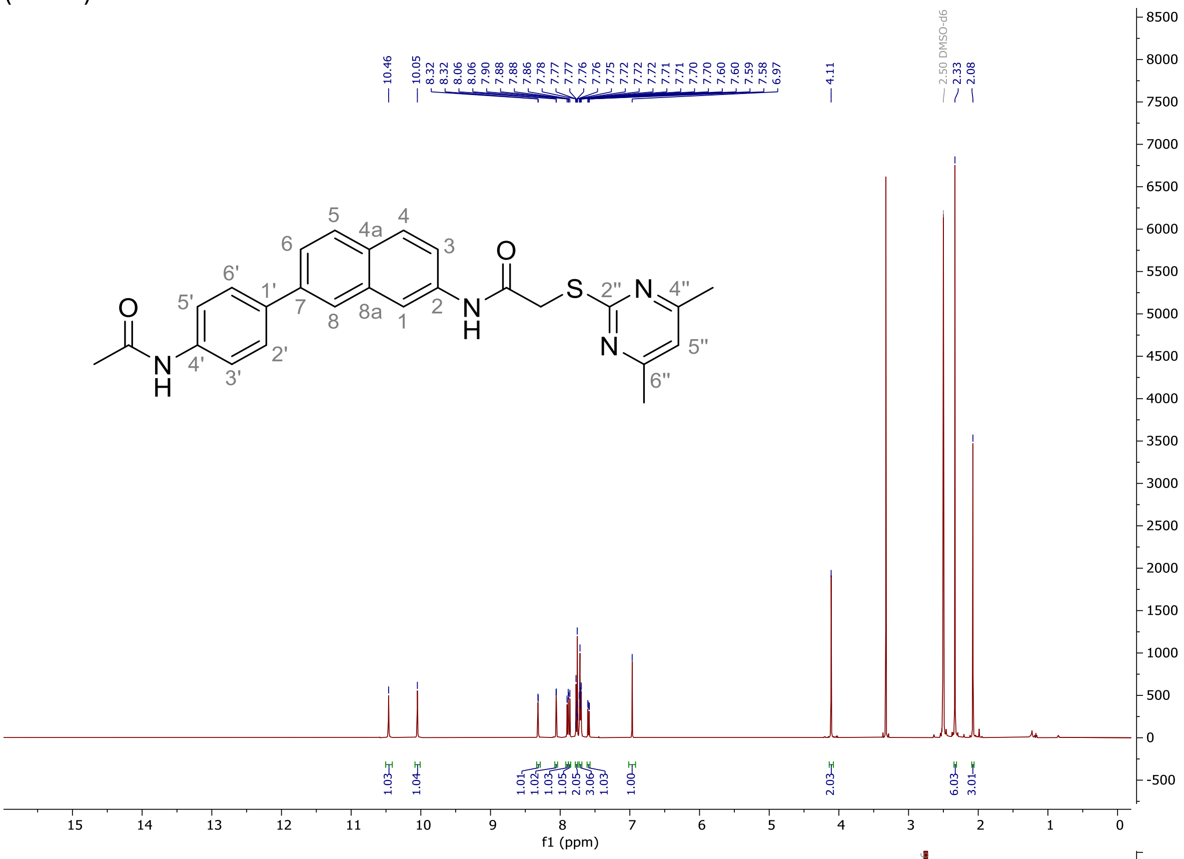

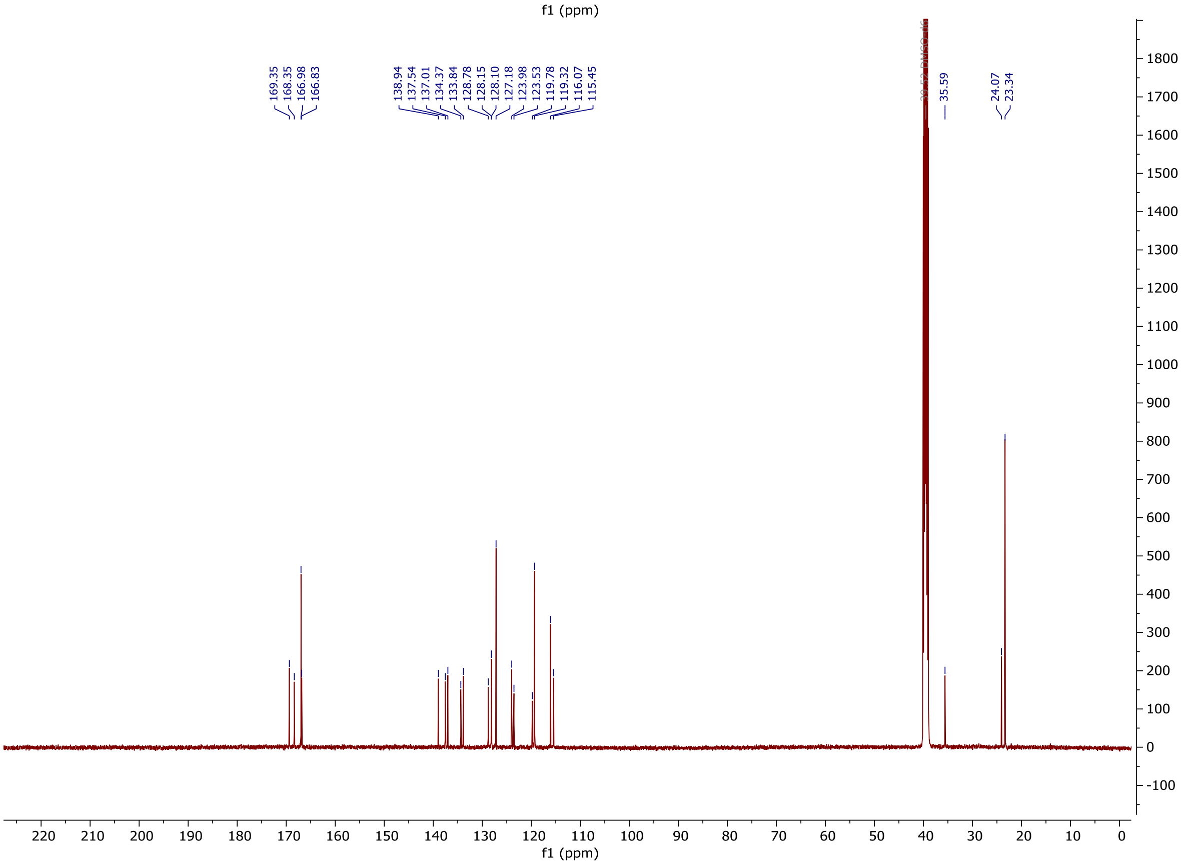

^{1}H and ^{13}C NMR spectra of
N-(7-(3-Acetamidophenyl)naphthalen-2-yl)-2-((4,6-dimethylpyrimidin-2-yl)thio)acetamide (**FM46**)

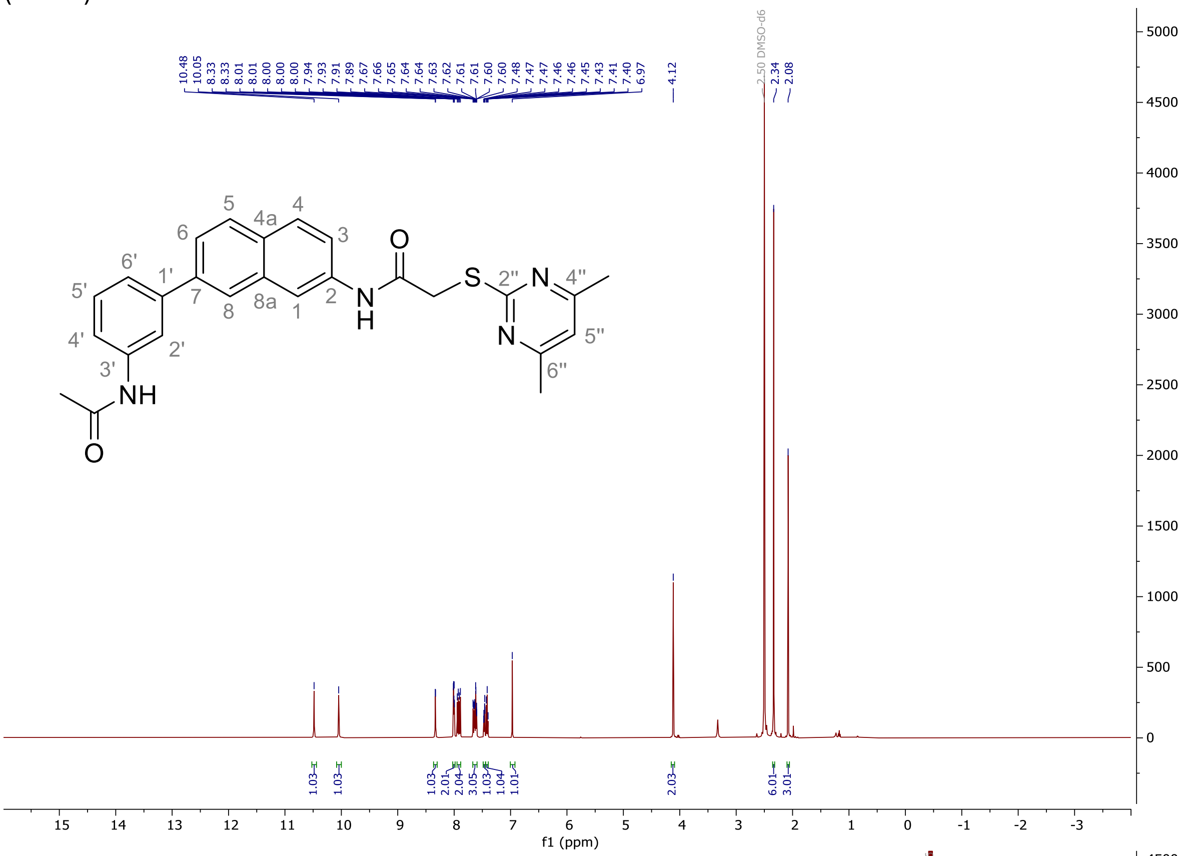

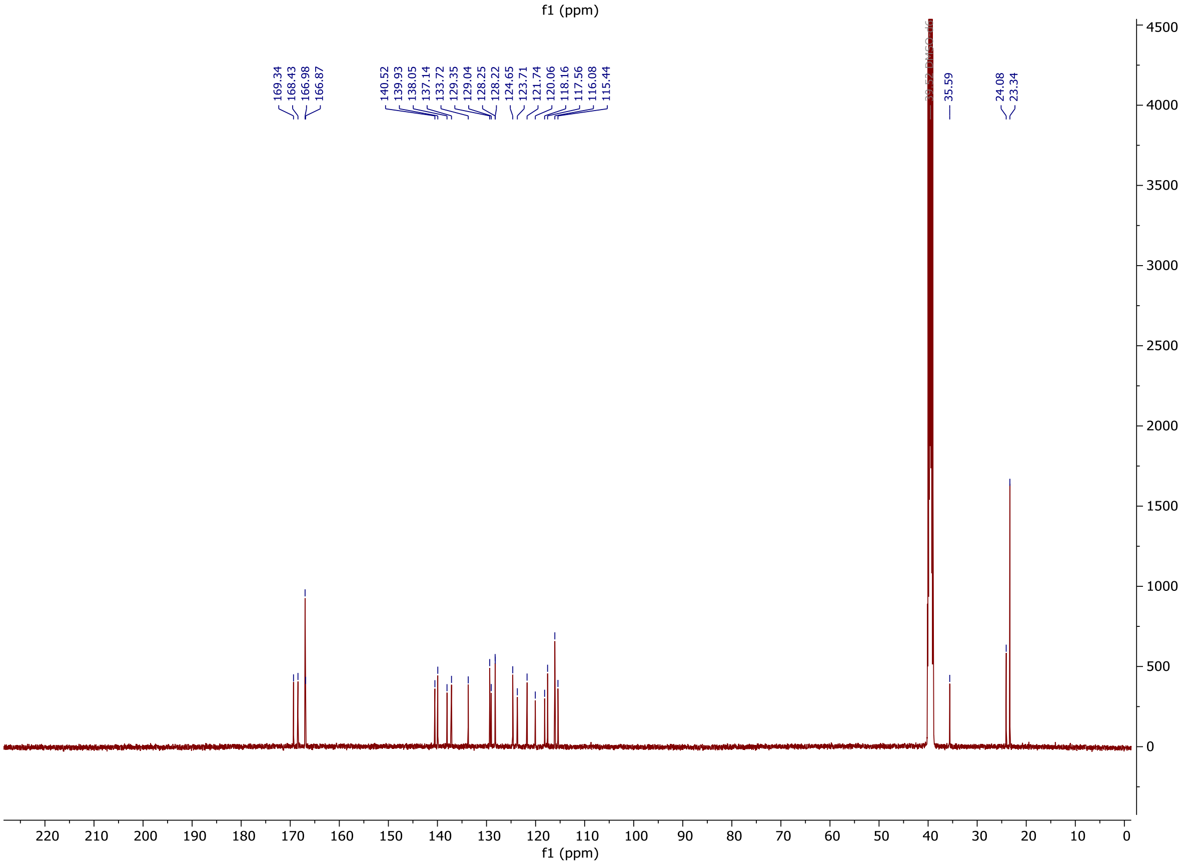

^{1}H and ^{13}C NMR spectra of 2-((4,6-Dimethylpyrimidin-2-yl)thio)-N-(7-(3-(phenylsulfonamido)phenyl)naphthalen-2-yl)acetamide (**FM47**)

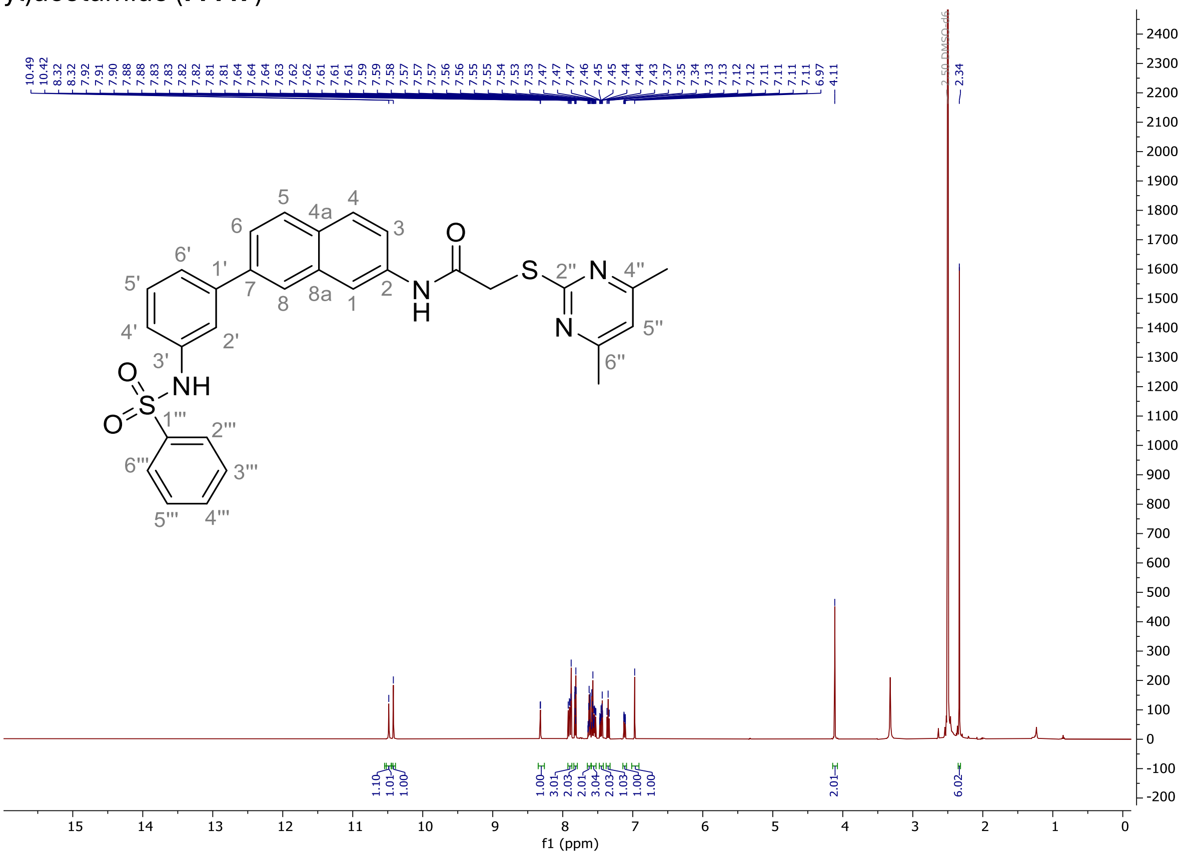

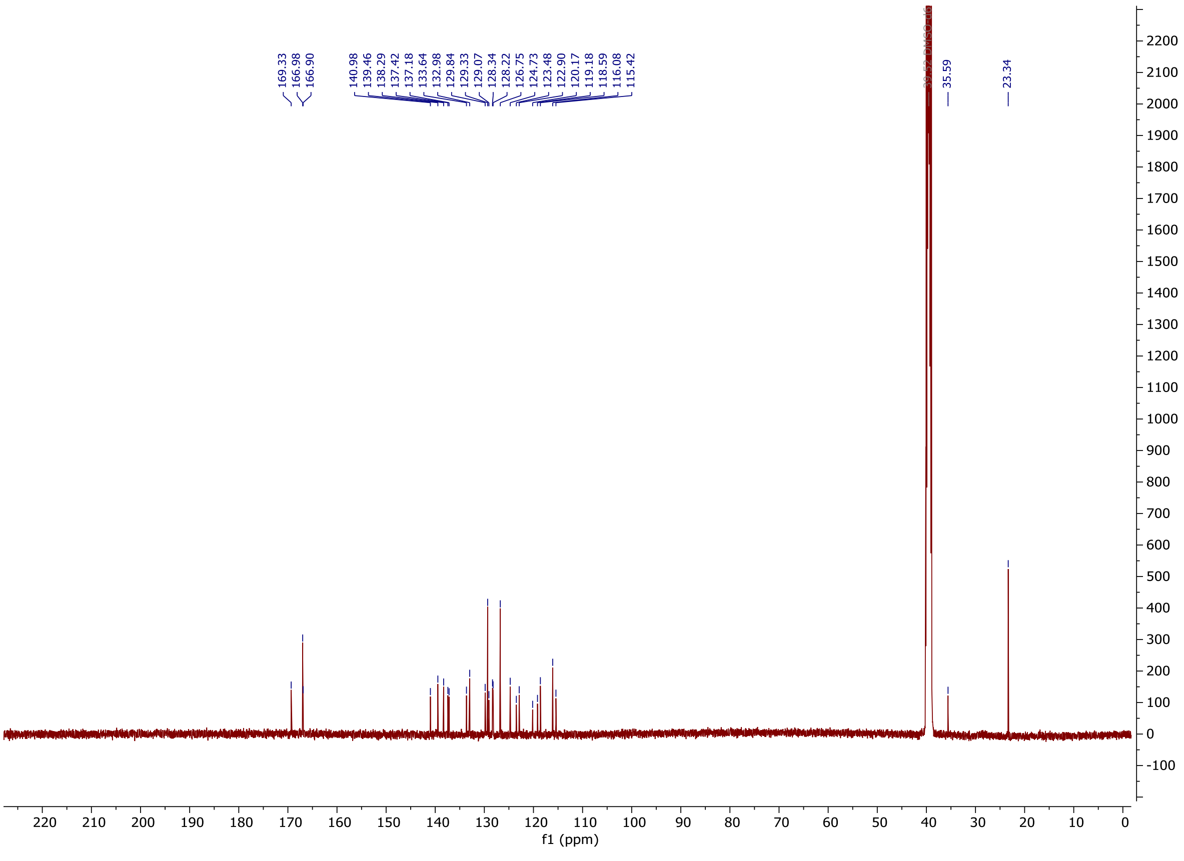

^1H and ^{13}C NMR spectra of N-(3-(7-(2-((4,6-Dimethylpyrimidin-2-yl)thio)acetamido)naphthalen-2-yl)phenyl)thiophene-2-carboxamide (**FM48**)

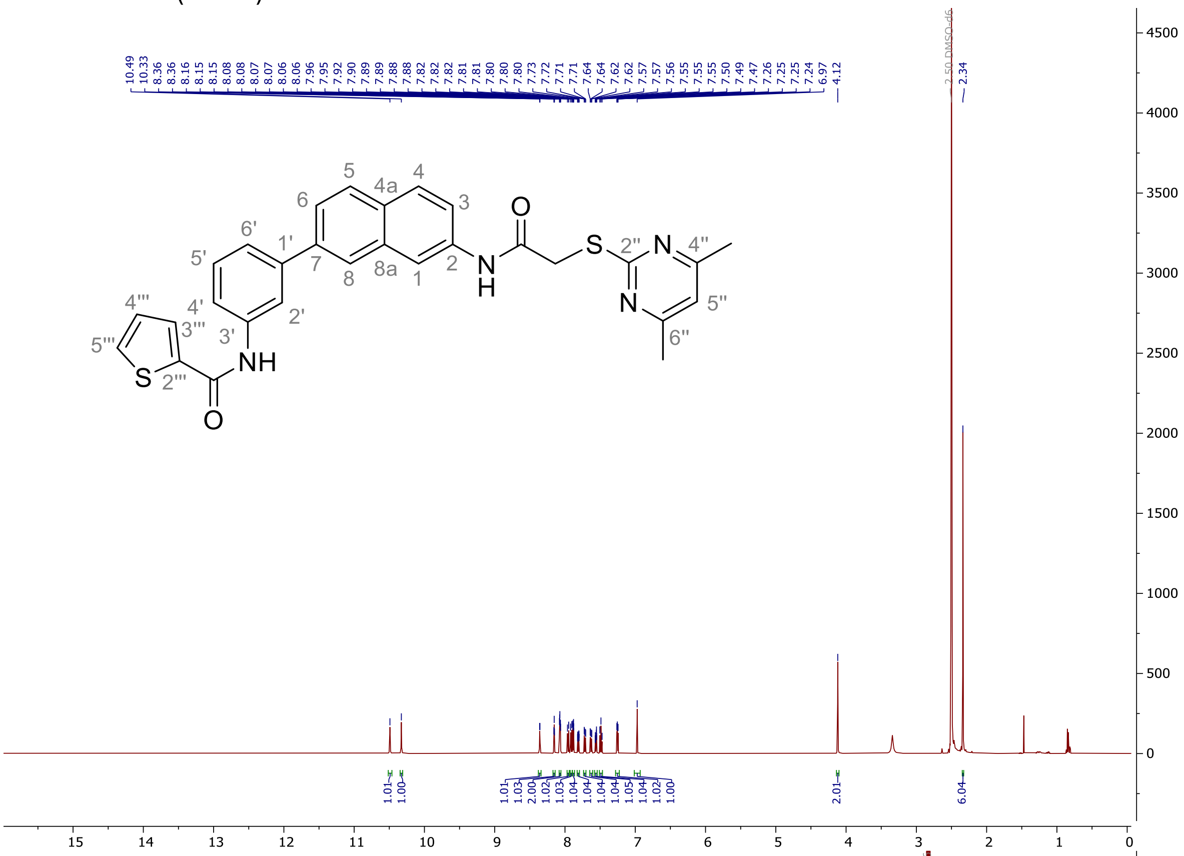

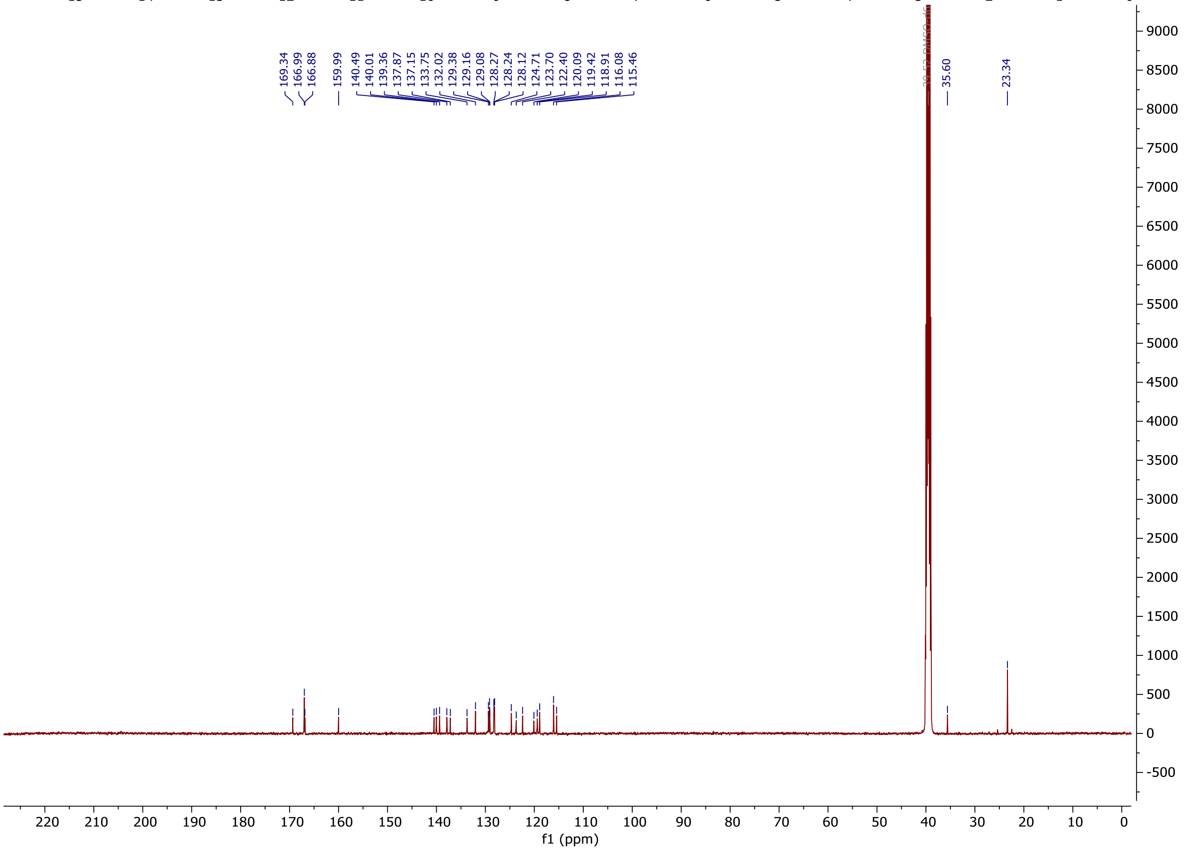

^{1}H and ^{13}C NMR spectra of
N-(4-(7-(2-((4,6-Dimethylpyrimidin-2-yl)thio)acetamido)naphthalen-2-yl)phenyl)thiophene-2-carboxamide (**FM50**)

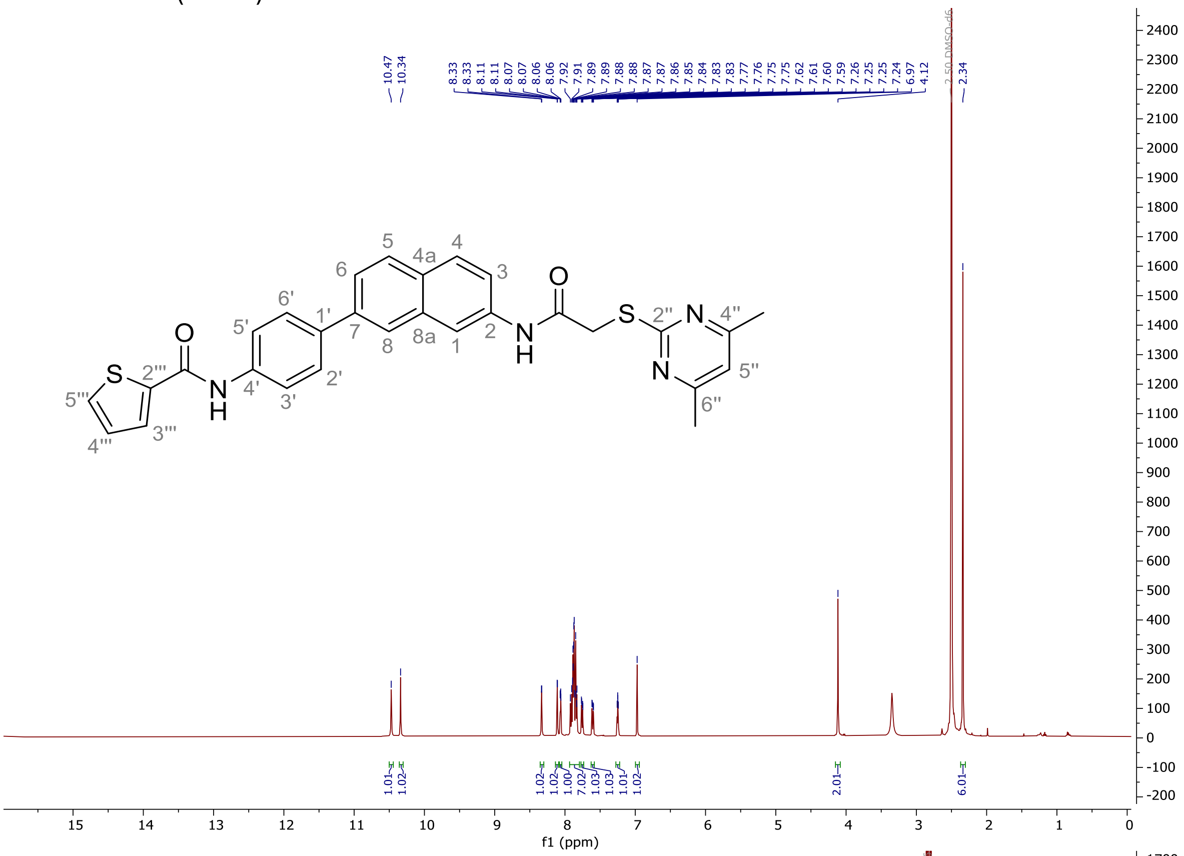

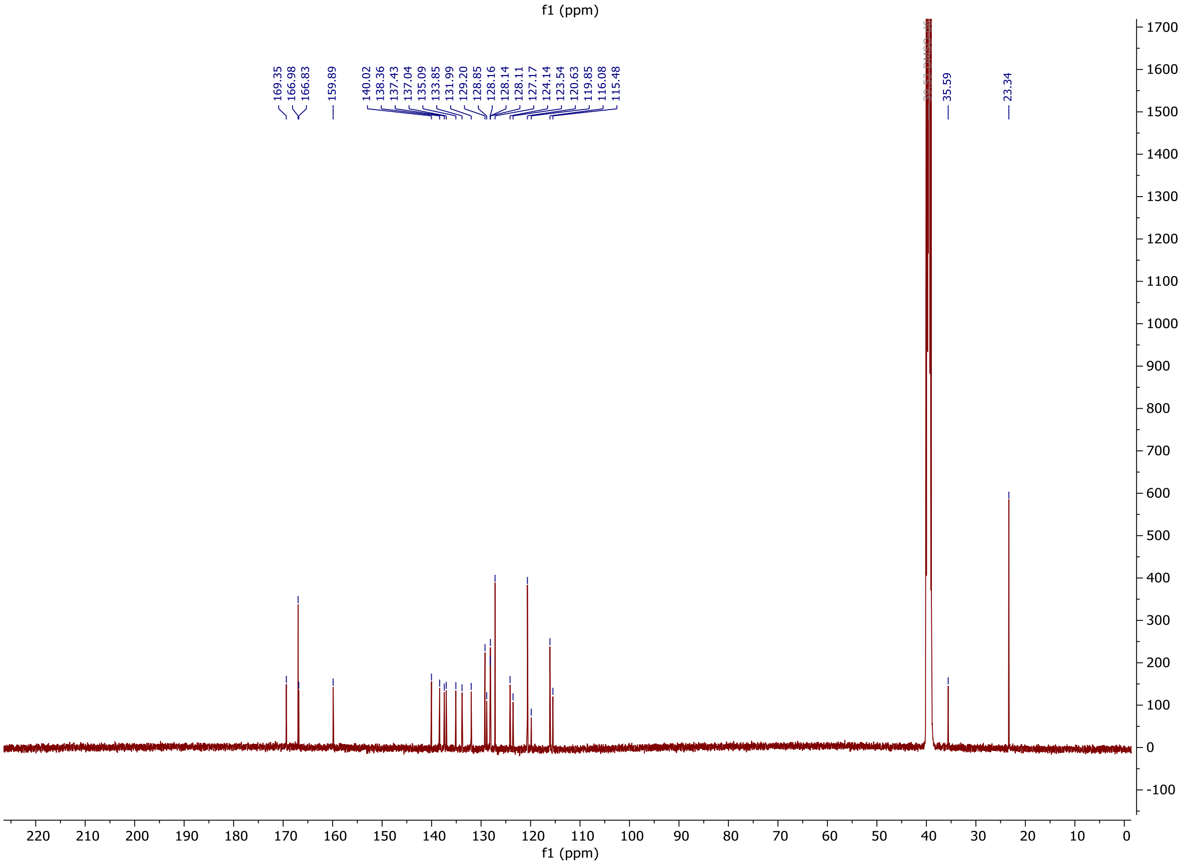

^{1}H and ^{13}C NMR spectra of
N-(4-(7-(2-((4,6-Dimethylpyrimidin-2-yl)thio)acetamido)naphthalen-2-yl)phenyl)benzamide (**FM53**)

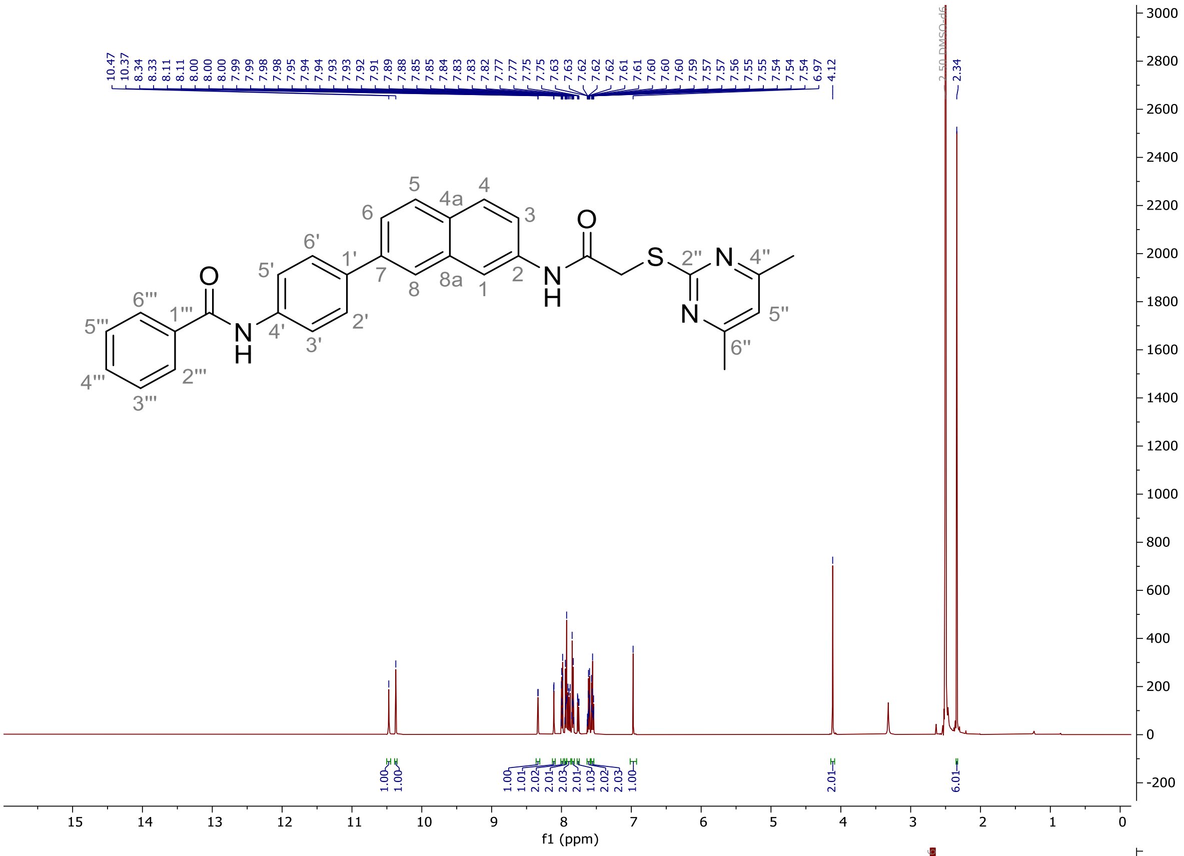

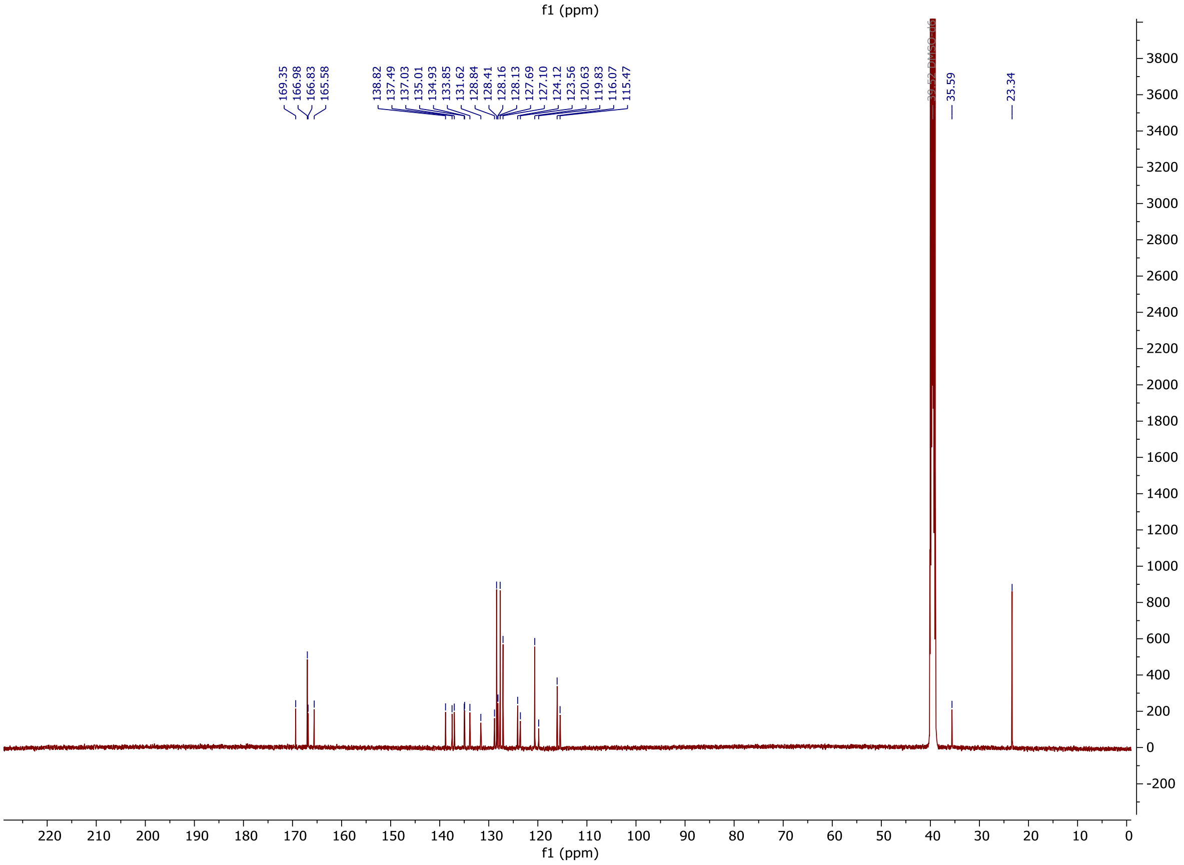

^{1}H and ^{13}C NMR spectra of *N*-(3-(7-(2-((4,6-Dimethylpyrimidin-2-yl)thio)acetamido)naphthalen-2-yl)phenyl)benzamide (**FM54**)

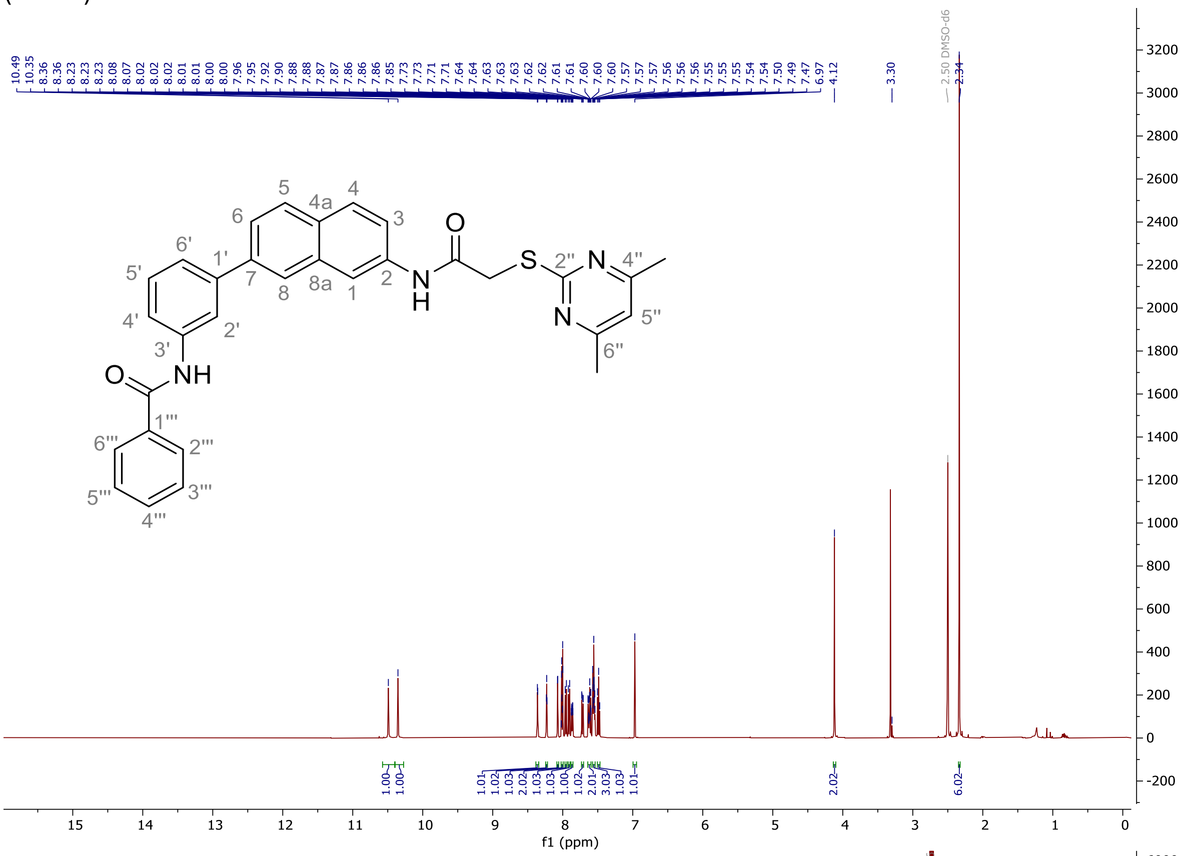

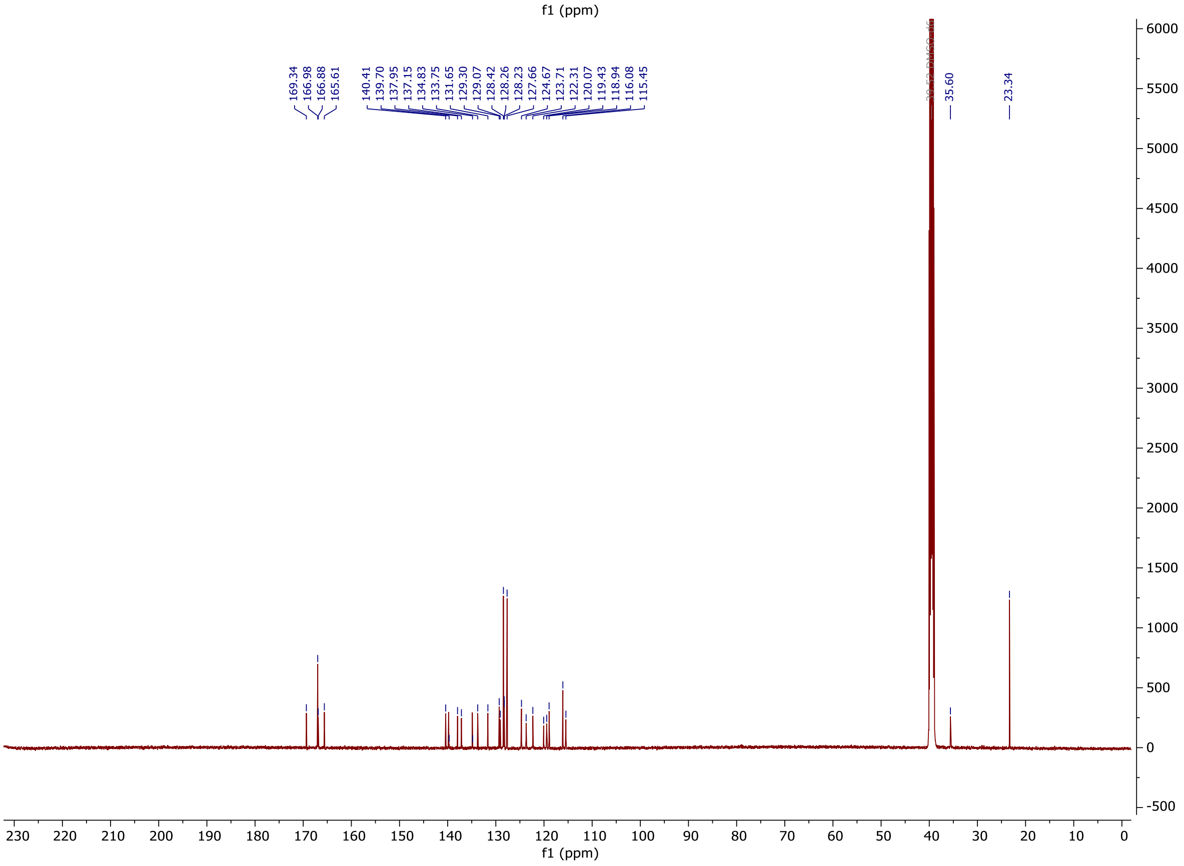

^{1}H and ^{13}C NMR spectra of
2-((4,6-Dimethylpyrimidin-2-yl)thio)-*N*-(7-(4-(phenylsulfonamido)phenyl)naphthalen-2-yl)acetamide (**FM56**)

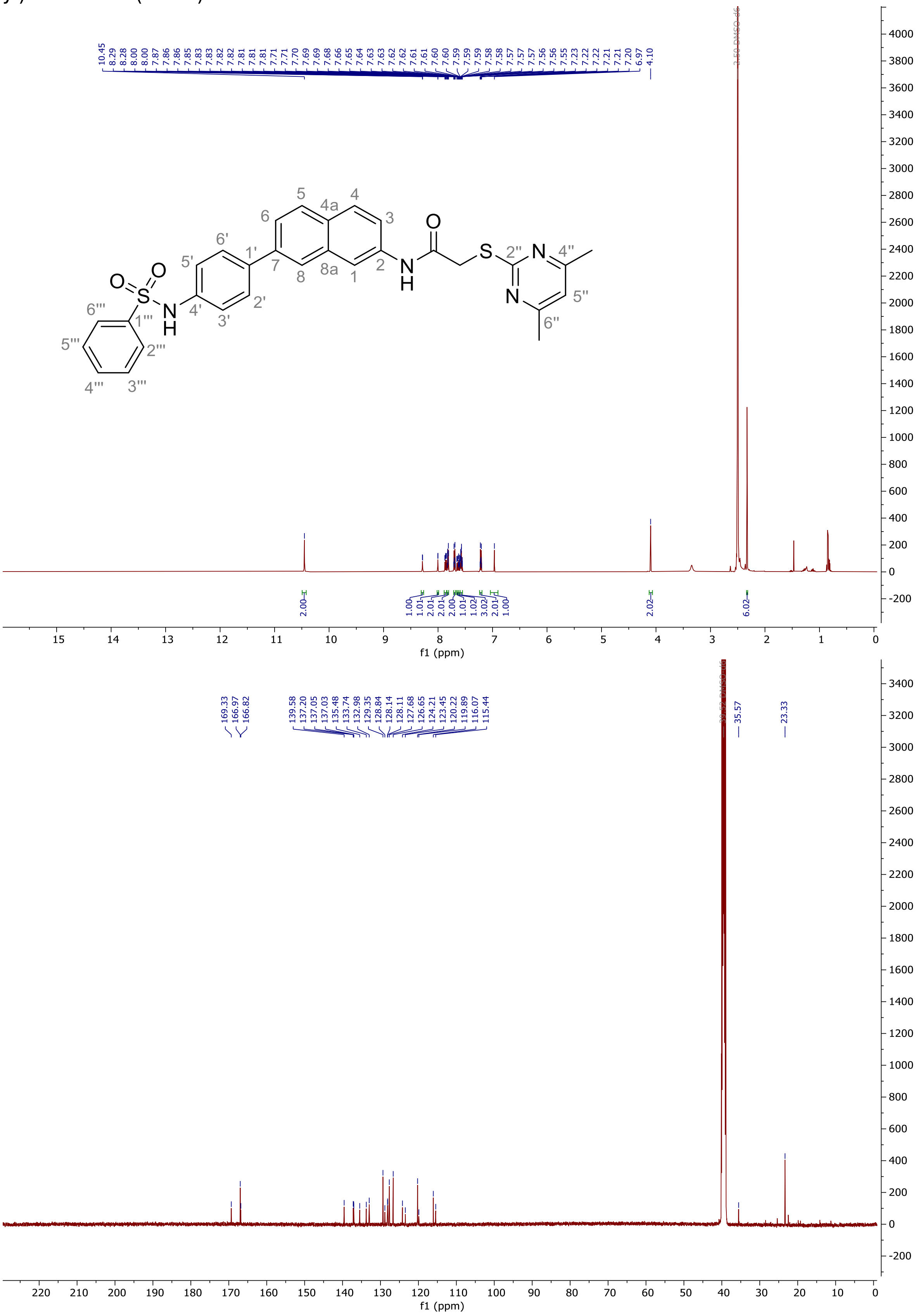

^{1}H and ^{13}C NMR spectra of
N-(3-(7-(2-((4,6-Dimethylpyrimidin-2-yl)thio)acetamido)naphthalen-2-yl)phenyl)-5-methylthiophene-2-carboxamide (**FM66**)

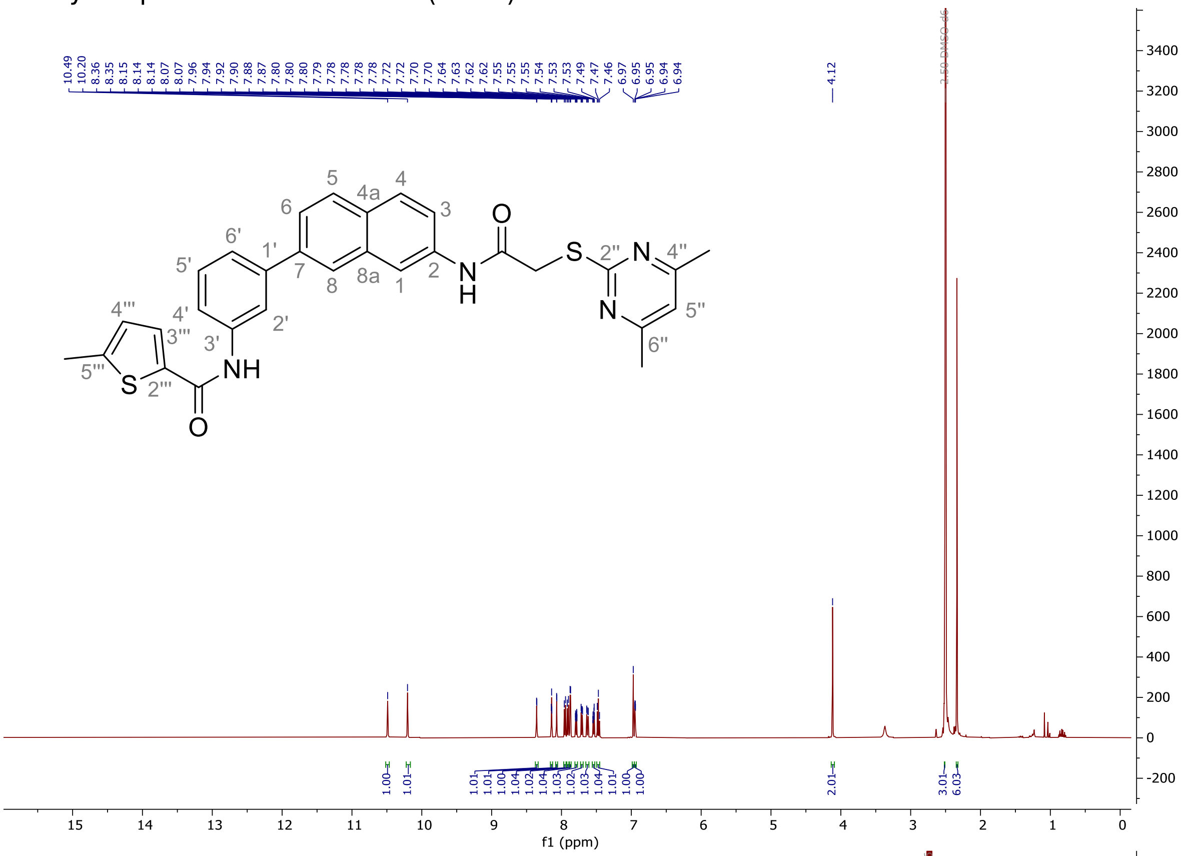

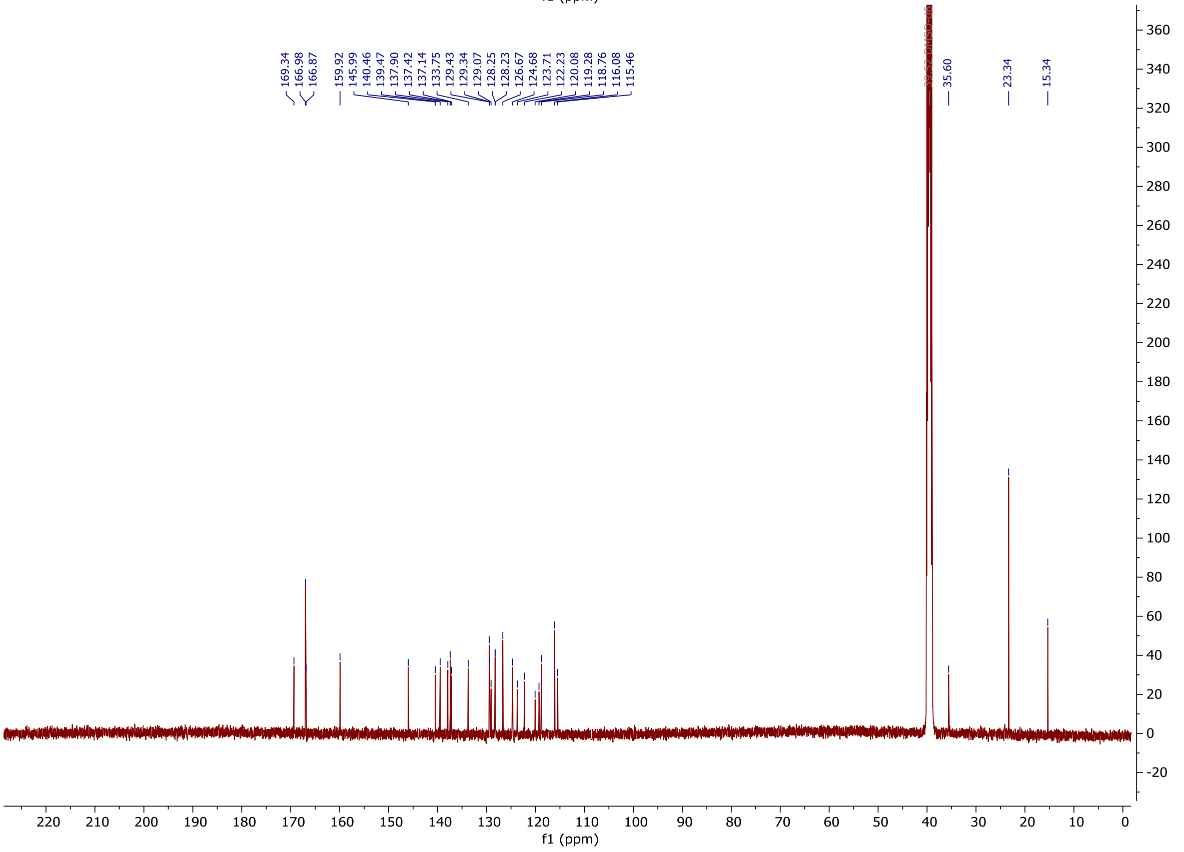

^{1}H and ^{13}C NMR spectra of
N-(4-(7-(2-((4,6-Dimethylpyrimidin-2-yl)thio)acetamido)naphthalen-2-yl)phenyl)-5-methylthiophene-2-carboxamide (**FM69**)

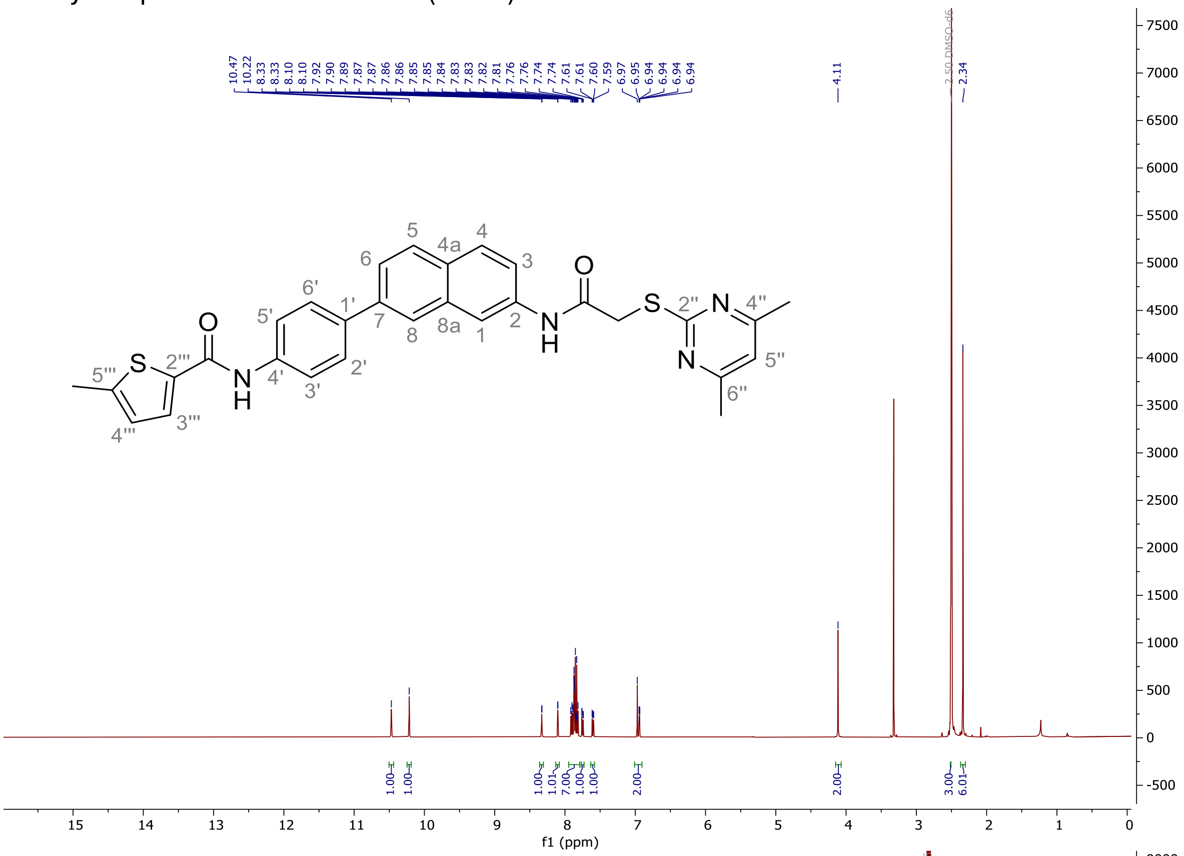

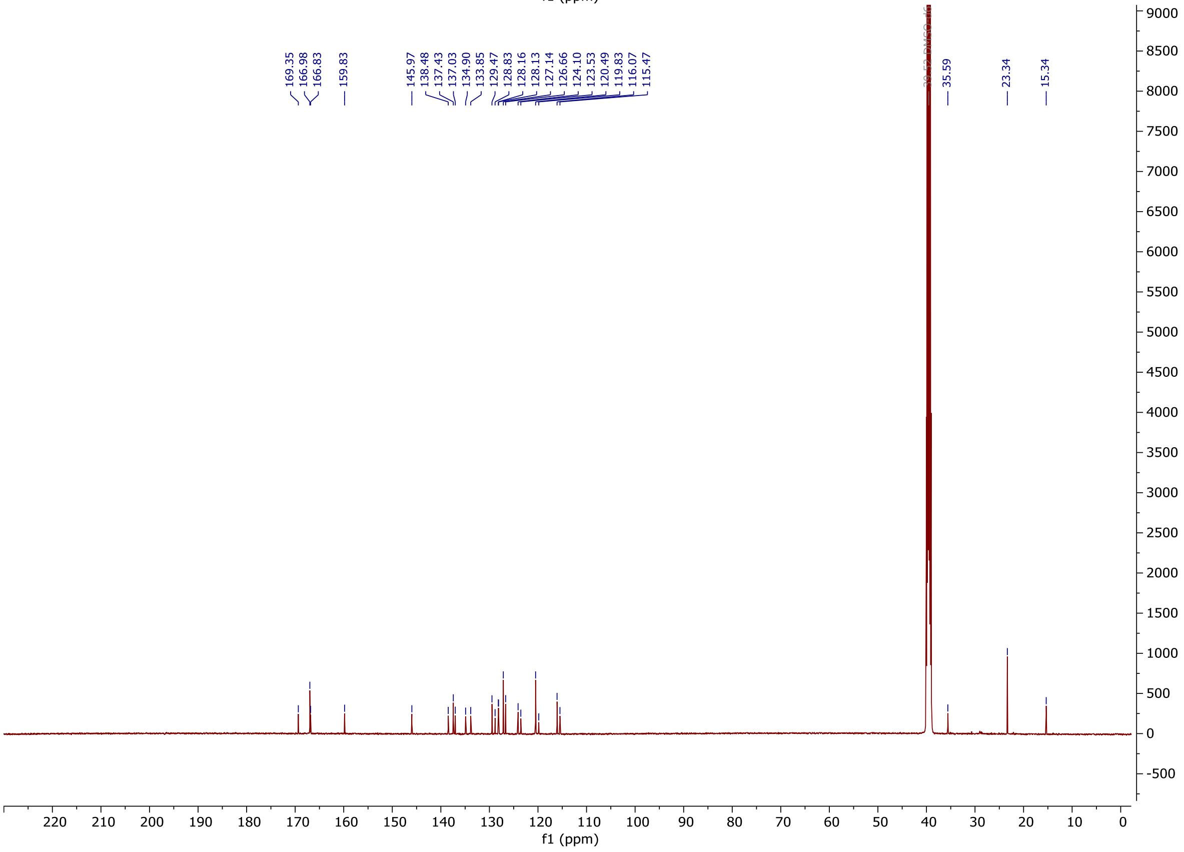

^1H and ^{13}C NMR spectra of 6-(3-Nitrophenyl)benzo[d]thiazol-2-amine (**13**).

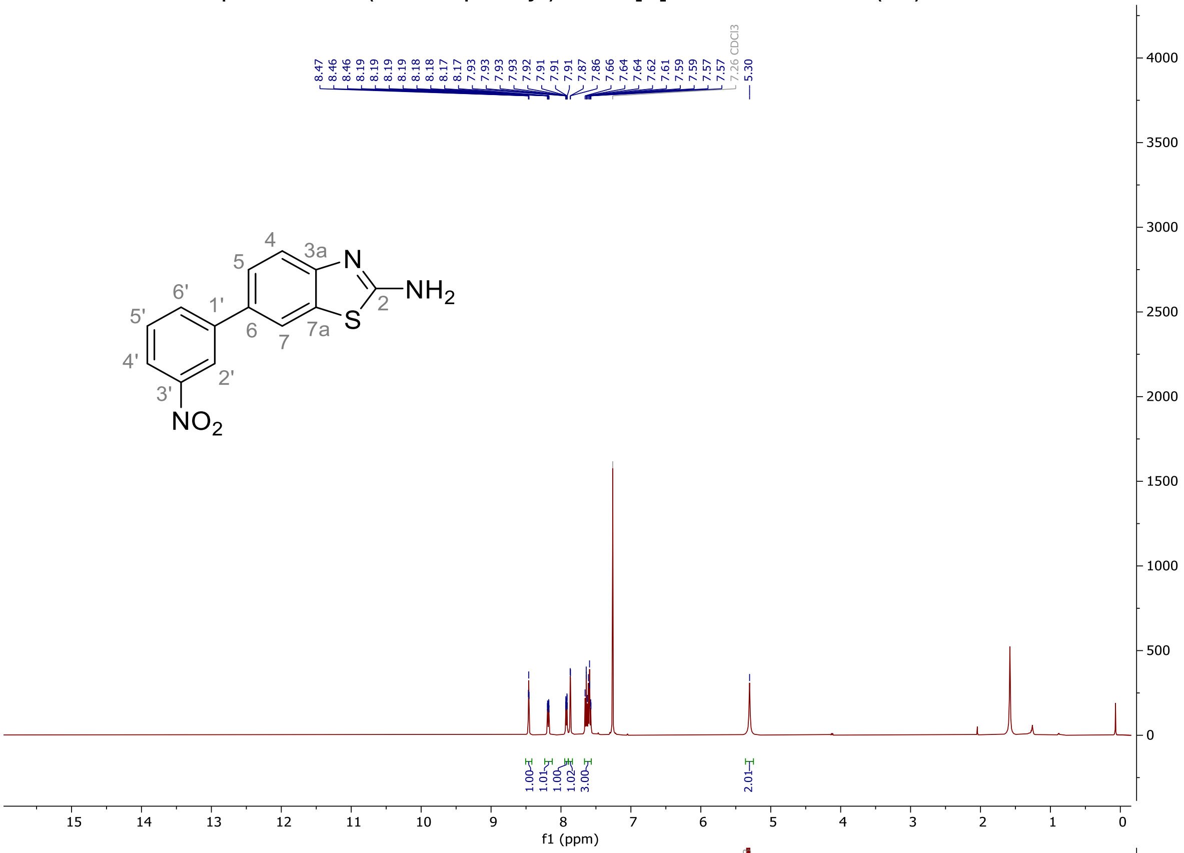

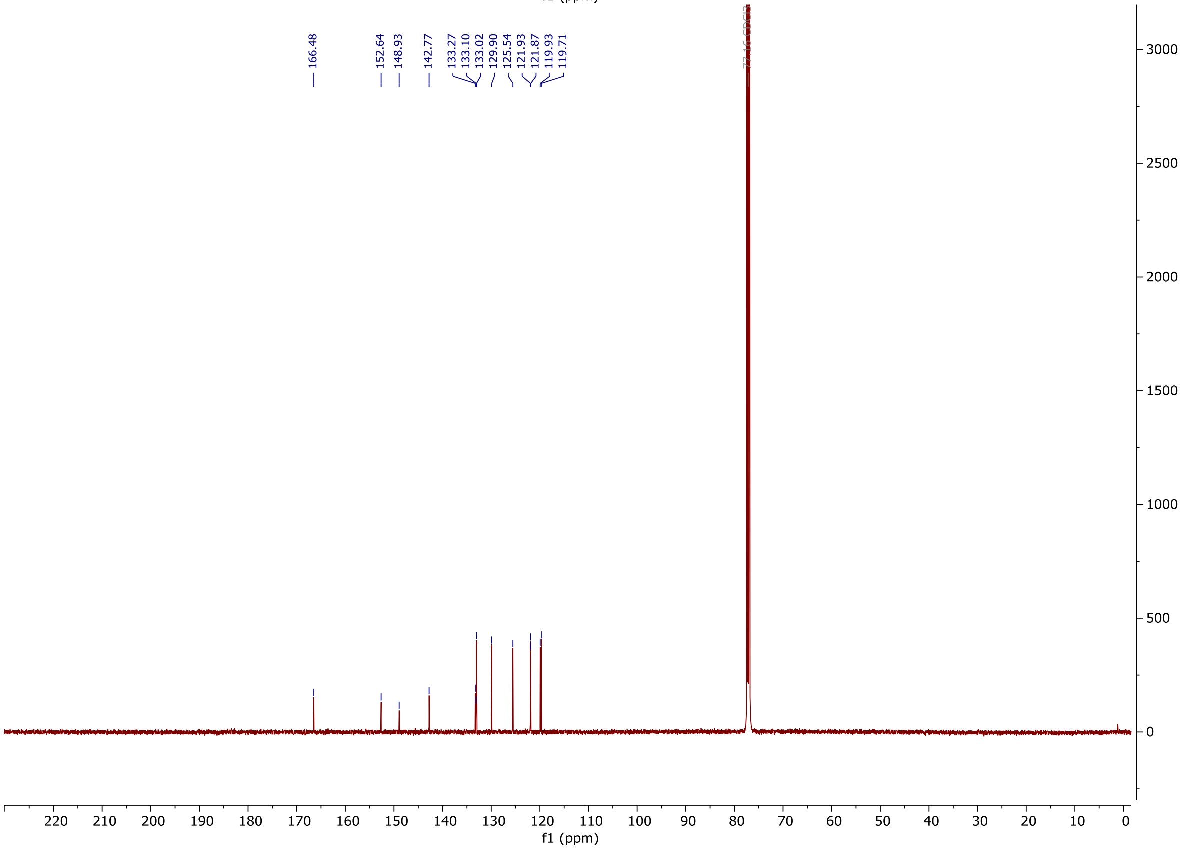

^{1}H and ^{13}C NMR spectra of *tert*-Butyl (4-(2-aminobenzo[d]thiazol-6-yl)phenyl)carbamate (**14**)

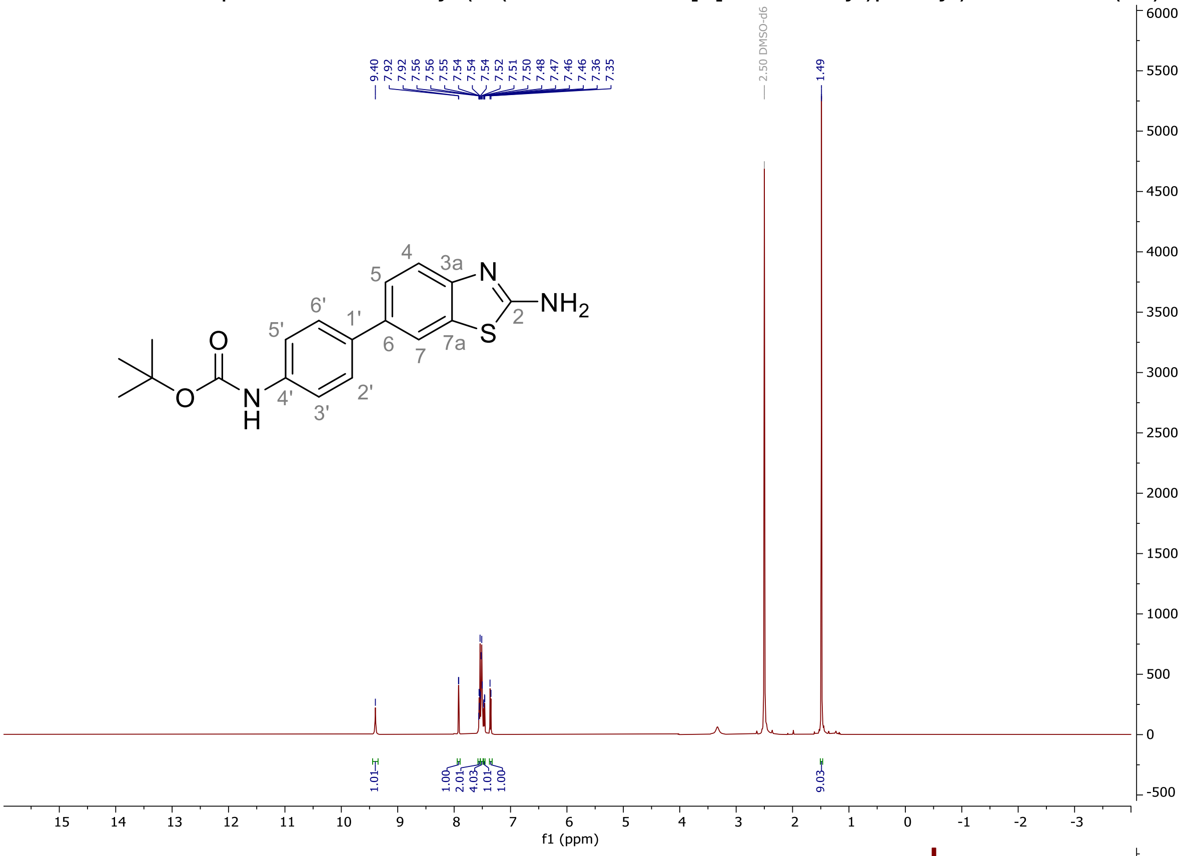

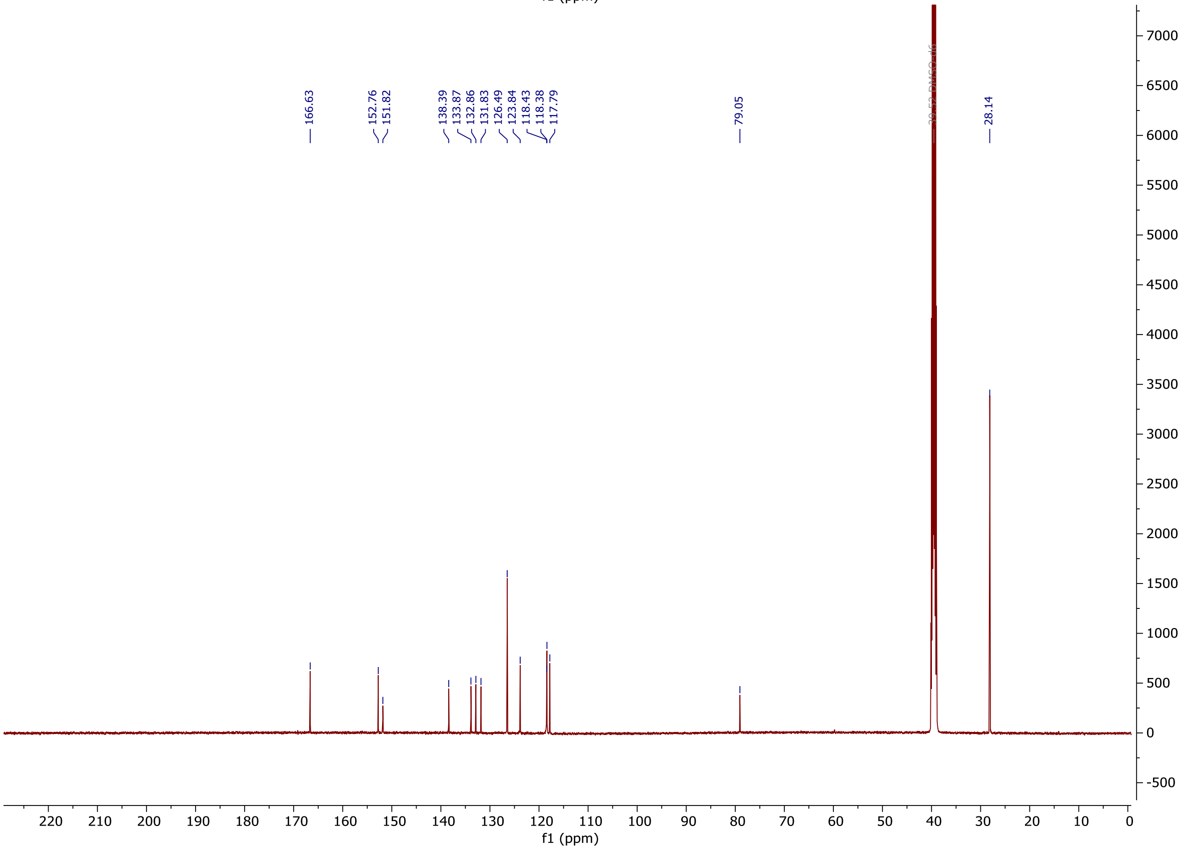

^{1}H and ^{13}C NMR spectra of 2-Bromo-*N*-(6-(3-nitrophenyl)benzo[d]thiazol-2-yl)acetamide (**15**)

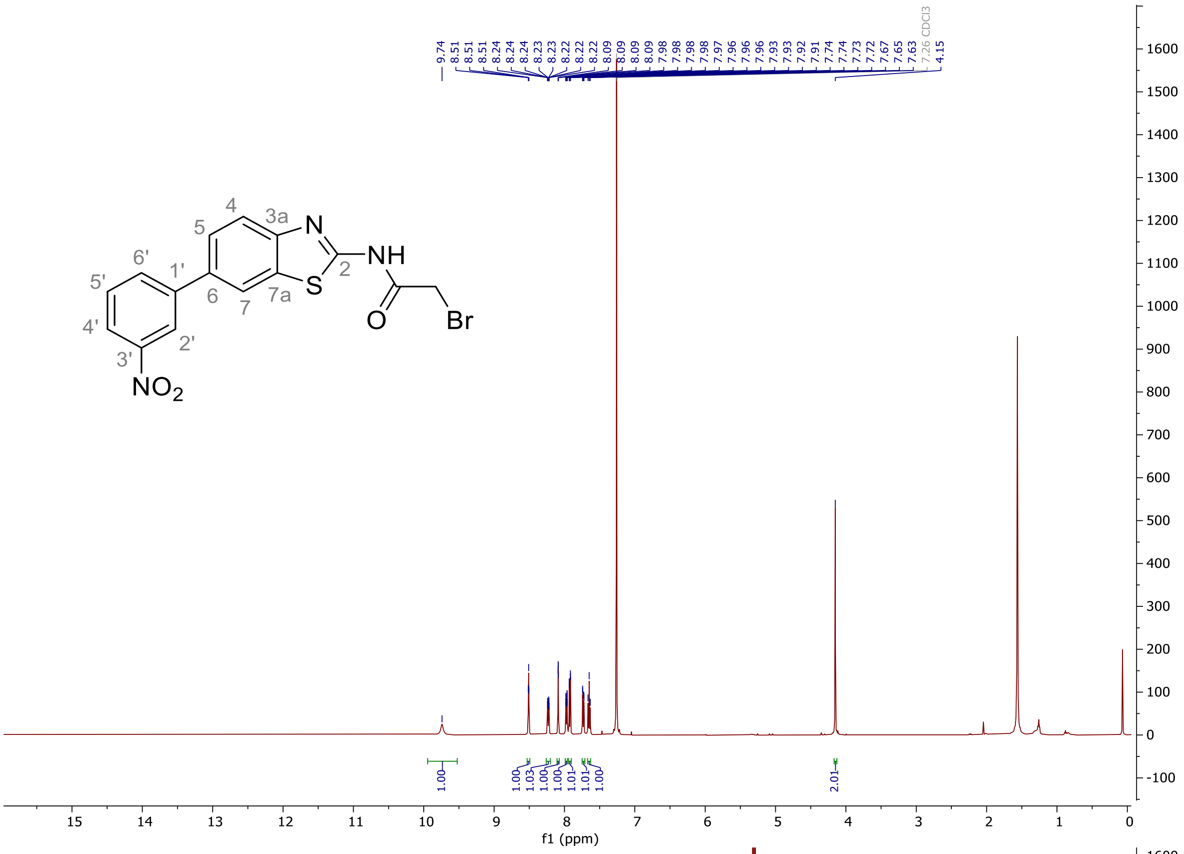

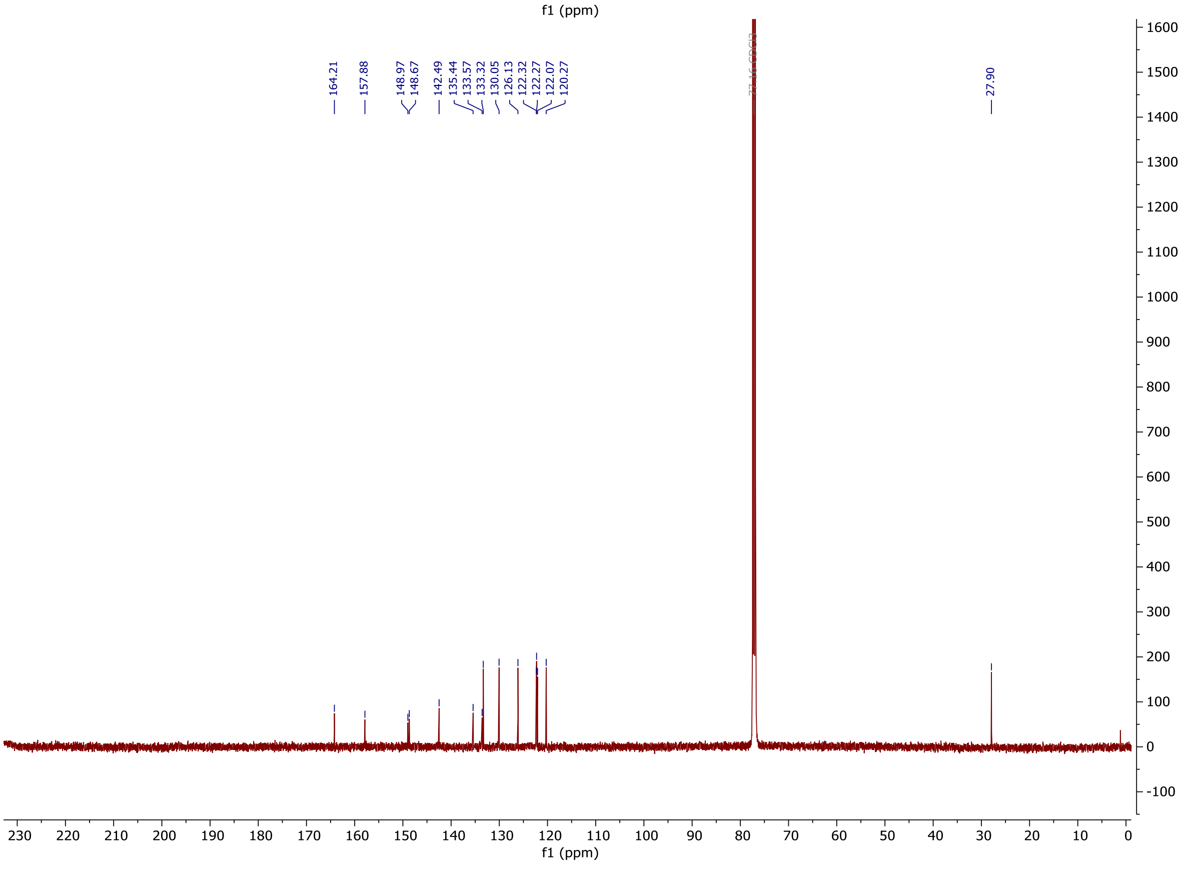

^{1}H and ^{13}C NMR spectra of

tert-Butyl (4-(2-(2-bromoacetamido)benzo[d]thiazol-6-yl)phenyl)carbamate (**16**)

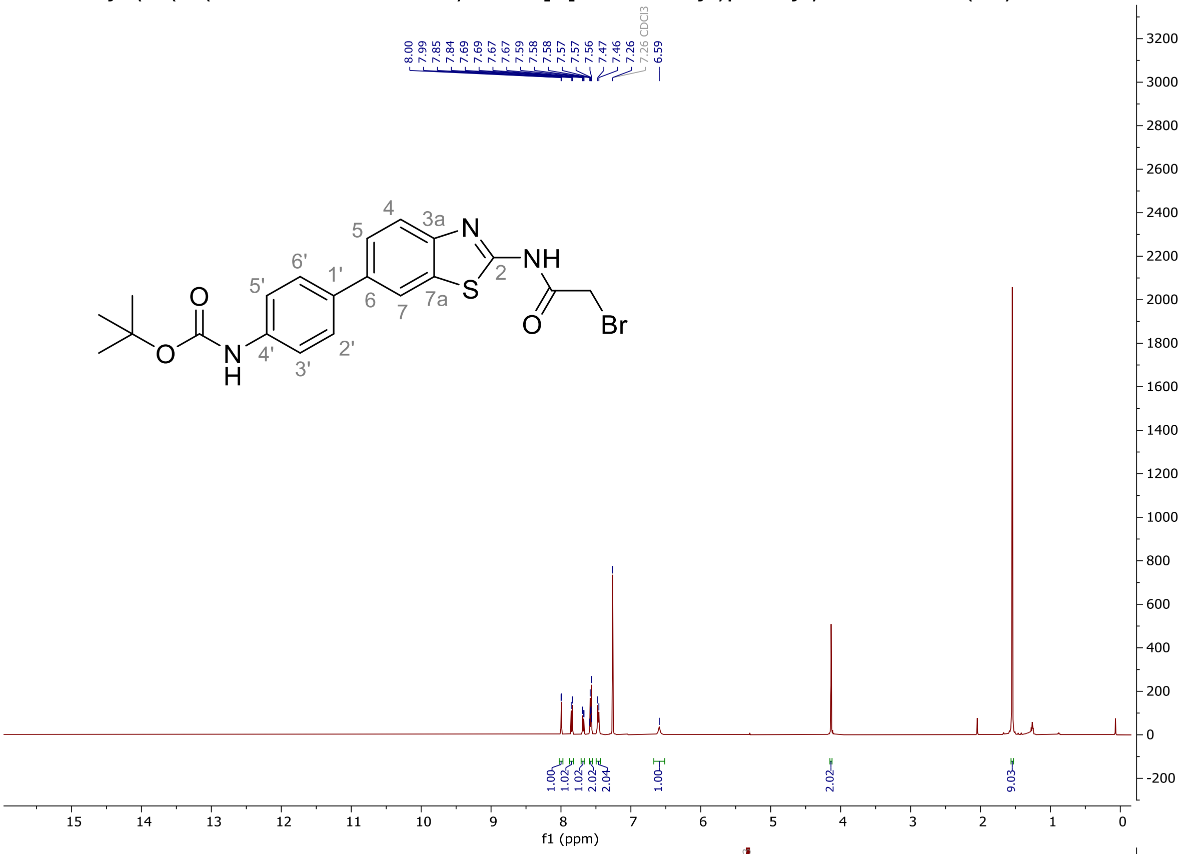

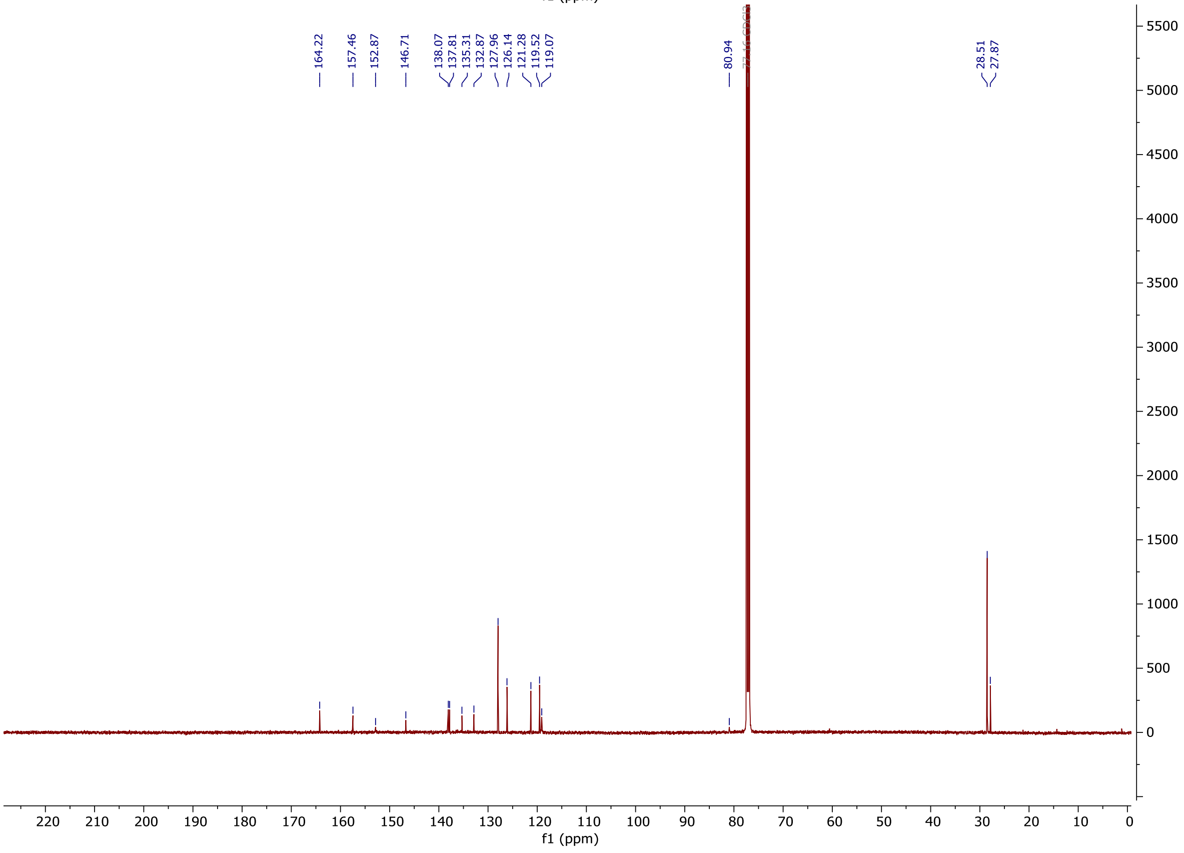

^{1}H and ^{13}C NMR spectra of
2-((4,6-Dimethylpyrimidin-2-yl)thio)-*N*-(6-(3-nitrophenyl)benzo[d]thiazol-2-yl)acetamide (**17**)

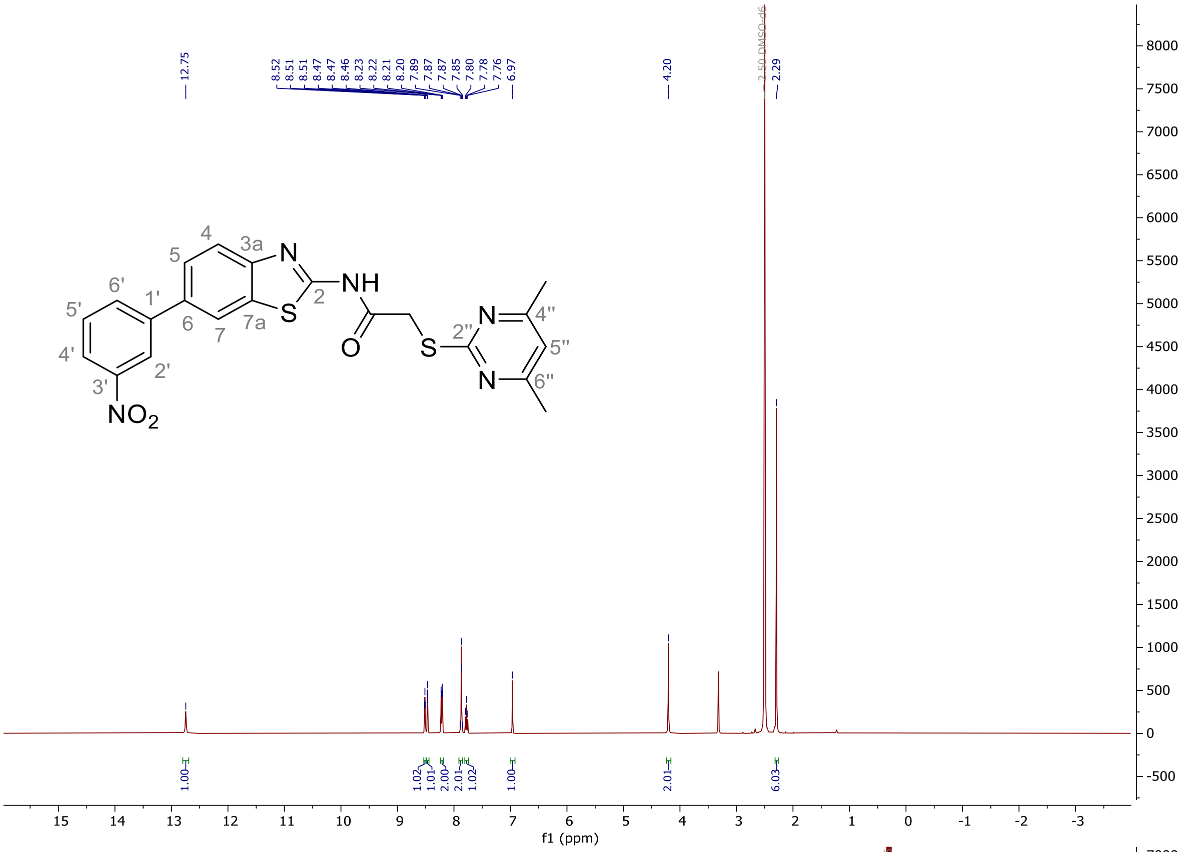

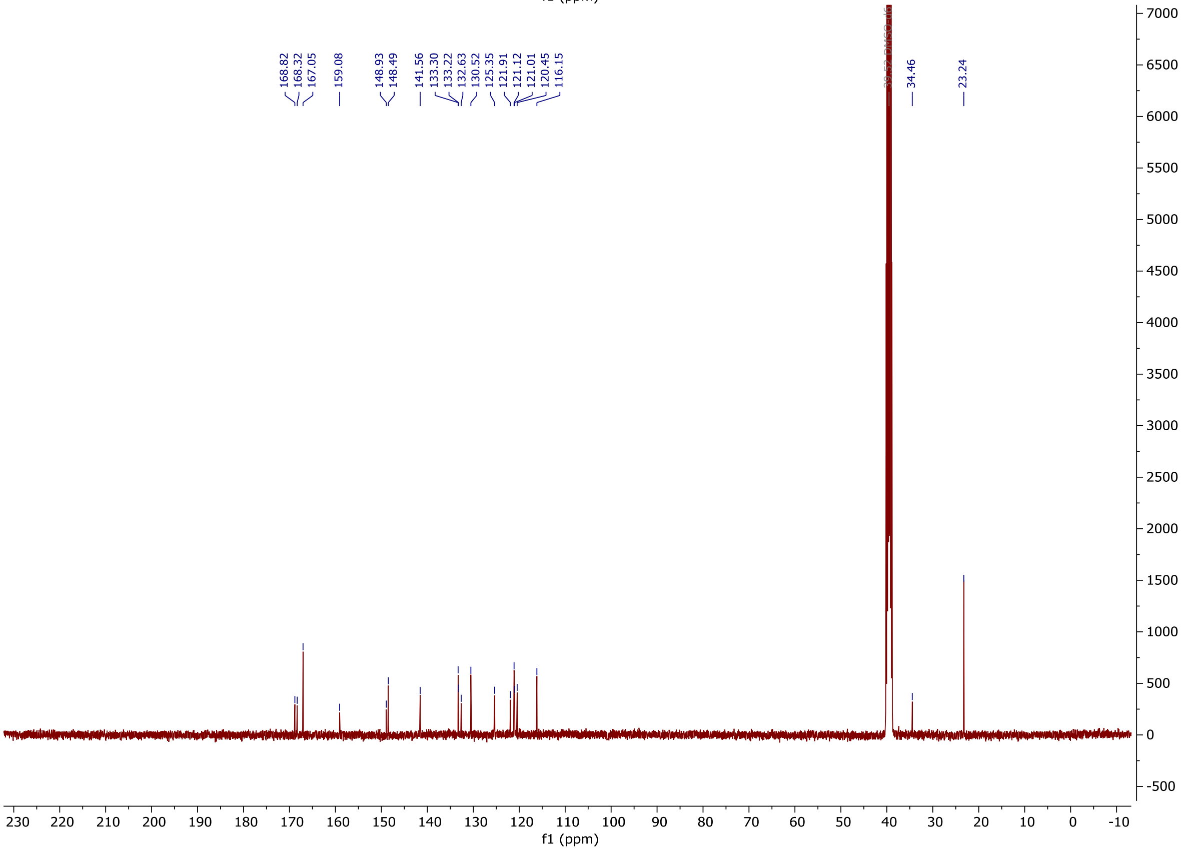

^{1}H and ^{13}C NMR spectra of
t-Butyl (4-(2-(2-((4,6-dimethylpyrimidin-2-yl)thio)acetamido)benzo[d]thiazol-6-yl)phenyl)carbamate (**18**)

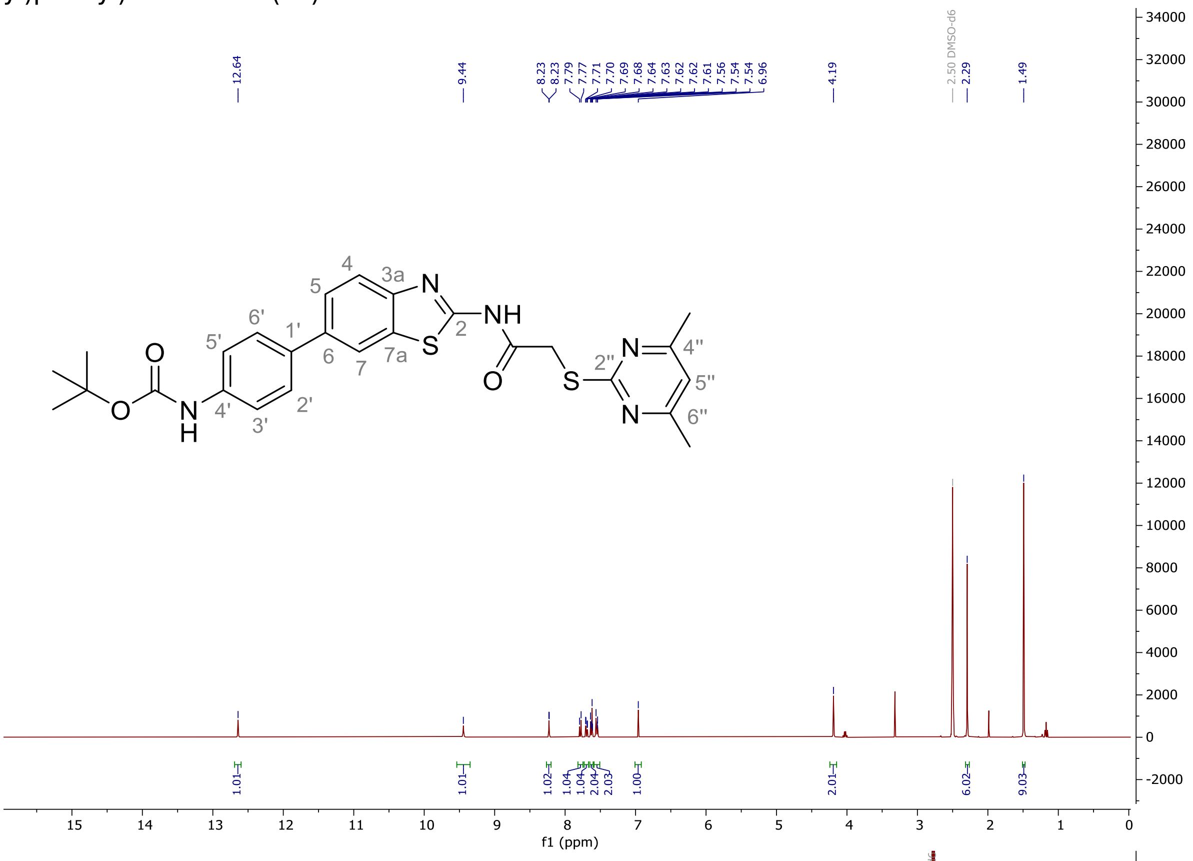

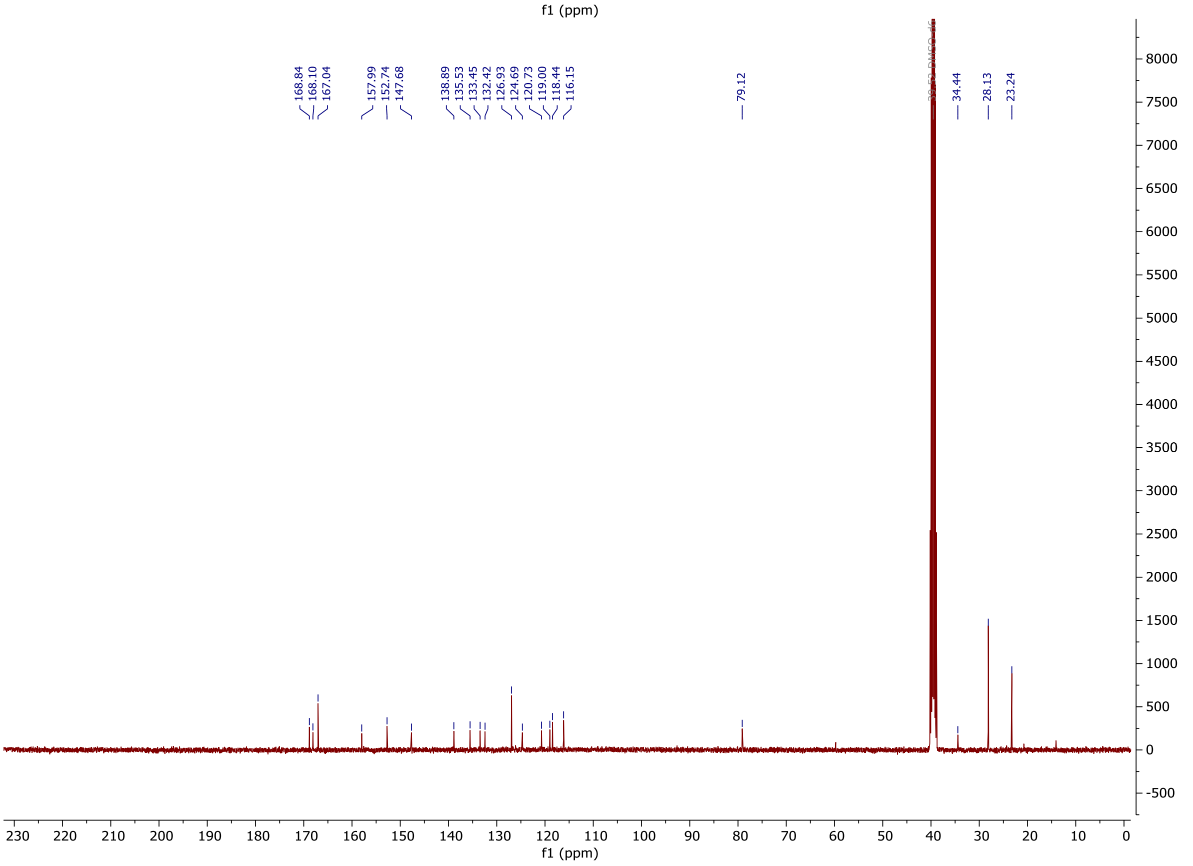

^{1}H and ^{13}C NMR spectra of
N-(6-(3-Aminophenyl)benzo[d]thiazol-2-yl)-2-((4,6-dimethylpyrimidin-2-yl)thio)acetamide (**19**)

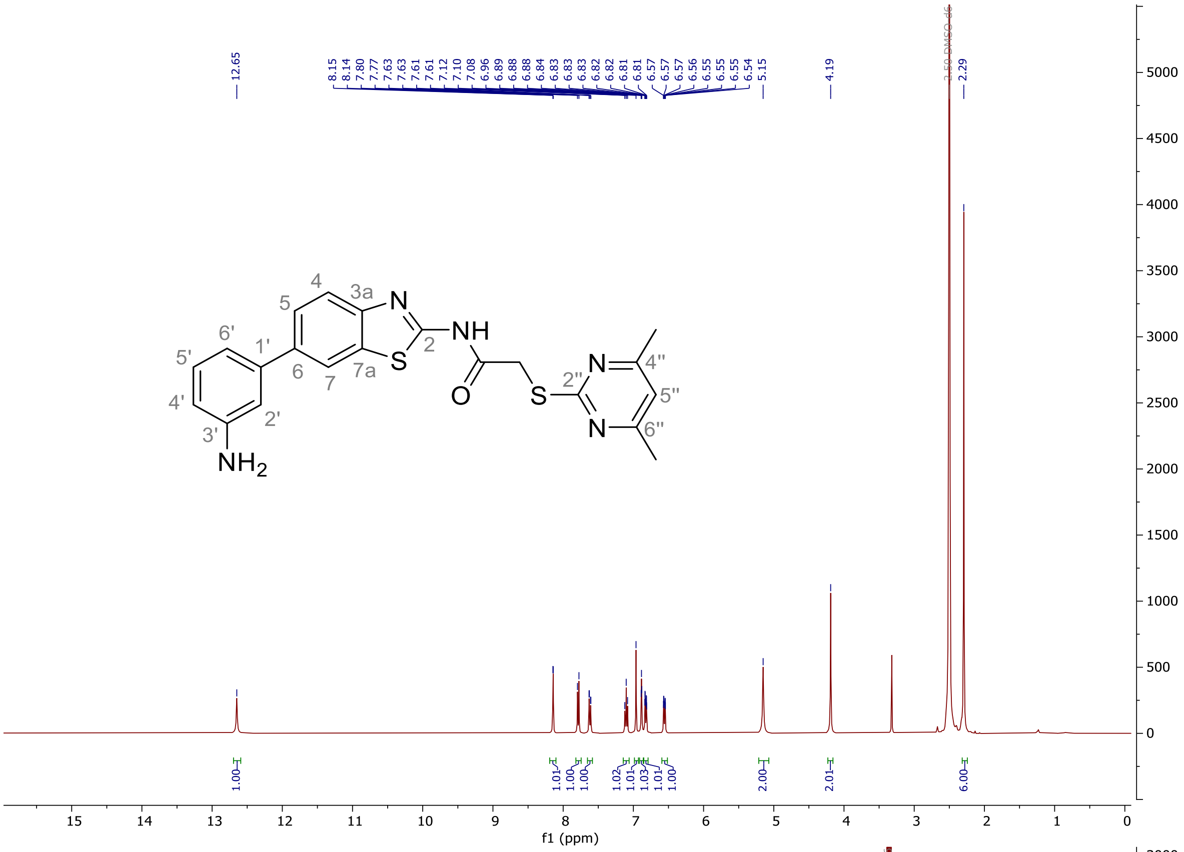

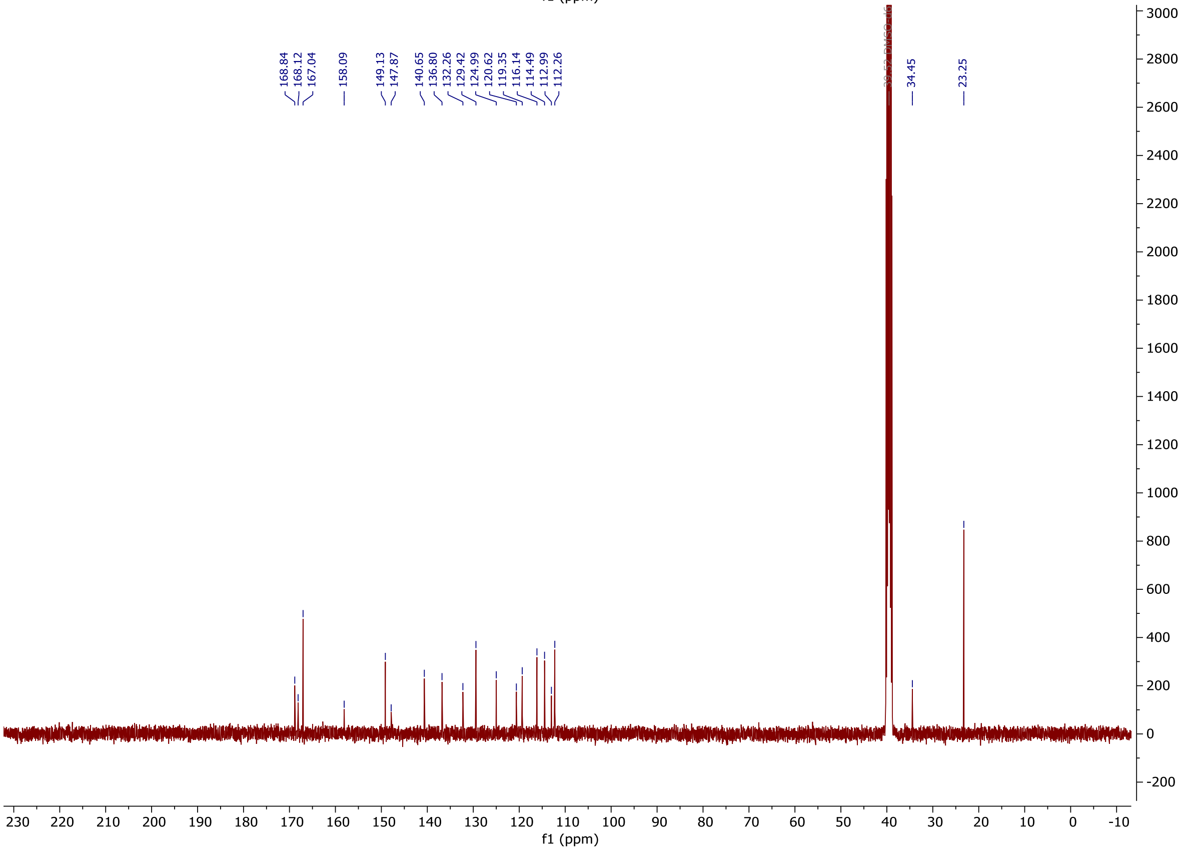

^{1}H and ^{13}C NMR spectra of

N-(6-(4-Aminophenyl)benzo[d]thiazol-2-yl)-2-((4,6-dimethylpyrimidin-2-yl)thio)acetamide (**20**)

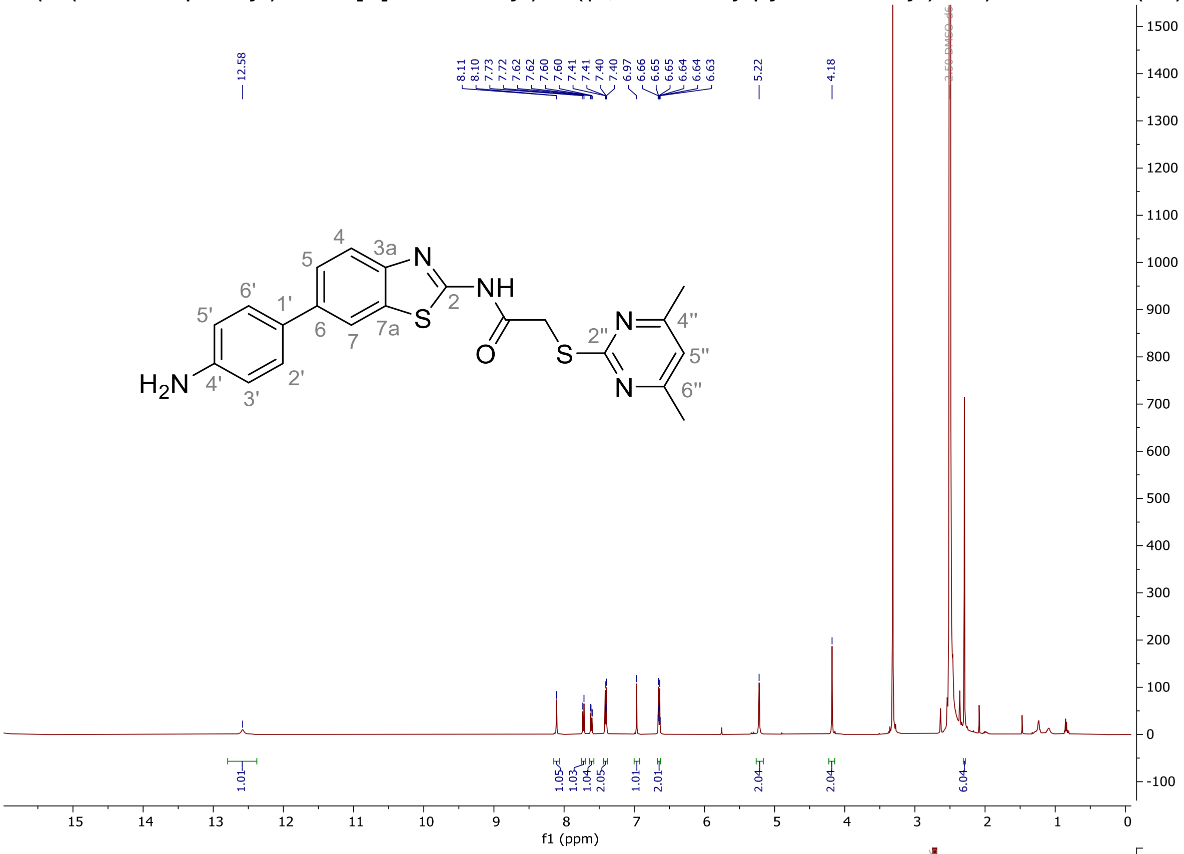

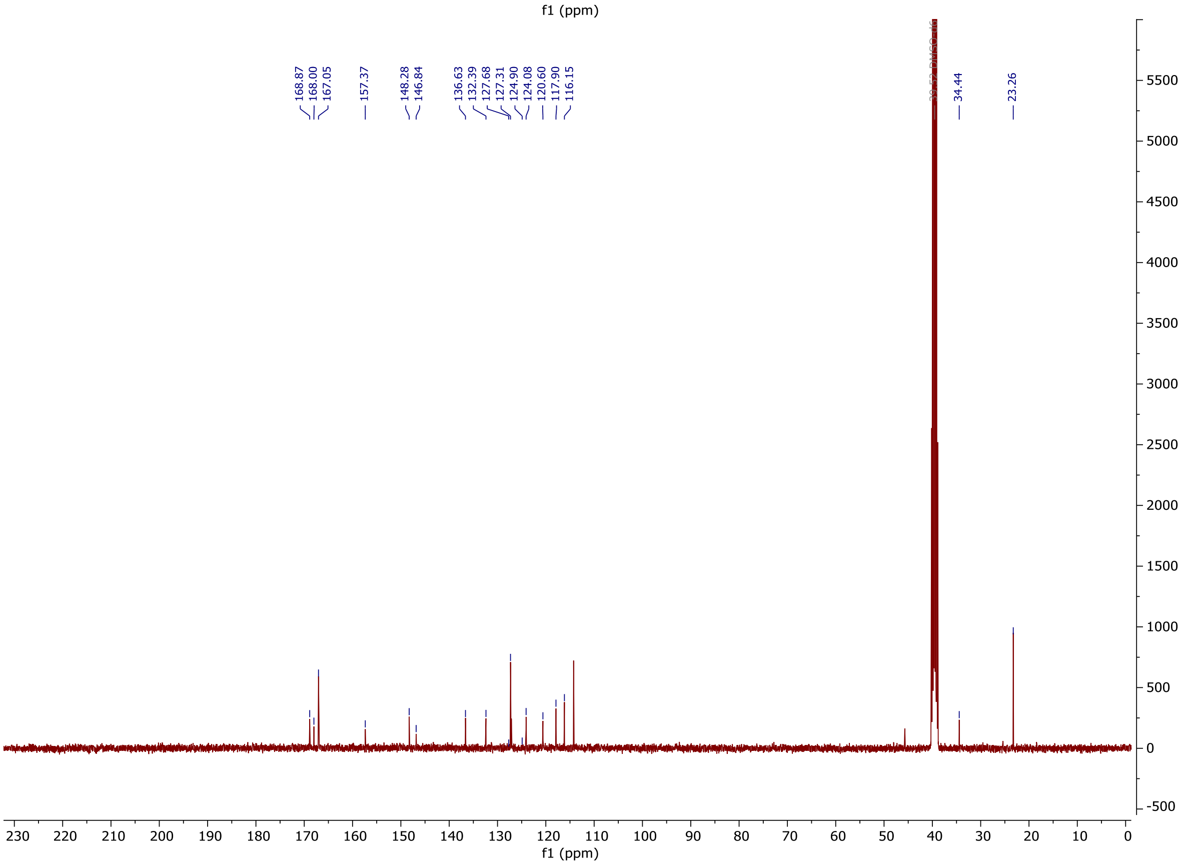

^1H and ^{13}C NMR spectra of

N-(6-(3-Acetamidophenyl)benzo[d]thiazol-2-yl)-2-((4,6-dimethylpyrimidin-2-yl)thio)acetamide (**FM94**)

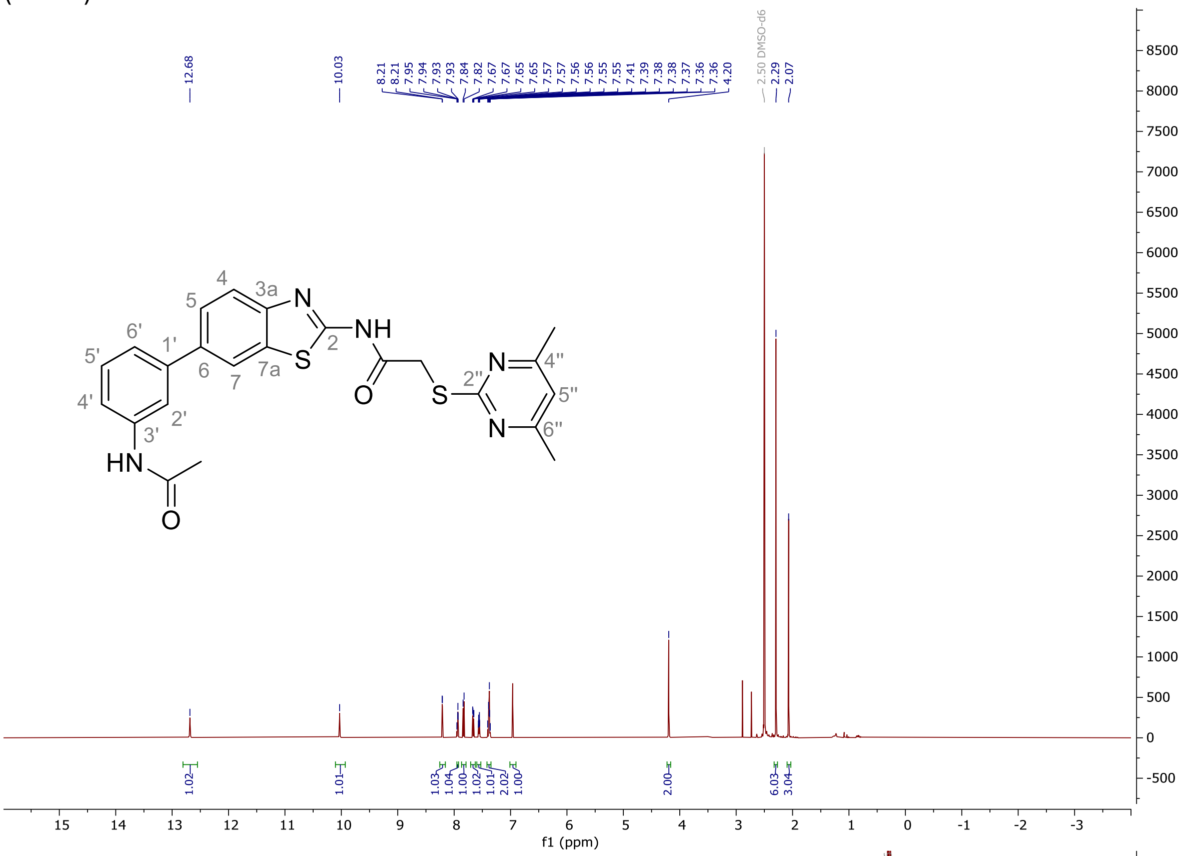

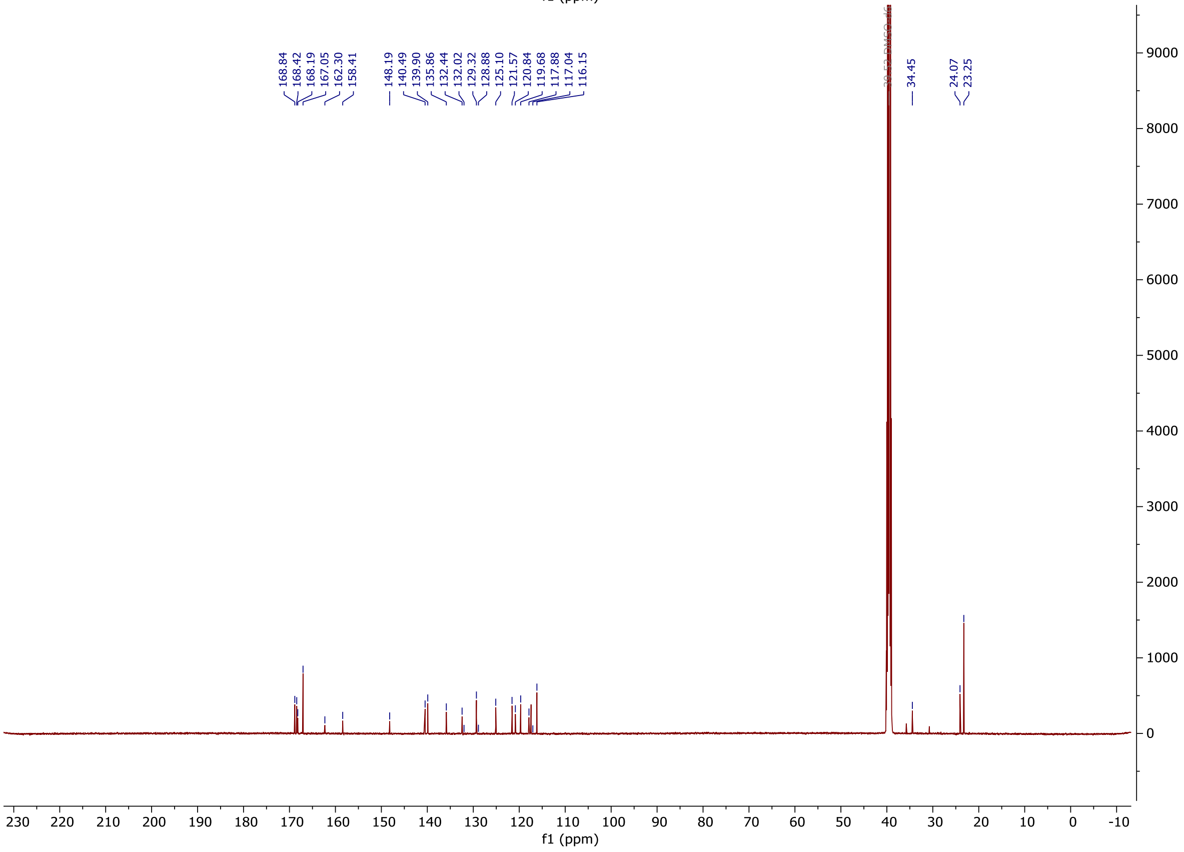

^{1}H and ^{13}C NMR spectra of
N-(3-(2-(2-((4,6-Dimethylpyrimidin-2-yl)thio)acetamido)benzo[d]thiazol-6-yl)phenyl)benzamide (**FM95**)

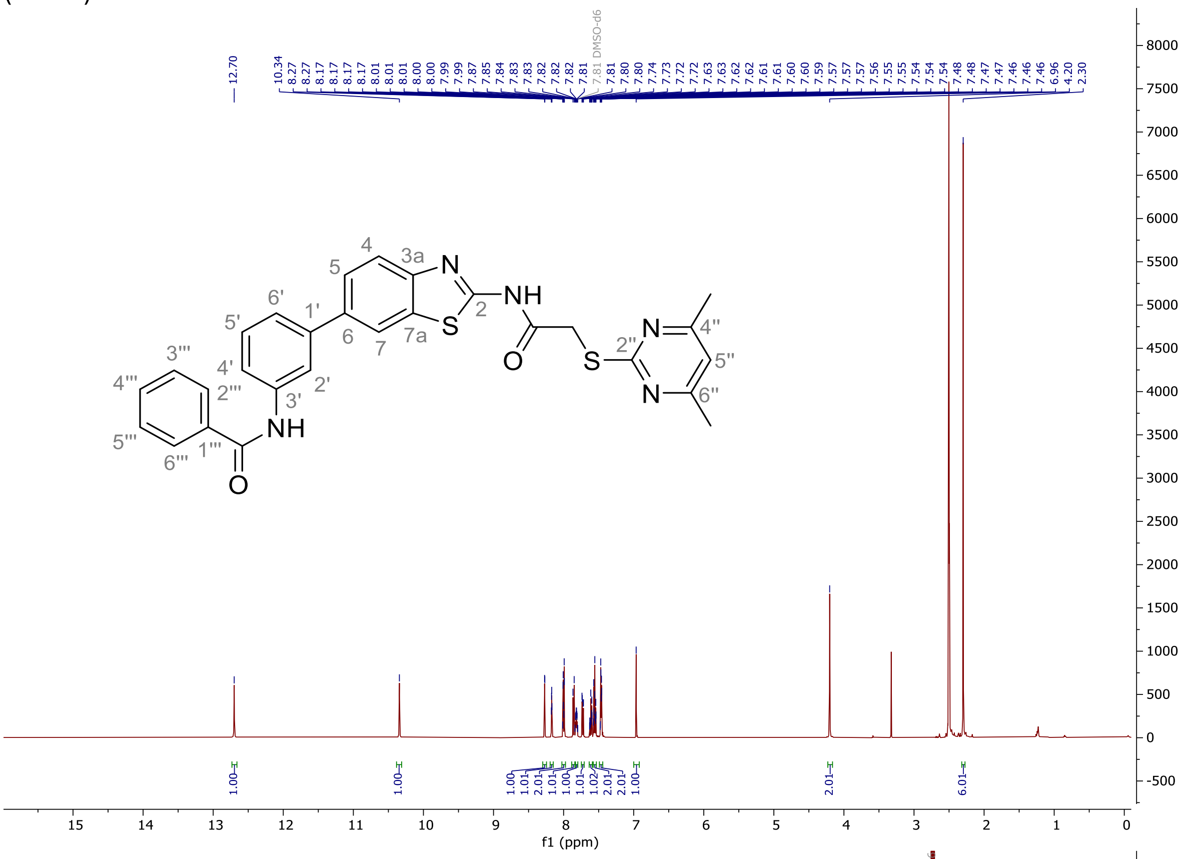

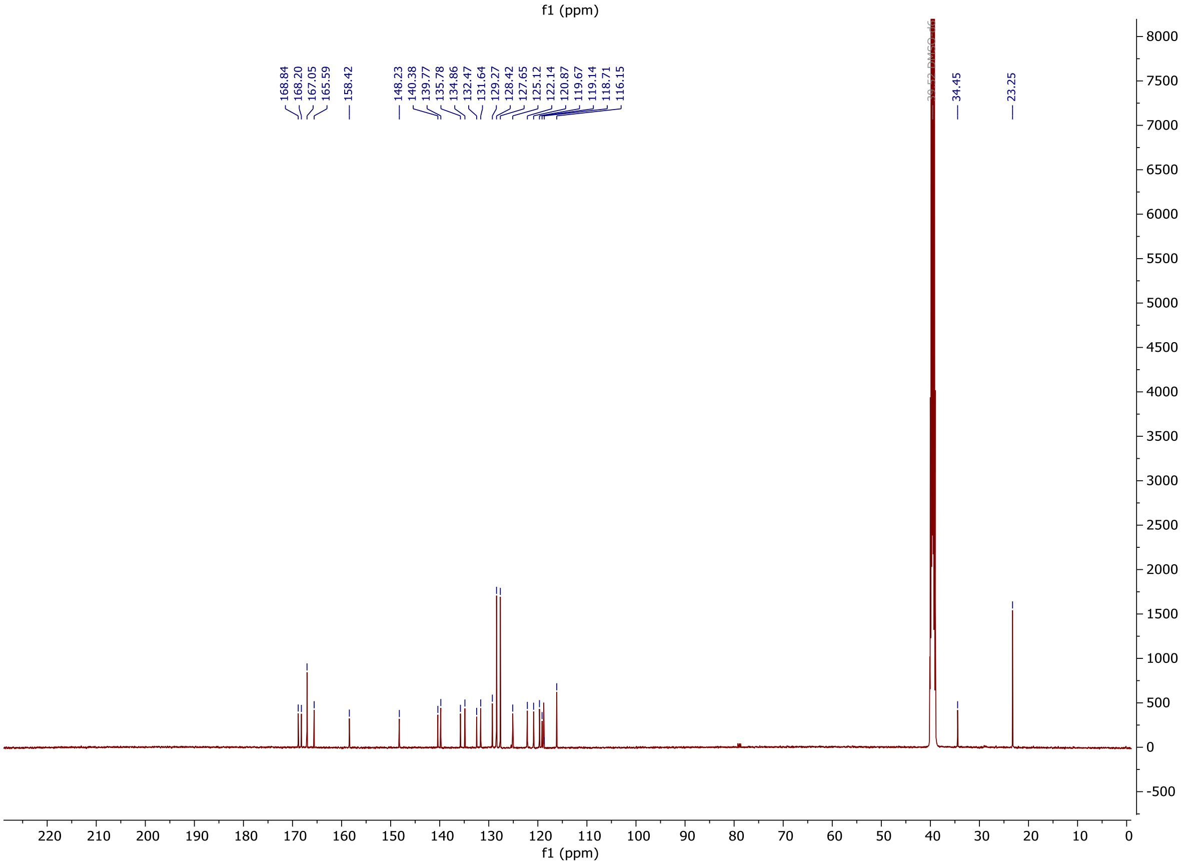

^{1}H and ^{13}C NMR spectra of

N-(3-(2-(2-((4,6-Dimethylpyrimidin-2-yl)thio)acetamido)benzo[d]thiazol-6-yl)phenyl)thiophene-2-carboxamide (**FM96**)

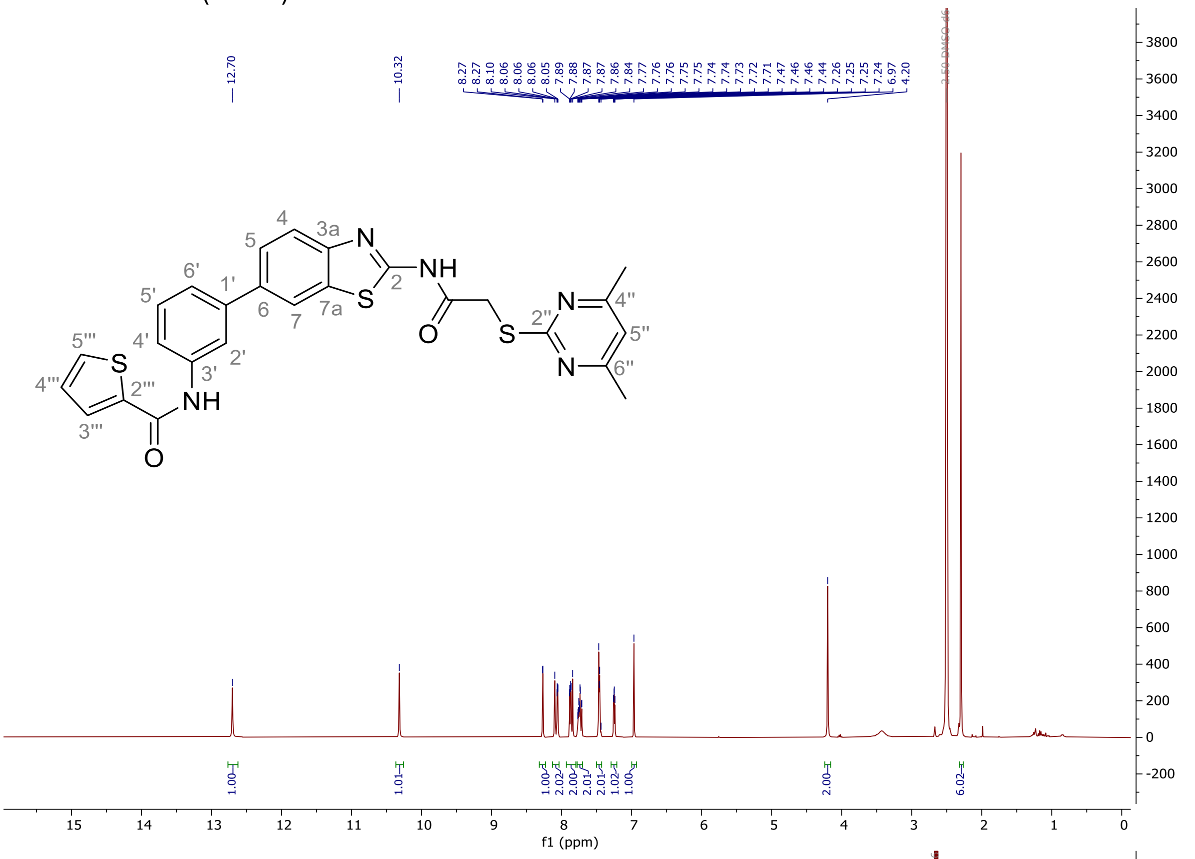

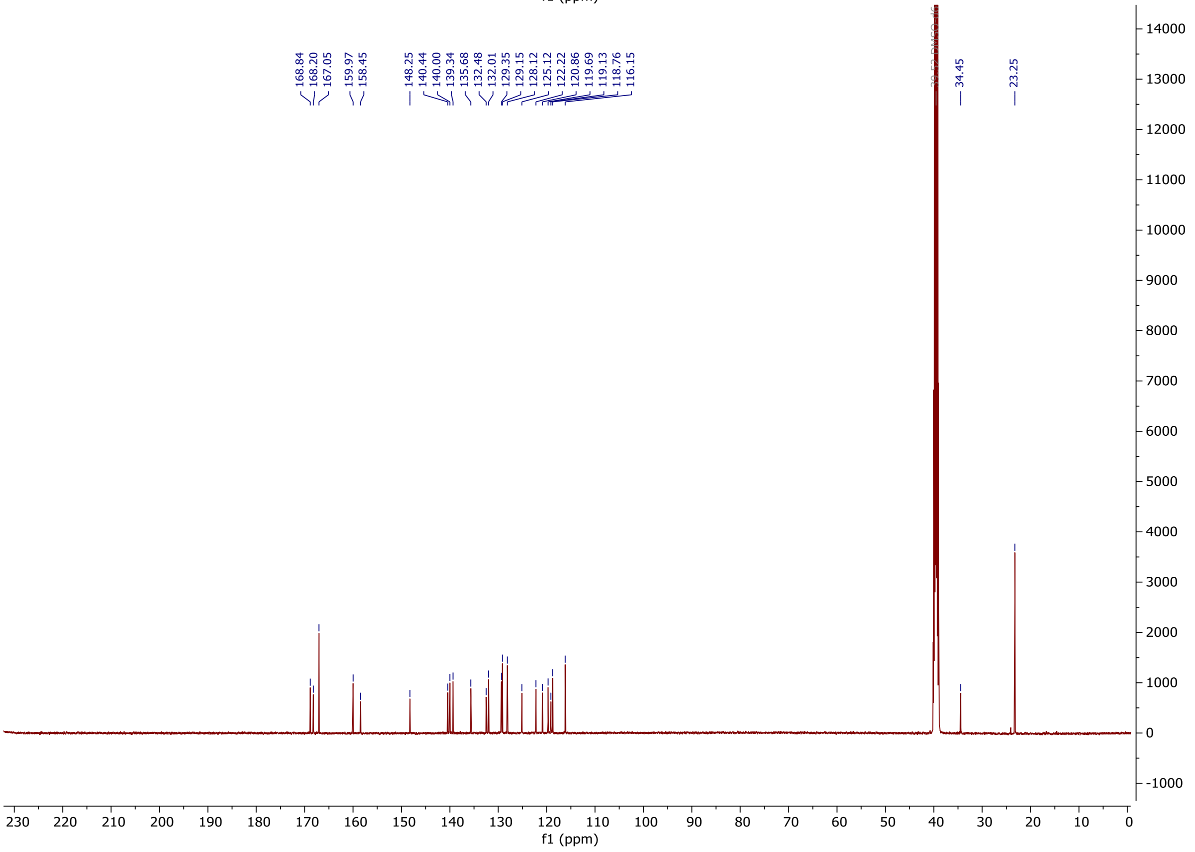

^{1}H and ^{13}C NMR spectra of 2-((4,6-Dimethylpyrimidin-2-yl)thio)-*N*-(6-(3-(phenylsulfonamido)phenyl)benzo[d]thiazol-2-yl)acetamide (**FM104**)

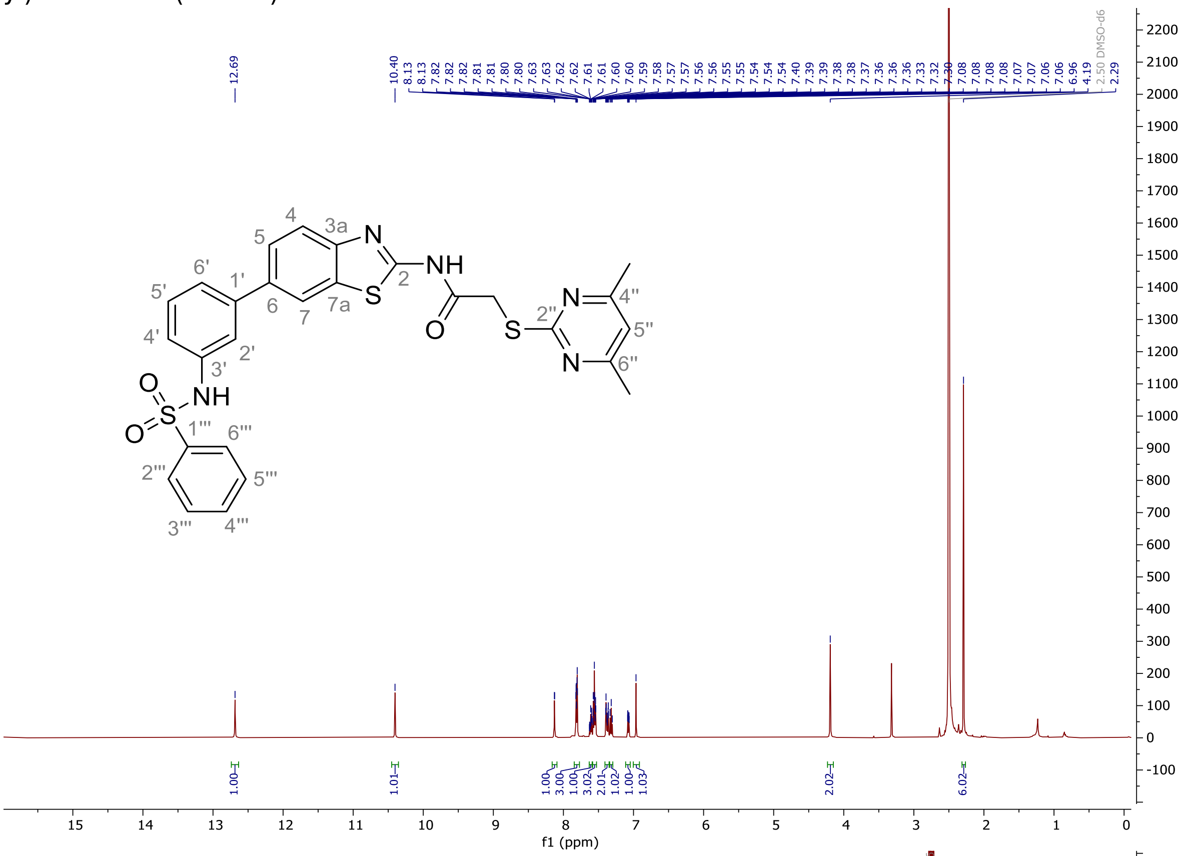

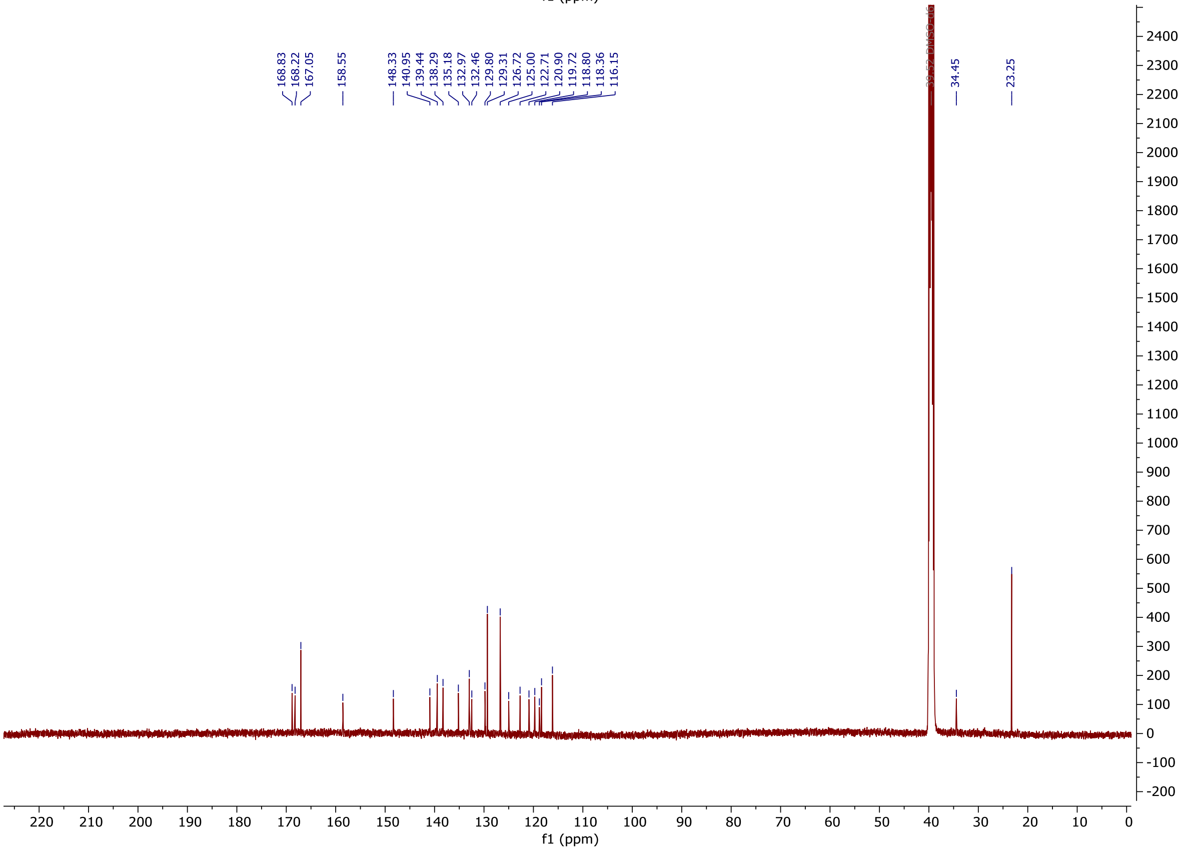

^{1}H and ^{13}C NMR spectra of
N-(3-(2-(2-((4,6-Dimethylpyrimidin-2-yl)thio)acetamido)benzo[d]thiazol-6-yl)phenyl)-5-methylthiophene-2-carboxamide (**FM108**)

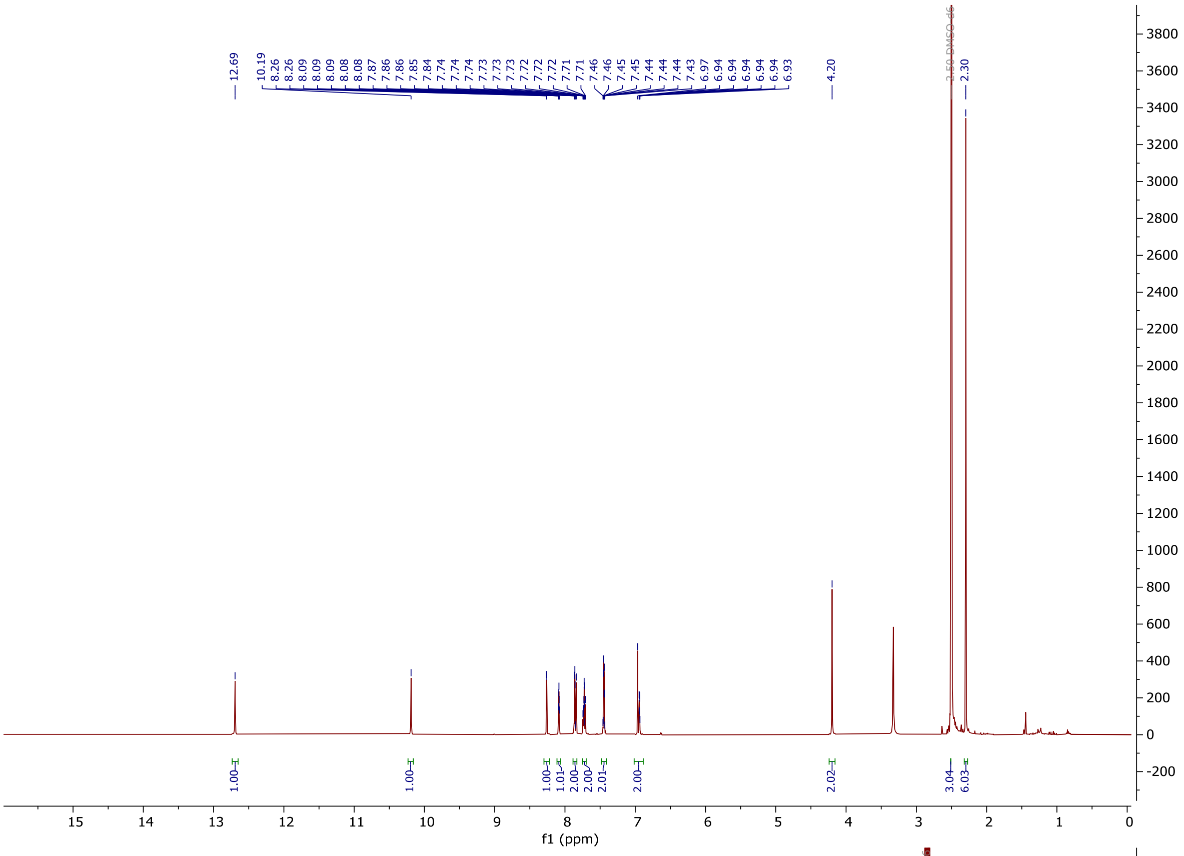

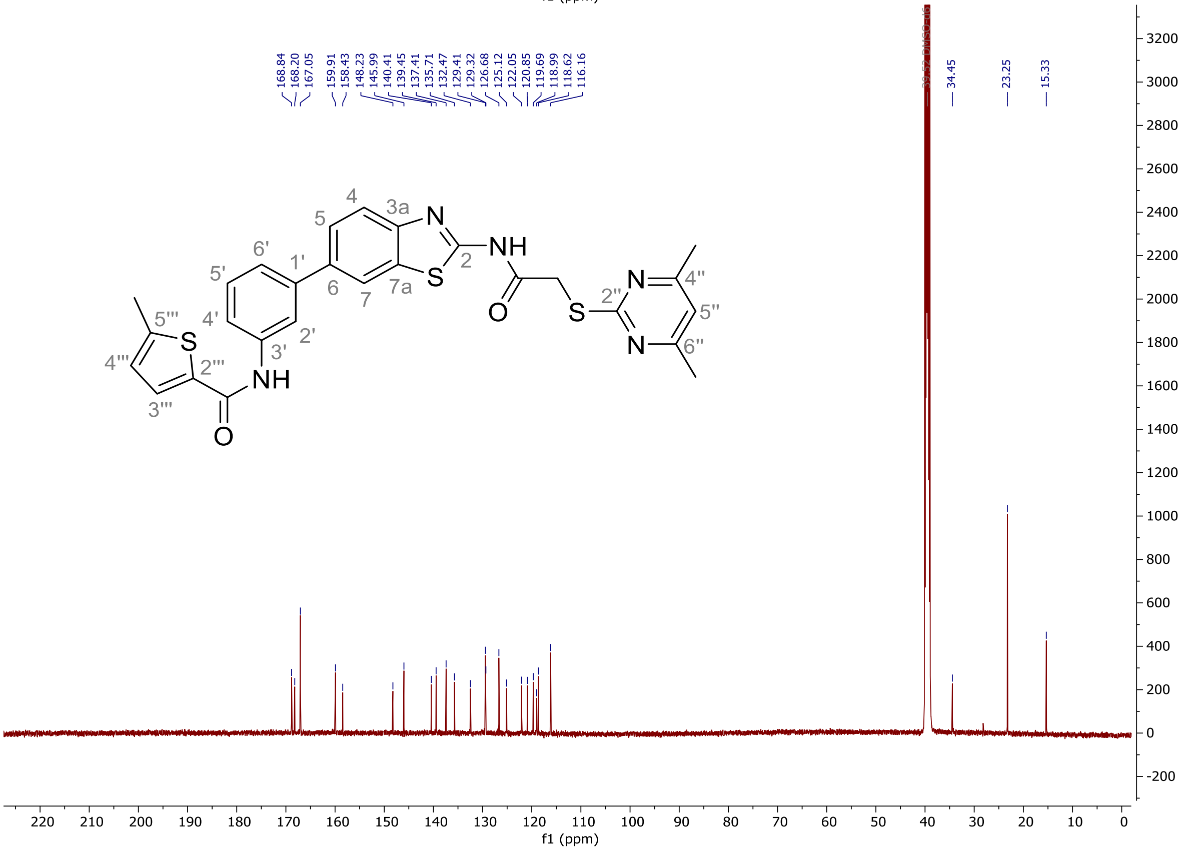

^{1}H and ^{13}C NMR spectra of

N-(4-(2-(2-((4,6-Dimethylpyrimidin-2-yl)thio)acetamido)benzo[d]thiazol-6-yl)phenyl)benzamide (**FM127**)

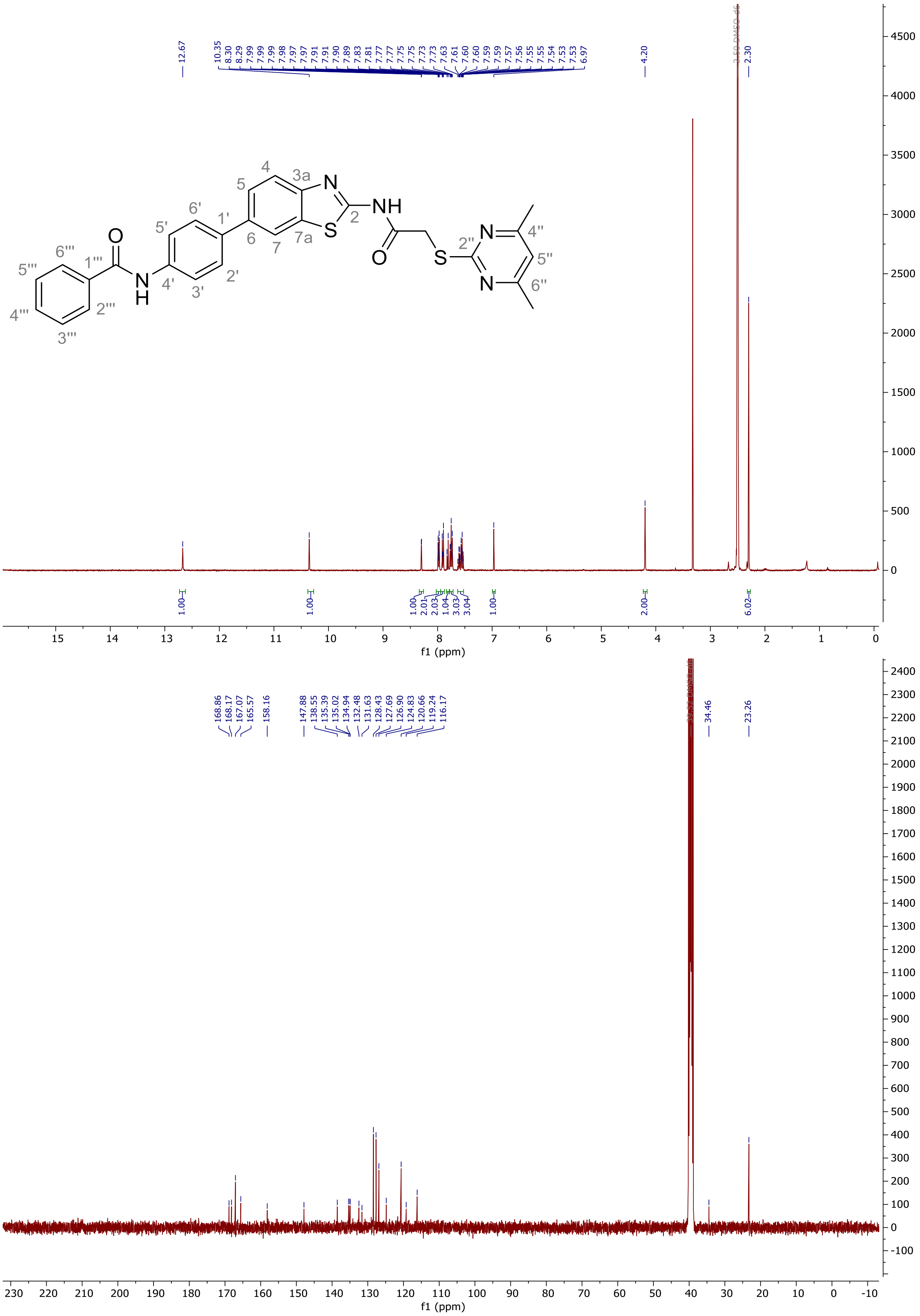

^{1}H and ^{13}C NMR spectra of
N-(6-(4-Acetamidophenyl)benzo[d]thiazol-2-yl)-2-((4,6-dimethylpyrimidin-2-yl)thio)acetamide (**FM128**)

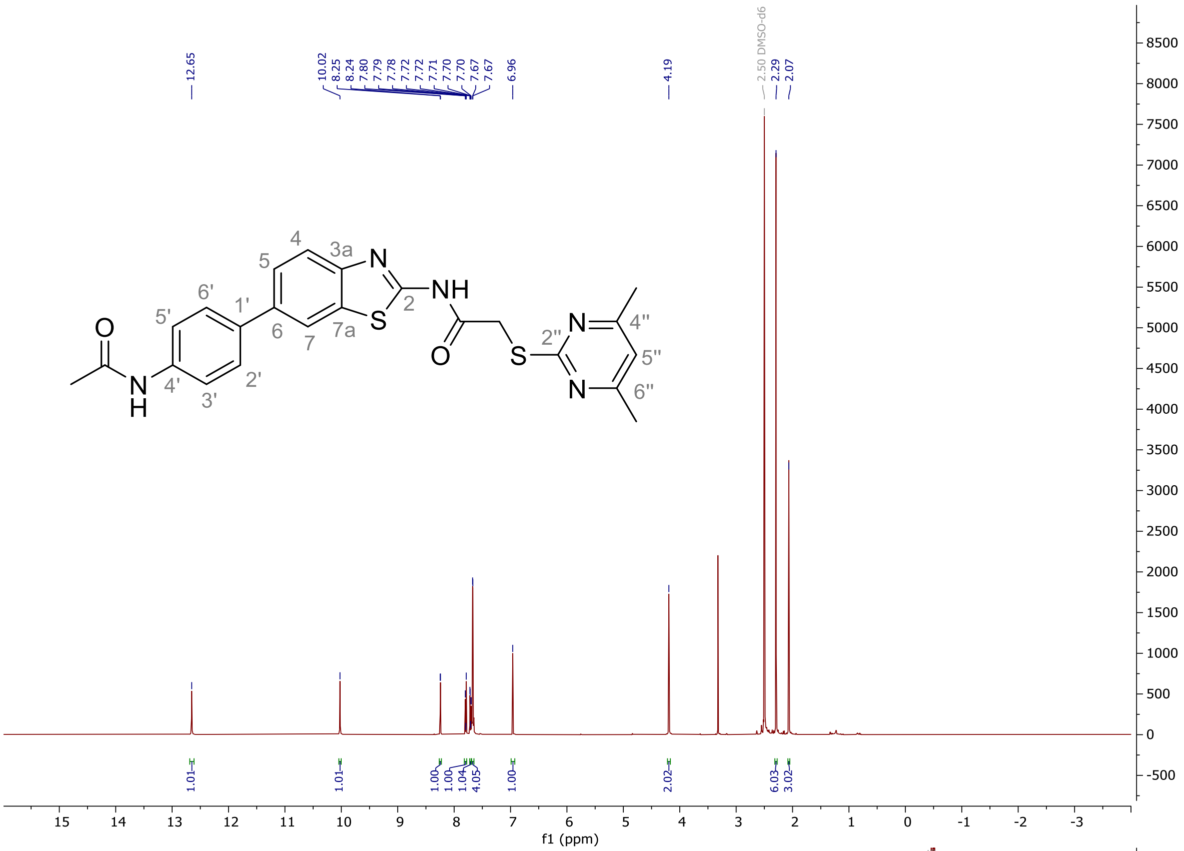

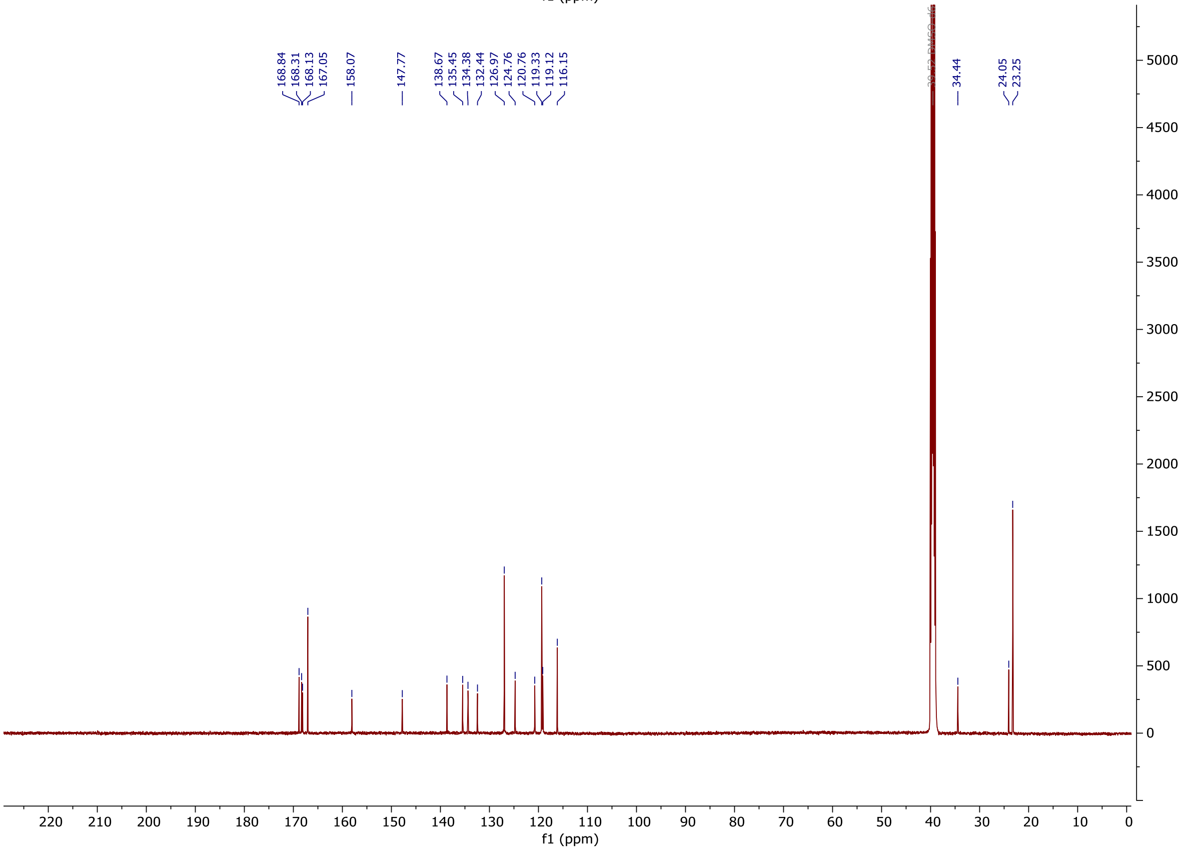

^{1}H and ^{13}C NMR spectra of
N-(4-(2-(2-((4,6-Dimethylpyrimidin-2-yl)thio)acetamido)benzo[d]thiazol-6-yl)phenyl)thiophene-2-carboxamide (**FM129**)

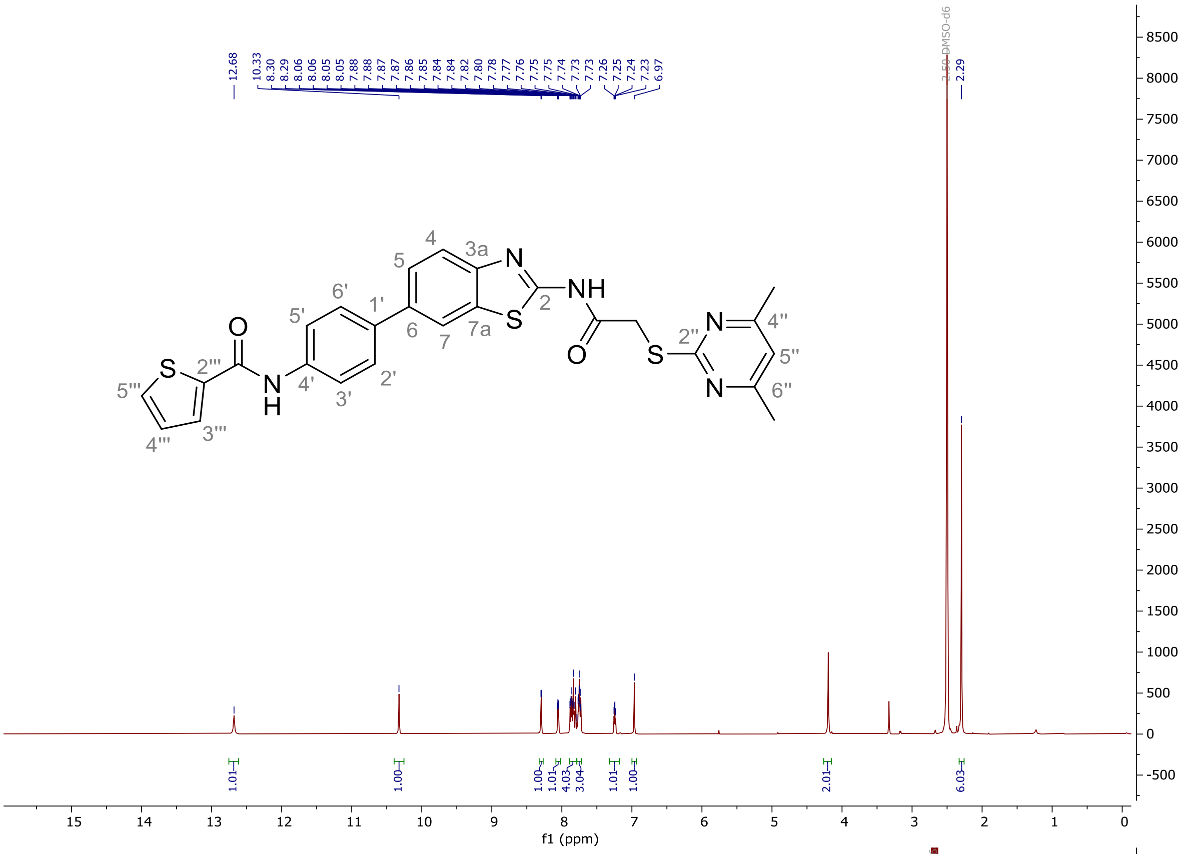

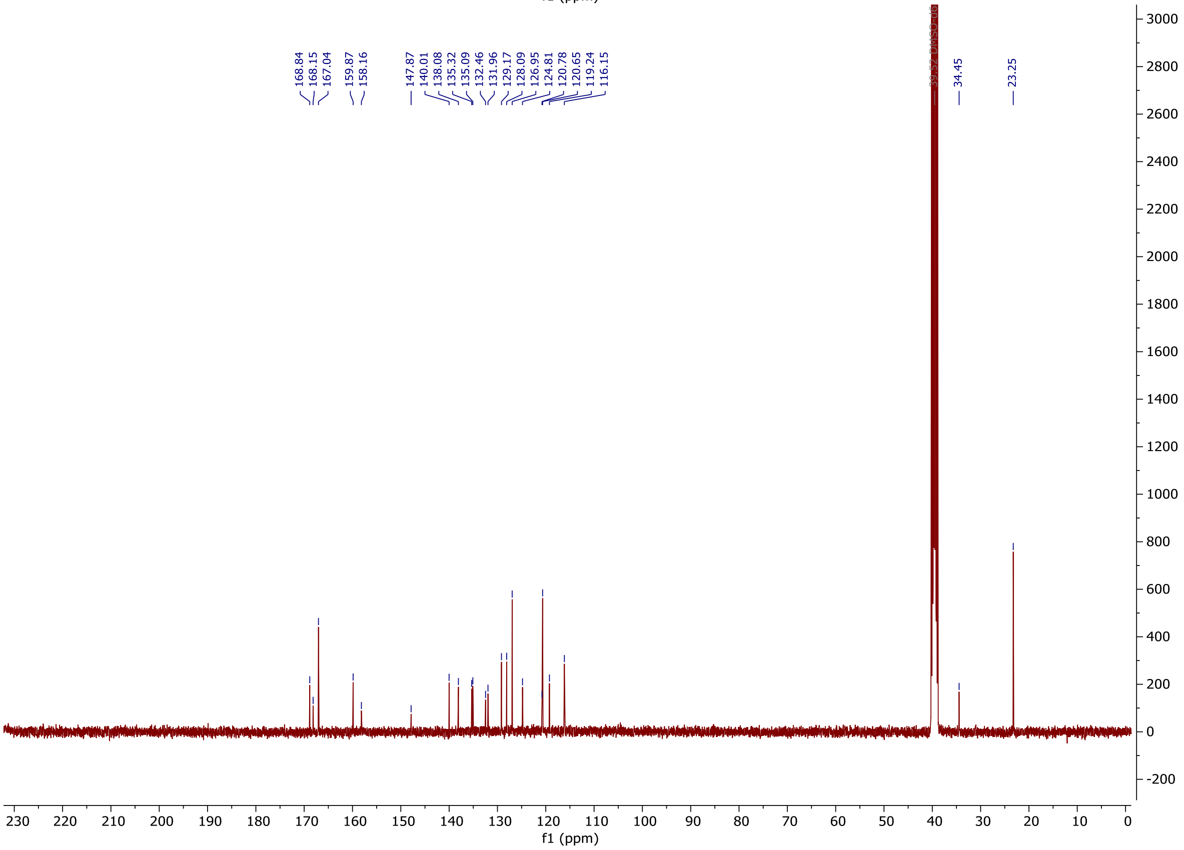

^{1}H and ^{13}C NMR spectra of 2-((4,6-Dimethylpyrimidin-2-yl)thio)-*N*-(6-(3-(phenylsulfonamido)phenyl)benzo[d]thiazol-2-yl)acetamide (**FM130**)

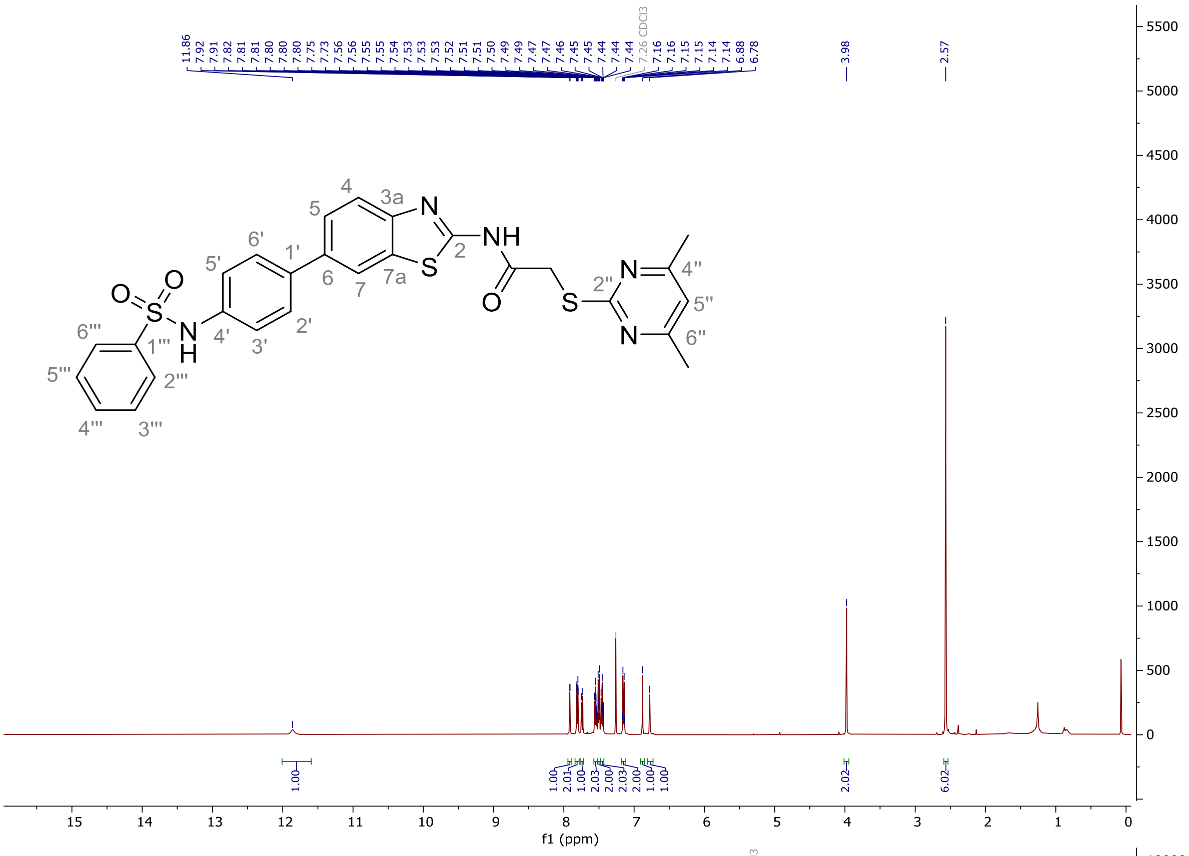

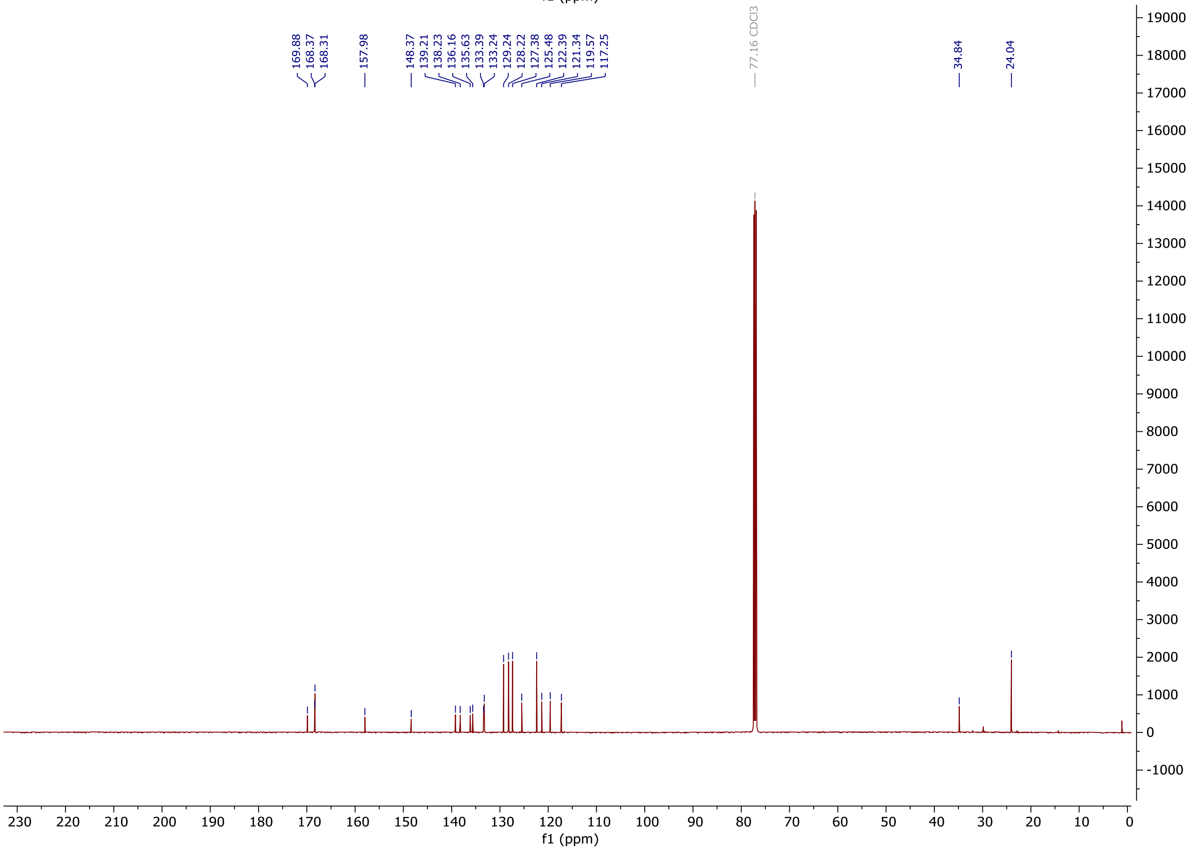

^{1}H and ^{13}C NMR spectra of
N-(4-(2-(2-((4,6-Dimethylpyrimidin-2-yl)thio)acetamido)benzo[d]thiazol-6-yl)phenyl)-5-methylthiophene-2-carboxamide (**FM131**)

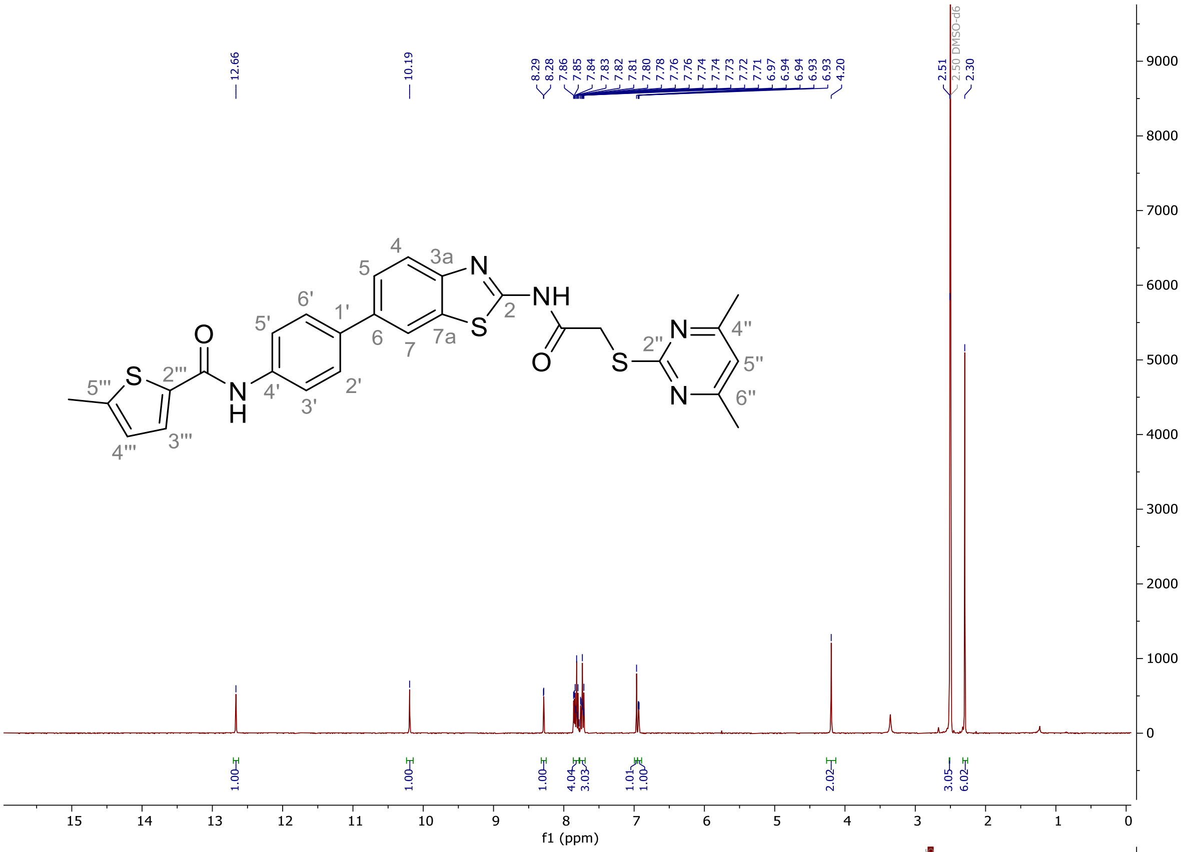

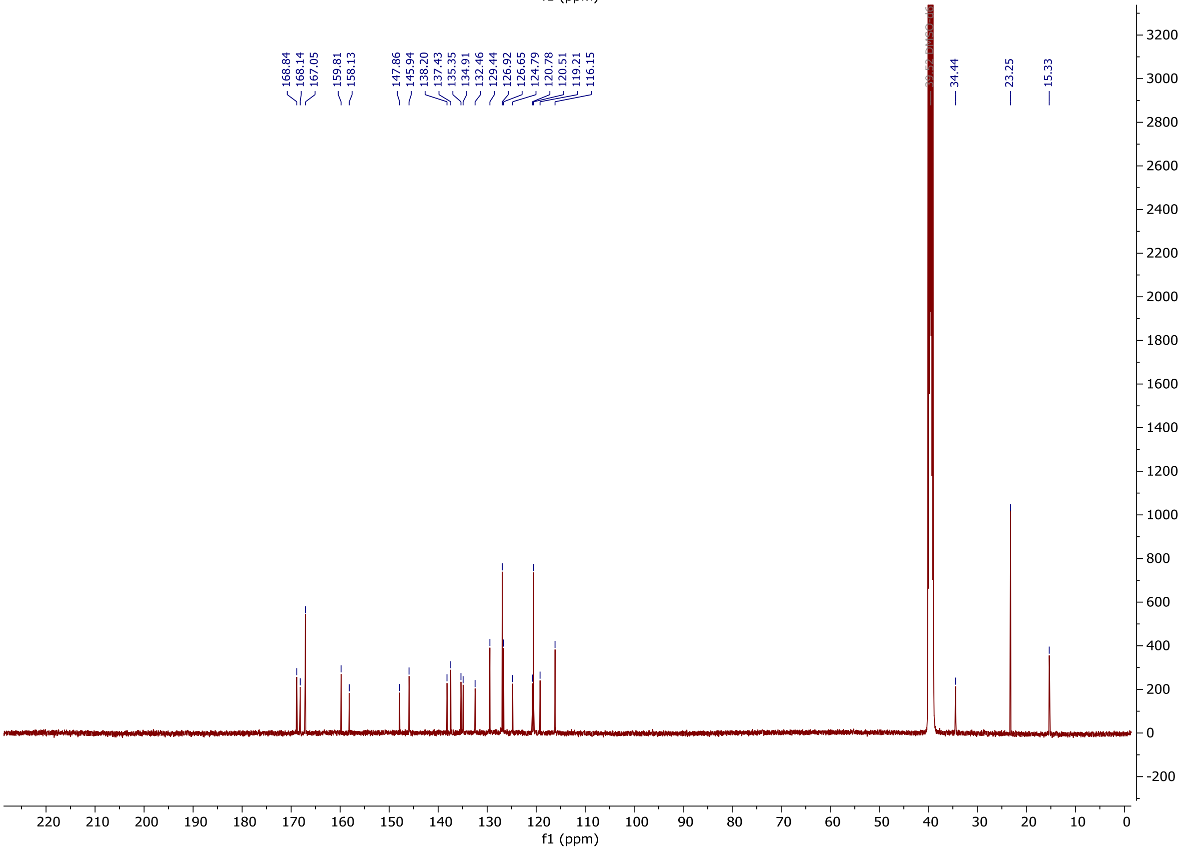

HPLC chromatogram of *N*-(7-(4-Acetamidophenyl)naphthalen-2-yl)-2-((4,6-dimethylpyrimidin-2-yl)thio)acetamide (**FM26**)

Chromatogram and Results

Injection Details

Injection Name:	fm26	*Run Time (min):*	9,99
Vial Number:	Vial:4	*Injection Volume:*	10,00 µL
Injection Type:	Unknown	*Wavelength:*	254 nm
		Wavelength:	210 nm
Instrument Method:	Trudel 70 ACN 30 Wasser	*Flow rate:*	1,0 ml/min
Column:	Zorbax Eclipse Plus C18 4,6x 150mm, 5µm	*Column Temperatur:*	30 °C
Injection Date/Time:	29.Apr.20 13:47		
		Pump Channel B:	70 ACN 30 Wasser

Chromatogram

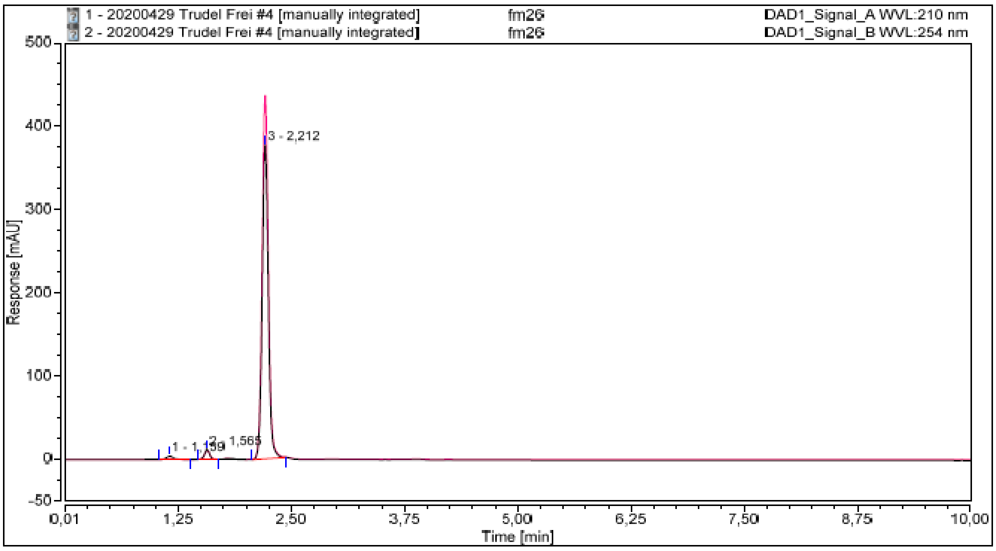

DAD1_Signal_A

No.	Retention Time min	Area mAU*min	Relative Area %
1	1,159	0,477	n.a.
2	1,565	0,837	2
3	2,212	29,351	98
Total:		30,666	100

DAD1_Signal_B

No.	Retention Time min	Area mAU*min	Relative Area %
1	1,565	0,812	2
2	2,212	34,453	98
Total:		35,265	100

HPLC chromatogram of *N*-(7-(3-Acetamidophenyl)naphthalen-2-yl)-2-((4,6-dimethylpyrimidin-2-yl)thio)acetamide (**FM46**)

Chromatogram and Results

Injection Details

Injection Name:	fm46	*Run Time (min):*	9,99
Vial Number:	Vial:8	*Injection Volume:*	10,00 µL
Injection Type:	Unknown	*Wavelength:*	254 nm
		Wavelength:	210 nm
Instrument Method:	Trudel 70 ACN 30 Wasser	*Flow rate:*	1,0 ml/min
Column:	Zorbax Eclipse Plus C18 4,6x 150mm, 5µm	*Column Temperatur:*	30 °C
Injection Date/Time:	29.Apr.20 14:30		
		Pump Channel B:	70 ACN 30 Wasser

Chromatogram

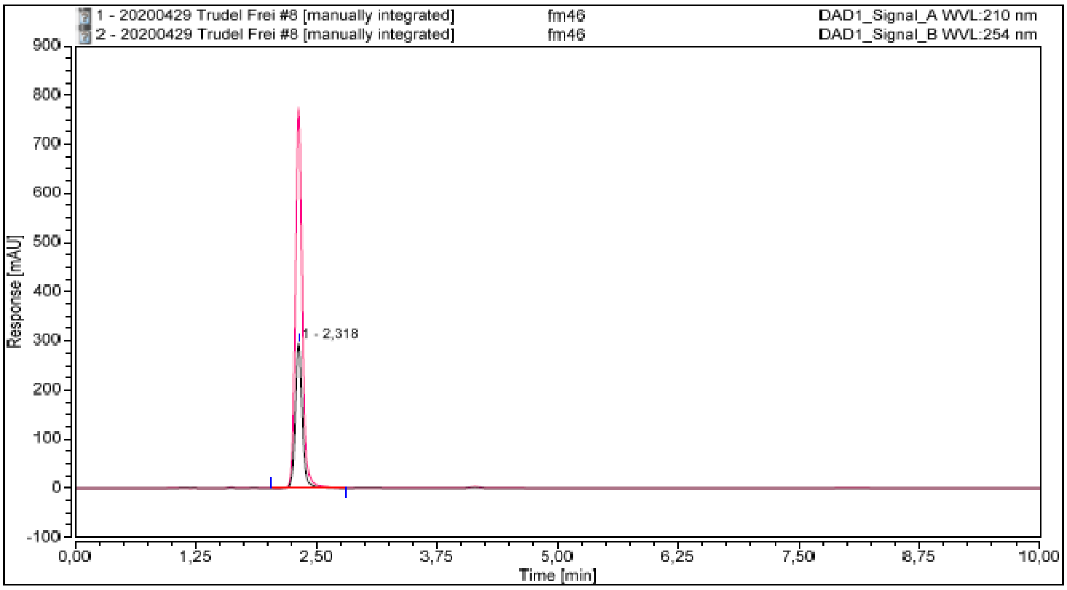

DAD1_Signal_A

No.	Retention Time min	Area mAU*min	Relative Area %
1	2,318	24,486	100
Total:		24,486	100

DAD1_Signal_B

No.	Retention Time min	Area mAU*min	Relative Area %
1	2,318	63,614	100
Total:		63,614	100

HPLC chromatogram of 2-((4,6-Dimethylpyrimidin-2-yl)thio)-N-(7-(3-(phenylsulfonamido)phenyl)naphthalen-2-yl)acetamide (**FM47**)

Page 1 of 1

Chromatogram and Results

Injection Details

Injection Name:	fm-47 (2)	Run Time (min):	10,00
Vial Number:	Vial:11	Injection Volume:	10,00 µL
Injection Type:	Unknown		nm
		Wavelength:	210 nm
Instrument Method:	Horst 70 ACN 30 Wasser 210nm	Flow rate:	1,0 ml/min
Column:	Zorbax Eclipse Plus C18 4,6x 150mm, 5µm	Column Temperatur:	50 °C
Injection Date/Time:	04.Jun.20 08:42		
		Pump Channel B:	100,0 70 ACN 30 Wasser

Chromatogram

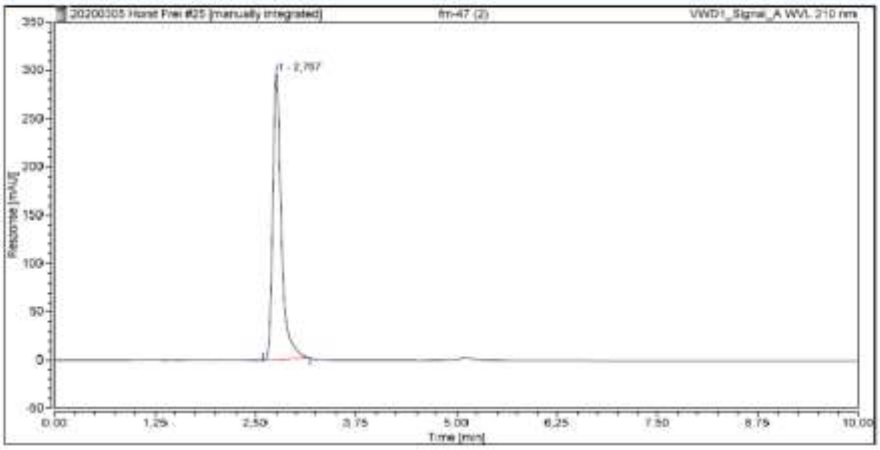

VWD1_Signal_A

No.	Retention Time min	Area mAU*min	Relative Area %
1	2,757	36,807	100,00
Total:		36,807	100

Page 1 of 1

Chromatogram and Results

Injection Details

Injection Name:	fm-47 (2)	Run Time (min):	9,99
Vial Number:	Vial:11	Injection Volume:	10,00 µL
Injection Type:	Unknown		nm
		Wavelength:	254 nm
Instrument Method:	Horst 70 ACN 30 Wasser 254nm	Flow rate:	1,0 ml/min
Column:	Zorbax Eclipse Plus C18 4,6x 150mm, 5µm	Column Temperatur:	50 °C
Injection Date/Time:	04.Jun.20 08:53		
		Pump Channel B:	100,0 70 ACN 30 Wasser

Chromatogram

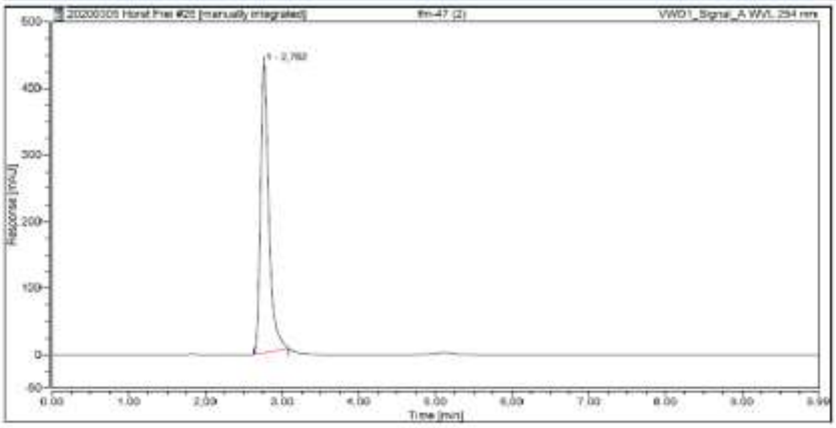

VWD1_Signal_A

No.	Retention Time min	Area mAU*min	Relative Area %
1	2,762	54,838	100,00
Total:		54,838	100

HPLC chromatogram of N-(3-(7-(2-((4,6-Dimethylpyrimidin-2-yl)thio)acetamido)naphthalen-2-yl)phenyl)thiophene-2-carboxamide (**FM48**)

Chromatogram and Results

Injection Details

Injection Name:	**fm48**	*Run Time (min):*	**9,99**
Vial Number:	**Vial:10**	*Injection Volume:*	**10,00 µL**
Injection Type:	**Unknown**	*Wavelength:*	**254 nm**
		Wavelength:	**210 nm**
Instrument Method:	**Trudel 70 ACN 30 Wasser**	*Flow rate:*	**1,0 ml/min**
Column:	**Zorbax Eclipse Plus C18 4,6x 150mm, 5µm**	*Column Temperatur:*	**30 °C**
Injection Date/Time:	**29.Apr.20 14:51**		
		Pump Channel B:	**70 ACN 30 Wasser**

Chromatogram

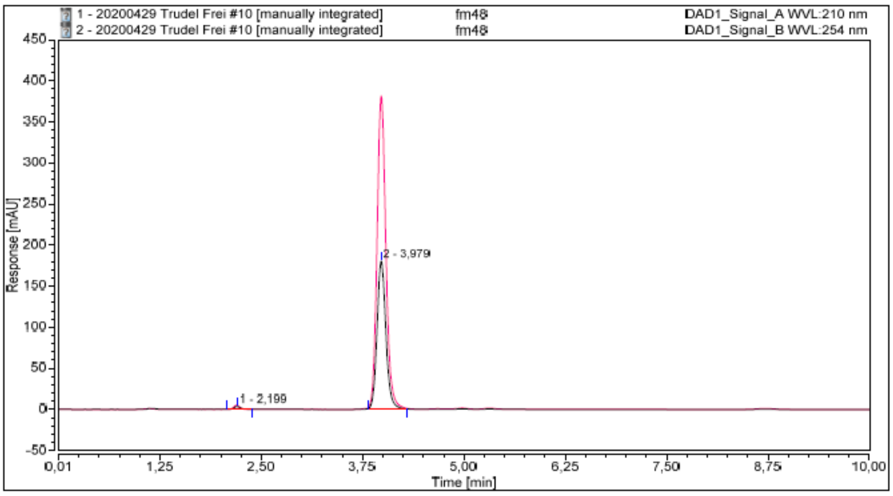

DAD1_Signal_A

No.	Retention Time min	Area mAU*min	Relative Area %
1	2,199	0,313	0,90
2	3,979	22,569	99,10
**Total:**		**22,882**	**100**

DAD1_Signal_B

No.	Retention Time min	Area mAU*min	Relative Area %
1	2,192	0,441	1
2	3,979	48,495	99
**Total:**		**48,936**	**100**

HPLC chromatogram of *N*-(4-(7-(2-((4,6-Dimethylpyrimidin-2-yl)thio)acetamido)naphthalen-2-yl)phenyl)thiophene-2-carboxamide (**FM50**)

Chromatogram and Results

Injection Details

Injection Name:	fm50 (2)	*Run Time (min):*	9,99
Vial Number:	Vial:3	*Injection Volume:*	10,00 µL
Injection Type:	Unknown	*Wavelength:*	254 nm
		Wavelength:	210 nm
Instrument Method:	Trudel 70 ACN 30 Wasser	*Flow rate:*	1,0 ml/min
Column:	Zorbax Eclipse Plus C18 4,6x 150mm, 5µm	*Column Temperatur:*	30 °C
Injection Date/Time:	29.Apr.20 13:36		
		Pump Channel B:	70 ACN 30 Wasser

Chromatogram

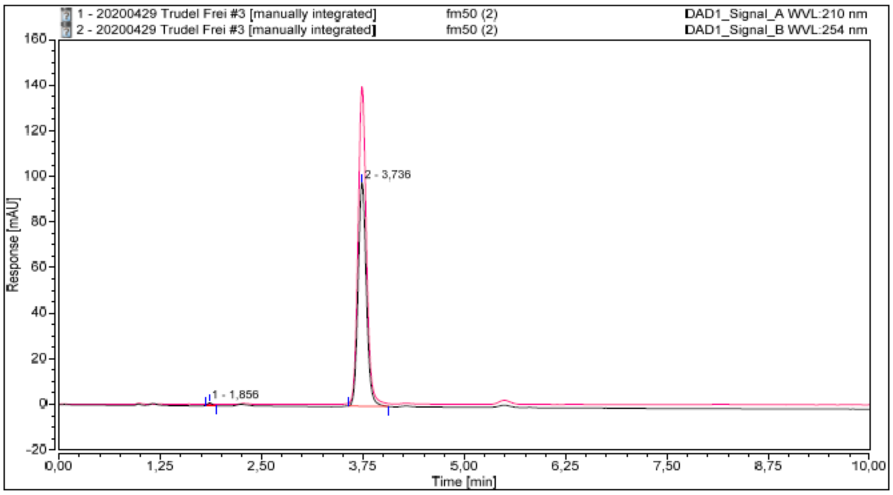

DAD1_Signal_A

No.	Retention Time min	Area mAU*min	Relative Area %
1	1,856	0,076	n.a.
2	3,736	11,432	98
Total:		11,507	98

DAD1_Signal_B

No.	Retention Time min	Area mAU*min	Relative Area %
1	3,736	16,439	98
2	5,496	0,321	2
Total:		16,760	100

HPLC chromatogram of *N*-(4-(7-(2-((4,6-Dimethylpyrimidin-2-yl)thio)acetamido)naphthalen-2-yl)phenyl)benzamide (**FM53**)

Instrument:Horst Sequence:20200305 Horst Frei

Page 1 of 1

Chromatogram and Results

Injection Details

Injection Name:	fm-53	*Run Time (min):*	9,99
Vial Number:	Vial:14	*Injection Volume:*	10,00 µL
Injection Type:	Unknown		nm
Instrument Method:	Horst 70 ACN 30 Wasser 254nm	*Wavelength:*	254 nm
Column:	Zorbax Eclipse Plus C18 4,6x 150mm, 5µm	*Flow rate:*	1,0 ml/min
Injection Date/Time:	04.Jun.20 09:58	*Column Temperatur:*	50 °C
		Pump Channel B:	100,0 70 ACN 30 Wasser

Chromatogram

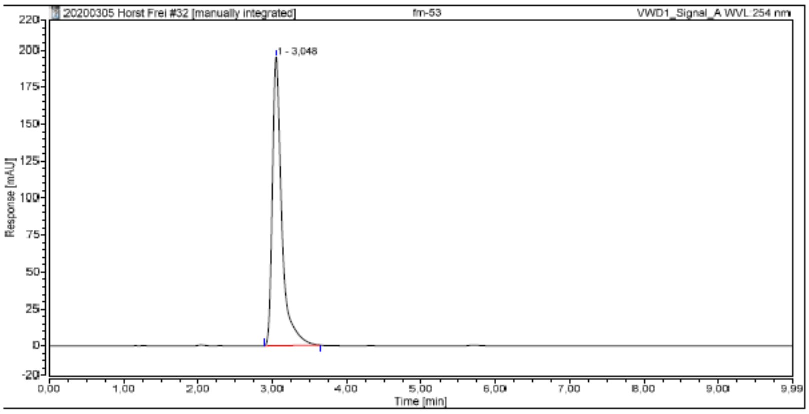

VWD1_Signal_A

No.	Retention Time min	Area mAU*min	Relative Area %
1	3,048	28,658	100,00
Total:		28,658	100

Chromatogram and Results

Injection Details

Injection Name:	fm-53	*Run Time (min):*	9,99
Vial Number:	Vial:14	*Injection Volume:*	10,00 µL
Injection Type:	Unknown		nm
Instrument Method:	Horst 70 ACN 30 Wasser 210nm	*Wavelength:*	210 nm
Column:	Zorbax Eclipse Plus C18 4,6x 150mm, 5µm	*Flow rate:*	1,0 ml/min
Injection Date/Time:	04.Jun.20 09:47	*Column Temperatur:*	50 °C
		Pump Channel B:	100,0 70 ACN 30 Wasser

Chromatogram

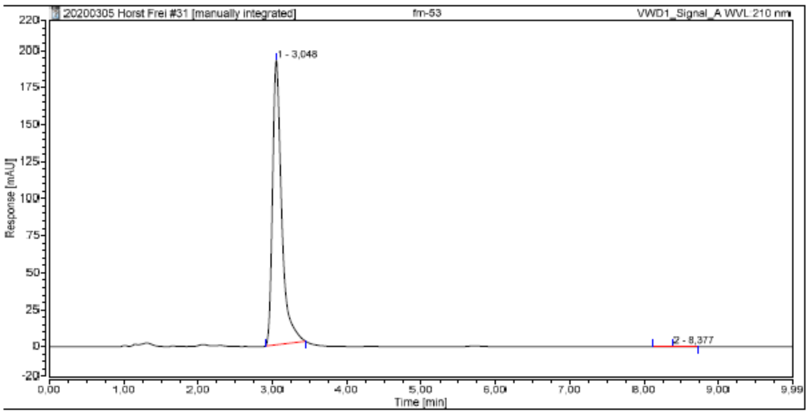

VWD1_Signal_A

No.	Retention Time min	Area mAU*min	Relative Area %
1	3,048	27,348	99,96
2	8,377	0,012	0,04
Total:		27,359	100

HPLC chromatogram of *N*-(3-(7-(2-((4,6-Dimethylpyrimidin-2-yl)thio)acetamido)naphthalen-2-yl)phenyl)benzamide (**FM54**)

Chromatogram and Results

Injection Details

Injection Name:	fm-54	Run Time (min):	9,99
Vial Number:	Vial:15	Injection Volume:	10,00 µL
Injection Type:	Unknown		nm
		Wavelength:	254 nm
Instrument Method:	Horst 70 ACN 30 Wasser 254nm	Flow rate:	1,0 ml/min
Column:	Zorbas Eclipse Plus C18 4,6x 150mm, 5µm	Column Temperatur:	50 °C
Injection Date/Time:	04.Jun.20 10:19		
		Pump Channel B:	100,0 70 ACN 30 Wasser

Chromatogram

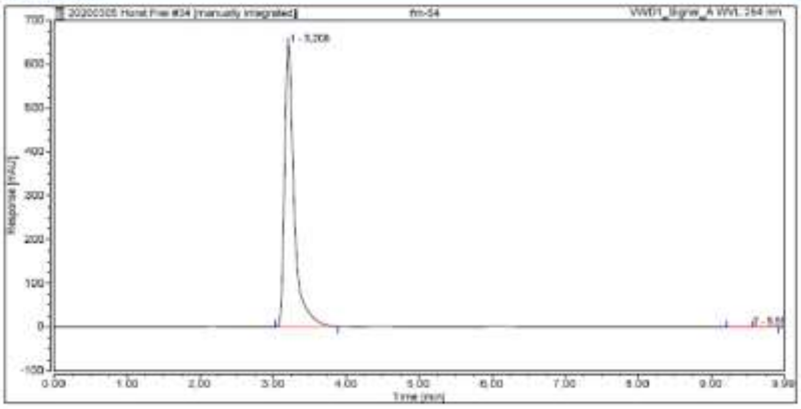

VWD1_Signal_A

No.	Retention Time min	Area mAU*min	Relative Area %
1	3,208	98,841	99,99
2	9,558	0,009	0,01
Total		98,850	100

Chromatogram and Results

Injection Details

Injection Name:	fm-54	Run Time (min):	9,99
Vial Number:	Vial:15	Injection Volume:	10,00 µL
Injection Type:	Unknown		nm
		Wavelength:	210 nm
Instrument Method:	Horst 70 ACN 30 Wasser 210nm	Flow rate:	1,0 ml/min
Column:	Zorbax Eclipse Plus C18 4,6x 150mm, 5µm	Column Temperatur:	50 °C
Injection Date/Time:	04.Jun.20 10:08		
		Pump Channel B:	100,0 70 ACN 30 Wasser

Chromatogram

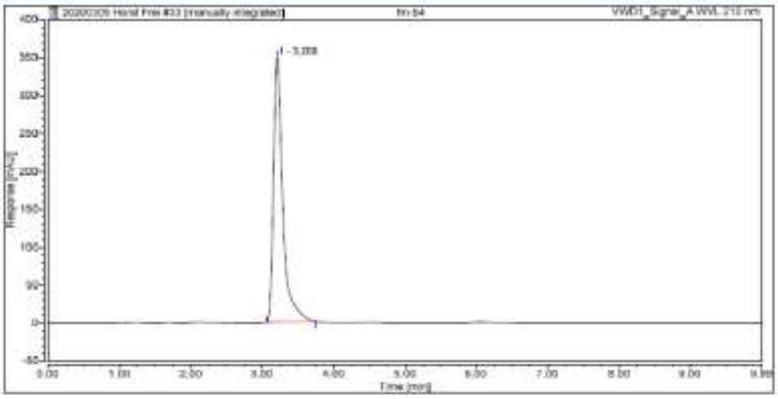

VWD1_Signal_A

No.	Retention Time min	Area mAU*min	Relative Area %
1	3,208	52,486	100,00
Total		52,486	100

HPLC chromatogram of 2-((4,6-Dimethylpyrimidin-2-yl)thio)-*N*-(7-(4 (phenylsulfonamido)phenyl)naphthalen-2-yl)acetamide (**FM56**)

Chromatogram and Results

Injection Details

Injection Name:	FM 56-1	*Run Time (min):*		9,99
Vial Number:	Vial:12	*Injection Volume:*		10,00 µL
Instrument Method:	70 ACN 30 Wasser 210nm	*Wavelength:*		210 nm
Column:	Zorbax Eclipse Plus C18 4,6x 150mm, 5µm	*Flow rate:*		1,5 ml/min
Injection Date/Time:	08.Sep.20 15:18	*Column Temperatur:*		35,0 °C
		Pump Channel A:	70,0	ACN
		Pump Channel B:		ACN

Chromatogram

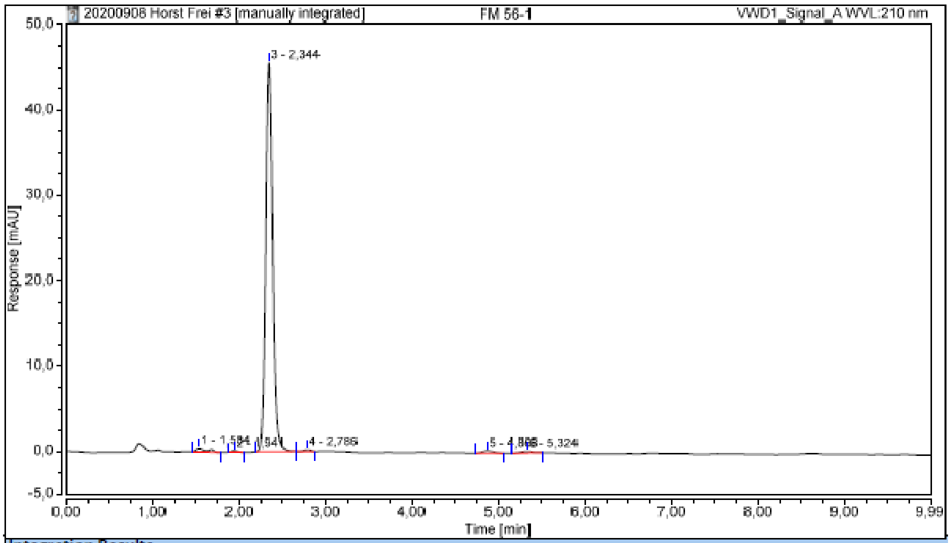

Integration Results

No.	Peak Name	Retention Time min	Area mAU*min	Relative Area %
1		1,534	0,056	1,28
2		1,941	0,012	0,27
3		2,344	4,232	96,52
4		2,786	0,016	0,37
5		4,868	0,041	0,93
6		5,324	0,027	0,62
**Total:**			**4,384**	**100,00**

HPLC chromatogram of 2-((4,6-Dimethylpyrimidin-2-yl)thio)-*N*-(7-(4-(phenylsulfonamido)phenyl)naphthalen-2-yl)acetamide (**FM56**)

Chromatogram and Results

Injection Details

Injection Name:	FM 56-1	*Run Time (min):*	9,99
Vial Number:	Vial:12	*Injection Volume:*	10,00 µL
Instrument Method:	70 ACN 30 Wasser 254nm	*Wavelength:*	254 nm
Column:	Zorbax Eclipse Plus C18 4,6x 150mm, 5µm	*Flow rate:*	1,5 ml/min
Injection Date/Time:	08.Sep.20 15:29	*Column Temperatur:*	35,0 °C
		Pump Channel A: 70,0	ACN
		Pump Channel B:	ACN

Chromatogram

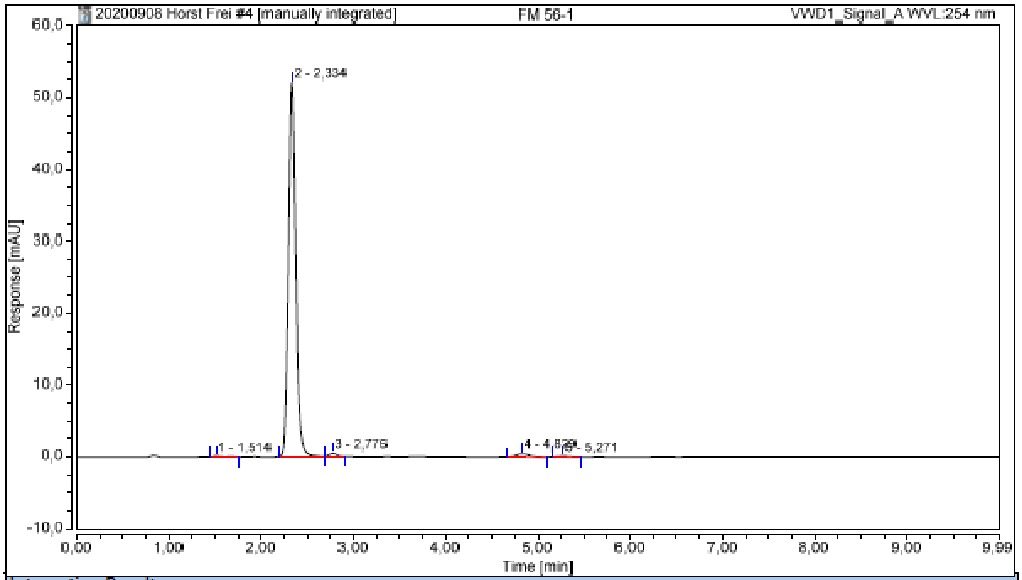

Integration Results

No.	Peak Name	Retention Time min	Area mAU*min	Relative Area %
1		1,514	0,020	0,40
2		2,334	4,774	96,95
3		2,776	0,039	0,79
4		4,829	0,074	1,49
5		5,271	0,018	0,37
Total:			4,924	100,00

HPLC chromatogram of *N*-(3-(7-(2-((4,6-Dimethylpyrimidin-2-yl)thio)acetamido)naphthalen-2-yl)phenyl)-5-methylthiophene-2-carboxamide (**FM66**)

Chromatogram and Results

Injection Details

Injection Name:	FM 66	Run Time (min):		9,99
Vial Number:	Vial:13	Injection Volume:		10,00 µL
Instrument Method:	70 ACN 30 Wasser 210nm	Wavelength:		210 nm
Column:	Zorbax Eclipse Plus C18 4,6x 150mm, 5µm	Flow rate:		1,5 ml/min
Injection Date/Time:	03.Sep.20 12:04	Column Temperatur:		35,0 °C
		Pump Channel A:	30,0	Wasser
		Pump Channel B:	70,0	ACN

Chromatogram

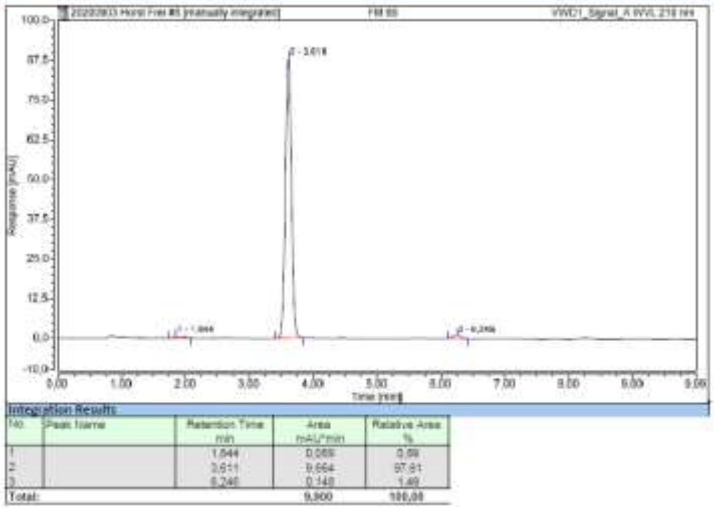

Integration Results

No.	Peak Name	Retention Time min	Area mAU*min	Relative Area %
1		1,844	0,069	0,69
2		3,611	9,664	97,81
3		6,246	0,148	1,49
Total:			9,900	100,00

Chromatogram and Results

Injection Details

Injection Name:	FM 66	Run Time (min):		9,99
Vial Number:	Vial:13	Injection Volume:		10,00 µL
Instrument Method:	70 ACN 30 Wasser 254nm	Wavelength:		254 nm
Column:	Zorbax Eclipse Plus C18 4,6x 150mm, 5µm	Flow rate:		1,5 ml/min
Injection Date/Time:	03.Sep.20 12:15	Column Temperatur:		35,0 °C
		Pump Channel A:	30,0	Wasser
		Pump Channel B:	70,0	ACN

Chromatogram

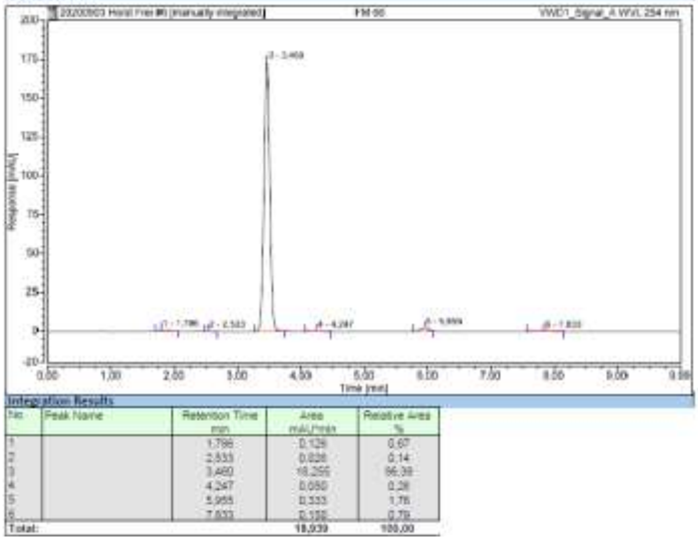

Integration Results

No.	Peak Name	Retention Time min	Area mAU*min	Relative Area %
1		1,796	0,129	0,67
2		2,533	0,028	0,14
3		3,460	18,255	96,38
4		4,247	0,050	0,26
5		5,955	0,333	1,76
6		7,833	0,150	0,79
Total:			18,939	100,00

HPLC chromatogram of *N*-(4-(7-(2-((4,6-Dimethylpyrimidin-2-yl)thio)acetamido)naphthalen-2-yl)phenyl)-5-methylthiophene-2-carboxamide (**FM69**)

Chromatogram and Results

Injection Details

Injection Name:	FM 69	*Run Time (min):*	9,99
Vial Number:	Vial:14	*Injection Volume:*	10,00 µL
Instrument Method:	70 ACN 30 Wasser 210nm	*Wavelength:*	210 nm
Column:	Zorbax Eclipse Plus C18 4,6x 150mm, 5µm	*Flow rate:*	1,5 ml/min
Injection Date/Time:	03.Sep.20 12:26	*Column Temperatur:*	35,0 °C
		Pump Channel A: 30,0	Wasser
		Pump Channel B: 70,0	ACN

Chromatogram

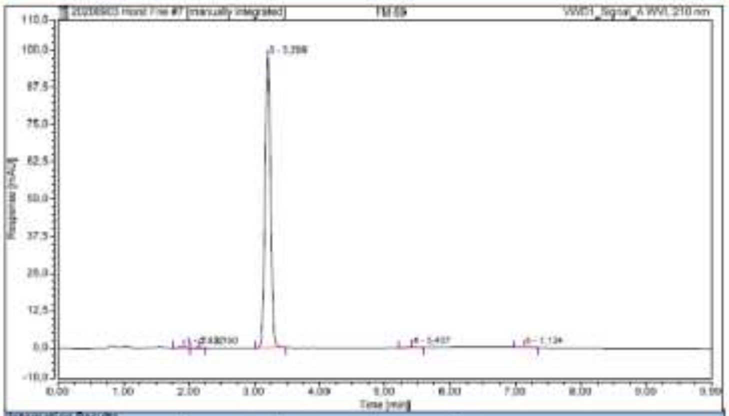

Integration Results

No.	Peak Name	Retention Time min	Area mAU*min	Relative Area %
1		1,932	0,047	0,48
2		2,150	0,031	0,31
3		3,208	9,662	98,50
4		5,407	0,029	0,29
5		7,134	0,041	0,42
Total:			9,810	100,00

Chromatogram and Results

Injection Details

Injection Name:	FM 69	*Run Time (min):*	9,99
Vial Number:	Vial:14	*Injection Volume:*	10,00 µL
Instrument Method:	70 ACN 30 Wasser 254nm	*Wavelength:*	254 nm
Column:	Zorbax Eclipse Plus C18 4,6x 150mm, 5µm	*Flow rate:*	1,5 ml/min
Injection Date/Time:	03.Sep.20 12:36	*Column Temperatur:*	35,0 °C
		Pump Channel A: 30,0	Wasser
		Pump Channel B: 70,0	ACN

Chromatogram

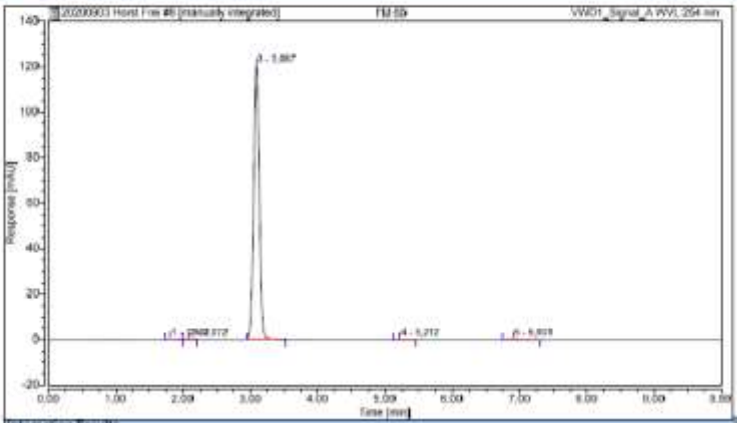

Integration Results

No.	Peak Name	Retention Time min	Area mAU*min	Relative Area %
1		1,801	0,036	0,30
2		2,072	0,015	0,12
3		3,087	11,800	98,95
4		5,212	0,024	0,20
5		6,901	0,050	0,42
Total:			11,924	100,00

HPLC chromatogram of *N*-(6-(3-Acetamidophenyl)benzo[d]thiazol-2-yl)-2-((4,6-dimethylpyrimidin-2-yl)thio)acetamide (**FM94**)

Chromatogram and Results

Injection Details

Injection Name:	fm 94 MeOH DMSO	*Run Time (min):*	14,99
Vial Number:	Vial:83	*Injection Volume:*	10 µL
Injection Type:	Unknown	*Wavelength:*	210 nm
		Wavelength:	254 nm
Instrument Method:	Trudel 50 ACN 50 Wasser	*Flow rate:*	1,0 ml/min
Column:	Eclipse Plus C18 5µm 4,6x 150mm USUXB17231	*Column Temperatur:*	30,0 °C
Injection Date/Time:	23.Apr.21 13:25	*Pump Channel A:*	
		Pump Channel B:	50 ACN 50 Wasser

Chromatogram

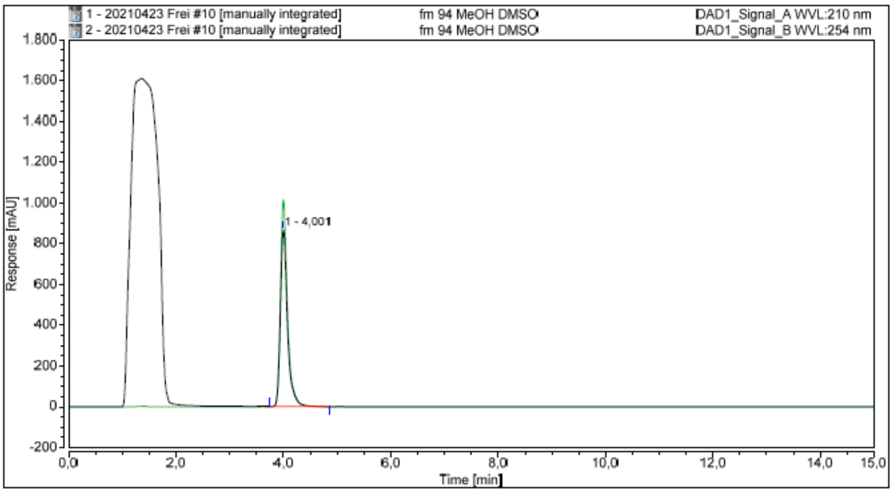

DAD1_Signal_A

No.	Retention Time min	Area mAU*min	Relative Area %
1	4,001	131,398	100,00
**Total:**		131,398	100

DAD1_Signal_B

No.	Retention Time min	Area mAU*min	Relative Area %
1	4,001	150,753	100,00
**Total:**		150,753	100

HPLC chromatogram of *N*-(3-(2-(2-((4,6-Dimethylpyrimidin-2-yl)thio)acetamido)benzo[d]thiazol-6-yl)phenyl)benzamide (**FM95**)

Chromatogram and Results

Injection Details

Injection Name:	fm 95 MeOH DMSO	*Run Time (min):*	14,99
Vial Number:	Vial:84	*Injection Volume:*	10 µL
Injection Type:	Unknown	*Wavelength:*	210 nm
		Wavelength:	254 nm
Instrument Method:	Trudel 50 ACN 50 Wasser	*Flow rate:*	1,0 ml/min
Column:	Eclipse Plus C18 5µm 4,6x 150mm USUXB17231	*Column Temperatur:*	30,0 °C
Injection Date/Time:	23.Apr.21 13:40	*Pump Channel A:*	
		Pump Channel B:	50 ACN 50 Wasser

Chromatogram

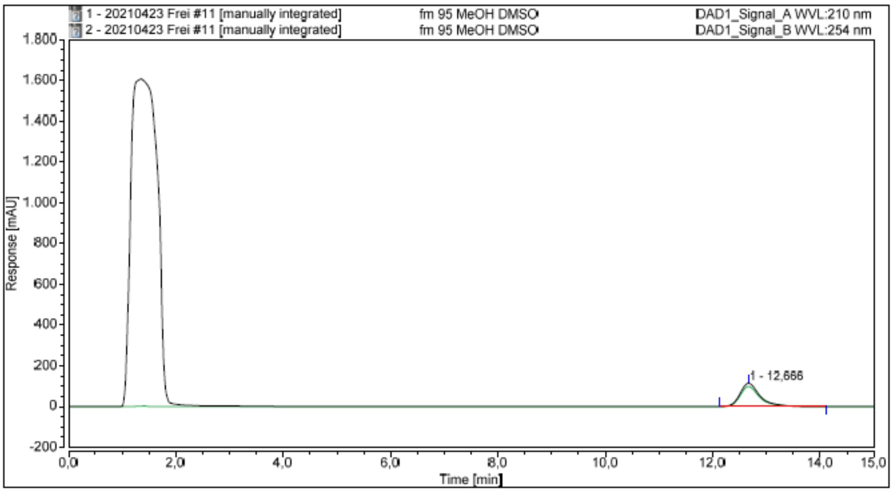

DAD1_Signal_A

No.	Retention Time min	Area mAU*min	Relative Area %
1	12,666	48,957	100,00
**Total:**		**48,957**	**100**

DAD1_Signal_B

No.	Retention Time min	Area mAU*min	Relative Area %
1	12,666	42,109	100,00
**Total:**		**42,109**	**100**

HPLC chromatogram of *N*-(3-(2-(2-((4,6-Dimethylpyrimidin-2-yl)thio)acetamido)benzo[d]thiazol-6-yl)phenyl)thiophene-2-carboxamide (**FM96**)

Instrument:Trudel Sequence:20210423 Frei

Page 1 of 1

Chromatogram and Results

Injection Details

Injection Name:	fm 96 MeOH DMSO	*Run Time (min):*	14,99
Vial Number:	Vial:85	*Injection Volume:*	10 µL
Injection Type:	Unknown	*Wavelength:*	210 nm
		Wavelength:	254 nm
Instrument Method:	Trudel 50 ACN 50 Wasser	*Flow rate:*	1,0 ml/min
Column:	Eclipse Plus C18 5µm 4,6x 150mm USUXB17231	*Column Temperatur:*	30,0 °C
Injection Date/Time:	23.Apr.21 13:56	*Pump Channel A:*	
		Pump Channel B:	50 ACN 50 Wasser

Chromatogram

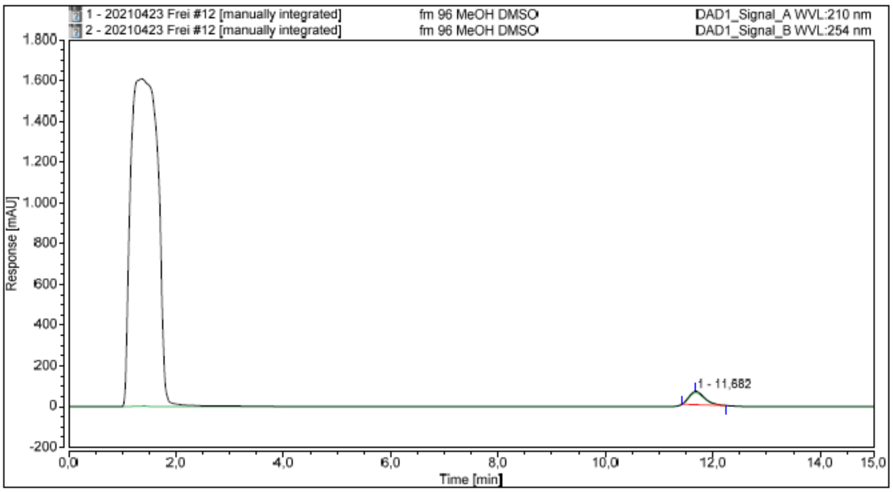

DAD1_Signal_A

No.	Retention Time min	Area mAU*min	Relative Area %
1	11,682	22,124	100,00
**Total:**		**22,124**	**100**

DAD1_Signal_B

No.	Retention Time min	Area mAU*min	Relative Area %
1	11,682	26,263	100,00
**Total:**		**26,263**	**100**

HPLC chromatogram of 2-((4,6-Dimethylpyrimidin-2-yl)thio)-*N*-(6-(3-(phenylsulfonamido)phenyl)benzo[d]thiazol-2-yl)acetamide (**FM104**)

Chromatogram and Results

Injection Details

Injection Name:	fm104 MeOH DMSO	*Run Time (min):*	14,99
Vial Number:	Vial:87	*Injection Volume:*	10 µL
Injection Type:	Unknown	*Wavelength:*	210 nm
		Wavelength:	254 nm
Instrument Method:	Trudel 50 ACN 50 Wasser	*Flow rate:*	1,0 ml/min
Column:	Eclipse Plus C18 5µm 4,6x 150mm USUXB17231	*Column Temperatur:*	30,0 °C
Injection Date/Time:	23.Apr.21 14:28	*Pump Channel A:*	
		Pump Channel B:	50 ACN 50 Wasser

Chromatogram

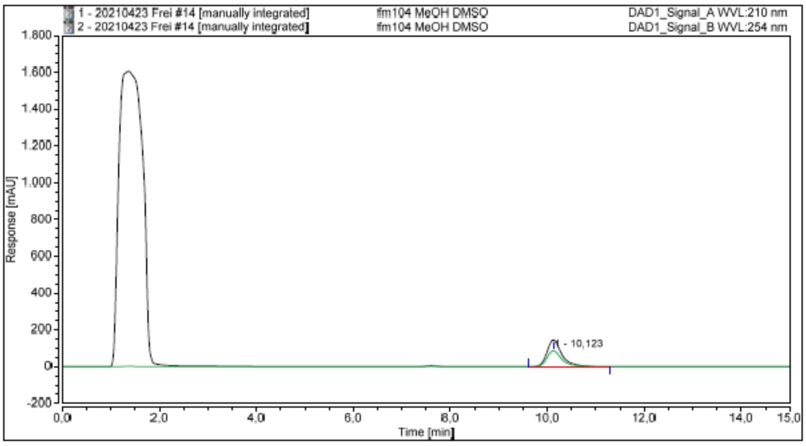

DAD1_Signal_A

No.	Retention Time min	Area mAU*min	Relative Area %
1	10,123	51,747	100,00
**Total:**		**51,747**	**100**

DAD1_Signal_B

No.	Retention Time min	Area mAU*min	Relative Area %
1	10,123	30,585	100,00
**Total:**		**30,585**	**100**

HPLC chromatogram of *N*-(3-(2-(2-((4,6-Dimethylpyrimidin-2-yl)thio)acetamido)benzo[d]thiazol-6-yl)phenyl)-5-methylthiophene-2-carboxamide (**FM108**)

Chromatogram and Results

Injection Details

Injection Name:	fm108 MeOH DMSO	*Run Time (min):*	14,99
Vial Number:	Vial:92	*Injection Volume:*	10 µL
Injection Type:	Unknown	*Wavelength:*	210 nm
		Wavelength:	254 nm
Instrument Method:	Trudel 70 ACN 30 Wasser	*Flow rate:*	1,0 ml/min
Column:	Eclipse Plus C18 5µm 4,6x 150mm USUXB17231	*Column Temperatur:*	30,0 °C
Injection Date/Time:	24.Apr.21 15:20	*Pump Channel A:*	70 ACN 30 Wasser
		Pump Channel B:	

Chromatogram

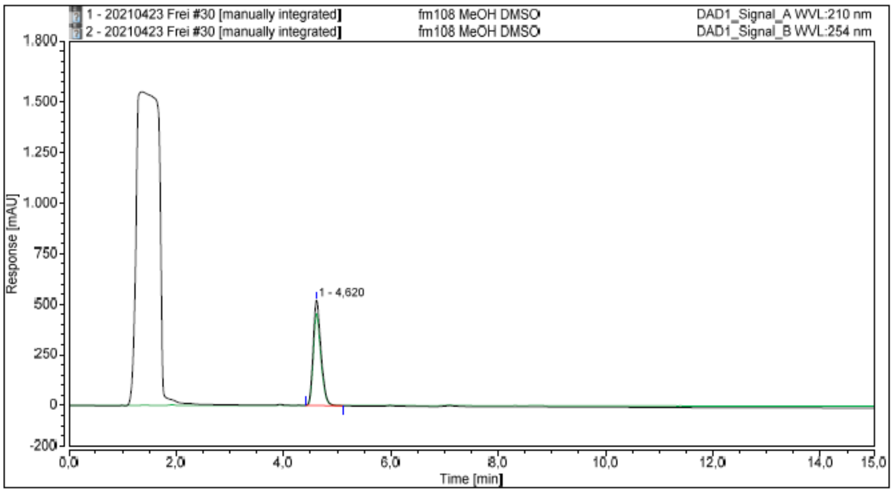

DAD1_Signal_A

No.	Retention Time min	Area mAU*min	Relative Area %
1	4,620	87,035	100,00
**Total:**		**87,035**	**100**

DAD1_Signal_B

No.	Retention Time min	Area mAU*min	Relative Area %
1	4,620	75,722	100,00
**Total:**		**75,722**	**100**

HPLC chromatogram of *N*-(4-(2-(2-((4,6-Dimethylpyrimidin-2-yl)thio)acetamido)benzo[d]thiazol-6-yl)phenyl)benzamide (**FM127**)

Chromatogram and Results

Injection Details

Injection Name:	fm127 MeOH DMSO	*Run Time (min):*	14,99
Vial Number:	Vial:89	*Injection Volume:*	10 µL
Injection Type:	Unknown	*Wavelength:*	210 nm
		Wavelength:	254 nm
Instrument Method:	Trudel 50 ACN 50 Wasser	*Flow rate:*	1,0 ml/min
Column:	Eclipse Plus C18 5µm 4,6x 150mm USUXB17231	*Column Temperatur:*	30,0 °C
Injection Date/Time:	23.Apr.21 14:59	*Pump Channel A:*	
		Pump Channel B:	50 ACN 50 Wasser

Chromatogram

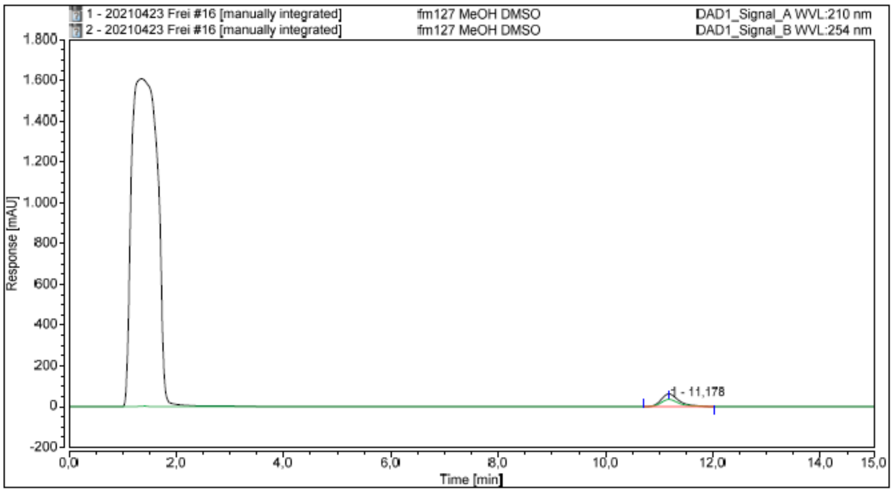

DAD1_Signal_A

No.	Retention Time min	Area mAU*min	Relative Area %
1	11,178	24,436	100,00
**Total:**		**24,436**	**100**

DAD1_Signal_B

No.	Retention Time min	Area mAU*min	Relative Area %
1	11,178	13,559	100,00
**Total:**		**13,559**	**100**

HPLC chromatogram of *N*-(6-(4-Acetamidophenyl)benzo[d]thiazol-2-yl)-2-((4,6-dimethylpyrimidin-2-yl)thio)acetamide (**FM128**)

Chromatogram and Results

Injection Details

Injection Name:	fm128 MeOH DMSO	*Run Time (min):*	14,99
Vial Number:	Vial:90	*Injection Volume:*	10 µL
Injection Type:	Unknown	*Wavelength:*	210 nm
		Wavelength:	254 nm
Instrument Method:	Trudel 50 ACN 50 Wasser	*Flow rate:*	1,0 ml/min
Column:	Eclipse Plus C18 5µm 4,6x 150mm USUXB17231	*Column Temperatur:*	30,0 °C
Injection Date/Time:	23.Apr.21 15:15	*Pump Channel A:*	
		Pump Channel B:	50 ACN 50 Wasser

Chromatogram

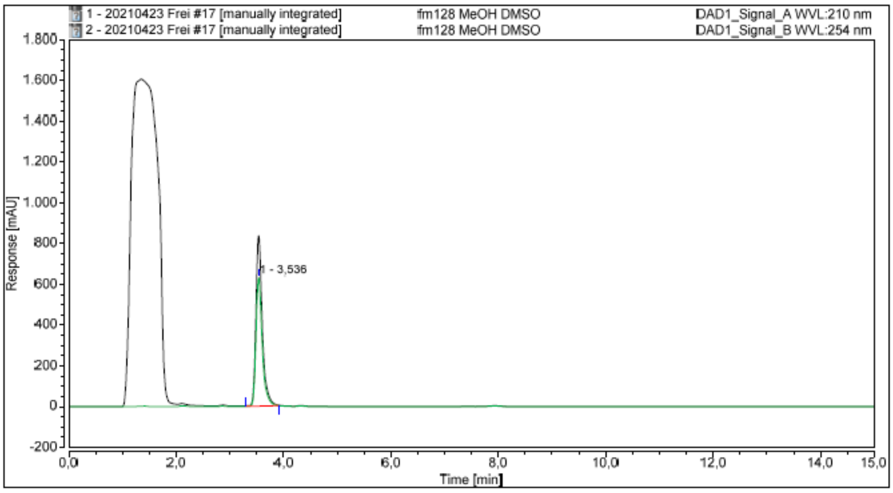

DAD1_Signal_A

No.	Retention Time min	Area mAU*min	Relative Area %
1	3,536	116,860	100,00
**Total:**		**116,860**	**100**

DAD1_Signal_B

No.	Retention Time min	Area mAU*min	Relative Area %
1	3,536	84,267	100,00
**Total:**		**84,267**	**100**

HPLC chromatogram of *N*-(4-(2-(2-((4,6-Dimethylpyrimidin-2-yl)thio)acetamido)benzo[d]thiazol-6-yl)phenyl)thiophene-2-carboxamide (**FM129**)

Chromatogram and Results

Injection Details

Injection Name:	fm 129 MeOH DMSO	*Run Time (min):*	14,99
Vial Number:	Vial:65	*Injection Volume:*	10 µL
Injection Type:	Unknown	*Wavelength:*	210 nm
		Wavelength:	254 nm
Instrument Method:	Trudel 70 ACN 30 Wasser	*Flow rate:*	1,0 ml/min
Column:	Eclipse Plus C18 5µm 4,6x 150mm USUXB17231	*Column Temperatur:*	30,0 °C
Injection Date/Time:	28.Apr.21 12:27	*Pump Channel A:*	70 ACN 30 Wasser
		Pump Channel B:	

Chromatogram

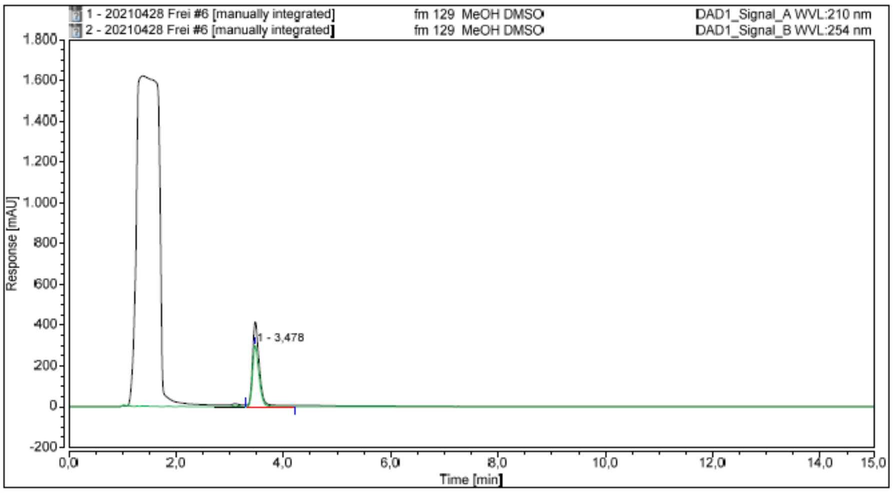

DAD1_Signal_A

No.	Retention Time min	Area mAU*min	Relative Area %
1	3,478	60,527	100,00
**Total:**		**60,527**	**100**

DAD1_Signal_B

No.	Retention Time min	Area mAU*min	Relative Area %
1	3,478	41,154	100,00
**Total:**		**41,154**	**100**

HPLC chromatogram of 2-((4,6-Dimethylpyrimidin-2-yl)thio)-*N*-(6-(3-(phenylsulfonamido)phenyl)benzo[d]thiazol-2-yl)acetamide (**FM130**)

Chromatogram and Results

Injection Details

Injection Name:	fm130 MeOH DMSO	*Run Time (min):*	14,99
Vial Number:	Vial:94	*Injection Volume:*	10 µL
Injection Type:	Unknown	*Wavelength:*	210 nm
		Wavelength:	254 nm
Instrument Method:	Trudel 50 ACN 50 Wasser	*Flow rate:*	1,0 ml/min
Column:	Eclipse Plus C18 5µm 4,6x 150mm USUXB17231	*Column Temperatur:*	30,0 °C
Injection Date/Time:	23.Apr.21 16:18	*Pump Channel A:*	
		Pump Channel B:	50 ACN 50 Wasser

Chromatogram

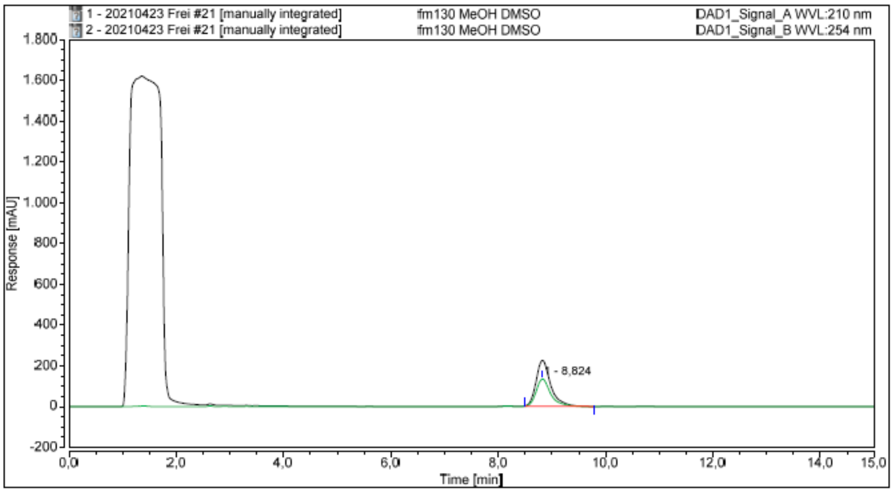

DAD1_Signal_A

No.	Retention Time min	Area mAU*min	Relative Area %
1	8,824	68,650	100,00
**Total:**		**68,650**	**100**

DAD1_Signal_B

No.	Retention Time min	Area mAU*min	Relative Area %
1	8,824	42,122	100,00
**Total:**		**42,122**	**100**

HPLC chromatogram of *N*-(4-(2-(2-((4,6-Dimethylpyrimidin-2-yl)thio)acetamido)benzo[d]thiazol-6-yl)phenyl)-5-methylthiophene-2-carboxamide (**FM131**)

Chromatogram and Results

Injection Details

Injection Name:	**fm131 MeOH DMSO**	*Run Time (min):*	**14,99**
Vial Number:	**Vial:66**	*Injection Volume:*	**10 µL**
Injection Type:	**Unknown**	*Wavelength:*	**210 nm**
		Wavelength:	**254 nm**
Instrument Method:	**Trudel 70 ACN 30 Wasser**	*Flow rate:*	**1,0 ml/min**
Column:	**Eclipse Plus C18 5µm 4,6x 150mm USUXB17231**	*Column Temperatur:*	**30,0 °C**
Injection Date/Time:	**28.Apr.21 12:45**	*Pump Channel A:*	**70 ACN 30 Wasser**
		Pump Channel B:	

Chromatogram

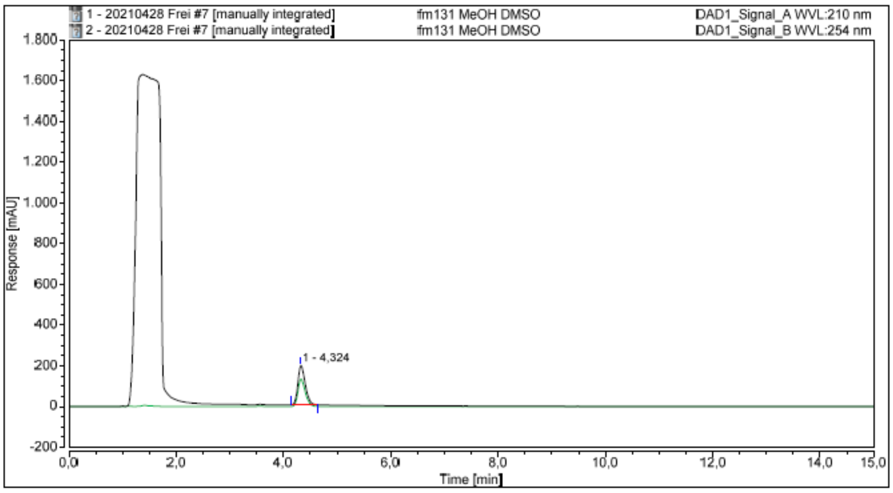

DAD1_Signal_A

No.	Retention Time min	Area mAU*min	Relative Area %
1	4,324	30,513	100,00
**Total:**		**30,513**	**100**

DAD1_Signal_B

No.	Retention Time min	Area mAU*min	Relative Area %
1	3,564	0,191	0,88
2	4,324	21,462	99,12
**Total:**		**21,652**	**100**
